# β‐Quaternary α‐Amino Acids via Iridium‐Catalyzed Branched and Enantioselective Hydroalkylation of 1,1‐Disubstituted Styrenes

**DOI:** 10.1002/anie.202504477

**Published:** 2025-05-28

**Authors:** Fenglin Hong, Yihong Wang, L. Anders Hammarback, Craig M. Robertson, John F. Bower

**Affiliations:** ^1^ Department of Chemistry University of Liverpool Crown Street Liverpool L69 7ZD UK

**Keywords:** Cross‐coupling, Enantioselectivity, Hydroalkylation, Quaternary centers, α‐Amino acids

## Abstract

A cationic Ir(I)‐complex modified with the chiral diphosphine DM‐SEGPHOS mediates the hydroalkylation of diverse α‐methyl styrenes with N‐aryl glycine derivatives. The processes occur with complete branched selectivity and high enantioselectivity. Styrenes possessing higher α‐alkyl substituents also participate to provide the targets with moderate to excellent levels of diastereoselectivity. The products are readily advanced to β‐quaternary α‐amino acids that are inaccessible or cumbersome to access by other means. In broader terms, the study demonstrates how catalyst controlled C─H additions across alkenes can be used to execute the by‐product‐free construction of contiguous acyclic trisubstituted and quaternary centers.

## Graphical abstract

A cationic Ir(I)‐complex modified with the chiral diphosphine DM‐SEGPHOS mediates the enantioselective hydroalkylation of diverse α‐substituted styrenes with N‐aryl glycine derivatives. The products are readily advanced to β‐quaternary α‐amino acids that are inaccessible or cumbersome to access by other means.

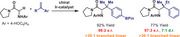

β‐Substituted α‐amino acids are challenging to access, and this issue is most pronounced for β‐quaternary variants (Scheme 1a). The simplest of these, L‐*tert*‐leucine, has been subject to extensive efforts to streamline its synthesis,^[^ ^1^ ^]^ and is now available at relatively low cost. However, synthetic accessibility becomes a major factor as β‐substitution becomes more diverse; for example, β,β‐dimethyl‐L‐phenylalanine and β,β‐dimethyltryptophan are approximately two hundred and fifty times more expensive and have limited availability, with the latter only sold as its racemate. These cost and accessibility issues reflect the limitations of current synthetic technologies and highlight the need for methods that can install the challenging α‐C(sp^3^)─C(sp^3^) bond. In particular, β,β‐dimethylated β‐quaternary amino acids are important targets,^[^ ^2^ ^]^ because these motifs have been shown to confer advantageous stability^[^ ^2^ ^]^ or conformational properties when incorporated into peptides.^[^ ^3^ ^]^

**Scheme 1:**
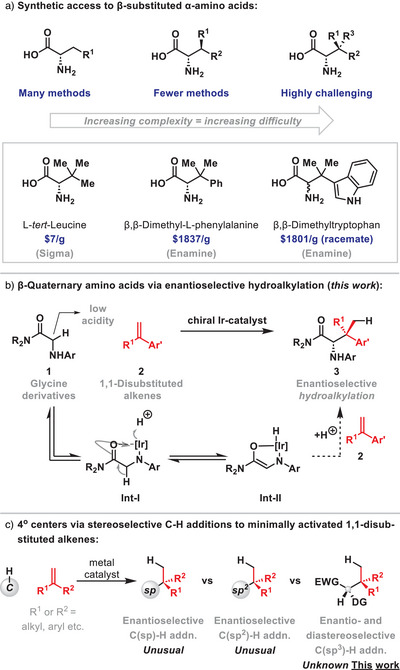
Introduction.

Recently, we outlined a new entry to complex α‐amino acids via the Ir‐catalyzed cross‐coupling of N‐aryl glycine derivatives **1** with minimally activated monosubstituted alkenes **2** (R^1^ = H) (Scheme 1b).^[^ ^4^ ^]^ These processes are predicated on a sequence of NH‐metalation (to **Int‐I**) in advance of soft enolization to generate geometrically defined Ir‐enolate **Int‐II**. Engagement of the alkene (R^1^ = H) then occurs in a branched selective manner to provide the targets. To address the synthetic issues outlined above, we questioned whether 1,1‐disubstituted styrenes (R^1^ ≠ H) could be accommodated in these processes. At the outset, this proposition was considered challenging because (a) 1,1‐disubstituted styrenes are not commonly tolerated in transition metal catalyzed C(sp^3^)─H addition reactions,^[^ ^5^, ^6^, ^7^ ^]^ (b) the target C─C bond is very hindered, (c) it was unclear whether high branched selectivity could be achieved, and (d), for R^1^ > Me, control of facial selectivity is required with respect to both **Int‐II** and **2**. Nevertheless, as outlined below, we have found that the approach is indeed feasible, providing easy access to previously challenging β‐quaternary α‐amino acids. From the viewpoint of alkene hydrofunctionalization, the processes are significant because they involve the regio‐, diastereo‐, and enantiocontrolled addition of a C(sp^3^)─H bond across a minimally activated 1,1‐disubstituted alkene to generate contiguous acyclic tertiary and quaternary centers (Scheme 1c).^[^ ^5^, ^6^, ^7^ ^]^ Although unusual, acyclic quaternary centers can be accessed by the stereoselective intermolecular addition of C(sp)─H or C(sp^2^)─H bonds to minimally activated 1,1‐disubstituted alkenes under Ir‐ or Ni‐catalyzed conditions.^[^ ^8^, ^9^, ^10^, ^11^, ^12^, ^13^, ^14^, ^15^, ^16^, ^17^, ^18^ ^]^ Nevertheless, to the best of our knowledge, stereocontrolled processes involving C(sp^3^)─H bonds have not been reported, with the most sophisticated examples described below being especially notable because high facial selectivity is achieved with respect to both **Int‐II** and **2**.

In early experiments toward the target process, we explored the hydroalkylative cross‐coupling of α‐amino amide **1a** with α‐methyl styrene **2a** (600 mol%) (Table 1). The selection of an N‐aryl group on **1a** was based on our earlier work involving monosubstituted styrenes, where this motif had been shown to be a critical requirement.^[^ ^4^ ^]^ By using the combination of [Ir(cod)_2_]BARF and **L1** (7.5 mol%) at 110 °C in 1,4‐dioxane, we were able to generate target **3aa** in 70% yield and 94.5:5.5 e.r. (Entry 1), and, importantly, with complete branched selectivity.^[^ ^19^, ^20^ ^]^ Under these conditions, other homochiral diphosphine ligands **L2‐10** were explored (Entries 2–10), and this revealed that several systems could provide reasonable chemical efficiency and enantioselectivity. The best balance was achieved with **L2** and so this ligand was advanced through further optimization studies that focused on the choice of precatalyst and solvent (Entries 11–14). This established that [Ir(cod)_2_]BARF is the optimal precatalyst and that the reaction can be conducted in many different solvents (see the Supporting Information). Ultimately, these studies resulted in the conditions in Entry 15 that use [Ir(cod)_2_]BARF/**L2** in *t*‐BuOH and provide **3aa** in 74% isolated yield (80% NMR yield) and 97:3 e.r. with 400 mol% of **2a**. Under these conditions, the loading of the alkene could be decreased to 300 mol% with only a minor impact on yield (Entry 16), whereas higher temperatures were detrimental (see the Supporting Information).

**Table 1:** Reaction optimization.^a)^

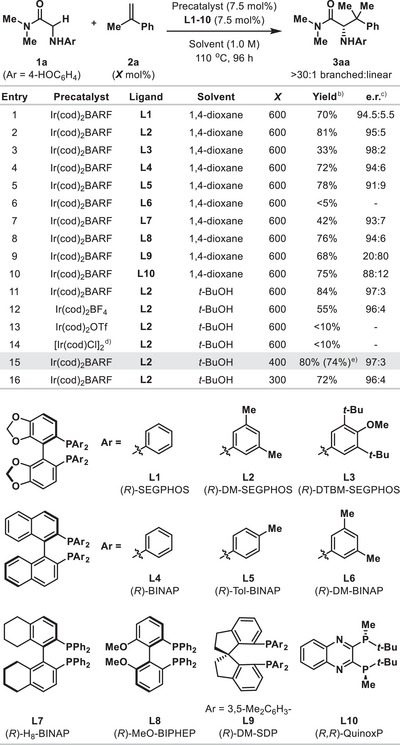

^a)^Further optimization results, including the evaluation of other ligands, are given in the Supporting Information.

^b)^Yields were measured by ^1^H NMR analysis using 1,3,5‐trimethoxybenzene as an internal standard.

^c)^Determined by chiral SFC analysis.

^d)^3.75 mol% of this dimeric precatalyst was used.

^e)^Isolated yield. [BARF = tetrakis(3,5‐bis(trifluoromethyl)phenyl)borate; cod = 1,5‐cyclooctadiene].

The optimized conditions from Table 1 were taken forward to establish working parameters for the pronucleophilic component, with the specific aim of identifying the most suitable structural variant for scope studies with respect to the alkene (Scheme 2). We have previously conducted an exhaustive evaluation of the limitations of the N‐aryl unit for processes involving monosubstituted alkenes.^[^ ^4^ ^]^ Accordingly, we focused only on variants that were deemed likely to give efficient reactivity, and, in the event, we established that the electronics of this unit have a relatively small influence on yield and enantioselectivity, as evidenced by the results obtained for **3aa–3ga** (Scheme 2a). From a synthetic viewpoint, the most notable point of difference was between N‐4‐hydroxy‐ and N‐4‐methoxyphenyl systems **1a** and **1b**, which both provide products that can easily be deprotected under oxidative conditions.^[^ ^21^ ^]^ The former was appreciably more effective, offering advantages in terms of yield and enantioselectivity. Accordingly, the N‐4‐hydroxyphenyl unit was retained for the evaluation of different amide units (**1h–1l**) (Scheme 2b). Although these processes were not rigorously optimized in each case, it was apparent that pyrrolidine amide **1i** was the most efficient pronucleophile, and cross‐coupling of this with **2a** provided **3ia** in 86% isolated yield (92% NMR yield) and 97:3 e.r. During these studies we also established that ketones (**1m**) and esters (**1n**) are not suitable. The former did participate but offered minimal enantiocontrol for the formation of **3ma**, whereas ester‐based target **3na** was not observed.^[^ ^22^ ^]^

**Scheme 2:**
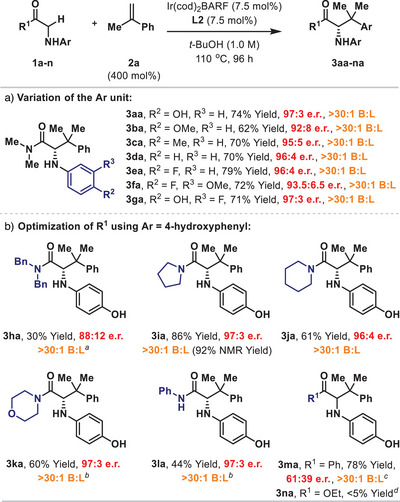
Evaluation of the pronucleophile. *^a^* **2a** (1000 mol%), **L2** (10 mol%), and [Ir(cod)_2_]BARF (10 mol%) were used. *^b^* The reaction concentration was 0.5 M. *^c^* **L9** was used as the ligand (45 h). *^d^* ***rac.‐*L4** was used as the ligand.

Based on the results above, the scope of the process with respect to the alkene was evaluated using **1i**, with an initial focus on α‐methylated styrenes (Scheme 3a). In general, *p*‐substituted systems were found to be excellent cross‐coupling partners, with generally high yields and enantioselectivities observed for **3ib–ii**. Notably, no strong electronic effects were identified with, for example, similar efficiencies observed for the conversion of 4‐methoxy system **2c** to **3ic** and 4‐fluoro system **2e** to **3ie**. Boronic esters (**3ii**) and aryl chlorides (**3if**) can be transferred, whereas diminished yields were observed for unprotected phenol **3id** and aryl bromide **3ig**. For the latter, competitive oxidative addition of the C(sp^2^)─Br bond of **2g** may attenuate catalysis. In general, styrenic systems possessing electron rich (hetero)aromatic units proved to be excellent substrates, with high yields observed for the formation of 2‐naphthyl (**3im**), benzofuryl (**3in** and **3ip**), 3‐indolyl (**3io**), and 3‐thienyl (**3iq**) adducts. Estrone derivative **2r** provided **3ir** with excellent levels of catalyst enforced diastereocontrol. At the present level of development, 1,1‐dialkylated alkenes and β‐substituted styrenes are not suitable reaction partners (see the Supporting Information: Scheme S1), and studies are ongoing to address these limitations.

**Scheme 3:**
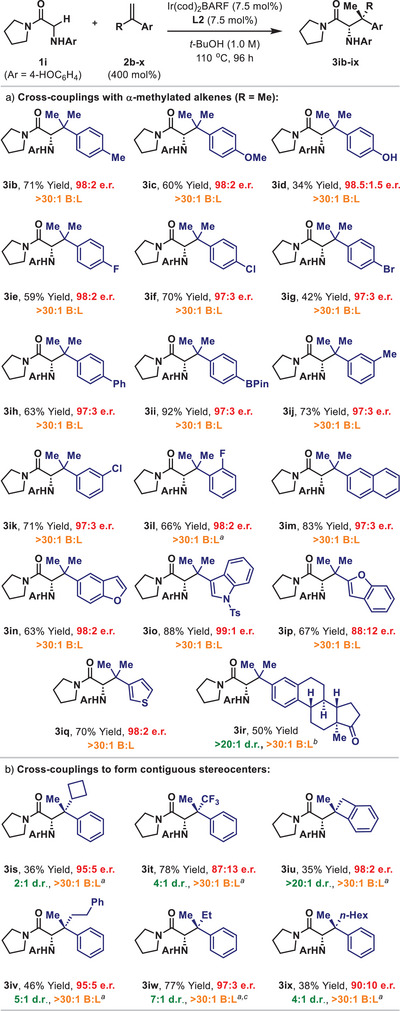
Scope of the alkene. *^a^* Alkene (1000 mol%), **L2** (10 mol%), and [Ir(cod)_2_]BARF (10 mol%) were used. *^b^* The reaction concentration was 0.5 M. *^c^* The absolute and relative stereochemistries were assigned by X‐ray analysis of a derivative (see Scheme 4a).

The high efficiencies observed in Scheme 3a raised the question of whether the method could tolerate styrenes possessing higher α‐alkyl substituents (i.e., R ≠ Me). This avenue was especially appealing because it would allow the direct installation of vicinal α‐trisubstituted and β‐quaternary stereocenters *under by‐product‐free conditions*. Existing cross‐coupling methods that achieve related enantioselective bond formations at the amide α‐position are dominated by Ni‐catalyzed processes, including cross‐couplings of organohalides with organozinc reagents,^[^ ^23^, ^24^, ^25^ ^]^ doubly decarboxylative cross‐couplings of NHPI‐esters,^[^ ^26^ ^]^ and reductive cross‐couplings of α‐halo or ‐pyridinium amides with olefins.^[^ ^27^, ^28^, ^29^ ^]^ Wang and Xu's Cu‐catalyzed protocol for the alkylation of N‐8‐quinolinyl glycine esters is also of relevance to this study.^[^ ^30^ ^]^ Each of these methods requires significant levels of prefunctionalization, and none of them provide a solution to controlling the installation of vicinal α‐trisubstituted and β‐quaternary stereocenters. For example, in contrast to the method here, prior processes that use alkenes as the coupling‐partner deliver linear selectivities,^[^ ^27^, ^28^, ^29^ ^]^ whereas Wang and Xu's method can install β‐quaternary centers but does not offer stereocontrol at this position.^[^ ^30^ ^]^

In the event, we found that the target processes, although challenging, could indeed be achieved. For example, α‐cyclobutyl styrene **2s** cross‐coupled with **1i** to provide **3is** in modest yield and diastereoselectivity, but, importantly, with excellent enantioselectivity. For other systems, diastereoselectivities were higher (4:1 to > 20:1 d.r.), and yields ranged from moderate to excellent. Perhaps most notably, α‐ethyl styrene **2w** provided **3iw**, which possesses a quaternary stereocenter defined by ethyl–methyl substitution, in 77% yield, 7:1 d.r., and 97:3 e.r. An electron withdrawing α‐CF_3_ unit (**3it**) and a cyclic system (**3iu**) were also competent partners, thereby demonstrating promising preliminary scope for this approach. Clearly, further development is required, but, even as they stand, the results significantly expand the horizons of this Ir‐catalyzed hydroalkylation approach. Importantly, for all cases in Scheme 3b complete branched selectivity was still observed, and high enantioselectivities were maintained, even across sterically diverse alkene reaction partners.

Scheme 4 outlines derivatization of the products described here. Firstly, oxidative deprotection of **3iw** with PIFA provided amine **4** efficiently, and this was converted to *p*‐bromobenzoyl derivative **5** in 67% yield (2 steps) (Scheme 4a). This compound was characterized by single crystal X‐ray diffraction, and this provided the basis for the absolute and relative stereochemical assignments elsewhere.^[^ ^31^ ^]^ To validate the applicability of the cross‐coupling method to the production of β‐tetrasubstituted α‐amino acids, we developed a high yielding two‐step conversion of **3aa** to β,β‐dimethyl‐phenylalanine **7** (Scheme 4b). Reduction of this to amino alcohol **8** and subsequent chiral SFC analysis revealed almost complete retention of enantiopurity during the conversion of **6** to **7**. Previously reported routes to **7** are cumbersome (see the Supporting Information for details), but, even so, this amino acid has still found use in medicinal chemistry; for example, it is a subunit of the potent antimicrotubule agent HTI‐286, which is a synthetic analogue of the tripeptide hemiasterlin.^[^ ^32^ ^]^ To demonstrate applicability in other areas, we removed the N‐aryl unit from **3im** to provide a known precursor of a chiral N,O‐ligand; this intermediate has previously been prepared in 8 steps (see the Supporting Information for details) versus the 3 step sequence outlined here (Scheme 4c).^[^ ^33^, ^34^ ^]^

**Scheme 4:**
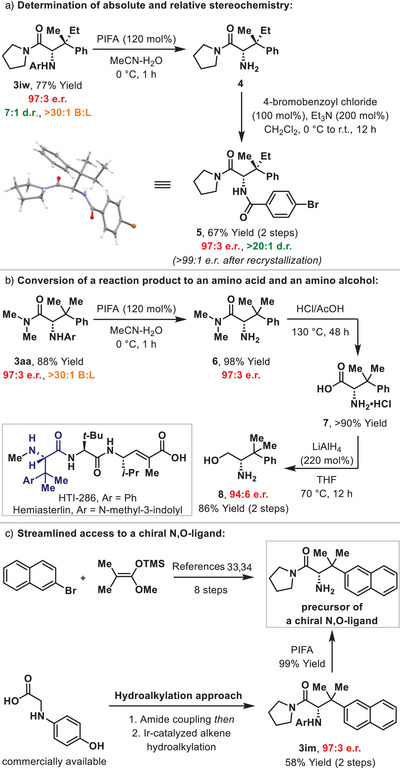
X‐ray analysis and the utility of the process.

With respect to mechanism, the conversion of **1** to **Int‐I** and **Int‐II** (see Scheme 1b) has been addressed in our earlier work.^[^ ^4^ ^]^ Specifically, amino‐directed Rh‐catalyzed cyclopropane C─C bond activations offer good precedent for the NH metalation step.^[^ ^35^ ^]^ The heightened acidity of the α‐C─H of **Int‐I** upon coordination of the carbonyl to the Ir‐center is supported by studies on the facile enolization of Rh‐ and Ir‐β‐keto phosphine complexes.^[^ ^36^ ^]^ As depicted, for the processes here, this enolization process may also be driven by oxidation of the Ir‐center. At present, the mechanistic details for the conversion of **Int‐II** and **2** to **3** are uncertain, and detailed studies are ongoing. Preliminary experiments indicate a first order dependency on [Ir] for the reaction of **1d** with **2a** to give **3da**.^[^ ^37^ ^]^ Accordingly, an inner sphere carbometallation process may be operative, wherein **Int‐III** is converted to **Int‐IV** (Scheme 5). For the latter, the amide unit may or may not be coordinated to the Ir‐center. This mechanism for C─C bond formation mirrors our previous proposition for heteroaryl directed processes,^[^ ^38^ ^]^ and, although unusual, finds precedent in stoichiometric studies involving the reaction of Ir(acac) complexes with alkynes.^[^ ^39^, ^40^ ^]^ The branched selectivity may reflect a preference for the bulky Ir‐center to move to the less hindered end of the alkene.

**Scheme 5:**
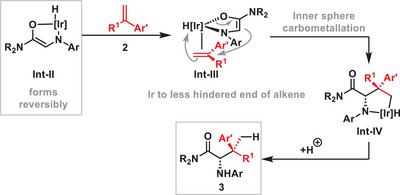
Mechanistic considerations.

In summary, we demonstrate a protocol for the regio‐, diastereo‐, and enantioselective cross‐coupling of glycine derivatives with α‐substituted styrenes. This provides a short entry to β‐quaternary α‐amino acids that are difficult to access or unavailable using other methods. Given the importance of *tert*‐leucine in, for example, chiral ligands^[^ ^1^, ^2^, ^3^, ^41^, ^42^, ^43^ ^]^ and peptide design,^[^ ^44^ ^]^ we suggest that the method described here will be of broad interest. In particular, the β,β‐dimethylated products described here appear to be primed for applications toward peptide therapeutics.^[^ ^3^ ^]^ From the viewpoint of reactivity, this study makes significant contributions to both alkene hydrocarbonation processes and the chemistry of DG‐generated Ir‐enolates. The latter have barely been exploited in enantioselective reaction design,^[^ ^4^, ^38^, ^45^ ^]^ yet evidently offer great potential for underpinning stereocontrolled and by‐product‐free C─C cross‐couplings that adhere to the green chemistry principles of high atom and step economy.^[^ ^46^, ^47^ ^]^ Studies to advance this avenue are ongoing and will be reported in due course.

## Conflict of Interests

The authors declare no conflicts of interest.

## Supporting information



Supporting Information

## Data Availability

The data that support the findings of this study are available in the Supporting Information of this article.
