# Supplementary material for: β‐Quaternary α‐Amino Acids via Iridium‐Catalyzed Branched and Enantioselective Hydroalkylation of 1,1‐Disubstituted Styrenes
Source: Angew Chem Int Ed Engl. 2025 May 28;64(26):e202504477. doi: 10.1002/anie.202504477 (PMC12184314; doi:10.1002/anie.202504477)

β-Quaternary α-Amino Acids via Iridium-Catalyzed Branched and Enantioselective Hydroalkylation of 1,1-Disubstituted Styrenes

Fenglin Hong, Yihong Wang, L. Anders Hammarback, Craig M. Robertson, and John F. Bower*

Department of Chemistry, University of Liverpool, Crown Street, Liverpool, L69 7ZD, United Kingdom

Supporting Information

Table of Contents

Contents

General experimental details ... 2
Experimental procedures and data ... 3
  Additional optimization studies ... 3
  Substrate synthesis ... 4
  Reaction scope ... 11
  Evaluation of the optimized protocol on a monosubstituted styrene ... 58
  Scheme S1 ... 58
  Applications and derivatizations ... 59
  Synthetic approach to 7 from the literature ... 65
  Synthetic approach to chiral ligand 10 from the literature ... 67
Mechanistic studies ... 69
  Figure S1: Kinetic experiments ... 69
References ... 71
NMR Spectra ... 73

General experimental details

All reagents requiring purification were purified using standard laboratory techniques according to methods published by Armarego, and Perrin (Pergamon Press, 1966). Catalytic reactions were carried out in Young-type re-sealable tubes. Styrene and other commercially available alkenes (liquid) were quickly distilled using a Hickman distilling head before use. All other commercially available alkenes (solid) were used as received without any further purification. Iridium catalysts were synthesized according to previously reported procedures[1,2]. Anhydrous THF, toluene, PhCl and CH_2Cl_2 (DCM) were obtained by either passed through drying columns supplied by Anhydrous Engineering Ltd or purchased from commercial sources (Acros or Aldrich). Anhydrous *t*-BuOH, *n*-Bu_2O, 1,4-dioxane, DMF and CH_3CN were purchased as anhydrous grade and stored over activated 4Å molecular sieves prior to use. All reactions were performed using dry solvents unless stated otherwise. Triethylamine (TEA) was distilled over CaH_2 and stored over activated 4Å molecular sieves under nitrogen. Flash column chromatography (FCC) was performed using silica gel (Aldrich 40-63 µm, 230-400 mesh). Thin layer chromatography was performed using aluminium backed 60 F_{254} silica plates. Visualisation was achieved by UV fluorescence or a basic $KMnO_4$ solution and heat. Proton nuclear magnetic resonance spectra (NMR) were recorded at 400 MHz or 500 MHz as stated. ^{13}C NMR spectra were recorded at 125 MHz as stated. Chemical shifts (δ) are given in parts per million (ppm). Peaks are described as singlets (s), doublets (d), triplets (t), quartets (q), septets (sept), multiplets (m) and broad (br.). Coupling constants (J) are quoted to the nearest 0.5 Hz. When compounds were isolated as a mixture of diastereoisomers, they are referred to as a (major) and b (minor). *In situ* yields were determined by employing 1,3,5-trimethoxybenzene as the internal standard. High resolution mass spectra were determined by the University of Liverpool mass spectrometry service, given to four decimal places. Mass spectra were recorded on Agilent 7200 Accurate Mass QTOF GC/MS (under condition of chemical ionization-CI) and Agilent 6540 UHD Accurate Mass Q-TOF LC/MS (under condition of electrospray ionization-ESI). Infrared spectra were recorded on a Perkin Elmer Spectrum Two FTIR spectrometer as thin films or solids compressed on a diamond plate. Melting points were determined using Reichert melting point apparatus and are uncorrected. Optical rotations were measured using an ADP440$^+$ polarimeter at the concentration and temperature stated. Enantiomeric excesses were determined using an Agilent 1290 Infinity chiral SFC as stated for each compound.

Experimental procedures and data

Additional optimization studies

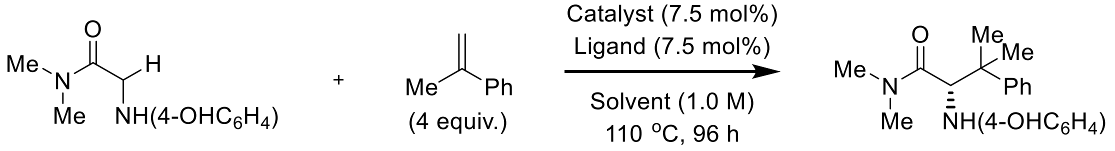

Entry	Ligand	Catalyst	Solvent	Yield[a]	e.r.[b]
1	**L2**	$Ir(cod)_2BARF$	toluene	46%	95:5
2	"	"	PhCl	80%	95:5
3	"	"	THF	76%	95:5
4	"	"	*n*-Bu_2O	43%	95:5
5	"	"	CH_3CN	<5%	-
6[c]	"	"	*t*-BuOH	72%	95:5
7	**L11**	"	"	24%	82:18
8	**L12**	"	"	<5%	-

Reaction conditions: **1a** (0.1 mmol), catalyst (7.5 μmol), **L** (7.5 μmol), prop-1-en-2-ylbenzene (0.4 mmol), solvent (0.1 mL), 110 °C, 96 h, in Schlenk tubes. [a]Measured by ^{1}H NMR using 1,3,5-trimethoxybenzene as the internal standard. [b]Determined by chiral SFC analysis. [c]The reaction was performed at 130 °C.

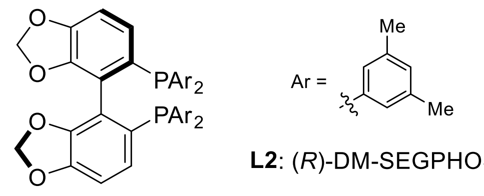

L2: (*R*)-DM-SEGPHOS

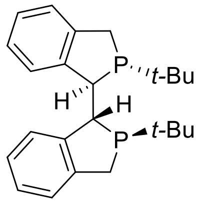

L11: (1*R*,1'*R*,2*S*,2'*S*)-DuanPhos

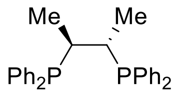

L12: (*S*,*S*)-Chiraphos

Substrate synthesis

Amides **1a-f** and **1k-l** have previously been prepared in our lab.[3]

General procedure A for the synthesis of amides 1g-j:

Amides **1g-j** were synthesized according to a modified procedure.[4]

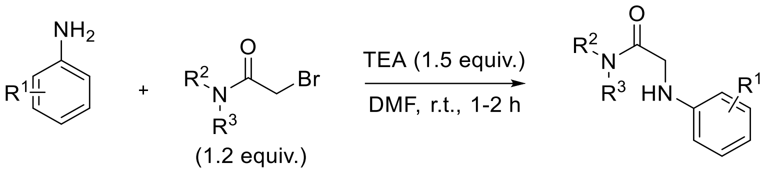

An oven-dried round bottom flask was charged with aniline (1.00 mmol, 100 mol%), TEA (0.21 mL, 1.50 mmol, 150 mol%) and anhydrous DMF (5 mL). α-Bromoamide (1.20 mmol, 120 mol%) in anhydrous DMF (5 mL) was then added dropwise over 5 minutes at room temperature (r.t.). After the addition was complete, the reaction mixture was stirred for 1-2 hour(s) at r.t. and the progress of the reaction was monitored by TLC. Upon completion, the reaction mixture was transferred to a separatory funnel, and brine (approx. 20 mL) was added. The aqueous phase was extracted with EtOAc (approx. 3 × 10 mL). The combined organic phases were dried over anhydrous $MgSO_4$, filtered and concentrated *in vacuo*. The residue was purified by FCC under the conditions noted.

2-((3-Fluoro-4-hydroxyphenyl)amino)-*N,N*-dimethylacetamide (1g):

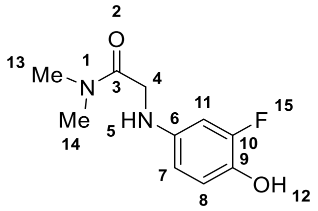

General procedure A: Purification by column chromatography (hexane/EtOAc, 50:50) afforded the title compound (95.4 mg, 45%) as a colorless solid. **m.p.** = 146 – 148 °C (hexane/EtOAc); **IR (thin film)** ν_{max}/cm^{-1}: 3218 (br), 1640 (s), 1520 (s), 1404 (s), 1205 (s); **^{1}H NMR** (500 MHz, CD_3OD) δ 6.77 – 6.73 (m, 1H, H^8), 6.49 – 6.45 (m, 1H, H^{11}), 6.36 – 6.34 (m, 1H, H^7), 3.88 (s, 2H, H^4), 3.07 (s, 3H, H^{13}), 2.98 (s, 3H, H^{14}); **^{13}C NMR** (126 MHz, CD_3OD) δ 170.6 (C^3), 152.1 (d, J = 236.3 Hz, C^{10}), 142.1 (d, J = 8.8 Hz, C^6), 135.9 (d, J = 13.8 Hz, C^9), 118.1 (d, J = 3.8 Hz, C^8), 108.9 (d, J = 2.5 Hz, C^7), 101.3 (d, J = 22.5 Hz, C^{11}), 45.8 (C^4), 35.1 (C^{13}), 34.5 (C^{14}); ^{19}F NMR (471 MHz, CD_3OD) δ -138.2; **HRMS** (ESl): calculated for $C_{10}H_{14}FN_2O_2$ $[M+H]^+$ requires *m/z* 213.1034, found *m/z* 213.1036.

***N,N*-Dibenzyl-2-((4-hydroxyphenyl)amino)acetamide (1h):**

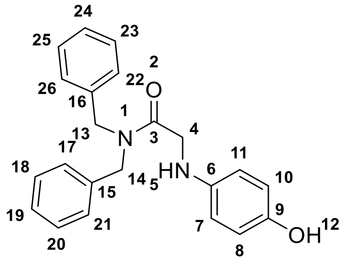

General procedure A: Purification by column chromatography (hexane/EtOAc, 60:40) afforded the title compound (121 mg, 35%) as a colorless solid. **m.p.** = 148 – 150 °C (hexane/EtOAc); **IR (thin film)** ν_{max}/cm^{-1}: 3368 (br), 1642 (s), 1516 (s), 1496 (s), 1220 (s); **^{1}H NMR** (500 MHz, CD_3OD) δ 7.39 – 7.21 (m, 10H, H^{17-21} + H^{22-26}), 6.65 (d, *J* = 8.5 Hz, 2H, H^8 + H^{10}), 6.56 (d, *J* = 8.5 Hz, 2H, H^7 + H^{11}), 4.64 (s, 2H, H^{13}), 4.59 (s, 2H, H^{14}), 4.00 (s, 2H, H^4); **^{13}C NMR** (126 MHz, CD_3OD) δ 171.7 (C^3), 149.6 (C^9), 141.1 (C^6), 136.8 (C^{15}), 136.3 (C^{16}), 128.6 (C^{18} + C^{20}), 128.3 (C^{23} + C^{25}), 127.7 (C^{17} + C^{21}), 127.4 (C^{19}), 127.1 (C^{24}), 126.5 (C^{22} + C^{26}), 115.5 (C^8 + C^{10}), 115.1 (C^7 + C^{11}), 49.2 (C^{13}), 48.7 (C^{14}), 46.8 (C^4); **HRMS** (ESI): calculated for $C_{22}H_{23}N_2O_2$ $[M+H]^+$ requires *m/z* 347.1754, found *m/z* 347.1760.

2-((4-Hydroxyphenyl)amino)-1-(pyrrolidin-1-yl)ethan-1-one (1i):

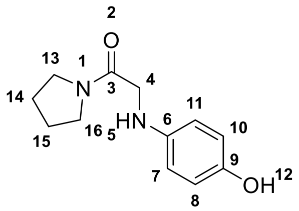

General procedure A: Purification by column chromatography (hexane/EtOAc, 50:50) afforded the title compound (143 mg, 65%) as a pale yellow solid. **m.p.** = 180 – 182 °C (hexane/EtOAc); **IR (thin film)** ν_{max}/cm^{-1}: 3348 (br), 1633 (s), 1517 (s), 1443 (s), 1245 (s); **^{1}H NMR** (500 MHz, CD_3OD) δ 6.66 (d, *J* = 9.0 Hz, 2H, H^8 + H^{10}), 6.60 (d, *J* = 9.0 Hz, 2H, H^7 + H^{11}), 3.84 (s, 2H, H^4), 3.54 (t, *J* = 7.0 Hz, 2H, H^{13}), 3.47 (d, *J* = 7.0 Hz, 2H, H^{16}), 2.05 – 1.99 (m, 2H, H^{14}), 1.93 – 1.88 (m, 2H, H^{15}); **^{13}C NMR** (126 MHz, CD_3OD) δ 169.5 (C^3), 149.4 (C^9), 141.2 (C^6), 115.5 (C^8 + C^{10}), 114.8 (C^7 + C^{11}), 47.2 (C^4), 45.7 (C^{13}), 45.4 (C^{16}), 25.6 (C^{14}), 23.7 (C^{15}); **HRMS** (ESI): calculated for $C_{12}H_{17}N_2O_2$ $[M+H]^+$ requires *m/z* 221.1285, found *m/z* 221.1287.

2-((4-Hydroxyphenyl)amino)-1-(piperidin-1-yl)ethan-1-one (1j):

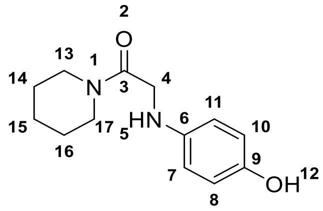

General procedure A: Purification by column chromatography (hexane/EtOAc, 50:50) afforded the title compound (170 mg, 52%) as a yellow solid. **m.p.** = 128 – 130 °C (hexane/EtOAc); **IR (thin film)** ν_{max}/cm^{-1}: 3350 (br), 2939 (s), 1633 (s), 1517 (s), 1444 (s), 1251 (s); **^{1}H NMR** (500 MHz, DMSO-*d*^{6}) δ 8.42 (s, 1H, H^{12}), 6.55 (d, *J* = 9.0 Hz, 2H, H^8 + H^{10}), 6.51 (d, *J* = 9.0 Hz, 2H, H^7 + H^{11}), 4.90 (t, *J* = 5.0 Hz, 1H, H^5), 3.77 (d, *J* = 5.0 Hz, 2H, H^4), 3.41 – 3.41 (m, 4H, H^{13} + H^{17}), 1.62 – 1.57 (s, 2H, H^{15}), 1.54 – 1.49 (m, 2H, H^{14}), 1.46 – 1.41 (m, 2H, H^{16}); **^{13}C NMR** (126 MHz, DMSO-*d*^{6}) δ 168.1 (C^3), 149.0 (C^9), 141.6 (C^6), 116.0 (C^8 + C^{10}), 114.2 (C^7 + C^{11}), 46.2 (C^4), 45.3 (C^{13}), 42.8 (C^{17}), 26.4 (C^{14}), 25.8 (C^{16}), 24.5 (C^{15}); **HRMS** (ESl): calculated for $C_{13}H_{19}N_2O_2$ $[M+H]^+$ requires *m/z* 235.1441, found *m/z* 235.1444.

2-((4-Hydroxyphenyl)amino)-1-phenylethan-1-one (1m):

Ketone **1m** was prepared by the reported procedure.[5]

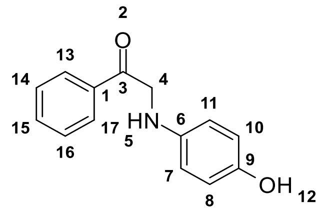

An oven-dried round bottom flask was charged with 4-aminophenol (109 mg, 1.00 mmol, 100 mol%), $NaHCO_3$ (84.0 mg, 1.00 mmol, 100 mol%) and EtOH (5 mL) at r.t. 2-Bromo-1-phenylethan-1-one (198 mg, 1.00 mmol, 100 mol%) in EtOH (2 mL) was then added dropwise to the stirring solution over 1 minute at r.t. After the addition was complete, the reaction mixture was stirred for 12 hours at r.t. and the progress of the reaction was monitored by TLC. Upon completion, the mixture was filtered and the filtrate was concentrated *in vacuo*, which was purified by column chromatography (hexane/EtOAc, 75:25) affording the title compound (95.4 mg, 42%) as a yellow solid. **m.p.** = 126 – 128 °C (hexane/EtOAc); **IR (thin film)** ν_{max}/cm^{-1}: 3382 (br), 1687 (s), 1516 (s), 1449 (s), 1222 (s); **^{1}H NMR** (500 MHz, $CDCl_3$) δ 8.03 (d, *J* = 7.0 Hz, 2H, H^{13} + H^{17}), 7.66 – 7.63 (m, 1H, H^{15}), 7.55 – 7.52 (m, 2H, H^{14} + H^{16}), 6.77 (d, *J* = 9.0 Hz, 2H, H^8 + H^{10}), 6.66 (d, *J* = 9.0 Hz, 2H, H^7 + H^{11}), 4.65 (br., 1H, H^5), 4.60 (s, 2H, H^4); **^{13}C NMR** (126 MHz, $CDCl_3$) δ 195.6 (C^3), 148.0 (C^9), 141.5 (C^6), 135.0 (C^1), 133.9

(C^{15}), 128.9 (C^{14} + C^{16}), 127.8 (C^{13} + C^{17}), 116.4 (C^{8} + C^{10}), 114.5 (C^{7} + C^{11}), 51.4 (C^{4}); **HRMS** (ESI): calculated for $C_{14}H_{14}NO_2$ $[M+H]^+$ requires *m/z* 228.1019, found *m/z* 228.1017.

Ethyl (4-hydroxyphenyl)glycinate (1n):

Ester **1n** was synthesized according to a modified procedure.[4]

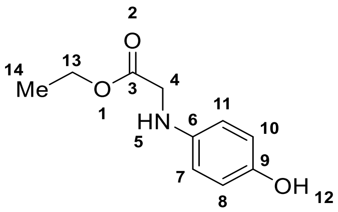

An oven-dried round bottom flask was charged with 4-aminophenol (109 mg, 1.00 mmol, 100 mol%), TEA (0.21 mL, 1.50 mmol, 150 mol%) and $CHCl_3$ (2 mL). Ethyl 2-bromoacetate (1.20 mmol, 120 mol%) in $CHCl_3$ (2 mL) was then added dropwise over 2 minutes at r.t. After the addition was complete, the reaction mixture was stirred for 48 hours at reflux and the progress of the reaction was monitored by TLC. Upon completion, the reaction mixture was concentrated *in vacuo*, which was purified by column chromatography (hexane/EtOAc, 50:50) affording the title compound (64.4 mg, 33%) as a pale-yellow solid. **m.p.** = 70 – 72 °C (hexane/EtOAc); **IR (thin film)** ν_{max}/cm^{-1}: 3384 (br), 1730 (s), 1516 (s), 1373 (s), 1204 (s); **^{1}H NMR** (500 MHz, $CDCl_3$) δ 6.72 (d, J = 7.5 Hz, 2H, H^8 + H^{10}), 6.55 (d, J = 7.5 Hz, 2H, H^7 + H^{11}), 4.25 (q, J = 7.0 Hz, 2H, H^{13}), 3.87 (s, 2H, H^4), 1.31 (t, J = 7.0 Hz, 3H, H^{14}); **^{13}C NMR** (126 MHz, $CDCl_3$) δ 171.6 (C^3), 148.4 (C^9), 141.2 (C^6), 116.3 (C^8 + C^{10}), 114.7 (C^7 + C^{11}), 61.3 (C^{13}), 47.0 (C^4), 14.2 (C^{14}); **HRMS** (ESI): calculated for $C_{10}H_{14}NO_3$ $[M+H]^+$ requires *m/z* 196.0968, found *m/z* 196.0967.

Alkenes:

Styrene **2a**, and simple substituted styrenes **2b-g**, **2j-k**, **2m, 2t** and **2w** were purchased and used as received.

2h has previously been prepared in our lab.[6]

Alkenes 4,4,5,5-tetramethyl-2-(4-(prop-1-en-2-yl)phenyl)-1,3,2-dioxaborolane **2i**, 1-fluoro-2-(prop-1-en-2-yl)benzene **2l**, 5-(prop-1-en-2-yl)benzofuran **2n**, 3-(prop-1-en-2-yl)-1-tosyl-1*H*-indole **2o**, 2-(prop-1-en-2-yl)benzofuran **2p**, 3-(prop-1-en-2-yl)thiophene **2q**, (8*R*,9*S*,13*S*,14*S*)-13-methyl-3-(prop-1-en-2-yl)-6,7,8,9,11,12,13,14,15,16-decahydro-17*H*-cyclopenta[*a*]phenanthren-17-one **2r**, (1-cyclobutylvinyl)benzene **2s**, 7-methylenebicyclo[4.2.0]octa-1,3,5-triene **2u**, but-3-ene-1,3-diyldibenzene **2v**, oct-1-en-2-ylbenzene **2x** were prepared by reported procedures.[7-17]

4,4,5,5-Tetramethyl-2-(4-(prop-1-en-2-yl)phenyl)-1,3,2-dioxaborolane (2i):

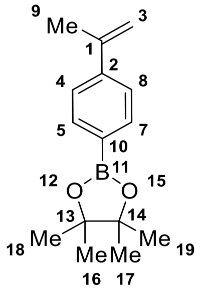

^{1}H NMR (500 MHz, $CDCl_3$) δ 7.81 (d, *J* = 8.0 Hz, 2H, $H^5 + H^7$), 7.50 (d, *J* = 8.0 Hz, 2H, H^4 + H^8), 5.45 (s, 1H, H^3), 5.15 (s, 1H, H^3), 2.19 (s, 3H, H^9), 1.38 (s, 12H, $H^{16\text{-}19}$).

The spectroscopic properties were consistent with the data available in the literature.[7]

1-Fluoro-2-(prop-1-en-2-yl)benzene (2l):

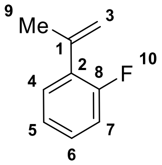

^{1}H NMR (500 MHz, $CDCl_3$) δ 7.35 – 7.32 (m, 1H, ArH), 7.28 – 7.24 (m, 1H, ArH), 7.14 – 7.11 (m, 1H, ArH), 7.09 – 7.05 (m, 1H, ArH), 5.27 (s, 2H, H^3), 2.19 (s, 3H, H^9).

The spectroscopic properties were consistent with the data available in the literature.[8]

5-(Prop-1-en-2-yl)benzofuran (2n):

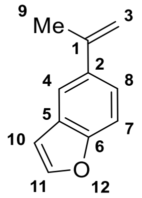

^{1}H NMR (500 MHz, $CDCl_3$) δ 7.79 (s, 1H, ArH), 7.70 (d, *J* = 2.0 Hz, 1H, H^{11}), 7.57 (s, 2H, ArH), 6.84 (d, *J* = 2.0 Hz, 1H, H^{10}), 5.50 (s, 1H, H^3), 5.22 (s, 1H, H^3), 2.34 (s, 3H, H^9).

The spectroscopic properties were consistent with the data available in the literature.[9]

3-(Prop-1-en-2-yl)-1-tosyl-1*H*-indole (2o):

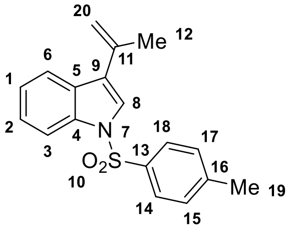

^{1}H NMR (500 MHz, $CDCl_3$) δ 8.04 (d, *J* = 8.5 Hz, 1H, ArH), 7.84 (d, *J* = 8.0 Hz, 1H, ArH), 7.80 (d, *J* = 8.5 Hz, 2H, H^{14} + H^{18}), 7.61 (s, 1H, H^8), 7.37 – 7.34 (m, 1H, ArH), 7.31 – 7.28 (m, 1H, ArH), 7.23 (d, *J* = 8.5 Hz, 2H, H^{15} + H^{17}), 5.55 (s, 1H, H^{20}), 5.26 (s, 1H, H^{20}), 2.35 (s, 3H, H^{19}), 2.20 (s, 3H, H^{12}).

The spectroscopic properties were consistent with the data available in the literature.[10]

2-(Prop-1-en-2-yl)benzofuran (2p):

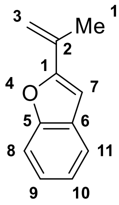

^{1}H NMR (500 MHz, $CDCl_3$) δ 7.58 (d, *J* = 7.5 Hz, 1H, ArH), 7.50 (d, *J* = 8.0 Hz, 1H, ArH), 7.33 – 7.30 (m, 1H, ArH), 7.26 – 7.23 (m, 1H, ArH), 6.68 (s, 1H, H^7), 5.86 (s, 1H, H^3), 5.23 (s, 1H, H^3), 2.18 (s, 3H, H^{12}).

The spectroscopic properties were consistent with the data available in the literature.[11]

3-(Prop-1-en-2-yl)thiophene (2q):

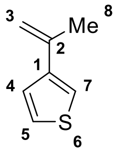

^{1}H NMR (500 MHz, $CDCl_3$) δ 7.41 – 7.40 (m, 1H, ArH), 7.37 – 7.35 (m, 1H, ArH), 7.30 – 7.29 (m, 1H, ArH), 5.50 (s, 1H, H^3), 5.16 (s, 1H, H^3), 2.24 (s, 3H, H^8).

The spectroscopic properties were consistent with the data available in the literature.[12]

(*8R,9S,13S,14S*)-13-Methyl-3-(prop-1-en-2-yl)-6,7,8,9,11,12,13,14,15,16-decahydro-17*H*-cyclopenta[*a*]phenanthren-17-one (2r):

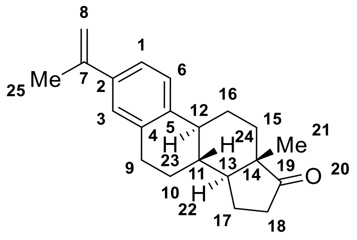

^{1}H NMR (500 MHz, $CDCl_3$) δ 7.32 – 7.28 (m, 2H, H^1 + H^6), 7.23 (s, 1H, H^3), 5.36 (s, 1H, H^8), 5.07 (s, 1H, H^8), 2.98 – 2.95 (m, 2H, H^{9-18}), 2.56 – 2.45 (m, 2H, H^{9-18}), 2.36 – 2.31 (m, 1H, H^{9-18}), 2.21 – 1.98 (m, 7H, H^{9-18} + H^{25}), 1.70 – 1.44 (m, 6H, H^{9-18}), 0.94 (s, 3H, H^{21}).

The spectroscopic properties were consistent with the data available in the literature.[13]

(1-Cyclobutylvinyl)benzene (2s):

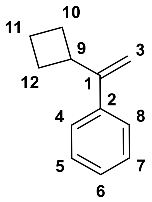

^{1}H NMR (500 MHz, $CDCl_3$) δ 7.45 – 7.43 (m, 2H, H^4 + H^8), 7.39 – 7.36 (m, 2H, H^5 + H^7), 7.33 – 7.30 (m, 1H, H^6), 5.41 (s, 1H, H^3), 5.11 (s, 1H, H^3), 3.57 – 3.50 (m, 1H, H^9), 2.33 – 2.24 (m, 2H, H^{10-12}), 2.11 – 1.98 (m, 3H, H^{10-12}), 1.88 – 1.79 (m, 1H, H^{10-12}).

The spectroscopic properties were consistent with the data available in the literature.[14]

7-Methylenebicyclo[4.2.0]octa-1,3,5-triene (2u):

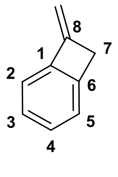

^{1}H NMR (500 MHz, $CDCl_3$) δ 7.29 – 7.19 (m, 4H, H^{2-5}), 5.35 (s, 1H, H^9), 4.98 (s, 1H, H^9), 3.67 (s, 2H, H^7).

The spectroscopic properties were consistent with the data available in the literature.[15]

But-3-ene-1,3-diyldibenzene (2v):

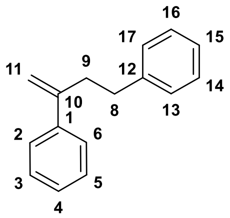

^{1}H NMR (500 MHz, $CDCl_3$) δ 7.47 (d, *J* = 7.0 Hz, 2H, H^{13} + H^{17}), 7.40 – 7.37 (m, 2H, ArH), 7.33 – 7.30 (m, 3H, ArH), 7.24 – 7.21 (m, 3H, ArH), 5.33 (s, 1H, H^{11}), 5.10 (s, 1H, H^{11}), 2.87 – 2.79 (m, 4H, H^8 + H^9).

The spectroscopic properties were consistent with the data available in the literature.[16]

Oct-1-en-2-ylbenzene (2x):

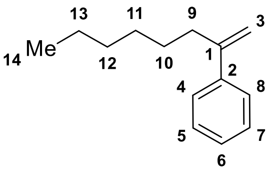

^{1}H NMR (500 MHz, $CDCl_3$) δ 7.53 (d, *J* = 8.0 Hz, 2H, H^4 + H^8), 7.44 – 7.41 (m, 2H, H^5 + H^7), 7.38 – 7.35 (m, 1H, H^6), 5.38 (s, 1H, H^3), 5.17 (s, 1H, H^3), 2.62 (t, *J* = 7.5 Hz, 2H, H^9), 1.61 – 1.55 (m, 2H, H^{10-13}), 1.48 – 1.36 (m, 6H, H^{10-13}), 1.00 (t, *J* = 6.5 Hz, 3H, H^{14}).

The spectroscopic properties were consistent with the data available in the literature.[17]

Reaction scope

General procedure B for the asymmetric α-alkylation of glycine derivatives:

A Schlenk tube was charged with substrate (0.10 mmol, 100 mol%), $[Ir(cod)_2]BARF$ (9.54 mg, 7.50 μmol, 7.5 mol%), (*R*)-DM-SEGPHOS (5.42 mg, 7.50 μmol, 7.5 mol%) and styrene derivative (if non-volatile, 400 mol%). The Schlenk tube was evacuated and refilled with N_2 (three cycles), then the alkene partner (if volatile, 400-1000 mol%) was added followed by anhydrous *t*-BuOH (0.1 mL, 1.0 M). The tube was sealed and heated at 110 °C for 96 hours. After cooling to r.t., the solvent was removed under reduced pressure and the crude reaction mixture was purified by FCC. The racemic products were also obtained using the above procedure (*rac*-BINAP was used in place of (*R*)-DM-SEGPHOS) and purified by FCC.

(*S*)-2-((4-Hydroxyphenyl)amino)-*N*,*N*,3-trimethyl-3-phenylbutanamide (3aa):

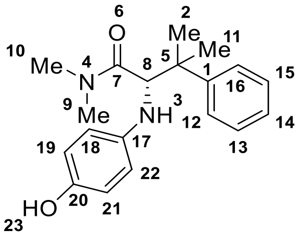

General procedure B: The reaction was carried out with substrate **1a** (19.4 mg, 0.10 mmol, 100 mol%) and prop-1-en-2-ylbenzene **2a** (52.0 µL, 0.40 mmol, 400 mol%). Purification of the residue by FCC (hexane/EtOAc 50:50) afforded the title compound (23.1 mg, 74%, >30:1 B:L, e.r. = 97:3) as a pale-yellow oil. ^{1}H NMR analysis of the crude material gave >30:1 B:L. $[\alpha]_D^{25}$ = -37.4 (c = 1.0, $CHCl_3$); **IR (thin film)** ν_{max}/cm^{-1}: 3378 (br), 2930 (s), 2862 (s), 1610 (s), 1515 (s), 1445 (s), 699 (s); **^{1}H NMR** (500 MHz, $CDCl_3$) δ 7.48 (d, *J* = 8.0 Hz, 2H, H^{12} + H^{16}), 7.35 – 7.32 (m, 2H, H^{13} + H^{15}), 7.26 – 7.24 (m, 1H, H^{14}), 6.67 – 6.55 (m, 5H, H^{18} + H^{19} + H^{21} + H^{22} + H^{23}), 4.95 – 3.78 (m, 2H, H^3 + H^8), 2.68 (s, 3H, H^9), 2.26 (s, 3H, H^{10}), 1.63 (s, 3H, H^2), 1.53 (s, 3H, H^{11}); **^{13}C NMR** (126 MHz, $CDCl_3$) δ 172.9 (C^7), 149.5 (C^{17}), 146.2 (C^1), 141.3 (C^{20}), 128.0 (C^{13} + C^{15}), 126.9 (C^{12} + C^{16}), 126.6 (C^{14}), 116.7 (ArC), 116.2 (ArC), 64.0 (C^8), 42.3 (C^5), 36.9 (C^9), 35.5 (C^{10}), 26.5 (C^2), 22.9 (C^{11}); **HRMS** (ESl): calculated for $C_{19}H_{25}N_2O_2$ $[M+H]^+$ requires *m/z* 313.1911, found *m/z* 313.1914; **Chiral SFC:** YMC Chiral ART Cellulose-SC column (25 cm), CO_2:*i*-PrOH 75:25, 3 mL/min, 228 bar, r.t. Retention times: 3.0 mins (major), 4.1 mins (minor), e.r. = 97:3.

SFC analysis of the racemate, prepared using rac-BINAP:

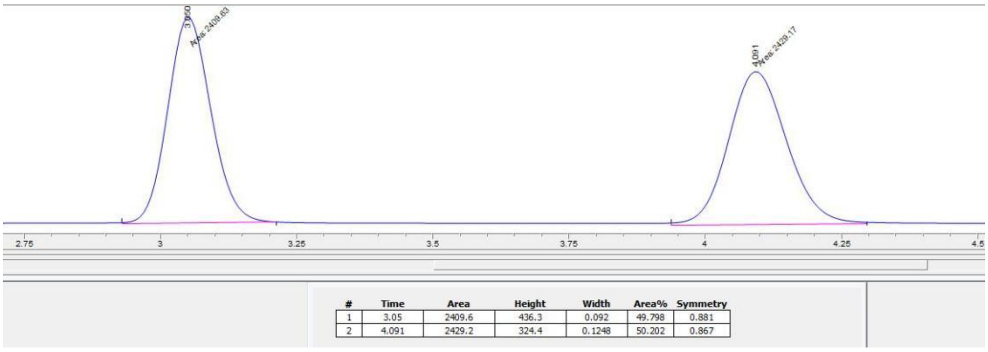

#	Time	Area	Height	Width	Area%	Symmetry
1	3.05	2409.6	436.3	0.092	49.798	0.881
2	4.091	2429.2	324.4	0.1248	50.202	0.867

SFC analysis of the enantioenriched material, prepared using (R)-DM-SEGPHOS:

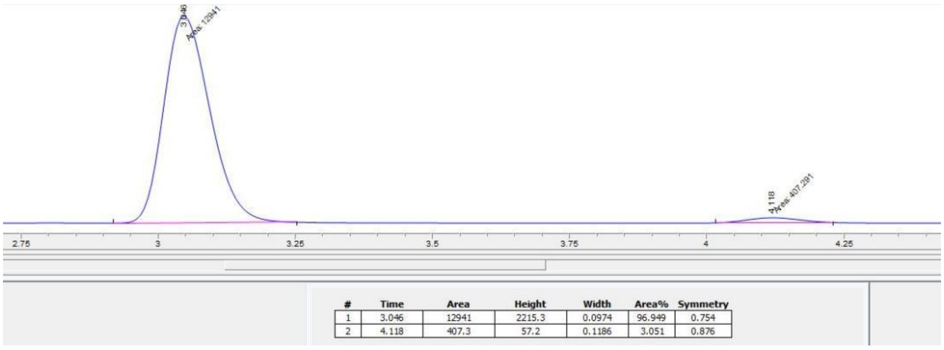

#	Time	Area	Height	Width	Area%	Symmetry
1	3.046	12941	2215.3	0.0974	96.949	0.754
2	4.118	407.3	57.2	0.1186	3.051	0.876

(*S*)-2-((4-Methoxyphenyl)amino)-*N*,*N*,3-trimethyl-3-phenylbutanamide (3ba):

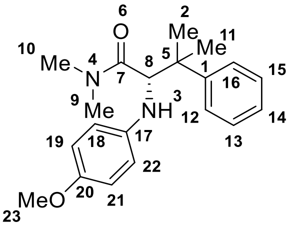

General procedure B: The reaction was carried out with substrate **1b** (20.8 mg, 0.10 mmol, 100 mol%) and prop-1-en-2-ylbenzene **2a** (52.0 µL, 0.40 mmol, 400 mol%). Purification of the residue by FCC (hexane/EtOAc 65:35) afforded the title compound (20.2 mg, 62%, >30:1 B:L, e.r. = 92:8) as a pale-yellow solid. ^{1}H NMR analysis of the crude material gave >30:1 B:L. **m.p.** = 111 – 113 °C (hexane/EtOAc); $[\boldsymbol{\alpha}]_{\boldsymbol{D}}^{\mathbf{25}}$ = -13.6 (c = 1.0, $CHCl_3$); **IR (thin film)** ν_{max}/cm^{-1}: 2934 (s), 1623 (s), 1511 (s), 1478 (s), 1243 (s), 829 (s), 700 (s); **^{1}H NMR** (500 MHz, $CDCl_3$) δ 7.49 (d, *J* = 7.0 Hz, 2H, H^{12} + H^{16}), 7.35 – 7.32 (m, 2H, H^{13} + H^{15}), 7.27 – 7.24 (m, 1H, H^{14}), 6.75 (d, *J* = 9.0 Hz, 2H, H^{19} + H^{21}), 6.63 (d, *J* = 9.0 Hz, 2H, H^{18} + H^{22}), 4.22 (s, 1H, H^8), 3.75 (s, 3H, H^{23}), 2.69 (s, 3H, H^9), 2.27 (s, 3H, H^{10}), 1.62 (s, 3H, H^2), 1.53 (s, 3H, H^{11}); **^{13}C NMR** (126 MHz, $CDCl_3$) δ 172.5 (C^7), 152.9 (C^{20}), 146.3 (C^{17}), 142.2 (C^1), 128.0 (C^{13} + C^{15}), 126.9 (C^{12} + C^{16}), 126.5 (C^{14}), 116.2 (C^{19} + C^{21}), 114.8 (C^{18} + C^{22}), 63.6 (C^8), 55.7 (C^{23}), 42.5 (C^5), 36.8 (C^9), 35.2 (C^{10}), 26.3 (C^2), 22.9 (C^{11}); **HRMS** (ESl): calculated for $C_{20}H_{27}N_2O_2$ $[M+H]^+$ requires *m/z* 327.2067, found *m/z* 327.2071; **Chiral SFC:** YMC Chiral ART Cellulose-SB column (25 cm), CO_2:*i*-PrOH 75:25, 3 mL/min, 223 bar, r.t. Retention times: 2.0 mins (major), 2.2 mins (minor), e.r. = 92:8.

SFC analysis of the racemate, prepared using rac-BINAP:

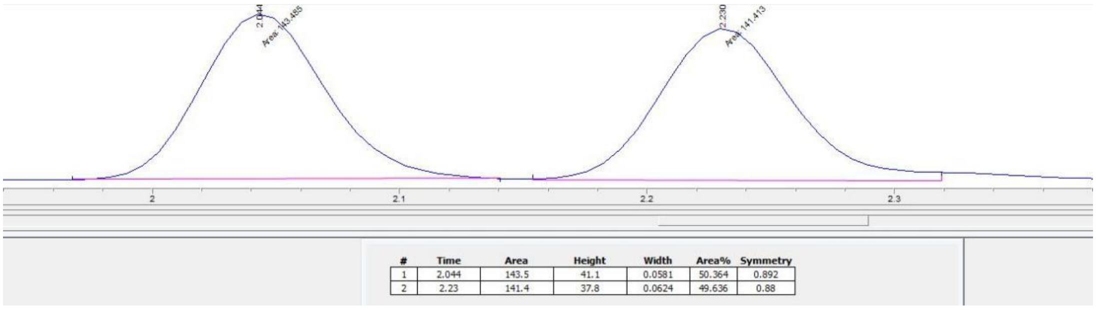

SFC analysis of the enantioenriched material, prepared using (R)-DM-SEGPHOS:

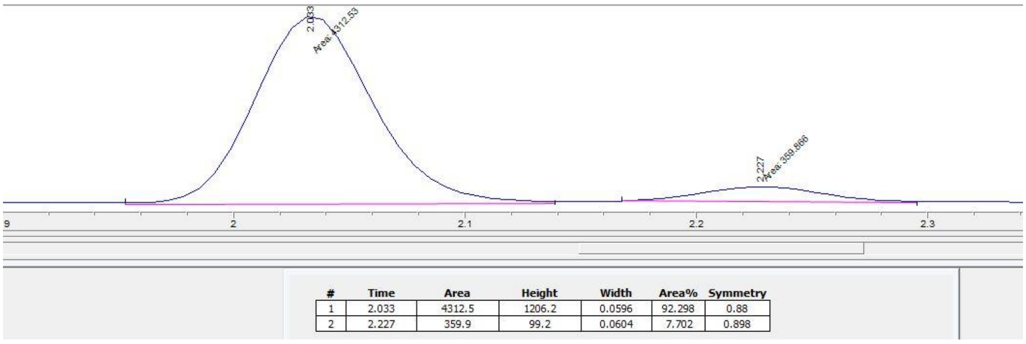

(*S*)-*N,N*,3-Trimethyl-3-phenyl-2-(*p*-tolylamino)butanamide (3ca):

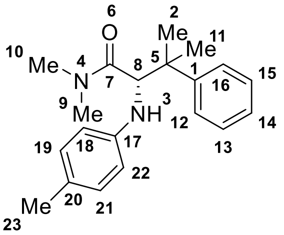

General procedure B: The reaction was carried out with substrate **1c** (19.2 mg, 0.10 mmol, 100 mol%) and prop-1-en-2-ylbenzene **2a** (52.0 µL, 0.40 mmol, 400 mol%). Purification of the residue by FCC (hexane/EtOAc 65:35) afforded the title compound (21.7 mg, 70%, >30:1 B:L, e.r. = 95:5) as a colorless solid. ^{1}H NMR analysis of the crude material gave >30:1 B:L. **m.p.** = 131 – 133 °C (hexane/EtOAc); $[\boldsymbol{\alpha}]_{\boldsymbol{D}}^{\mathbf{25}}$ = -19.0 (c = 1.0, $CHCl_3$); **IR (thin film)** ν_{max}/cm^{-1}: 3352 (s), 2922 (s), 1629 (s), 1512 (s), 1478 (s), 1243 (s), 810 (s), 698 (s); **^{1}H NMR** (500 MHz, $CDCl_3$) δ 7.50 (d, *J* = 7.5 Hz, 2H, H^{12} + H^{16}), 7.36 – 7.33 (m, 2H, H^{13} + H^{15}), 7.27 – 7.24 (m, 1H, H^{14}), 6.97 (d, *J* = 8.0 Hz, 2H, H^{19} + H^{21}), 6.59 (d, *J* = 8.0 Hz, 2H, H^{18} + H^{22}), 4.60 (br. s, 1H, H^3), 4.32 (s, 1H, H^8), 2.70 (s, 3H, H^9), 2.27 (s, 3H, H^{10}), 2.24 (s, 3H, H^{23}), 1.61 (s, 3H, H^2), 1.53 (s, 3H, H^{11}); **^{13}C NMR** (126 MHz, $CDCl_3$) δ 172.3 (C^7), 146.3 (C^{17}), 145.8 (C^1), 129.8 (C^{19} + C^{21}), 128.0 (C^{13} + C^{15}), 127.5 (C^{20}), 126.9 (C^{12} + C^{16}), 126.5 (C^{14}), 114.4 (C^{18} + C^{22}), 62.1 (C^8), 42.7 (C^5), 36.8 (C^9), 35.2 (C^{10}), 26.4 (C^2), 22.8 (C^{11}), 20.4 (C^{23}); **HRMS** (ESl): calculated for $C_{20}H_{27}N_2O$ $[M+H]^+$ requires *m/z* 311.2118, found *m/z* 311.2120; **Chiral SFC:**

YMC Chiral ART Cellulose-SC column (25 cm), CO_2:*i*-PrOH 75:25, 3 mL/min, 228 bar, r.t. Retention times: 2.7 mins (major), 4.3 mins (minor), e.r. = 95:5.

SFC analysis of the racemate, prepared using rac-BINAP:

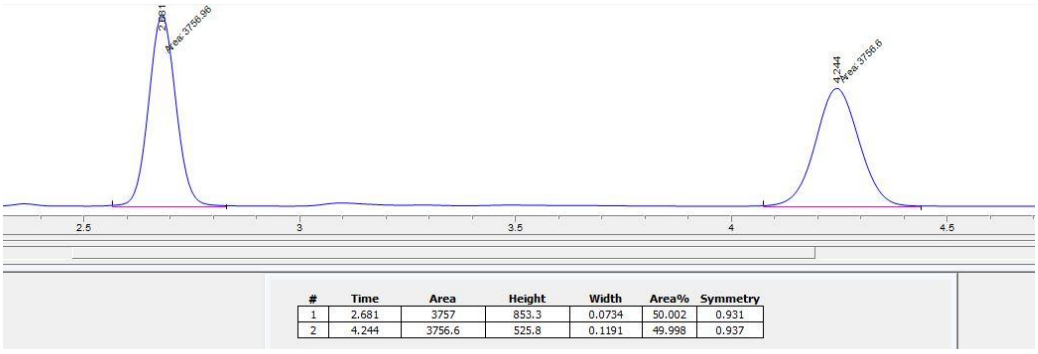

#	Time	Area	Height	Width	Area%	Symmetry
1	2.681	3757	853.3	0.0734	50.002	0.931
2	4.244	3756.6	525.8	0.1191	49.998	0.937

SFC analysis of the enantioenriched material, prepared using (R)-DM-SEGPHS:

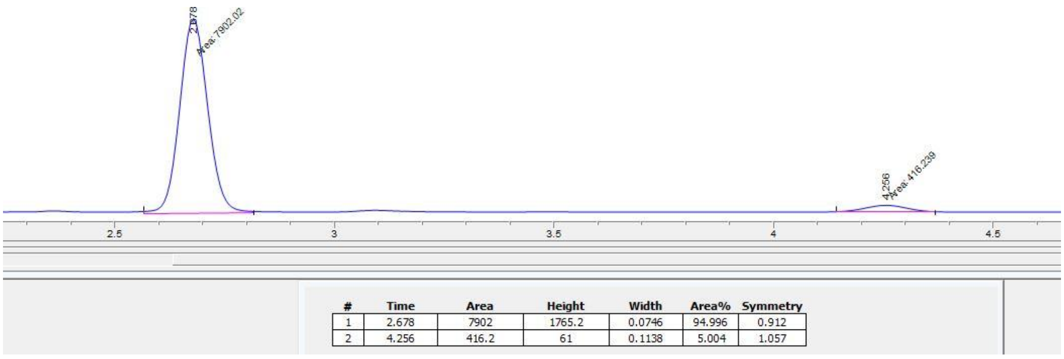

#	Time	Area	Height	Width	Area%	Symmetry
1	2.678	7902	1765.2	0.0746	94.996	0.912
2	4.256	416.2	61	0.1138	5.004	1.057

(*S*)-*N*,*N*,3-Trimethyl-3-phenyl-2-(phenylamino)butanamide (3da):

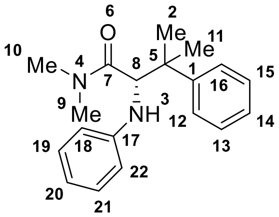

General procedure B: The reaction was carried out with substrate **1d** (17.8 mg, 0.10 mmol, 100 mol%) and prop-1-en-2-ylbenzene **2a** (52.0 µL, 0.40 mmol, 400 mol%). Purification of the residue by FCC (hexane/EtOAc 65:35) afforded the title compound (20.7 mg, 70%, >30:1 B:L, e.r. = 96:4) as a colorless solid. ^{1}H NMR analysis of the crude material gave >30:1 B:L. **m.p.** = 122 – 124 °C (hexane/EtOAc); $[\alpha]_D^{25}$ = -14.2 (c = 1.0, $CHCl_3$); **IR (thin film)** ν_{max}/cm^{-1}: 2936 (s), 1625 (s), 1599 (s), 1510 (s), 1394 (s), 741 (s), 694 (s); **^{1}H NMR** (500 MHz, $CDCl_3$) δ 7.47 (d, *J* = 7.5 Hz, 2H, H^{12} + H^{16}), 7.34 – 7.30 (m, 2H, H^{13} + H^{15}), 7.25 – 7.22 (m, 1H, H^{14}), 7.16 – 7.12 (m, 2H, H^{19} + H^{21}), 6.73 – 6.70 (m, 1H, H^{20}), 6.65 (d, *J* = 7.5 Hz, 2H, H^{18} + H^{22}), 4.81 (br. s, 1H, H^3), 4.35 (s, 1H, H^8), 2.68 (s, 3H, H^9), 2.26 (s, 3H, H^{10}), 1.59 (s, 3H, H^2), 1.52

(s, 3H, H[11]); **^{13}C NMR** (126 MHz, $CDCl_3$) δ 172.0 (C[7]), 147.9 (C[17]), 146.1 (C[1]), 129.3 (C[19] + C[21]), 128.0 (C[13] + C[15]), 126.9 (C[12] + C[16]), 126.6 (C[14]), 118.3 (C[18] + C[22]), 114.2 (C[20]), 61.6 (C[8]), 42.7 (C[5]), 36.8 (C[9]), 35.2 (C[10]), 26.3 (C[2]), 22.8 (C[11]); **HRMS** (ESI): calculated for $C_{19}H_{25}N_2O$ $[M+H]^+$ requires *m/z* 297.1961, found *m/z* 297.1968; **Chiral SFC:** YMC Chiral ART Cellulose-SC column (25 cm), CO_2:*i*-PrOH 75:25, 3 mL/min, 228 bar, r.t. Retention times: 2.4 mins (major), 3.5 mins (minor), e.r. = 96:4.

SFC analysis of the racemate, prepared using rac-BINAP:

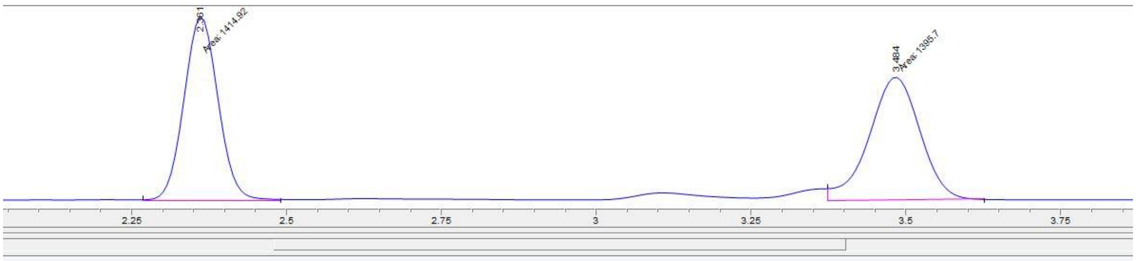

#	Time	Area	Height	Width	Area%	Symmetry
1	2.361	1414.9	364.1	0.0648	50.342	0.935
2	3.484	1395.7	242.8	0.0958	49.658	1.052

SFC analysis of the enantioenriched material, prepared using (R)-DM-SEGPHOS:

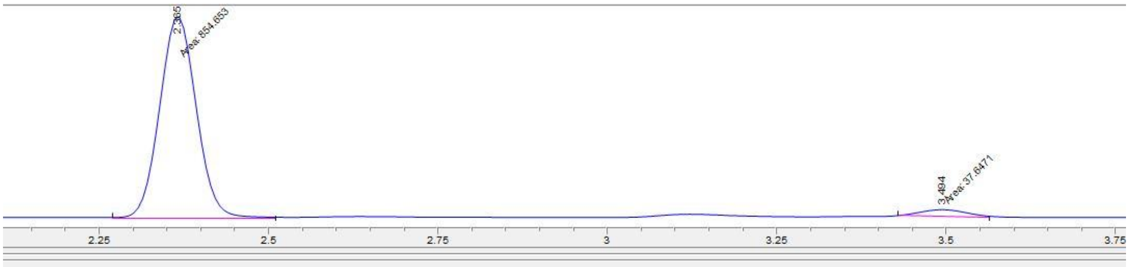

#	Time	Area	Height	Width	Area%	Symmetry
1	2.365	854.7	224.9	0.0633	95.781	0.933
2	3.494	37.6	7.7	0.0811	4.219	0.794

(*S*)-2-((4-Fluorophenyl)amino)-*N*,*N*,3-trimethyl-3-phenylbutanamide (3ea):

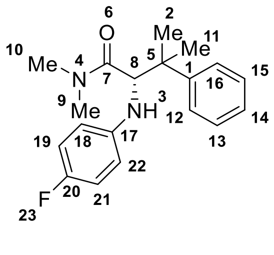

General procedure B: The reaction was carried out with substrate **1e** (19.6 mg, 0.10 mmol, 100 mol%) and prop-1-en-2-ylbenzene **2a** (52.0 µL, 0.40 mmol, 400 mol%). Purification of the residue by FCC (hexane/EtOAc 65:35) afforded the title compound (24.8 mg, 79%, >30:1 B:L, e.r. = 96:4) as a colorless solid. ^{1}H NMR analysis of the crude material gave >30:1 B:L.

m.p. = 119 – 121 °C (hexane/EtOAc); $[\alpha]_D^{25}$ = -7.1 (c = 1.0, $CHCl_3$); **IR (thin film)** ν_{max}/cm^{-1}: 2925 (s), 1624 (s), 1509 (s), 1387 (s), 1216 (s), 827 (s), 774 (s), 699 (s); **^{1}H NMR** (500 MHz, $CDCl_3$) δ 7.48 (d, *J* = 7.0 Hz, 2H, H^{12} + H^{16}), 7.36 – 7.33 (m, 2H, H^{13} + H^{15}), 7.27 – 7.24 (m, 1H, H^{14}), 6.87 – 6.84 (m, 2H, H^{19} + H^{21}), 6.60– 6.56 (m, 2H, H^{18} + H^{22}), 4.50 (br. s, 1H, H^3), 4.24 (s, 1H, H^8), 2.71 (s, 3H, H^9), 2.29 (s, 3H, H^{10}), 1.61 (s, 3H, H^2), 1.52 (s, 3H, H^{11}); **^{13}C NMR** (126 MHz, $CDCl_3$) δ 172.2 (C^7), 156.3 (d, *J* = 235.0 Hz, C^{20}), 146.1 (C^1), 144.5 (d, *J* = 2.5 Hz, C^{17}), 128.1 (C^{13} + C^{15}), 126.9 (C^{12} + C^{16}), 126.6 (C^{14}), 115.7 (d, *J* = 22.5 Hz, C^{19} + C^{21}), 115.5 (d, *J* = 7.5 Hz, C^{18} + C^{22}), 63.0 (C^8), 42.6 (C^5), 36.8 (C^9), 35.3 (C^{10}), 26.2 (C^2), 22.9 (C^{11}); ^{19}F NMR (471 MHz, $CDCl_3$) δ -126.7; **HRMS** (ESI): calculated for $C_{19}H_{24}FN_2O$ $[M+H]^+$ requires *m/z* 315.1867, found *m/z* 315.1871; **Chiral SFC:** YMC Chiral ART Cellulose-SC column (25 cm), CO_2:*i*-PrOH 75:25, 3 mL/min, 228 bar, r.t. Retention times: 1.9 mins (major), 2.9 mins (minor), e.r. = 96:4.

SFC analysis of the racemate, prepared using rac-BINAP:

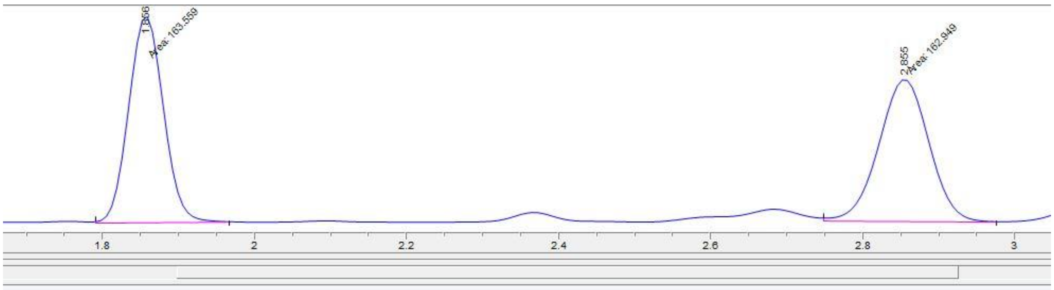

#	Time	Area	Height	Width	Area%	Symmetry
1	1.856	163.6	51.1	0.0533	50.094	0.937
2	2.855	162.9	35.1	0.0773	49.906	1.013

SFC analysis of the enantioenriched material, prepared using (R)-DM-SEGPHOS:

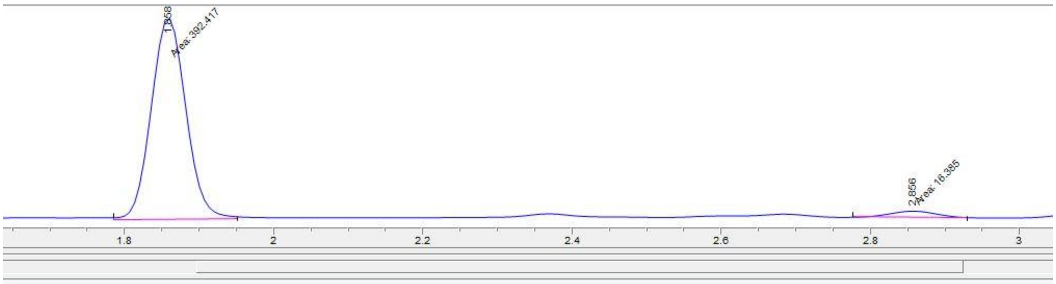

#	Time	Area	Height	Width	Area%	Symmetry
1	1.858	392.4	121.7	0.0538	95.992	0.937
2	2.856	16.4	3.8	0.0718	4.008	0.886

(*S*)-2-((4-Fluoro-3-methoxyphenyl)amino)-*N*,*N*,3-trimethyl-3-phenylbutanamide (3fa):

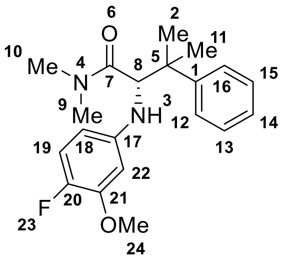

General procedure B: The reaction was carried out with substrate **1f** (22.6 mg, 0.10 mmol, 100 mol%) and prop-1-en-2-ylbenzene **2a** (52.0 µL, 0.40 mmol, 400 mol%). Purification of the residue by FCC (hexane/EtOAc 65:35) afforded the title compound (24.8 mg, 72%, >30:1 B:L, e.r. = 93.5:6.5) as a colorless oil. ^{1}H NMR analysis of the crude material gave >30:1 B:L. $[\boldsymbol{\alpha}]_{\boldsymbol{D}}^{\mathbf{25}}$ = +15.1 (c = 1.0, $CHCl_3$); **IR (thin film)** ν_{max}/cm^{-1}: 3386 (s), 2923 (s), 1631 (s), 1610 (s), 1515 (s), 1218 (s), 1121 (s), 775 (s), 693 (s); **^{1}H NMR** (500 MHz, $CDCl_3$) δ 7.48 (d, *J* = 7.5 Hz, 2H, $H^{12} + H^{16}$), 7.36 – 7.33 (m, 2H, $H^{13} + H^{15}$), 7.28 – 7.25 (m, 1H, H^{14}), 6.85 (dd, *J* = 11.0, 8.5 Hz, 1H, H^{19}), 6.23 (dd, *J* = 7.5, 2.5 Hz, 1H, H^{18}), 6.11 – 6.08 (m, 1H, H^{22}), 4.24 (s, 1H, H^{8}), 3.82 (s, 3H, H^{24}), 2.74 (s, 3H, H^{9}), 2.33 (s, 3H, H^{10}), 1.60 (s, 3H, H^{2}), 1.52 (s, 3H, H^{11}); **^{13}C NMR** (126 MHz, $CDCl_3$) δ 172.2 (C^{7}), 147.9 (d, *J* = 11.3 Hz, C^{21}), 146.16 (C^{1}), 146.15 (d, *J* = 233.8 Hz, C^{20}), 145.0 (d, *J* = 2.5 Hz, C^{17}), 128.1 ($C^{13} + C^{15}$), 126.9 ($C^{12} + C^{16}$), 126.6 (C^{14}), 116.1 (d, *J* = 18.8 Hz, C^{19}), 104.9 (d, *J* = 6.3 Hz, C^{22}), 101.4 (C^{18}), 62.8 (C^{8}), 56.2 (C^{24}), 42.5 (C^{5}), 36.9 (C^{9}), 35.3 (C^{10}), 26.0 (C^{2}), 23.0 (C^{11}); ^{19}F NMR (471 MHz, $CDCl_3$) δ -148.3; **HRMS** (ESI): calculated for $C_{20}H_{26}FN_2O_2$ $[M+H]^+$ requires *m/z* 345.1973, found *m/z* 345.1975; **Chiral SFC:** YMC Chiral ART Cellulose-SC column (25 cm), CO_2:*i*-PrOH 75:25, 3 mL/min, 228 bar, r.t. Retention times: 2.4 mins (major), 3.3 mins (minor), e.r. = 93.5:6.5.

SFC analysis of the racemate, prepared using rac-BINAP:

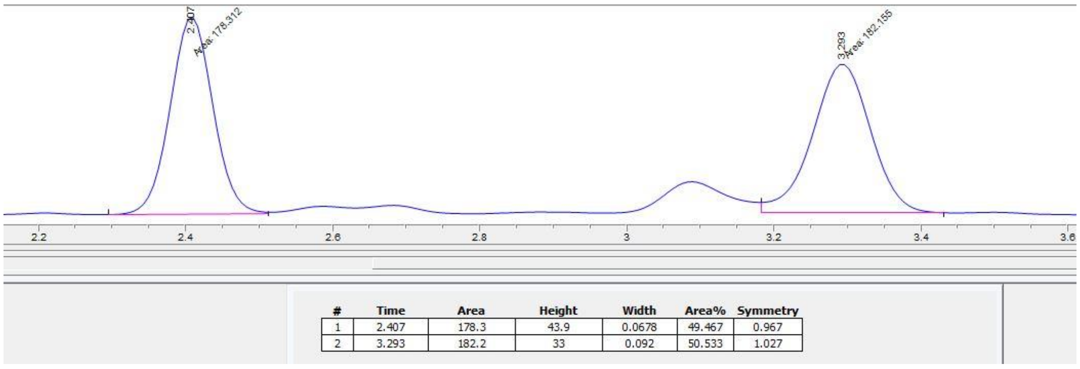

#	Time	Area	Height	Width	Area%	Symmetry
1	2.407	178.3	43.9	0.0678	49.467	0.967
2	3.293	182.2	33	0.092	50.533	1.027

SFC analysis of the enantioenriched material, prepared using (R)-DM-SEGPHOS:

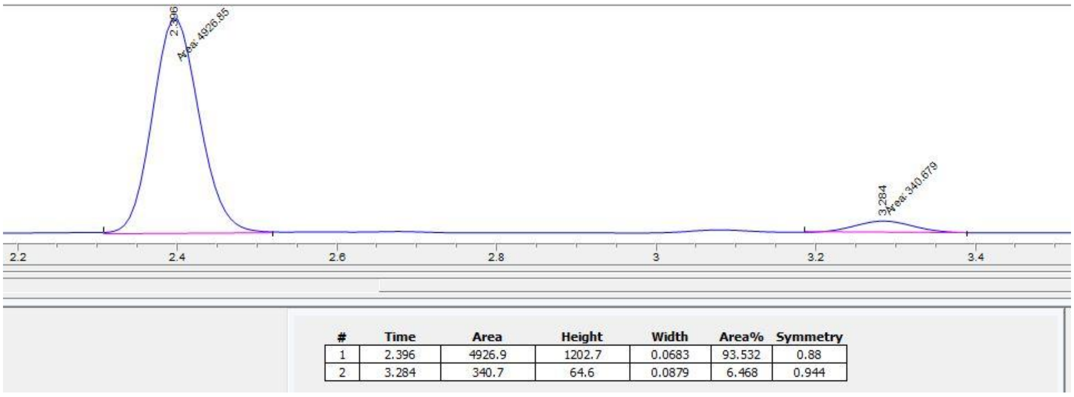

#	Time	Area	Height	Width	Area%	Symmetry
1	2.396	4926.9	1202.7	0.0683	93.532	0.88
2	3.284	340.7	64.6	0.0879	6.468	0.944

(*S*)-2-((3-Fluoro-4-hydroxyphenyl)amino)-*N*,*N*,3-trimethyl-3-phenylbutanamide (3ga):

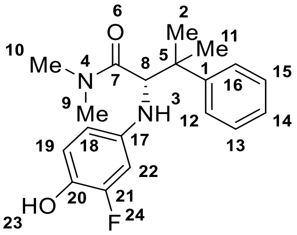

General procedure B: The reaction was carried out with substrate **1g** (21.2 mg, 0.10 mmol, 100 mol%) and prop-1-en-2-ylbenzene **2a** (52.0 µL, 0.40 mmol, 400 mol%). Purification of the residue by FCC (hexane/EtOAc 65:35) afforded the title compound (23.4 mg, 71%, >30:1 B:L, e.r. = 97:3) as a colorless oil. ^{1}H NMR analysis of the crude material gave >30:1 B:L. $[\boldsymbol{\alpha}]_{\boldsymbol{D}}^{\boldsymbol{25}}$ = -11.4 (c = 1.0, $CHCl_3$); **IR (thin film)** ν_{max}/cm^{-1}: 3251 (br), 2969 (s), 1621 (s), 1599 (s), 1516 (s), 1206 (s), 734 (s), 701 (s); **^{1}H NMR** (500 MHz, $CDCl_3$) δ 7.47 (d, *J* = 7.0 Hz, 2H, H^{12} + H^{16}), 7.36 – 7.33 (m, 2H, H^{13} + H^{15}), 7.27 – 7.24 (m, 1H, H^{14}), 6.78 – 6.74 (m, 1H, H^{19}), 6.37 (dd, *J* = 12.5, 2.5 Hz, 1H, H^{22}), 6.32 – 6.29 (m, 1H, H^{18}), 5.14 (br. s, 1H, H^{23}), 4.41 (d, *J* = 10.0 Hz, 1H, H^3), 4.17 (d, *J* = 10.0 Hz, 1H, H^8), 2.72 (s, 3H, H^9), 2.29 (s, 3H, H^{10}), 1.60 (s, 3H, H^2), 1.50 (s, 3H, H^{11}); **^{13}C NMR** (126 MHz, $CDCl_3$) δ 172.3 (C^7), 151.5 (d, *J* = 235.0 Hz, C^{21}), 146.1 (C^1), 142.5 (d, *J* = 7.5 Hz, C^{17}), 135.9 (d, *J* = 15.0 Hz, C^{20}), 128.1 (C^{13} + C^{15}), 126.8 (C^{12} + C^{16}), 126.6 (C^{14}), 117.9 (d, *J* = 2.5 Hz, C^{19}), 111.0 (d, *J* = 2.5 Hz, C^{18}), 102.4 (d, *J* = 21.3 Hz, C^{22}), 63.1 (C^8), 42.5 (C^5), 36.8 (C^9), 35.3 (C^{10}), 26.2 (C^2), 22.9 (C^{11}); ^{19}F NMR (471 MHz, $CDCl_3$) δ -138.4; **HRMS** (ESI): calculated for $C_{19}H_{24}FN_2O_2$ $[M+H]^+$ requires *m/z* 331.1816, found *m/z* 331.1821; **Chiral SFC:** YMC Chiral ART Cellulose-SC column (25 cm), CO_2:*i*-PrOH 75:25, 3 mL/min, 228 bar, r.t. Retention times: 2.4 mins (major), 3.3 mins (minor), e.r. = 97:3.

SFC analysis of the racemate, prepared using rac-BINAP:

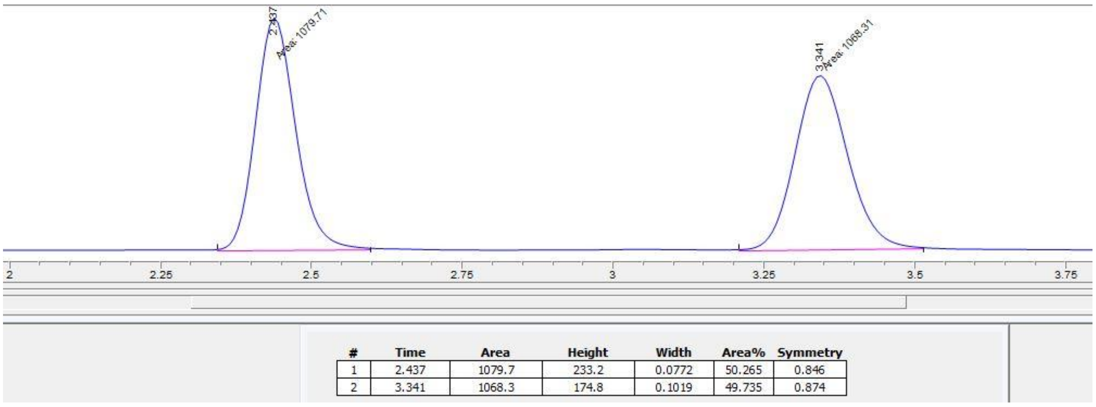

#	Time	Area	Height	Width	Area%	Symmetry
1	2.437	1079.7	233.2	0.0772	50.265	0.846
2	3.341	1068.3	174.8	0.1019	49.735	0.874

SFC analysis of the enantioenriched material, prepared using (R)-DM-SEGPHOS:

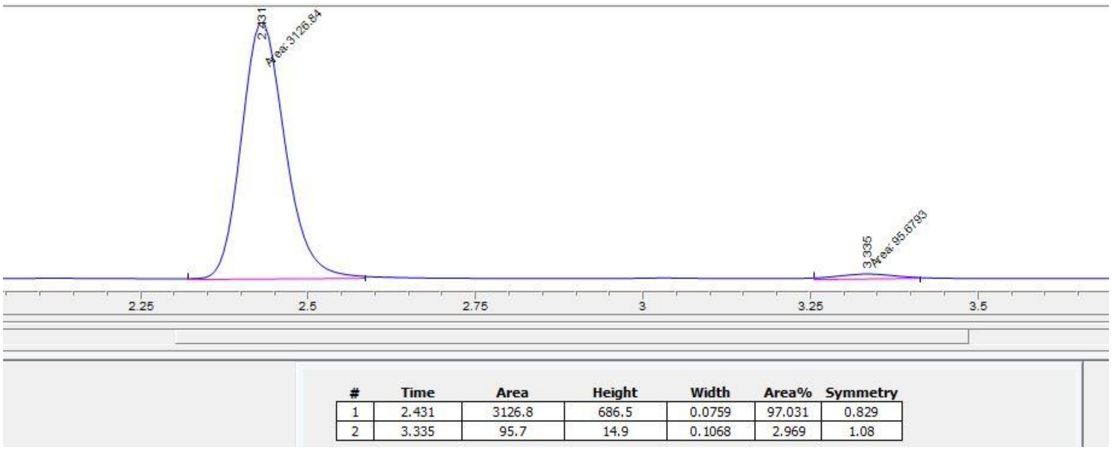

#	Time	Area	Height	Width	Area%	Symmetry
1	2.431	3126.8	686.5	0.0759	97.031	0.829
2	3.335	95.7	14.9	0.1068	2.969	1.08

(*S*)-*N*,*N*-Dibenzyl-2-((4-hydroxyphenyl)amino)-3-methyl-3-phenylbutanamide (3ha):

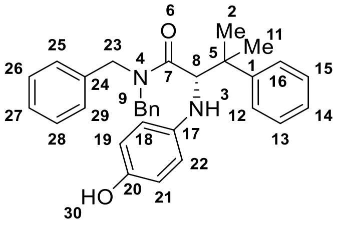

General procedure B: The reaction was carried out with substrate **1h** (34.6 mg, 0.10 mmol, 100 mol%), prop-1-en-2-ylbenzene **2a** (130 µL, 1.00 mmol, 1000 mol%), [Ir(cod)$_2$]BARF (12.7 mg, 10.0 µmol, 10 mol%) and (*R*)-DM-SEGPHOS (7.23 mg, 10.0 µmol, 10 mol%). Purification of the residue by FCC (hexane/EtOAc 60:40) afforded the title compound (13.9 mg, 30%, >30:1 B:L, e.r. = 88:12) as a pale-yellow oil. ^{1}H NMR analysis of the crude material gave >30:1 B:L. $[\boldsymbol{\alpha}]_{\boldsymbol{D}}^{\mathbf{25}}$ = -62.7 (c = 1.0, CHCl$_3$); **IR (thin film)** ν_{max}/cm^{-1}: 3345 (br), 2926 (s), 1615 (s), 1513 (s), 1496 (s), 1214 (s), 735 (s), 699 (s); **^{1}H NMR** (500 MHz, CDCl$_3$) δ 7.40 – 7.39 (m, 2H, ArH), 7.31 – 7.30 (m, 3H, ArH), 7.19 – 7.06 (m, 8H, ArH), 6.63 – 6.52 (m, 6H, ArH + H^{18} + H^{19} + H^{21} + H^{22}), 5.12 (d, *J* = 14.5 Hz, 1H, H^{23}), 4.90 (br. s, 1H, H^{30}), 4.19 (s, 1H, H^8), 3.99 (d, *J* = 17.0 Hz, 1H, H^{23}), 3.54 (d, *J* = 14.5 Hz, 1H, H^{23}), 3.37 (d, *J* = 17.0 Hz, 1H,

H[23]), 1.62 (s, 3H, H[2]), 1.57 (s, 3H, H[11]); **^{13}C NMR** (126 MHz, $CDCl_3$) δ 172.2 (C[7]), 146.2 (C[1]), 136.5 (C[24]), 135.5 (C[24]), 129.2 (ArC), 128.7 (ArC), 128.4 (ArC), 128.2 (ArC), 127.5 (ArC), 126.9 (ArC), 126.6 (ArC), 126.5 (ArC), 118.3 (C[19] + C[21]), 116.1 (C[18] + C[22]), 61.2 (C[8]), 48.9 (C[23]), 48.0 (C[23]), 42.6 (C[5]), 26.7 (C[2]), 23.2 (C[11]); **HRMS** (ESl): calculated for $C_{31}H_{33}N_2O_2$ $[M+H]^+$ requires *m/z* 465.2537, found *m/z* 465.2546; **Chiral SFC:** YMC Chiral ART Cellulose-SC column (25 cm), CO_2:*i*-PrOH 75:25, 2 mL/min, 187 bar, r.t. Retention times: 3.7 mins (major), 4.3 mins (minor), e.r. = 88:12.

SFC analysis of the racemate, prepared using rac-BINAP:

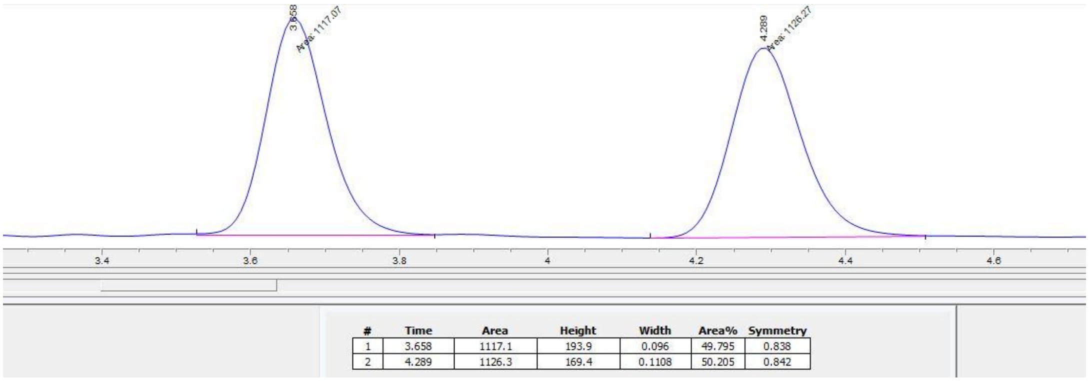

#	Time	Area	Height	Width	Area%	Symmetry
1	3.658	1117.1	193.9	0.096	49.795	0.838
2	4.289	1126.3	169.4	0.1108	50.205	0.842

SFC analysis of the enantioenriched material, prepared using (R)-DM-SEGPHOS:

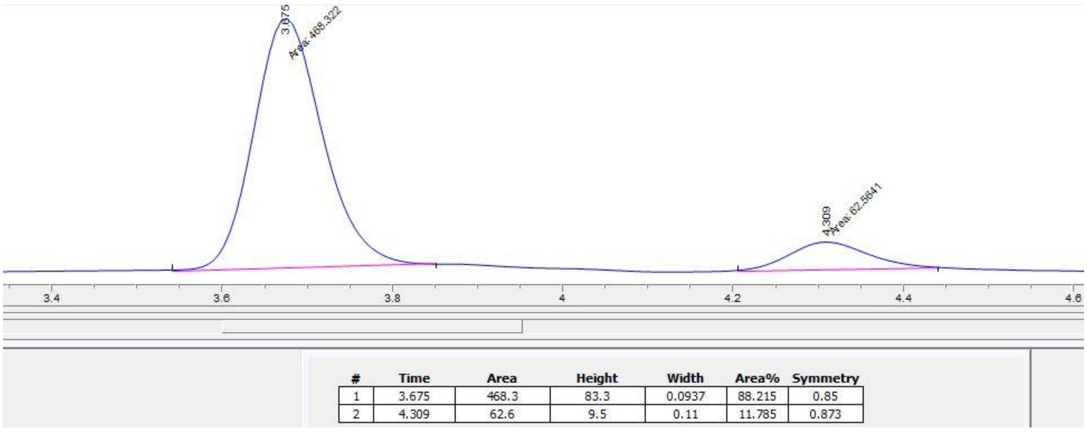

#	Time	Area	Height	Width	Area%	Symmetry
1	3.675	468.3	83.3	0.0937	88.215	0.85
2	4.309	62.6	9.5	0.11	11.785	0.873

(*S*)-2-((4-Hydroxyphenyl)amino)-3-methyl-3-phenyl-1-(pyrrolidin-1-yl)butan-1-one (3ia):

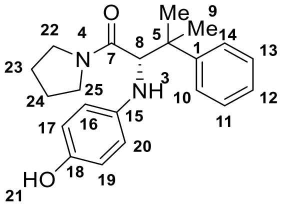

General procedure B: The reaction was carried out with substrate **1i** (22.0 mg, 0.10 mmol, 100 mol%) and prop-1-en-2-ylbenzene **2a** (52.0 µL, 0.40 mmol, 400 mol%). Purification of the residue by FCC (hexane/EtOAc 60:40) afforded the title compound (29.1 mg, 86%, >30:1 B:L, e.r. = 97:3) as a pale-yellow oil. ^{1}H NMR analysis of the crude material gave >30:1 B:L. $[\boldsymbol{\alpha}]_D^{25}$ = -11.0 (c = 1.0, $CHCl_3$); **IR (thin film)** ν_{max}/cm^{-1}: 3349 (br), 2972 (s), 1611 (s), 1513 (s), 1447 (s), 1240 (s), 734 (s), 700 (s); **^{1}H NMR** (500 MHz, $CDCl_3$) δ 7.51 (d, *J* = 7.0 Hz, 2H, H^{10} + H^{14}), 7.34 – 7.31 (m, 2H, H^{11} + H^{13}), 7.25 – 7.22 (m, 1H, H^{12}), 6.69 (d, *J* = 8.0 Hz, 2H, H^{17} + H^{19}), 6.56 (d, *J* = 8.0 Hz, 2H, H^{16} + H^{20}), 6.45 (br. s, 1H, H^{21}), 4.30 (br. s, 1H, H^3), 3.97 (s, 1H, H^8), 3.32 – 3.27 (m, 1H, H^{22}), 3.17 – 3.12 (m, 1H, H^{22}), 2.98 – 2.93 (m, 1H, H^{25}), 2.34 – 2.29 (m, 1H, H^{25}), 1.63 (s, 3H, H^2), 1.60 – 1.44 (m, 6H, H^9 + H^{23} + H^{24}), 1.34 – 1.27 (m, 1H, H^{24}); **^{13}C NMR** (126 MHz, $CDCl_3$) δ 171.3 (C^7), 149.4 (C^{15}), 146.2 (C^1), 141.4 (C^{18}), 128.0 (C^{11} + C^{13}), 126.9 (C^{10} + C^{14}), 126.5 (C^{12}), 116.6 (C^{16} + C^{20}), 116.2 (C^{17} + C^{19}), 66.6 (C^8), 46.5 (C^{22}), 45.5 (C^{25}), 42.3 (C^5), 26.4 (C^2), 25.7 (C^{23}), 23.7 (C^{24}), 22.9 (C^9); **HRMS** (ESl): calculated for $C_{21}H_{27}N_2O_2$ $[M+H]^+$ requires *m/z* 339.2067, found *m/z* 339.2071; **Chiral SFC:** YMC Chiral ART Cellulose-SC column (25 cm, CO_2:*i*-PrOH 75:25, 3 mL/min, 228 bar, r.t. Retention times: 4.4 mins (major), 7.1 mins (minor), e.r. = 97:3.

SFC analysis of the racemate, prepared using rac-BINAP:

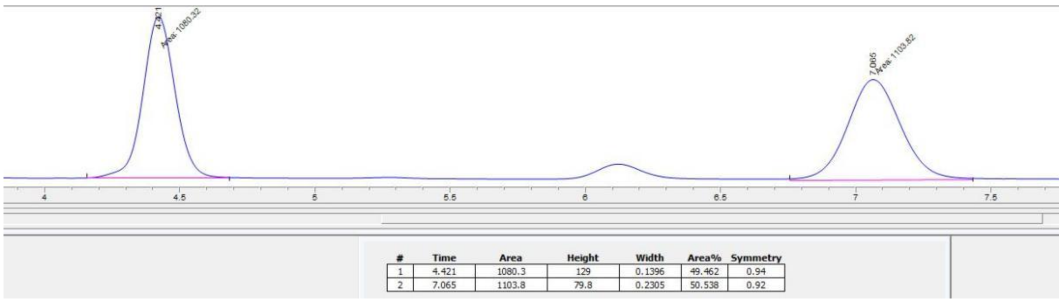

SFC analysis of the enantioenriched material, prepared using (R)-DM-SEGPHOS:

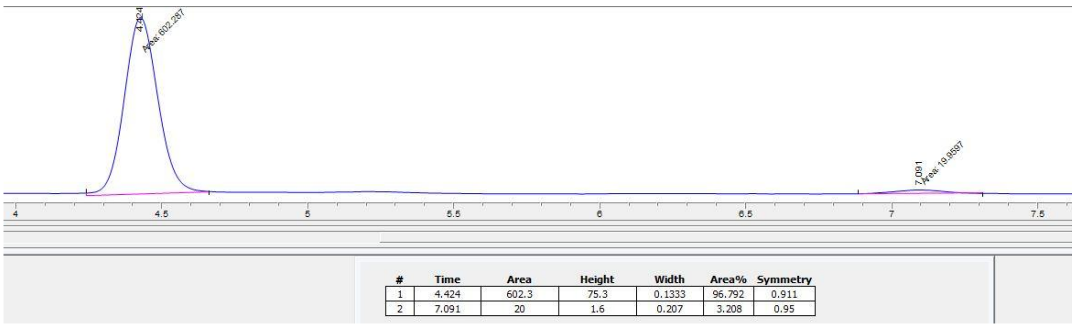

(*S*)-2-((4-Hydroxyphenyl)amino)-3-methyl-3-phenyl-1-(piperidin-1-yl)butan-1-one (3ja):

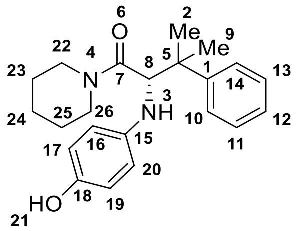

General procedure B: The reaction was carried out with substrate **1j** (23.4 mg, 0.10 mmol, 100 mol%) and prop-1-en-2-ylbenzene **2a** (52.0 µL, 0.40 mmol, 400 mol%). Purification of the residue by FCC (hexane/EtOAc 65:35) afforded the title compound (21.5 mg, 61%, >30:1 B:L, e.r. = 96:4) as a pale-yellow solid. ^{1}H NMR analysis of the crude material gave >30:1 B:L. **m.p.** = 133 – 135 °C (hexane/EtOAc); $[\boldsymbol{\alpha}]_{\boldsymbol{D}}^{\mathbf{25}}$ = -9.7 (c = 1.0, $CHCl_3$); **IR (thin film)** ν_{max}/cm^{-1}: 3383 (br), 2935 (s), 1609 (s), 1513 (s), 1444 (s), 1241 (s), 735 (s), 700 (s); **^{1}H NMR** (500 MHz, $CDCl_3$) δ 7.48 (d, *J* = 7.0 Hz, 2H, H^{10} + H^{14}), 7.35 – 7.32 (m, 2H, H^{11} + H^{13}), 7.25 – 7.23 (m, 1H, H^{12}), 6.61 (d, *J* = 8.5 Hz, 2H, H^{17} + H^{19}), 6.53 (d, *J* = 8.0 Hz, 2H, H^{16} + H^{20}), 5.77 (br. s, 1H, H^{21}), 4.26 (s, 1H, H^{8}), 3.70 – 3.65 (m, 1H, H^{22}), 3.14 – 3.09 (m, 1H, H^{22}), 3.01 – 2.95 (m, 1H, H^{26}), 2.48 – 2.43 (m, 1H, H^{26}), 1.62 (s, 3H, H^{2}), 1.53 (s, 3H, H^{9}), 1.47 – 1.41 (m, 1H, H^{23}), 1.34 – 1.23 (m, 3H, H^{23} + H^{25}), 1.08 – 0.95 (m, 2H, H^{24}); **^{13}C NMR** (126 MHz, $CDCl_3$) δ 170.9 (C^{7}), 149.4 (C^{15}), 146.4 (C^{1}), 141.3 (C^{18}), 128.1 (C^{11} + C^{13}), 126.9 (C^{10} + C^{14}), 126.4 (C^{12}), 117.0 (C^{16} + C^{20}), 116.1 (C^{17} + C^{19}), 63.5 (C^{8}), 46.6 (C^{22}), 42.8 (C^{26}), 42.0 (C^{5}), 26.5 (C^{2}), 25.9 (C^{23}), 25.3 (C^{25}), 24.1 (C^{24}), 23.3 (C^{9}); **HRMS** (ESI): calculated for $C_{22}H_{29}N_2O_2$ $[M+H]^+$ requires *m/z* 353.2224, found *m/z* 353.2229; **Chiral SFC:** YMC Chiral ART Cellulose-SC column (25 cm), CO_2:*i*-PrOH 75:25, 3 mL/min, 228 bar, r.t. Retention times: 3.1 mins (major), 4.6 mins (minor), e.r. = 96:4.

SFC analysis of the racemate, prepared using rac-BINAP:

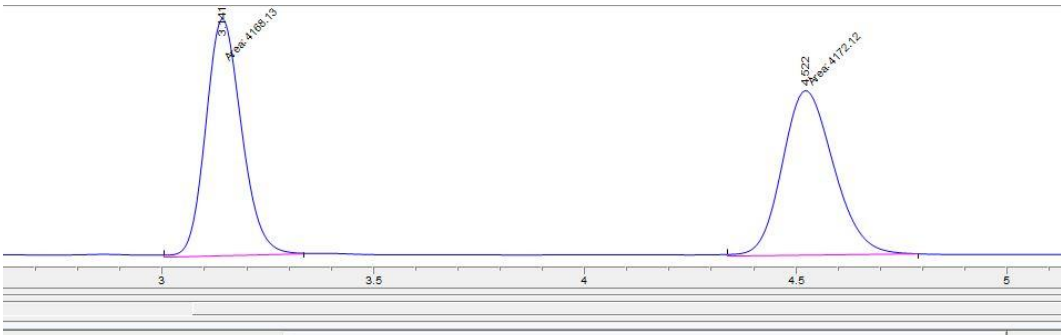

#	Time	Area	Height	Width	Area%	Symmetry
1	3.141	4168.1	706.8	0.0983	49.976	0.842
2	4.522	4172.1	489.5	0.1421	50.024	0.817

SFC analysis of the enantioenriched material, prepared using (R)-DM-SEGPHOS:

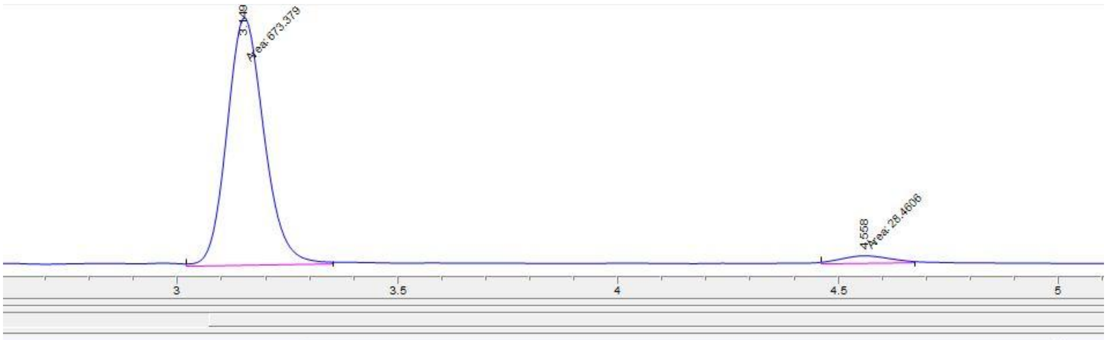

#	Time	Area	Height	Width	Area%	Symmetry
1	3.149	673.4	114.7	0.0978	95.945	0.853
2	4.558	28.5	3.6	0.1325	4.055	0.925

(*S*)-2-((4-Hydroxyphenyl)amino)-3-methyl-1-morpholino-3-phenylbutan-1-one (3ka):

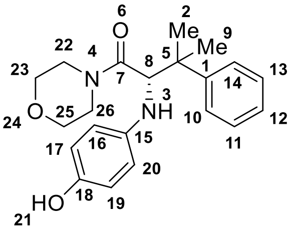

General procedure B: The reaction was carried out with substrate **1k** (23.6 mg, 0.10 mmol, 100 mol%), prop-1-en-2-ylbenzene **2a** (52.0 µL, 0.40 mmol, 400 mol%) and was run in *t*-BuOH (0.2 mL). Purification of the residue by FCC (hexane/EtOAc 65:35) afforded the title compound (21.3 mg, 60%, >30:1 B:L, e.r. = 97:3) as a pale-yellow oil. ^{1}H NMR analysis of the crude material gave >30:1 B:L. $[\alpha]_D^{25}$ = -15.9 (c = 1.0, $CHCl_3$); **IR (thin film)** ν_{max}/cm^{-1}: 3269 (br), 2856 (s), 1606 (s), 1593 (s), 1517 (s), 1243 (s), 1220 (s), 701 (s); **^{1}H NMR** (500 MHz, $CDCl_3$) δ 7.48 (d, *J* = 7.5 Hz, 2H, $H^{10} + H^{14}$), 7.37 – 7.34 (m, 2H, $H^{11} + H^{13}$), 7.29 – 7.26 (m, 1H, H^{12}), 6.65 (d, *J* = 8.5 Hz, 2H, $H^{17} + H^{19}$), 6.57 (d, *J* = 8.5 Hz, 2H, $H^{16} + H^{20}$), 5.23 (br. s, 1H, H^{21}), 4.18 (s, 1H, H^{8}), 3.49 – 3.25 (m, 4H, $H^{23} + H^{25}$), 3.16 – 3.12 (m, 1H, H^{22}), 3.05 – 3.01 (m, 1H, H^{22}), 2.84 – 2.80 (m, 1H, H^{26}), 2.57 – 2.52 (m, 1H, H^{26}), 1.62 (s, 3H, H^{2}), 1.56 (s, 3H, H^{9}); **^{13}C NMR** (126 MHz, $CDCl_3$) δ 171.6 (C^{7}), 149.2 (C^{15}), 146.2 (C^{1}), 141.5 (C^{18}), 128.3 ($C^{11} + C^{13}$), 126.9 ($C^{10} + C^{14}$), 126.8 (C^{12}), 116.9 ($C^{16} + C^{20}$), 116.2 ($C^{17} + C^{19}$), 66.5 (C^{23}), 65.9 (C^{25}), 63.4 (C^{8}), 45.9 (C^{22}), 42.1 (C^{5}), 41.9 (C^{26}), 27.0 (C^{2}), 22.6 (C^{9}); **HRMS** (ESI): calculated for $C_{21}H_{27}N_2O_3$ $[M+H]^+$ requires *m/z* 355.2016, found *m/z* 355.2022; **Chiral SFC:** YMC Chiral ART Cellulose-SB column (25 cm), CO_2:*i*-PrOH 75:25, 3 mL/min, 223 bar, r.t. Retention times: 2.4 mins (major), 3.0 mins (minor), e.r. = 97:3.

SFC analysis of the racemate, prepared using rac-BINAP:

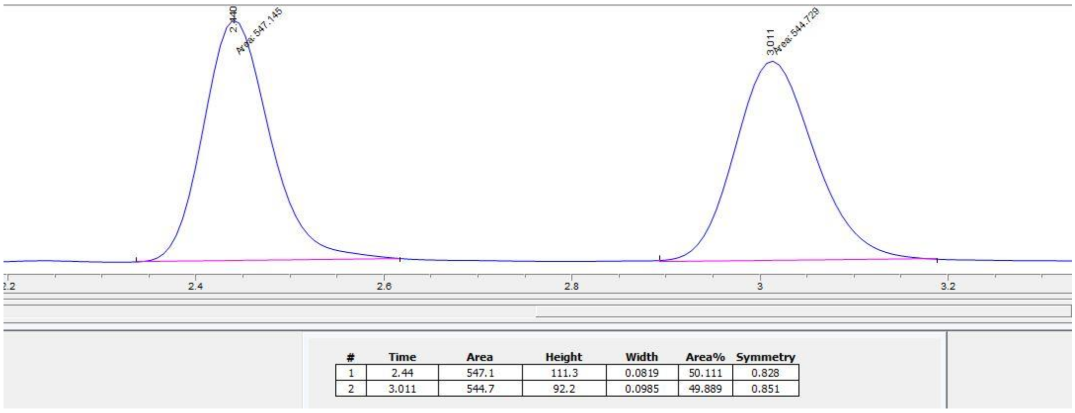

#	Time	Area	Height	Width	Area%	Symmetry
1	2.44	547.1	111.3	0.0819	50.111	0.828
2	3.011	544.7	92.2	0.0985	49.889	0.851

SFC analysis of the enantioenriched material, prepared using (R)-DM-SEGPHOS:

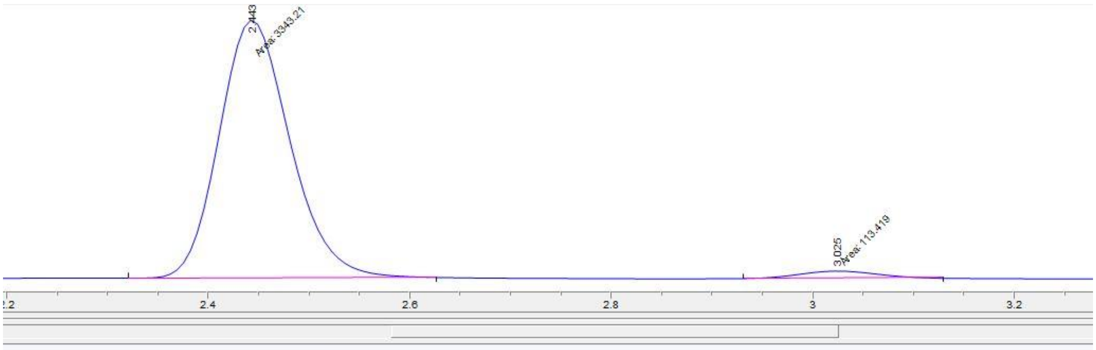

#	Time	Area	Height	Width	Area%	Symmetry
1	2.443	3343.2	679.7	0.082	96.719	0.827
2	3.025	113.4	20.2	0.0934	3.281	1.007

(*S*)-2-((4-Hydroxyphenyl)amino)-3-methyl-*N*,3-diphenylbutanamide (3la):

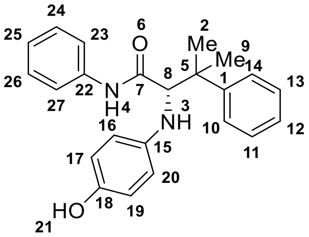

General procedure B: The reaction was carried out with substrate **1l** (24.2 mg, 0.10 mmol, 100 mol%), prop-1-en-2-ylbenzene **2a** (52.0 µL, 0.40 mmol, 400 mol%) and was run in *t*-BuOH (0.2 mL). Purification of the residue by FCC (hexane/EtOAc 60:40) afforded the title compound (15.9 mg, 44%, >30:1 B:L, e.r. = 97:3) as a pale-yellow solid. ^{1}H NMR analysis of the crude material gave >30:1 B:L. **m.p.** = 159 – 161 °C (hexane/EtOAc); $[\boldsymbol{\alpha}]_{\boldsymbol{D}}^{\boldsymbol{25}}$ = -12.7 (c = 1.0, $CHCl_3$); **IR (thin film)** ν_{max}/cm^{-1}: 3314 (br), 2919 (s), 1652 (s), 1517 (s), 1442 (s), 1237 (s), 751 (s), 700 (s); ^{1}**H NMR** (500 MHz, $CDCl_3$) δ 8.62 (s, 1H, H^4), 7.47 – 7.45 (m, 2H, ArH), 7.42 – 7.41 (m, 2H, ArH), 7.39 – 7.35 (m, 2H, ArH), 7.33 – 7.29 (m, 3H, ArH), 7.14 – 7.11 (m, 1H, H^{25}), 6.65 (d, *J* = 8.5 Hz, 2H, $H^{17} + H^{19}$), 6.36 (d, *J* = 8.5 Hz, 2H, $H^{16} + H^{20}$), 5.60 (br.

s, 1H, H[21]), 3.79 (s, 1H, H[8]), 3.54 (br. s, 1H, H[3]), 1.61 (s, 3H, H[2]), 1.56 (s, 3H, H[9]); **^{13}C NMR** (126 MHz, $CDCl_3$) δ 170.7 (C[7]), 149.7 (C[15]), 145.5 (C[1]), 140.8 (C[18]), 137.2 (ArC), 129.0 (ArC), 128.9 (ArC), 127.0 (ArC), 126.1 (ArC), 124.6 (ArC), 120.1 (C[25]), 116.3 (C[17] + C[19]), 116.0 (C[16] + C[20]), 70.4 (C[8]), 42.2 (C[5]), 28.5 (C[2]), 23.1 (C[9]); **HRMS** (ESI): calculated for $C_{23}H_{25}N_2O_2$ $[M+H]^+$ requires *m/z* 361.1911, found *m/z* 361.1917; **Chiral SFC:** YMC Chiral ART Cellulose-SC column (25 cm), CO_2:*i*-PrOH 75:25, 3 mL/min, 228 bar, r.t. Retention times: 2.6 mins (major), 2.8 mins (minor), e.r. = 97:3.

SFC analysis of the racemate, prepared using rac-BINAP:

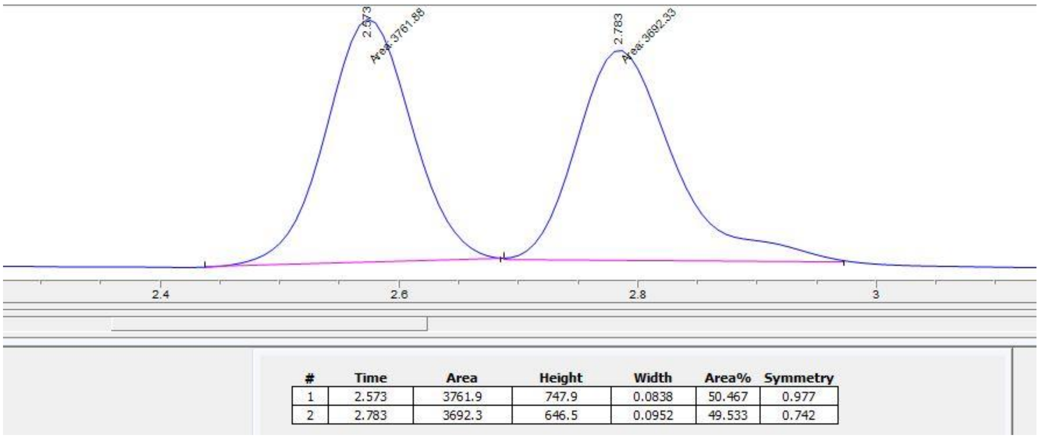

#	Time	Area	Height	Width	Area%	Symmetry
1	2.573	3761.9	747.9	0.0838	50.467	0.977
2	2.783	3692.3	646.5	0.0952	49.533	0.742

SFC analysis of the enantioenriched material, prepared using (R)-DM-SEGPHOS:

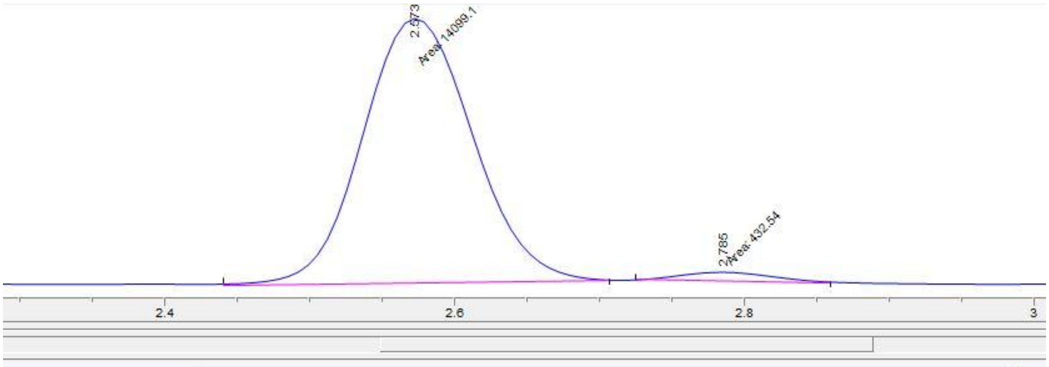

#	Time	Area	Height	Width	Area%	Symmetry
1	2.573	14099.1	2657.9	0.0884	97.023	0.899
2	2.785	432.5	93.6	0.077	2.977	0.686

(*S*)-2-((4-Hydroxyphenyl)amino)-3-methyl-1,3-diphenylbutan-1-one (3ma):

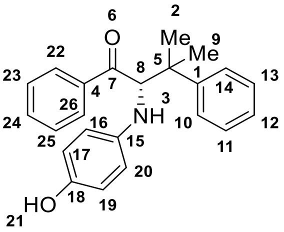

General procedure B: The reaction was carried out with substrate **1m** (22.7 mg, 0.10 mmol, 100 mol%), prop-1-en-2-ylbenzene **2a** (52.0 µL, 0.40 mmol, 400 mol%) and (*R*)-DM-SDP (5.25 mg, 7.50 µmol, 7.5 mol%) for 45 hours. Purification of the residue by FCC (hexane/EtOAc 75:25) afforded the title compound (26.9 mg, 78%, >30:1 B:L, e.r. = 61:39) as a brown solid. ^{1}H NMR analysis of the crude material gave >30:1 B:L. **m.p.** = 85 – 87 °C (hexane/EtOAc); $[\boldsymbol{\alpha}]_{\boldsymbol{D}}^{\boldsymbol{25}}$ = +3.5 (c = 1.0, $CHCl_3$); **IR (thin film)** ν_{max}/cm^{-1}: 3333 (br), 1674 (s), 1516 (s), 1445 (s), 1220 (s), 821 (s), 755 (s), 688 (s); **^{1}H NMR** (500 MHz, $CDCl_3$) δ 7.65 (d, *J* = 7.0 Hz, 2H, H^{22} + H^{26}), 7.45 – 7.42 (m, 1H, H^{24}), 7.38 (d, *J* = 7.0 Hz, 2H, H^{10} + H^{14}), 7.32 – 7.29 (m, 2H, H^{23} + H^{25}), 7.22 – 7.19 (m, 2H, H^{11} + H^{13}), 7.12 – 7.09 (m, 1H, H^{12}), 6.62 (d, *J* = 9.0 Hz, 2H, H^{17} + H^{19}), 6.55 (d, *J* = 9.0 Hz, 2H, H^{16} + H^{20}), 5.01 (s, 1H, H^{8}), 4.68 (br. s, 1H, H^{21}), 1.55 (s, 3H, H^{2}), 1.46 (s, 3H, H^{9}); **^{13}C NMR** (126 MHz, $CDCl_3$) δ 203.5 (C^{7}), 148.7 (C^{15}), 145.6 (ArC), 141.9 (C^{18}), 138.3 (ArC), 132.8 (ArC), 128.3 (ArC), 128.1 (ArC), 128.0 (ArC), 126.8 (ArC), 126.5 (ArC), 116.6 (C^{17} + C^{19}), 116.1 (C^{16} + C^{20}), 68.1 (C^{8}), 42.3 (C^{5}), 26.1 (C^{2}), 24.9 (C^{9}); **HRMS** (ESI): calculated for $C_{23}H_{24}NO_2$ $[M+H]^+$ requires *m/z* 346.1802, found *m/z* 346.1807; **Chiral SFC:** YMC Chiral ART Cellulose-SC column (25 cm), CO_2:*i*-PrOH 80:20, 2 mL/min, 178 bar, r.t. Retention times: 4.1 mins (major), 4.7 mins (minor), e.r. = 61:39.

SFC analysis of the racemate, prepared using rac-BINAP:

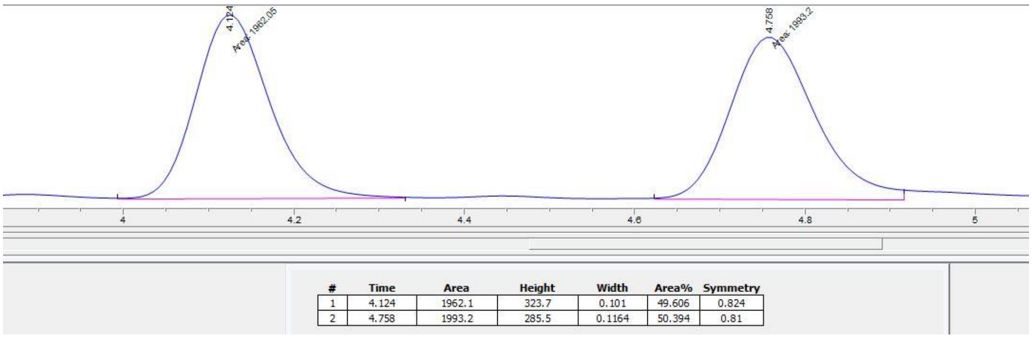

#	Time	Area	Height	Width	Area%	Symmetry
1	4.124	1962.1	323.7	0.101	49.606	0.824
2	4.758	1993.2	285.5	0.1164	50.394	0.81

SFC analysis of the enantioenriched material, prepared using (R)-DM-SDP:

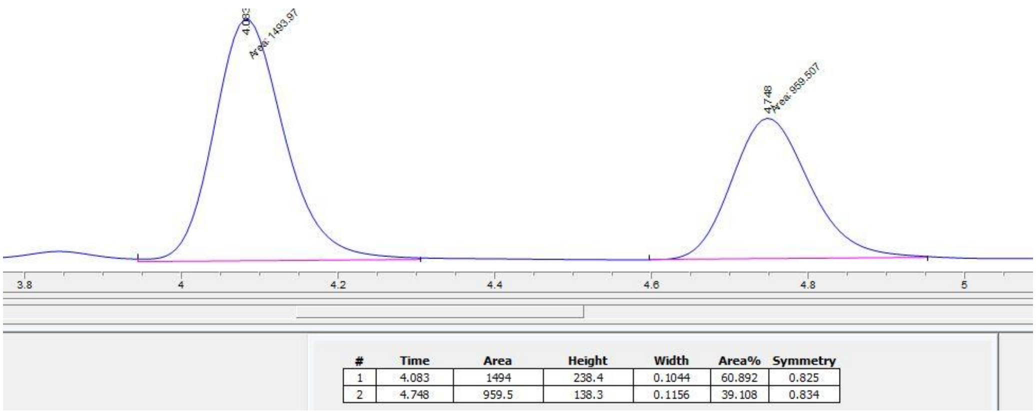

#	Time	Area	Height	Width	Area%	Symmetry
1	4.083	1494	238.4	0.1044	60.892	0.825
2	4.748	959.5	138.3	0.1156	39.108	0.834

(*S*)-2-((4-Hydroxyphenyl)amino)-3-methyl-1-(pyrrolidin-1-yl)-3-(*p*-tolyl)butan-1-one (3ib):

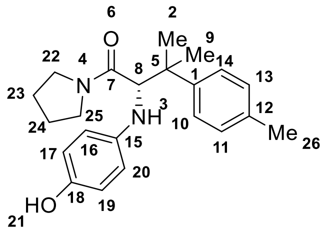

General procedure B: The reaction was carried out with substrate **1i** (22.0 mg, 0.10 mmol, 100 mol%) and 1-methyl-4-(prop-1-en-2-yl)benzene **2b** (58.0 µL, 0.40 mmol, 400 mol%). Purification of the residue by FCC (hexane/EtOAc 55:45) afforded the title compound (25.0 mg, 71%, >30:1 B:L, e.r. = 98:2) as a pale-yellow oil. ^{1}H NMR analysis of the crude material gave >30:1 B:L. $[\alpha]_D^{25}$ = -11.6 (c = 1.0, $CHCl_3$); **IR (thin film)** ν_{max}/cm^{-1}: 3354 (br), 2972 (s), 1612 (s), 1513 (s), 1450 (s), 1241 (s), 818 (s), 735 (s); **^{1}H NMR** (500 MHz, $CDCl_3$) δ 7.38 (d, *J* = 8.5 Hz, 2H, H^{10} + H^{14}), 7.13 (d, *J* = 8.5 Hz, 2H, H^{11} + H^{13}), 6.63 (d, *J* = 9.0 Hz, 2H, H^{17} + H^{19}), 6.52 (d, *J* = 9.0 Hz, 2H, H^{16} + H^{20}), 6.20 (br. s, 1H, H^{21}), 4.21 (br. s, 1H, H^3), 3.95 (s, 1H, H^8), 3.33 – 3.28 (m, 1H, H^{22}), 3.19 – 3.13 (m, 1H, H^{22}), 3.00 – 2.96 (m, 1H, H^{25}), 2.40 – 2.35 (m, 1H, H^{25}), 2.34 (s, 3H, H^{26}), 1.62 – 1.55 (m, 5H, H^2 + H^{23}), 1.54 – 1.46 (m, 4H, H^9 + H^{24}), 1.36 – 1.30 (m, 1H, H^{24}); **^{13}C NMR** (126 MHz, $CDCl_3$) δ 171.5 (C^7), 149.1 (C^{15}), 143.2 (C^1), 141.7 (C^{18}), 136.0 (C^{12}), 128.6 (C^{11} + C^{13}), 126.8 (C^{10} + C^{14}), 116.4 (ArC), 116.2 (ArC), 66.4 (C^8), 46.5 (C^{22}), 45.5 (C^{25}), 41.9 (C^5), 26.4 (C^2), 25.7 (C^{23}), 23.8 (C^{24}), 23.0 (C^9), 20.9 (C^{26}); **HRMS** (ESl): calculated for $C_{22}H_{29}N_2O_2$ $[M+H]^+$ requires *m/z* 353.2224, found *m/z* 353.2225; **Chiral SFC:** YMC Chiral ART Cellulose-SC column (25 cm), CO_2:*i*-PrOH 75:25, 3 mL/min, 228 bar, r.t. Retention times: 4.8 mins (major), 5.4 mins (minor), e.r. = 98:2.

SFC analysis of the racemate, prepared using rac-BINAP:

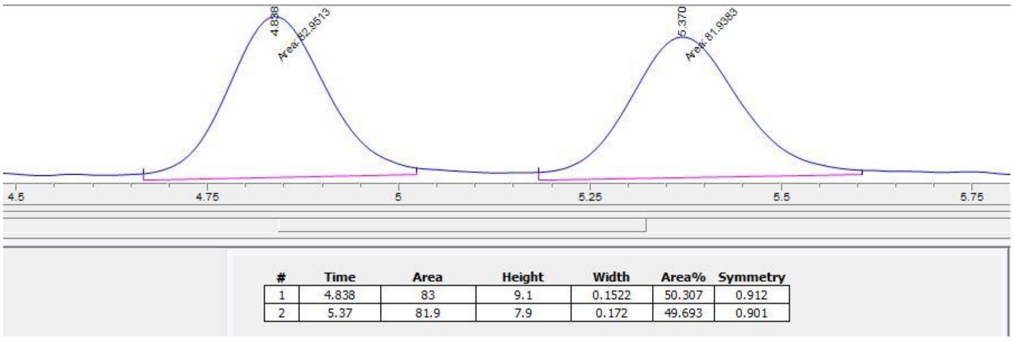

#	Time	Area	Height	Width	Area%	Symmetry
1	4.838	83	9.1	0.1522	50.307	0.912
2	5.37	81.9	7.9	0.172	49.693	0.901

SFC analysis of the enantioenriched material, prepared using (R)-DM-SEGPHOS:

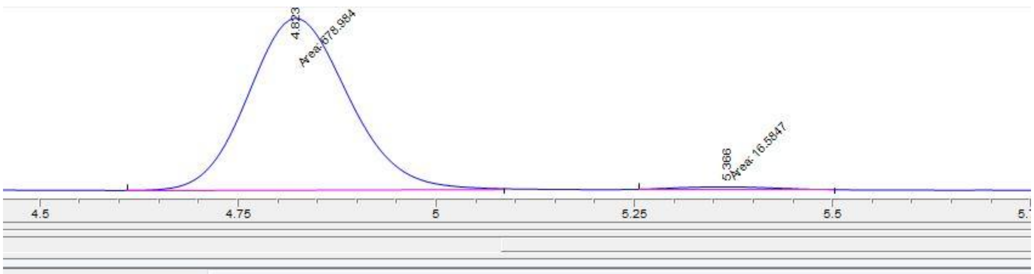

#	Time	Area	Height	Width	Area%	Symmetry
1	4.823	679	76.4	0.1481	97.616	0.879
2	5.366	16.6	1.7	0.1671	2.384	0.929

(*S*)-2-((4-Hydroxyphenyl)amino)-3-(4-methoxyphenyl)-3-methyl-1-(pyrrolidin-1-yl)butan-1-one (3ic):

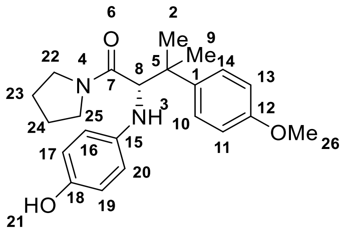

General procedure B: The reaction was carried out with substrate **1i** (22.0 mg, 0.10 mmol, 100 mol%) and 1-methoxy-4-(prop-1-en-2-yl)benzene **2c** (59.3 mg, 0.40 mmol, 400 mol%). Purification of the residue by FCC (hexane/EtOAc 50:50) afforded the title compound (22.1 mg, 60%, >30:1 B:L, e.r. = 98:2) as a pale-yellow oil. ^{1}H NMR analysis of the crude material gave >30:1 B:L. $[\boldsymbol{\alpha}]_{\boldsymbol{D}}^{\mathbf{25}}$ = -6.6 (c = 1.0, $CHCl_3$); **IR (thin film)** ν_{max}/cm^{-1}: 3342 (br), 2970 (s), 1611 (s), 1512 (s), 1449 (s), 1248 (s), 830 (s), 736 (s); **^{1}H NMR** (500 MHz, $CDCl_3$) δ 7.41 (d, *J* = 9.0 Hz, 2H, H^{10} + H^{14}), 6.86 (d, *J* = 9.0 Hz, 2H, H^{11} + H^{13}), 6.63 (d, *J* = 9.0 Hz, 2H, H^{17} + H^{19}), 6.52 (d, *J* = 9.0 Hz, 2H, H^{16} + H^{20}), 5.94 (br. s, 1H, H^{21}), 4.21 (br. s, 1H, H^3), 3.93 (s, 1H, H^8), 3.81 (s, 3H, H^{26}), 3.34 – 3.29 (m, 1H, H^{22}), 3.20 – 3.15 (m, 1H, H^{22}), 3.03 – 2.99 (m, 1H, H^{25}), 2.44 – 2.39 (m, 1H, H^{25}), 1.64 – 1.56 (m, 5H, H^2 + H^{23}), 1.55 – 1.48 (m, 4H, H^9 + H^{24}), 1.42 – 1.35 (m, 1H, H^{24}); **^{13}C NMR** (126 MHz, $CDCl_3$) δ 171.5 (C^7), 158.1 (C^{12}), 149.0 (C^{15}), 141.8 (C^{18}), 138.4 (C^1), 128.0 (C^{10} + C^{14}), 116.4 (ArC), 116.2 (ArC), 113.2 (C^{11} + C^{13}), 66.5 (C^8), 55.4 (C^{26}), 46.5 (C^{22}), 45.5 (C^{25}), 41.6 (C^5), 26.5 (C^2), 25.8 (C^{23}), 23.8 (C^{24}), 23.2 (C^9); **HRMS** (ESI): calculated for $C_{22}H_{29}N_2O_3$ $[M+H]^+$ requires *m/z* 369.2173, found *m/z* 369.2178; **Chiral SFC:** YMC Chiral ART Cellulose-SC column (25 cm), CO_2:*i*-PrOH 80:20, 2 mL/min, 178 bar, r.t. Retention times: 12.4 mins (minor), 13.5 mins (major), e.r. = 98:2.

SFC analysis of the racemate, prepared using rac-BINAP:

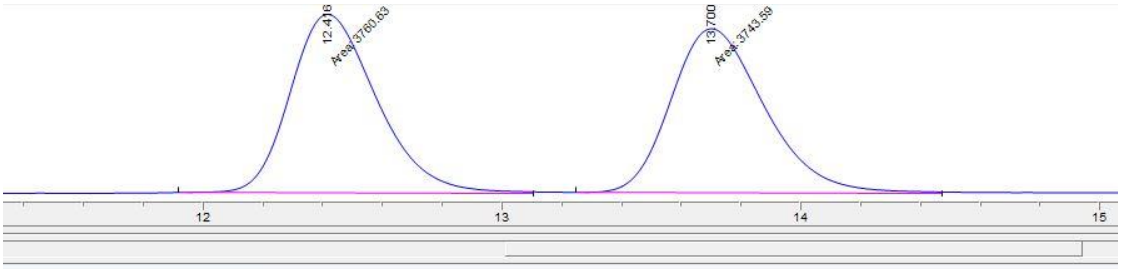

#	Time	Area	Height	Width	Area%	Symmetry
1	12.416	3760.6	186	0.3371	50.113	0.737
2	13.7	3743.6	170.9	0.3651	49.887	0.739

SFC analysis of the enantioenriched material, prepared using (R)-DM-SEGPHOS:

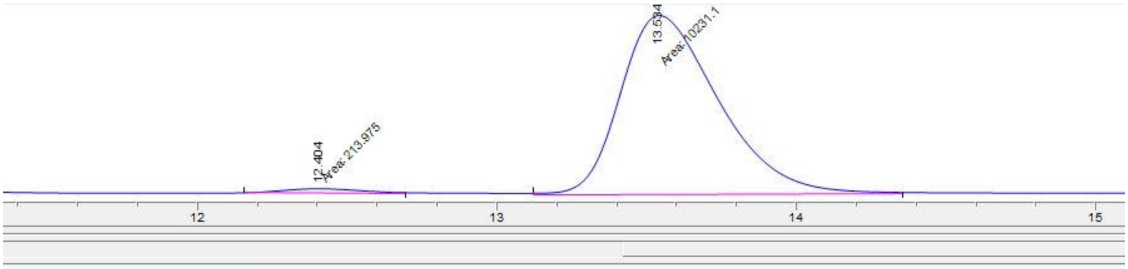

#	Time	Area	Height	Width	Area%	Symmetry
1	12.404	214	11.3	0.3166	2.049	0.733
2	13.534	10231.1	447.3	0.3812	97.951	0.64

(*S*)-3-(4-Hydroxyphenyl)-2-((4-hydroxyphenyl)amino)-3-methyl-1-(pyrrolidin-1-yl)butan-1-one (3id):

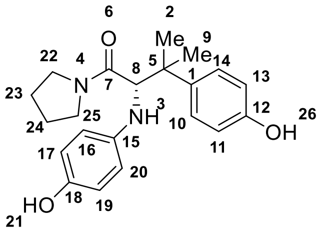

General procedure B: The reaction was carried out with substrate **1i** (22.0 mg, 0.10 mmol, 100 mol%) and 4-(prop-1-en-2-yl)phenol **2d** (53.6 mg, 0.40 mmol, 400 mol%). Purification of the residue by FCC (hexane/EtOAc 50:50) afforded the title compound (12.0 mg, 34%, >30:1 B:L, e.r. = 98.5:1.5) as a pale-yellow oil. ^{1}H NMR analysis of the crude material gave >30:1 B:L. $[\boldsymbol{\alpha}]_{\boldsymbol{D}}^{\mathbf{25}}$ = +5.3 (c = 1.0, $CHCl_3$); **IR (thin film)** ν_{max}/cm^{-1}: 3349 (br), 2923 (s), 2852 (s), 1611 (s), 1515 (s), 1355 (s), 1278 (s), 1127 (s), 745 (s), 698 (s); **^{1}H NMR** (500 MHz, CD_3OD) δ 7.32 (d, *J* = 9.0 Hz, 2H, $H^{10} + H^{14}$), 6.74 (d, *J* = 9.0 Hz, 2H, $H^{11} + H^{13}$), 6.61 (s, 4H, $H^{16} + H^{17} + H^{19} + H^{20}$), 4.02 (s, 1H, H^8), 3.23 – 3.18 (m, 1H, H^{22}), 3.11 – 3.02 (m, 2H, $H^{22} + H^{25}$), 2.52 – 2.48 (m, 1H, H^{25}), 1.64 – 1.52 (m, 6H, $H^2 + H^{23} + H^{24}$), 1.48 (s, 3H, H^9), 1.42 – 1.37 (m, 1H, H^{24}); **^{13}C NMR** (126 MHz, CD_3OD) δ 172.3 (C^7), 155.7 (C^{12}), 150.0 (C^{15}), 141.1 (C^{18}), 136.8 (C^1), 127.7 ($C^{10} + C^{14}$), 116.6 (ArC), 115.4 (ArC), 114.2 ($C^{11} + C^{13}$), 66.7 (C^8), 46.5 (C^{22}), 45.2

(C^{25}), 40.9 (C^5), 25.9 (C^2), 25.3 (C^{23}), 23.3 (C^{24}), 21.9 (C^9); **HRMS** (ESI): calculated for $C_{21}H_{27}N_2O_3$ $[M+H]^+$ requires *m/z* 355.2016, found *m/z* 355.2023; **Chiral SFC:** YMC Chiral ART Cellulose-SC column (25 cm), CO_2:*i*-PrOH 75:25, 3 mL/min, 229 bar, r.t. Retention times: 2.9 mins (major), 4.0 mins (minor), e.r. = 98.5:1.5.

SFC analysis of the racemate, prepared using rac-BINAP:

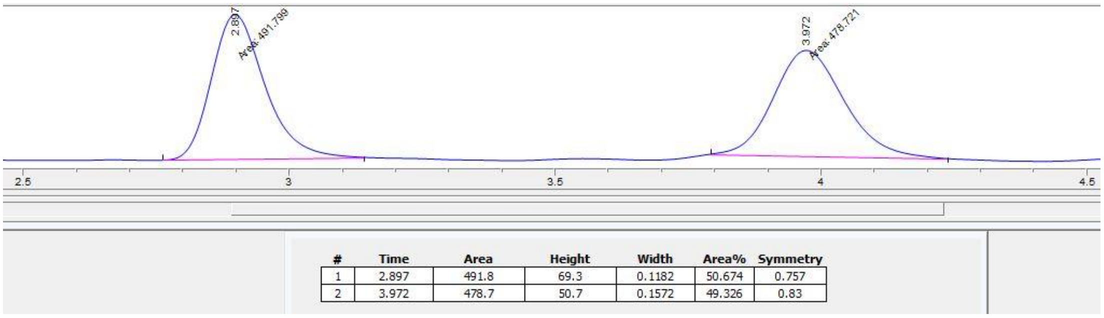

#	Time	Area	Height	Width	Area%	Symmetry
1	2.897	491.8	69.3	0.1182	50.674	0.757
2	3.972	478.7	50.7	0.1572	49.326	0.83

SFC analysis of the enantioenriched material, prepared using (R)-DM-SEGPHOS:

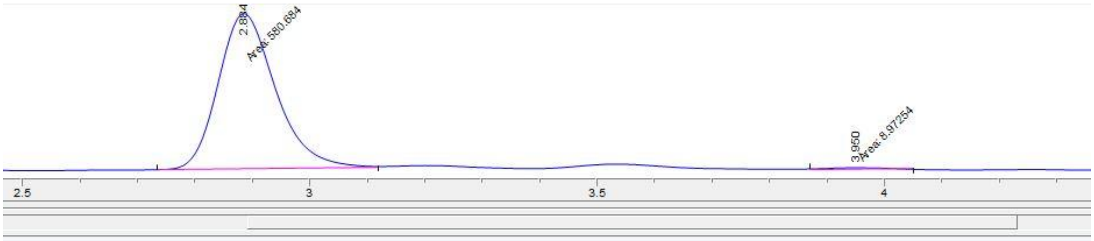

#	Time	Area	Height	Width	Area%	Symmetry
1	2.884	580.7	83.6	0.1158	98.478	0.786
2	3.95	9	1.2	0.1268	1.522	1.069

(*S*)-3-(4-Fluorophenyl)-2-((4-hydroxyphenyl)amino)-3-methyl-1-(pyrrolidin-1-yl)butan-1-one (3ie):

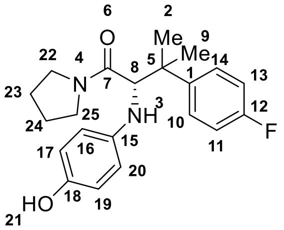

General procedure B: The reaction was carried out with substrate **1i** (22.0 mg, 0.10 mmol, 100 mol%) and 1-fluoro-4-(prop-1-en-2-yl)benzene **2e** (56.0 μL, 0.40 mmol, 400 mol%). Purification of the residue by FCC (hexane/EtOAc 50:50) afforded the title compound (21.0 mg, 59%, >30:1 B:L, e.r. = 98:2) as a colorless oil. ^{1}H NMR analysis of the crude material gave >30:1 B:L. $[\alpha]_D^{25}$ = -13.9 (c = 1.0, $CHCl_3$); **IR (thin film)** ν_{max}/cm^{-1}: 3351 (br), 2974 (s), 2878 (s), 1612 (s), 1510 (s), 1449 (s), 1227 (s), 1166 (s), 834 (s), 736 (s); **^{1}H NMR** (500 MHz, $CDCl_3$) δ 7.48 – 7.45 (m, 2H, H^{10} + H^{14}), 7.02 – 6.98 (m, 2H, H^{11} + H^{13}), 6.68 (d, *J* = 9.0 Hz,

2H, H[17] + H[19]), 6.54 (d, *J* = 9.0 Hz, 2H, H[16] + H[20]), 6.38 (br. s, 1H, H[21]), 4.19 (br. s, 1H, H[3]), 3.94 (s, 1H, H[8]), 3.34 – 3.29 (m, 1H, H[22]), 3.18 – 3.13 (m, 1H, H[22]), 3.05 – 3.00 (m, 1H, H[25]), 2.49 – 2.45 (m, 1H, H[25]), 1.64 – 1.57 (m, 5H, H[2] + H[23]), 1.56 – 1.50 (m, 4H, H[9] + H[24]), 1.46 – 1.39 (m, 1H, H[24]); **^{13}C NMR** (126 MHz, $CDCl_3$) δ 171.4 (C[7]), 161.5 (d, *J* = 243.8 Hz, C[12]), 149.3 (C[15]), 142.0 (d, *J* = 3.8 Hz, C[1]), 141.5 (C[18]), 128.6 (d, *J* = 7.5 Hz, C[10] + C[14]), 116.6 (ArC), 116.2 (ArC), 114.5 (d, *J* = 20.0 Hz, C[11] + C[13]), 66.5 (C[8]), 46.7 (C[22]), 45.5 (C[25]), 41.9 (C[5]), 26.5 (C[2]), 25.7 (C[23]), 23.8 (C[24]), 23.4 (C[9]); ^{19}F NMR (471 MHz, $CDCl_3$) δ -116.7; **HRMS** (ESl): calculated for $C_{21}H_{26}FN_2O_2$ $[M+H]^+$ requires *m/z* 357.1973, found *m/z* 357.1976; **Chiral SFC:** YMC Chiral ART Cellulose-SC column (25 cm), CO_2:*i*-PrOH 80:20, 2 mL/min, 178 bar, r.t. Retention times: 7.0 mins (minor), 7.3 mins (major), e.r. = 98:2.

SFC analysis of the racemate, prepared using rac-BINAP:

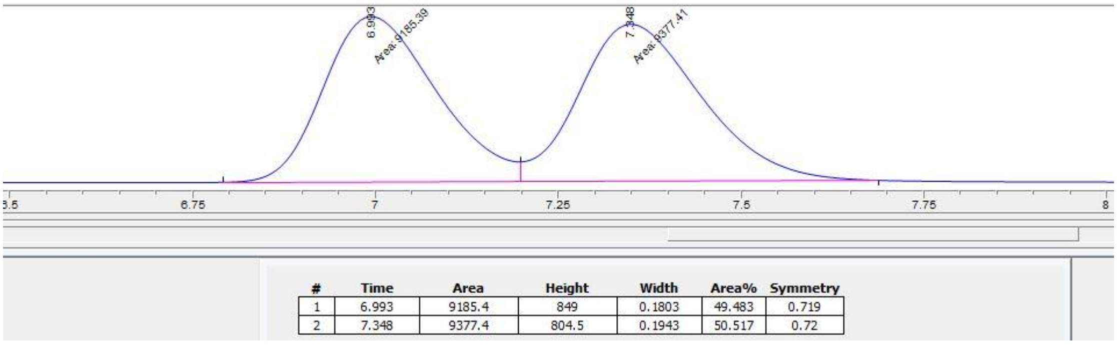

#	Time	Area	Height	Width	Area%	Symmetry
1	6.993	9185.4	849	0.1803	49.483	0.719
2	7.348	9377.4	804.5	0.1943	50.517	0.72

SFC analysis of the enantioenriched material, prepared using (R)-DM-SEGPHOS:

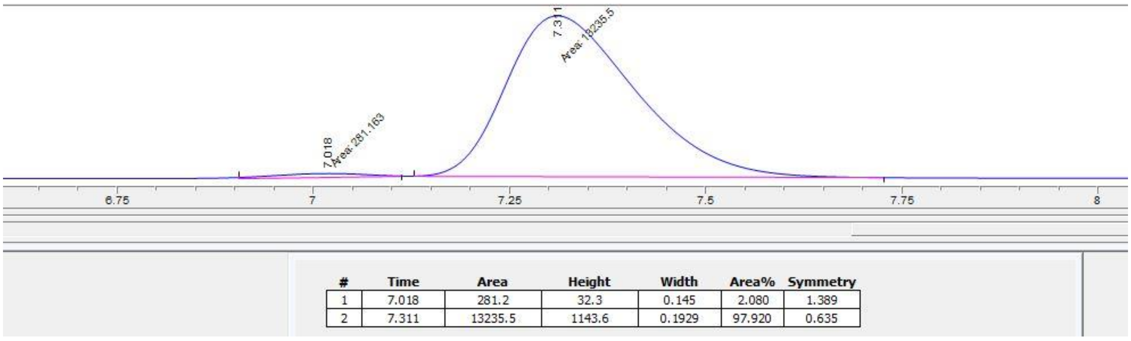

#	Time	Area	Height	Width	Area%	Symmetry
1	7.018	281.2	32.3	0.145	2.080	1.389
2	7.311	13235.5	1143.6	0.1929	97.920	0.635

(*S*)-3-(4-Chlorophenyl)-2-((4-hydroxyphenyl)amino)-3-methyl-1-(pyrrolidin-1-yl)butan-1-one (3if):

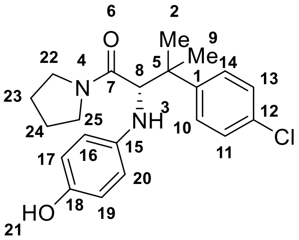

General procedure B: The reaction was carried out with substrate **1i** (22.0 mg, 0.10 mmol, 100 mol%) and 1-chloro-4-(prop-1-en-2-yl)benzene **2f** (57.0 µL, 0.40 mmol, 400 mol%). Purification of the residue by FCC (hexane/EtOAc 50:50) afforded the title compound (26.1 mg, 70%, >30:1 B:L, e.r. = 97:3) as a pale-yellow oil. ^{1}H NMR analysis of the crude material gave >30:1 B:L. $[\alpha]_D^{25}$ = -18.5 (c = 1.0, $CHCl_3$); **IR (thin film)** ν_{max}/cm^{-1}: 3239 (br), 2971 (s), 1612 (s), 1513 (s), 1448 (s), 1240 (s), 824 (s), 735 (s); **^{1}H NMR** (500 MHz, $CDCl_3$) δ 7.43 (d, *J* = 8.5 Hz, 2H, H^{10} + H^{14}), 7.29 (d, *J* = 8.5 Hz, 2H, H^{11} + H^{13}), 6.64 (d, *J* = 9.0 Hz, 2H, H^{17} + H^{19}), 6.52 (d, *J* = 9.0 Hz, 2H, H^{16} + H^{20}), 5.96 (br. s, 1H, H^{21}), 4.18 (br. s, 1H, H^3), 3.94 (s, 1H, H^8), 3.35 – 3.30 (m, 1H, H^{22}), 3.19 – 3.14 (m, 1H, H^{22}), 3.07 – 3.02 (m, 1H, H^{25}), 2.54 – 2.50 (m, 1H, H^{25}), 1.67 – 1.55 (m, 6H, H^2 + H^{23} + H^{24}), 1.52 (s, 3H, H^9), 1.47 – 1.41 (m, 1H, H^{24}); **^{13}C NMR** (126 MHz, $CDCl_3$) δ 171.2 (C^7), 149.2 (C^{15}), 144.8 (C^1), 141.5 (C^{18}), 132.4 (C^{12}), 128.5 (ArC), 127.9 (ArC), 116.6 (ArC), 116.2 (ArC), 66.3 (C^8), 46.7 (C^{22}), 45.5 (C^{25}), 42.0 (C^5), 26.2 (C^2), 25.7 (C^{23}), 23.8 (C^{24}), 23.3 (C^9); **HRMS** (ESl): calculated for $C_{21}H_{26}{}^{35}ClN_2O_2$ $[M+H]^+$ requires *m/z* 373.1677, found *m/z* 373.1684; **Chiral SFC:** YMC Chiral ART Cellulose-SC column (25 cm), CO_2:*i*-PrOH 75:25, 3 mL/min, 230 bar, r.t. Retention times: 3.9 mins (minor), 4.1 mins (major), e.r. = 97:3.

SFC analysis of the racemate, prepared using rac-BINAP:

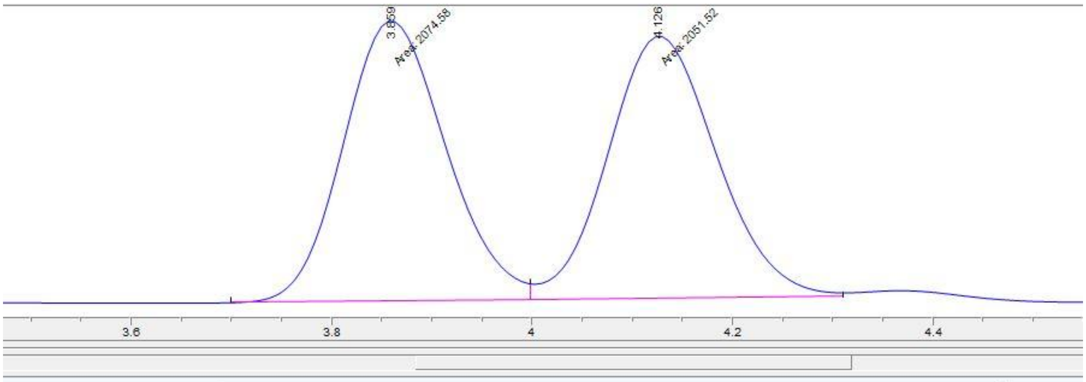

#	Time	Area	Height	Width	Area%	Symmetry
1	3.859	2074.6	291.2	0.1188	50.279	0.856
2	4.126	2051.5	272.3	0.1255	49.721	0.875

SFC analysis of the enantioenriched material, prepared using (R)-DM-SEGPHOS:

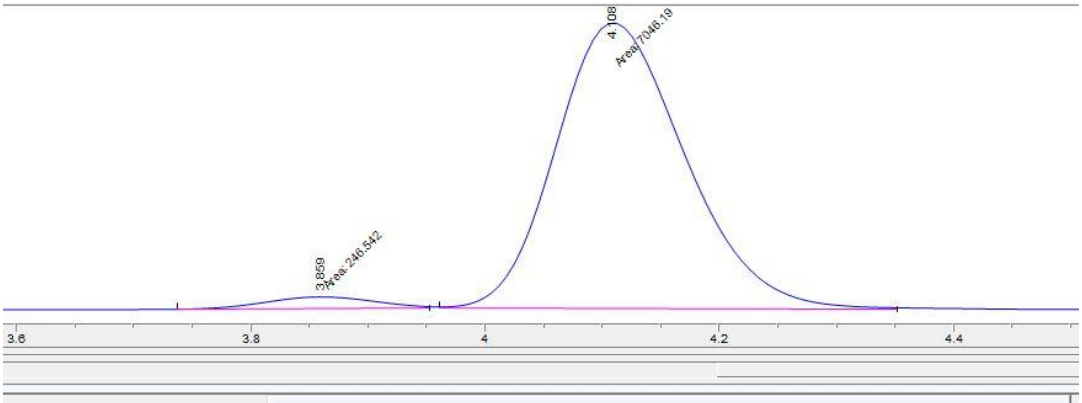

#	Time	Area	Height	Width	Area%	Symmetry
1	3.859	246.5	38	0.1082	3.381	1.012
2	4.108	7046.2	922.1	0.1274	96.619	0.781

(*S*)-3-(4-Bromophenyl)-2-((4-hydroxyphenyl)amino)-3-methyl-1-(pyrrolidin-1-yl)butan-1-one (3ig):

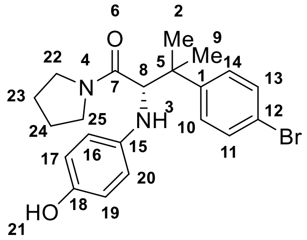

General procedure B: The reaction was carried out with substrate **1i** (22.0 mg, 0.10 mmol, 100 mol%) and 1-bromo-4-(prop-1-en-2-yl)benzene **2g** (58.0 µL, 0.40 mmol, 400 mol%). Purification of the residue by FCC (hexane/EtOAc 50:50) afforded the title compound (17.5 mg, 42%, >30:1 B:L, e.r. = 97:3) as a pale-yellow oil. ^{1}H NMR analysis of the crude material gave >30:1 B:L. $[\boldsymbol{\alpha}]_{\boldsymbol{D}}^{\mathbf{25}}$ = -8.4 (c = 1.0, $CHCl_3$); **IR (thin film)** ν_{max}/cm^{-1}: 3257 (br), 2971 (s), 1614 (s), 1514 (s), 1449 (s), 1241 (s), 1007 (s), 824 (s), 736 (s); **^{1}H NMR** (500 MHz, $CDCl_3$) δ 7.44 (d, *J* = 8.5 Hz, 2H, H^{11} + H^{13}), 7.38 (d, *J* = 8.5 Hz, 2H, H^{10} + H^{14}), 6.64 (d, *J* = 9.0 Hz, 2H, H^{17} + H^{19}), 6.53 (d, *J* = 9.0 Hz, 2H, H^{16} + H^{20}), 5.39 (br. s, 1H, H^{21}), 4.18 (br. s, 1H, H^3), 3.94 (s, 1H, H^8), 3.36 – 3.31 (m, 1H, H^{22}), 3.18 – 3.13 (m, 1H, H^{22}), 3.07 – 3.02 (m, 1H, H^{25}), 2.55 – 2.50 (m, 1H, H^{25}), 1.68 – 1.58 (m, 6H, H^2 + H^{23} + H^{24}), 1.52 (s, 3H, H^9), 1.48 – 1.43 (m, 1H, H^{24}); **^{13}C NMR** (126 MHz, $CDCl_3$) δ 171.1 (C^7), 148.9 (C^{15}), 145.4 (ArC), 141.7 (C^{18}), 130.9 (C^{11} + C^{13}), 128.9 (C^{10} + C^{14}), 120.5 (ArC), 116.5 (ArC), 116.2 (ArC), 66.1 (C^8), 46.7 (C^{22}), 45.5 (C^{25}), 42.1 (C^5), 26.1 (C^2), 25.8 (C^{23}), 23.9 (C^{24}), 23.2 (C^9); **HRMS** (ESI): calculated for $C_{21}H_{26}{}^{79}BrN_2O_2$ $[M+H]^+$ requires *m/z* 417.1172, found *m/z* 417.1177; **Chiral SFC:** YMC Chiral ART Cellulose-SC column (25 cm), CO_2:*i*-PrOH 80:20, 2 mL/min, 175 bar, r.t. Retention times: 10.5 mins (minor), 11.4 mins (major), e.r. = 97:3.

SFC analysis of the racemate, prepared using rac-BINAP:

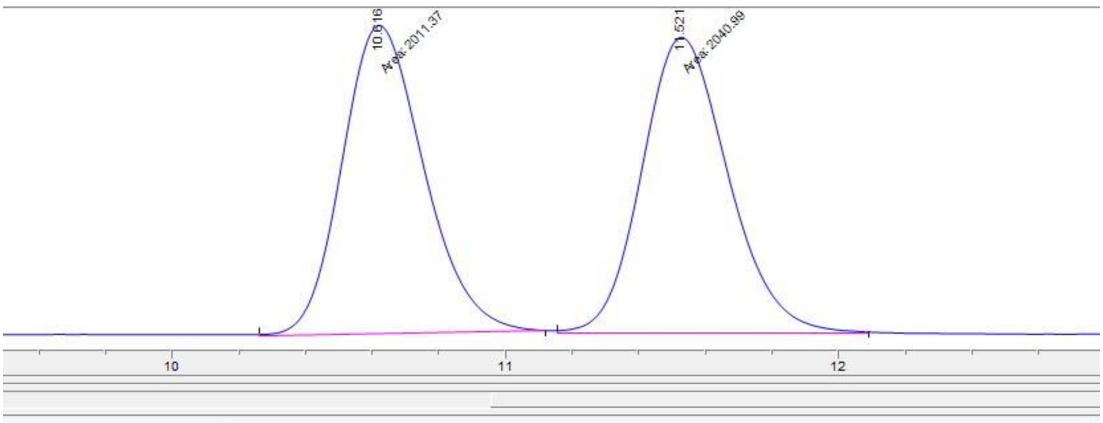

#	Time	Area	Height	Width	Area%	Symmetry
1	10.616	2011.4	117.7	0.2849	49.634	0.811
2	11.521	2041	112.9	0.3012	50.366	0.822

SFC analysis of the enantioenriched material, prepared using (R)-DM-SEGPHOS:

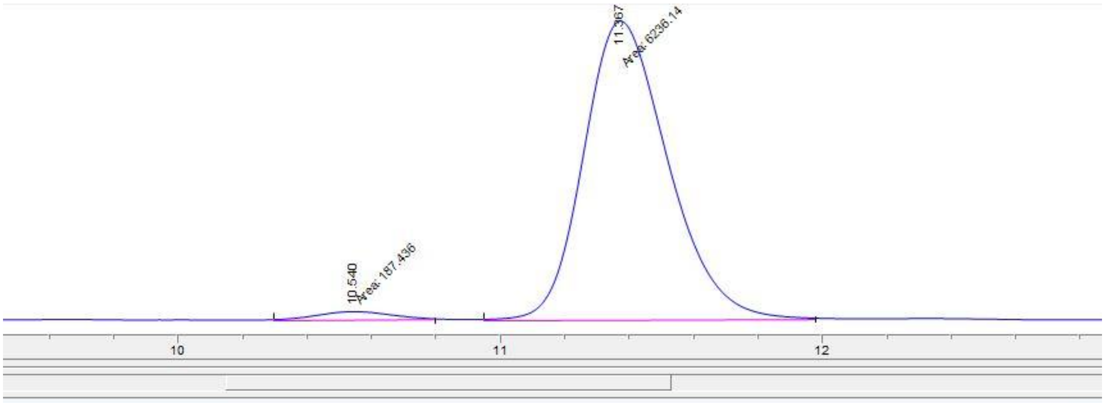

#	Time	Area	Height	Width	Area%	Symmetry
1	10.54	187.4	10.6	0.2952	2.918	0.988
2	11.367	6236.1	340.3	0.3054	97.082	0.759

(*S*)-3-([1,1'-Biphenyl]-4-yl)-2-((4-hydroxyphenyl)amino)-3-methyl-1-(pyrrolidin-1-yl)butan-1-one (3ih):

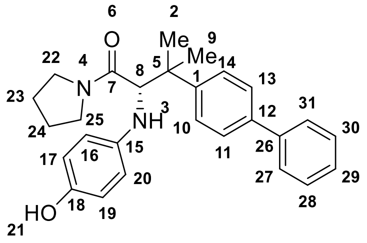

General procedure B: The reaction was carried out with substrate **1i** (22.0 mg, 0.10 mmol, 100 mol%) and 4-(prop-1-en-2-yl)-1,1'-biphenyl **2h** (77.6 mg, 0.40 mmol, 400 mol%). Purification of the residue by FCC (hexane/EtOAc 50:50) afforded the title compound (26.1 mg, 63%, >30:1 B:L, e.r. = 97:3) as a brown solid. ^{1}H NMR analysis of the crude material gave >30:1 B:L. **m.p.** = 135 – 137 °C (hexane/EtOAc); $[\alpha]_D^{25}$ = -11.6 (c = 1.0, $CHCl_3$); **IR (thin**

film) ν_{max}/cm^{-1}: 3238 (br), 2968 (s), 1612 (s), 1514 (s), 1448 (s), 1240 (s), 767 (s), 735 (s), 698 (s); **^{1}H NMR** (500 MHz, $CDCl_3$) δ 7.60 – 7.55 (m, 6H, H^{10} + H^{11} + H^{13} + H^{14} + H^{27} + H^{31}), δ 7.48 – 7.45 (m, 2H, H^{28} + H^{30}), δ 7.39 – 7.36 (m, 1H, H^{29}), 6.65 (d, J = 9.0 Hz, 2H, H^{17} + H^{19}), 6.56 (d, J = 9.0 Hz, 2H, H^{16} + H^{20}), 5.94 (br. s, 1H, H^{21}), 4.26 (br. s, 1H, H^{3}), 4.00 (s, 1H, H^{8}), 3.35 – 3.30 (m, 1H, H^{22}), 3.21 – 3.15 (m, 1H, H^{22}), 3.03 – 2.98 (m, 1H, H^{25}), 2.48 – 2.43 (m, 1H, H^{25}), 1.66 (s, 3H, H^{2}), 1.61 – 1.53 (m, 5H, H^{9} + H^{23}), 1.51 – 1.44 (m, 1H, H^{24}), 1.35 – 1.29 (m, 1H, H^{24}); **^{13}C NMR** (126 MHz, $CDCl_3$) δ 171.4 (C^{7}), 149.1 (C^{15}), 145.4 (ArC), 141.7 (C^{18}), 140.7 (C^{26}), 139.4 (ArC), 128.9 (ArC), 127.4 (ArC), 127.3 (ArC), 127.0 (ArC), 126.5 (ArC), 116.5 (ArC), 116.2 (ArC), 66.4 (C^{8}), 46.6 (C^{22}), 45.5 (C^{25}), 42.2 (C^{5}), 26.4 (C^{2}), 25.7 (C^{23}), 23.8 (C^{24}), 22.9 (C^{9}); **HRMS** (ESl): calculated for $C_{27}H_{31}N_2O_2$ $[M+H]^+$ requires *m/z* 415.2380, found *m/z* 415.2384; **Chiral SFC:** YMC Chiral ART Cellulose-SC column (25 cm), CO_2:*i*-PrOH 75:25, 3 mL/min, 228 bar, r.t. Retention times: 7.0 mins (minor), 7.7 mins (major), e.r. = 97:3.

SFC analysis of the racemate, prepared using rac-BINAP:

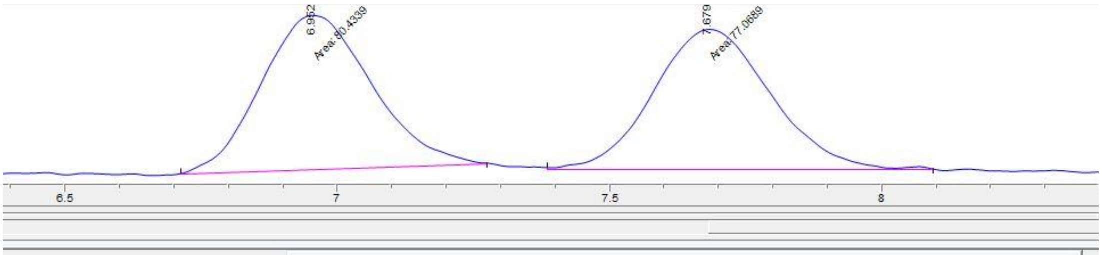

#	Time	Area	Height	Width	Area%	Symmetry
1	6.952	80.4	5.7	0.2363	51.068	0.856
2	7.679	77.1	5.1	0.2503	48.932	0.89

SFC analysis of the enantioenriched material, prepared using (R)-DM-SEGPHOS:

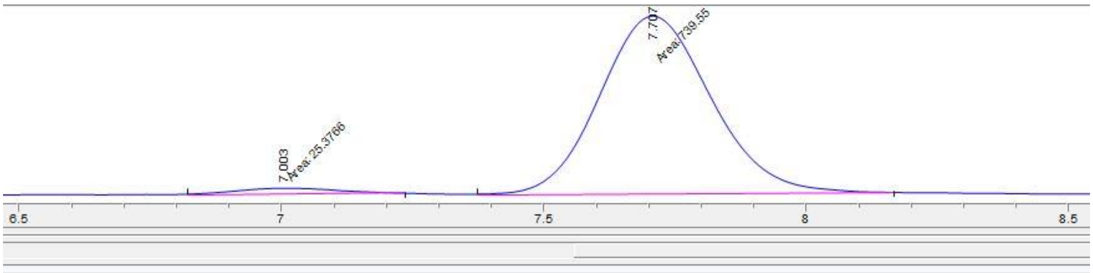

#	Time	Area	Height	Width	Area%	Symmetry
1	7.003	25.4	1.8	0.2375	3.318	0.901
2	7.707	739.6	49.7	0.2481	96.682	0.889

(*S*)-2-((4-Hydroxyphenyl)amino)-3-methyl-1-(pyrrolidin-1-yl)-3-(4-(4,4,5,5-tetramethyl-1,3,2-dioxaborolan-2-yl)phenyl)butan-1-one (3ii):

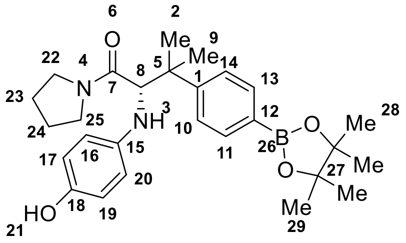

General procedure B: The reaction was carried out with substrate **1i** (22.0 mg, 0.10 mmol, 100 mol%) and 4,4,5,5-tetramethyl-2-(4-(prop-1-en-2-yl)phenyl)-1,3,2-dioxaborolane **2i** (97.7 mg, 0.40 mmol, 400 mol%). Purification of the residue by FCC (hexane/EtOAc 50:50) afforded the title compound (42.7 mg, 92%, >30:1 B:L, e.r. = 97:3) as a pale-yellow oil. ^{1}H NMR analysis of the crude material gave >30:1 B:L. $[\boldsymbol{\alpha}]_{\boldsymbol{D}}^{\mathbf{25}}$ = -7.4 (c = 1.0, $CHCl_3$); **IR (thin film)** ν_{max}/cm^{-1}: 3261 (br), 2973 (s), 1614 (s), 1515 (s), 1449 (s), 1241 (s), 825 (s), 702 (s); **^{1}H NMR** (500 MHz, $CDCl_3$) δ 7.76 (d, *J* = 8.0 Hz, 2H, H^{11} + H^{13}), 7.51 (d, *J* = 8.0 Hz, 2H, H^{10} + H^{14}), 6.66 (d, *J* = 8.0 Hz, 2H, H^{17} + H^{19}), 6.56 (d, *J* = 8.0 Hz, 2H, H^{16} + H^{20}), 5.29 (br. s, 1H, H^{21}), 4.27 (br. s, 1H, H^3), 3.97 (s, 1H, H^8), 3.34 – 3.29 (m, 1H, H^{22}), 3.17 – 3.12 (m, 1H, H^{22}), 2.98 – 2.94 (m, 1H, H^{25}), 2.37 – 2.33 (m, 1H, H^{25}), 1.63 – 1.47 (m, 9H, H^2 + H^9 + H^{23} + H^{24}), 1.36 (s, 6H, H^{28}), 1.29 (s, 7H, H^{24} + H^{29}); **^{13}C NMR** (126 MHz, $CDCl_3$) δ 171.1 (C^7), 149.7 (C^1), 148.7 (C^{18}), 142.0 (C^{15}), 134.5 (C^{11} + C^{13}), 127.9 (C^{12}), 126.3 (C^{10} + C^{14}), 116.3 (ArC), 116.1 (ArC), 83.8 (C^{27}), 66.1 (C^8), 46.5 (C^{22}), 45.4 (C^{25}), 42.6 (C^5), 26.2 (C^2), 25.8 (C^{23}), 24.9 – 23.8 (m, C^{24} + C^{28} + C^{29}), 23.0 (C^9); **HRMS** (ESI): calculated for $C_{27}H_{38}BN_2O_4$ $[M+H]^+$ requires *m/z* 465.2919, found *m/z* 465.2925; **Chiral SFC:** YMC Chiral ART Cellulose-SC column (25 cm), CO_2:*i*-PrOH 75:25, 4 mL/min, 270 bar, r.t. Retention times: 2.4 mins (major), 3.7 mins (minor), e.r. = 97:3.

SFC analysis of the racemate, prepared using rac-BINAP:

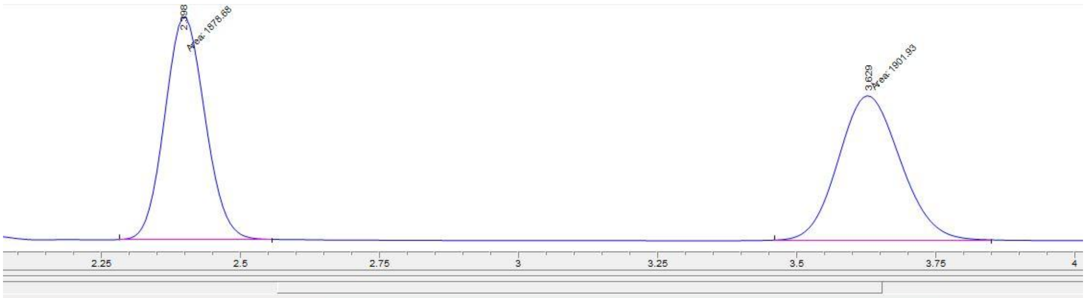

#	Time	Area	Height	Width	Area%	Symmetry
1	2.398	1878.7	370.1	0.0846	49.693	0.903
2	3.629	1901.9	240.4	0.1319	50.307	0.871

SFC analysis of the enantioenriched material, prepared using (R)-DM-SEGPHOS:

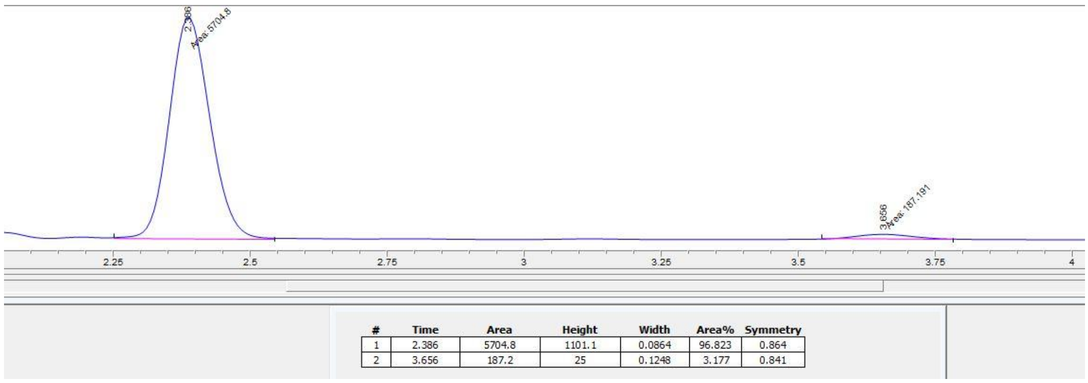

#	Time	Area	Height	Width	Area%	Symmetry
1	2.386	5704.8	1101.1	0.0864	96.823	0.864
2	3.656	187.2	25	0.1248	3.177	0.841

(*S*)-2-((4-Hydroxyphenyl)amino)-3-methyl-1-(pyrrolidin-1-yl)-3-(*m*-tolyl)butan-1-one (3ij):

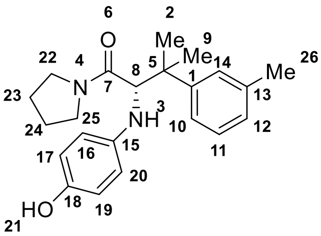

General procedure B: The reaction was carried out with substrate **1i** (22.0 mg, 0.10 mmol, 100 mol%) and 1-methyl-3-(prop-1-en-2-yl)benzene **2j** (58.0 µL, 0.40 mmol, 400 mol%). Purification of the residue by FCC (hexane/EtOAc 50:50) afforded the title compound (25.7 mg, 73%, >30:1 B:L, e.r. = 97:3) as a colorless oil. ^{1}H NMR analysis of the crude material gave >30:1 B:L. $[\alpha]_D^{25}$ = -21.3 (c = 1.0, $CHCl_3$); **IR (thin film)** ν_{max}/cm^{-1}: 3263 (br), 2970 (s), 1612 (s), 1514 (s), 1449 (s), 1241 (s), 824 (s), 705 (s); **^{1}H NMR** (500 MHz, $CDCl_3$) δ 7.32 – 7.30 (m, 2H, ArH), 7.23 – 7.20 (m, 1H, H^{11}), 7.05 (d, *J* = 7.5 Hz, 1H, ArH), 6.64 (d, *J* = 9.0 Hz, 2H, H^{17} + H^{19}), 6.53 (d, *J* = 9.0 Hz, 2H, H^{16} + H^{20}), 6.16 (br. s, 1H, H^{21}), 4.25 (br. s, 1H, H^3), 3.95 (s, 1H, H^8), 3.32 – 3.27 (m, 1H, H^{22}), 3.18 – 3.13 (m, 1H, H^{22}), 2.99 – 2.95 (m, 1H, H^{25}), 2.36 (s, 3H, H^{26}), 2.30 – 2.25 (m, 1H, H^{25}), 1.63 – 1.55 (m, 5H, H^2 + H^{23}), 1.53 (s, 3H, H^9), 1.50 – 1.44 (m, 1H, H^{24}), 1.35 – 1.31 (m, 1H, H^{24}); **^{13}C NMR** (126 MHz, $CDCl_3$) δ 171.5 (C^7), 149.1 (C^{15}), 146.2 (C^1), 141.7 (C^{18}), 137.4 (C^{13}), 127.9 (ArC), 127.7 (ArC), 127.2 (ArC), 123.9 (ArC), 116.4 (ArC), 116.2 (ArC), 66.4 (C^8), 46.4 (C^{22}), 45.5 (C^{25}), 42.2 (C^5), 26.5 (C^2), 25.8 (C^{23}), 23.8 (C^{24}), 22.8 (C^9), 21.6 (C^{26}); **HRMS** (ESI): calculated for $C_{22}H_{29}N_2O_2$ $[M+H]^+$ requires *m/z* 353.2224, found *m/z* 353.2229; **Chiral SFC:** YMC Chiral ART Cellulose-SC column (25 cm), CO_2:*i*-PrOH 75:25, 3 mL/min, 228 bar, r.t. Retention times: 4.3 mins (major), 7.1 mins (minor), e.r. = 97:3.

SFC analysis of the racemate, prepared using rac-BINAP:

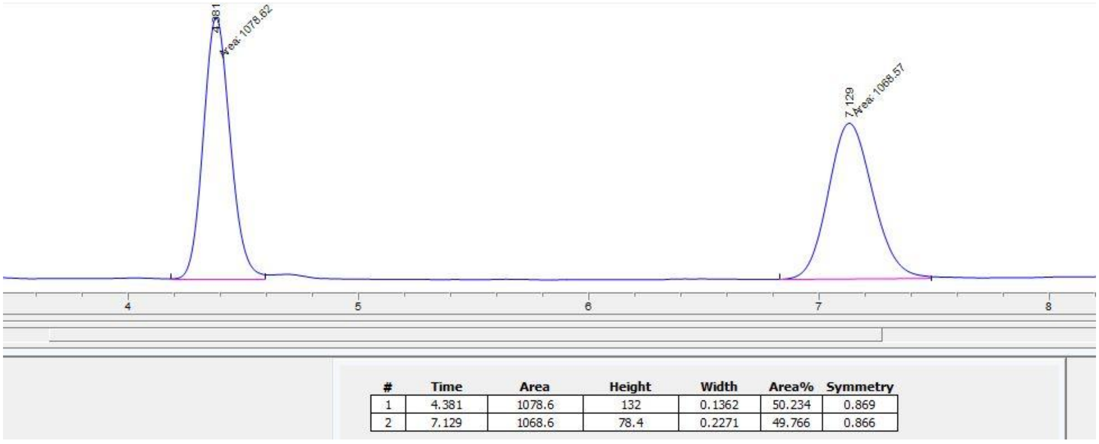

#	Time	Area	Height	Width	Area%	Symmetry
1	4.381	1078.6	132	0.1362	50.234	0.869
2	7.129	1068.6	78.4	0.2271	49.766	0.866

SFC analysis of the enantioenriched material, prepared using (R)-DM-SEGPHOS:

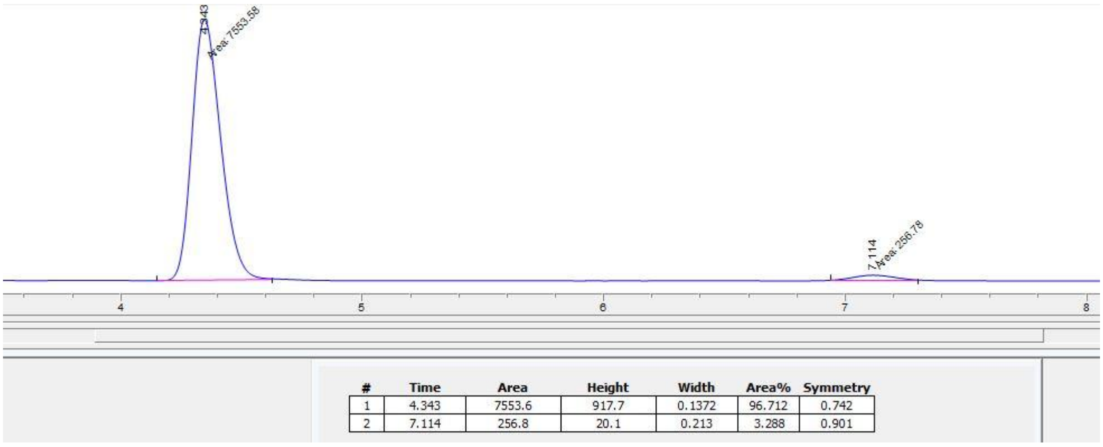

#	Time	Area	Height	Width	Area%	Symmetry
1	4.343	7553.6	917.7	0.1372	96.712	0.742
2	7.114	256.8	20.1	0.213	3.288	0.901

(*S*)-3-(3-Chlorophenyl)-2-((4-hydroxyphenyl)amino)-3-methyl-1-(pyrrolidin-1-yl)butan-1-one (3ik):

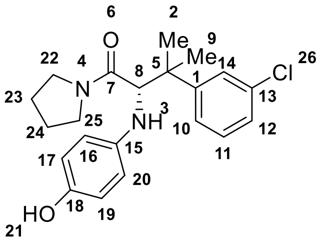

General procedure B: The reaction was carried out with substrate **1i** (22.0 mg, 0.10 mmol, 100 mol%) and 1-chloro-3-(prop-1-en-2-yl)benzene **2k** (60.0 µL, 0.40 mmol, 400 mol%). Purification of the residue by FCC (hexane/EtOAc 50:50) afforded the title compound (26.4 mg, 71%, >30:1 B:L, e.r. = 97:3) as a pale-yellow solid. ^{1}H NMR analysis of the crude material gave >30:1 B:L. **m.p.** = 138 – 140 °C (hexane/EtOAc); $[\alpha]_D^{25}$ = -18.5 (c = 1.0, $CHCl_3$); **IR (thin film)** ν_{max}/cm^{-1}: 3264 (br), 2969 (s), 1613 (s), 1513 (s), 1449 (s), 1239 (s), 735 (s), 695 (s); **^{1}H NMR** (500 MHz, $CDCl_3$) δ 7.48 – 7.47 (m, 1H, H^{14}), 7.41 – 7.38 (m, 1H, ArH), 7.27 –

7.22 (m, 2H, ArH), 6.64 (d, J = 9.0 Hz, 2H, H^{17} + H^{19}), 6.53 (d, J = 9.0 Hz, 2H, H^{16} + H^{20}), 6.03 (br. s, 1H, H^{21}), 4.22 (br. s, 1H, H^{3}), 3.94 (s, 1H, H^{8}), 3.34 – 3.29 (m, 1H, H^{22}), 3.23 – 3.18 (m, 1H, H^{22}), 3.06 – 3.01 (m, 1H, H^{25}), 2.43 – 2.38 (m, 1H, H^{25}), 1.67 – 1.54 (m, 6H, H^{2} + H^{23} + H^{24}), 1.52 (s, 3H, H^{9}), 1.46 – 1.39 (m, 1H, H^{24}); **^{13}C NMR** (126 MHz, $CDCl_3$) δ 171.2 (C^{7}), 149.2 (C^{15}), 148.6 (C^{1}), 141.5 (C^{18}), 133.9 (C^{14}), 129.2 (ArC), 127.4 (ArC), 126.6 (ArC), 125.1 (ArC), 116.6 (ArC), 116.2 (ArC), 66.3 (C^{8}), 46.7 (C^{22}), 45.6 (C^{25}), 42.5 (C^{5}), 26.2 (C^{2}), 25.8 (C^{23}), 23.8 (C^{24}), 22.9 (C^{9}); **HRMS** (ESI): calculated for $C_{21}H_{26}{}^{35}ClN_2O_2$ $[M+H]^+$ requires *m/z* 373.1677, found *m/z* 373.1686; **Chiral SFC:** YMC Chiral ART Cellulose-SC column (25 cm), CO_2:*i*-PrOH 75:25, 3 mL/min, 228 bar, r.t. Retention times: 3.7 mins (major), 4.1 mins (minor), e.r. = 97:3.

SFC analysis of the racemate, prepared using rac-BINAP:

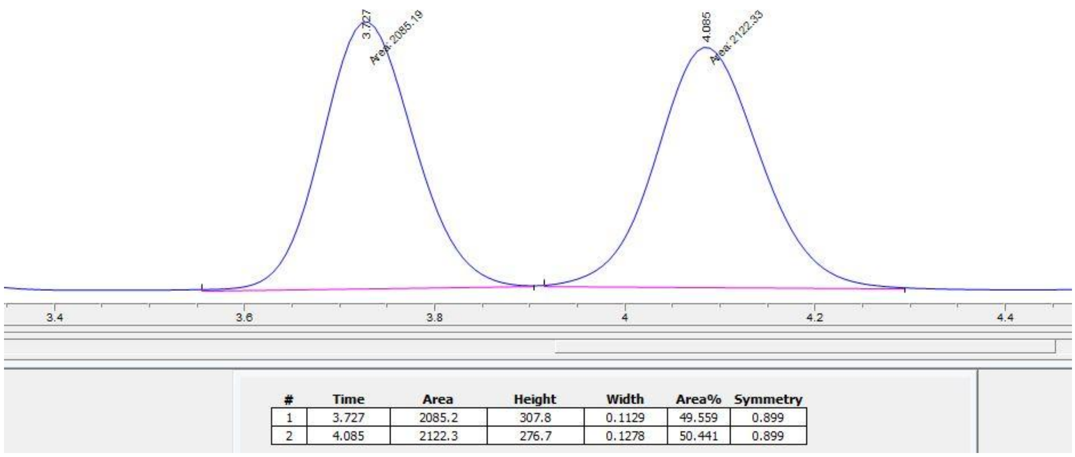

#	Time	Area	Height	Width	Area%	Symmetry
1	3.727	2085.2	307.8	0.1129	49.559	0.899
2	4.085	2122.3	276.7	0.1278	50.441	0.899

SFC analysis of the enantioenriched material, prepared using (R)-DM-SEGPHOS:

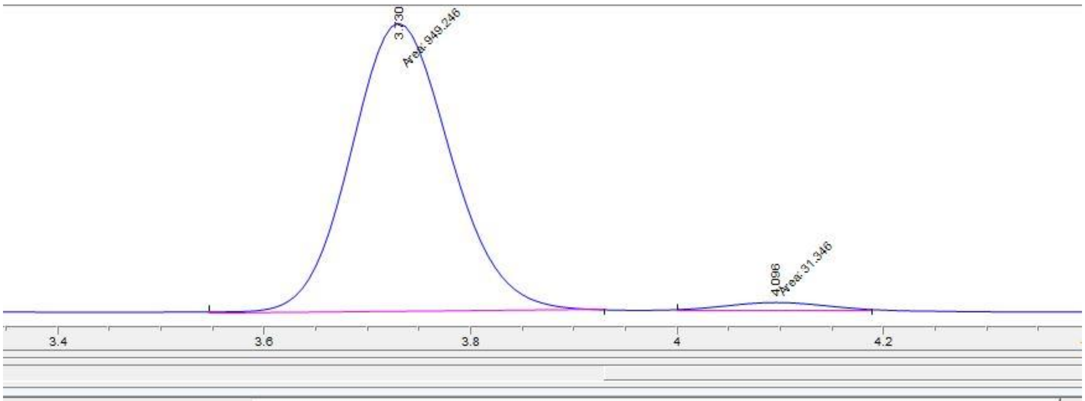

#	Time	Area	Height	Width	Area%	Symmetry
1	3.73	949.2	140.1	0.1129	96.803	0.9
2	4.096	31.3	4.4	0.1196	3.197	0.963

(*S*)-3-(2-Fluorophenyl)-2-((4-hydroxyphenyl)amino)-3-methyl-1-(pyrrolidin-1-yl)butan-1-one (3il):

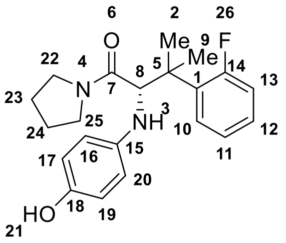

General procedure B: The reaction was carried out with substrate **1i** (22.0 mg, 0.10 mmol, 100 mol%) and 1-fluoro-2-(prop-1-en-2-yl)benzene **2l** (140 µL, 1.00 mmol, 1000 mol%), $[Ir(cod)_2]BARF$ (12.7 mg, 10.0 µmol, 10 mol%) and (*R*)-DM-SEGPHOS (7.23 mg, 10.0 µmol, 10 mol%). Purification of the residue by FCC (hexane/EtOAc 50:50) afforded the title compound (23.5 mg, 66%, >30:1 B:L, e.r. = 98:2) as a pale-yellow oil. ^{1}H NMR analysis of the crude material gave >30:1 B:L. $[\alpha]_D^{25}$ = -12.4 (c = 1.0, $CHCl_3$); **IR (thin film)** ν_{max}/cm^{-1}: 3280 (br), 2924 (s), 1613 (s), 1514 (s), 1445 (s), 1240 (s), 824 (s), 761 (s), 735 (s); **^{1}H NMR** (500 MHz, $CDCl_3$) δ 7.38 – 7.34 (m, 1H, H^{10}), 7.25 – 7.20 (m, 1H, H^{12}), 7.10 – 7.04 (m, 2H, H^{11} + H^{13}), 6.64 (d, *J* = 8.0 Hz, 2H, H^{17} + H^{19}), 6.51 (d, *J* = 8.0 Hz, 2H, H^{16} + H^{20}), 5.96 (br. s, 1H, H^{21}), 4.67 (s, 1H, H^8), 4.21 (br. s, 1H, H^3), 3.40 – 3.35 (m, 1H, H^{22}), 3.33 – 3.28 (m, 1H, H^{22}), 3.26 – 3.18 (m, 2H, H^{25}), 1.76 – 1.58 (m, 7H, H^2 + H^{23} + H^{24}), 1.53 (s, 3H, H^9); **^{13}C NMR** (126 MHz, $CDCl_3$) δ 171.5 (C^7), 161.8 (d, *J* = 243.8 Hz, C^{14}), 149.1 (C^{15}), 141.7 (C^{18}), 133.6 (d, *J* = 10.0 Hz, C^1), 129.4 (d, *J* = 5.0 Hz, C^{10}), 128.4 (d, *J* = 8.8 Hz, C^{12}), 124.3 (d, *J* = 3.8 Hz, C^{11}), 116.6 (ArC), 116.1 (ArC), 116.0 (d, *J* = 21.3 Hz, C^{13}), 66.0 (C^8), 47.0 (C^{22}), 45.7 (C^{25}), 42.1 (C^5), 25.9 (C^{23}), 24.4 (d, *J* = 3.8 Hz, C^2), 23.9 (C^{24}), 23.5 (d, *J* = 2.5 Hz, C^9); ^{19}F NMR (471 MHz, $CDCl_3$) δ -109.0; **HRMS** (ESl): calculated for $C_{21}H_{26}FN_2O_2$ $[M+H]^+$ requires *m/z* 357.1973, found *m/z* 357.1978; **Chiral SFC:** YMC Chiral ART Cellulose-SC column (25 cm), CO_2:*i*-PrOH 80:20, 1 mL/min, 148 bar, r.t. Retention times: 16.7 mins (minor), 17.0 mins (major), e.r. = 98:2.

SFC analysis of the racemate, prepared using rac-BINAP:

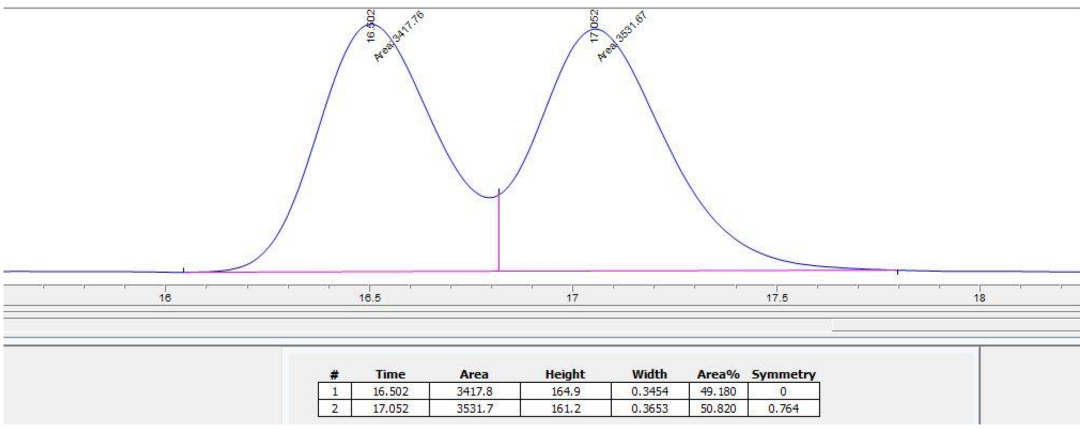

#	Time	Area	Height	Width	Area%	Symmetry
1	16.502	3417.8	164.9	0.3454	49.180	0
2	17.052	3531.7	161.2	0.3653	50.820	0.764

SFC analysis of the enantioenriched material, prepared using (R)-DM-SEGPHOS:

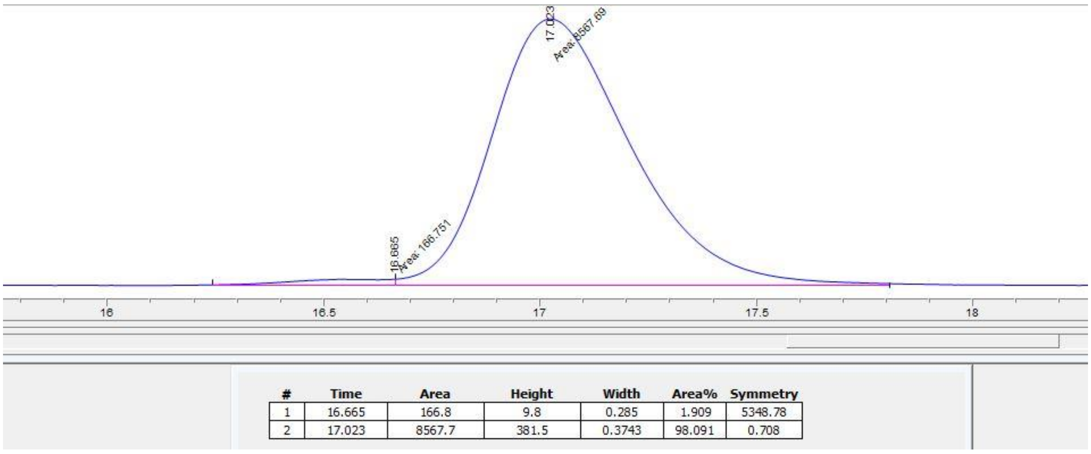

#	Time	Area	Height	Width	Area%	Symmetry
1	16.665	166.8	9.8	0.285	1.909	5348.78
2	17.023	8567.7	381.5	0.3743	98.091	0.708

(*S*)-2-((4-Hydroxyphenyl)amino)-3-methyl-3-(naphthalen-2-yl)-1-(pyrrolidin-1-yl)butan-1-one (3im):

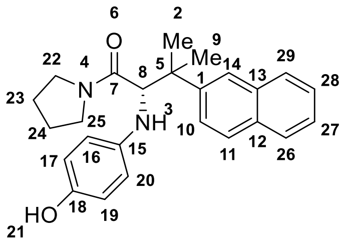

General procedure B: The reaction was carried out with substrate **1i** (22.0 mg, 0.10 mmol, 100 mol%) and 2-(prop-1-en-2-yl)naphthalene **2m** (67.2 mg, 0.40 mmol, 400 mol%). Purification of the residue by FCC (hexane/EtOAc 60:40) afforded the title compound (32.2 mg, 83%, >30:1 B:L, e.r. = 97:3) as a colorless oil. ^{1}H NMR analysis of the crude material gave >30:1 B:L. $[\boldsymbol{\alpha}]_{\boldsymbol{D}}^{\mathbf{25}}$ = +1.1 (c = 1.0, $CHCl_3$); **IR (thin film)** ν_{max}/cm^{-1}: 3260 (br), 2973 (s), 1611 (s), 1513 (s), 1447 (s), 1240 (s), 819 (s), 734 (s); **^{1}H NMR** (500 MHz, $CDCl_3$) δ 7.94 (d, *J* = 2.0 Hz, 1H, H^{14}), 7.85 – 7.78 (m, 3H, ArH), 7.67 – 7.65 (m, 1H, ArH), 7.50 – 7.45 (m, 2H, ArH), 6.69 (d, *J* = 8.5 Hz, 2H, H^{17} + H^{19}), 6.58 (d, *J* = 8.5 Hz, 2H, H^{16} + H^{20}), 6.51 (br. s, 1H,

H[21]), 4.30 (br. s, 1H, H[3]), 4.07 (s, 1H, H[8]), 3.29 – 3.24 (m, 1H, H[22]), 3.14 – 3.09 (m, 1H, H[22]), 2.94 – 2.90 (m, 1H, H[25]), 2.26 – 2.20 (m, 1H, H[25]), 1.74 (s, 3H, H[2]), 1.66 (s, 3H, H[9]), 1.49 – 1.35 (m, 2H, H[23]), 1.27 – 1.19 (m, 1H, H[24]), 1.00 – 0.92 (m, 1H, H[24]); **^{13}C NMR** (126 MHz, $CDCl_3$) δ 171.4 (C[7]), 149.4 (C[15]), 143.6 (C[1]), 141.4 (C[18]), 133.1 (C[13]), 132.0 (C[12]), 128.1 (ArC), 127.3 (ArC), 127.2 (ArC), 126.1 (ArC), 125.8 (ArC), 125.6 (ArC), 125.4 (ArC), 116.6 (ArC), 116.2 (ArC), 66.5 (C[8]), 46.5 (C[22]), 45.6 (C[25]), 42.5 (C[5]), 26.5 (C[2]), 25.5 (C[23]), 23.6 (C[24]), 23.2 (C[9]); **HRMS** (ESl): calculated for $C_{25}H_{29}N_2O_2$ $[M+H]^+$ requires *m/z* 389.2224, found *m/z* 389.2228; **Chiral SFC:** YMC Chiral ART Cellulose-SC column (25 cm), CO_2:*i*-PrOH 75:25, 3 mL/min, 228 bar, r.t. Retention times: 6.2 mins (major), 7.1 mins (minor), e.r. = 97:3.

SFC analysis of the racemate, prepared using rac-BINAP:

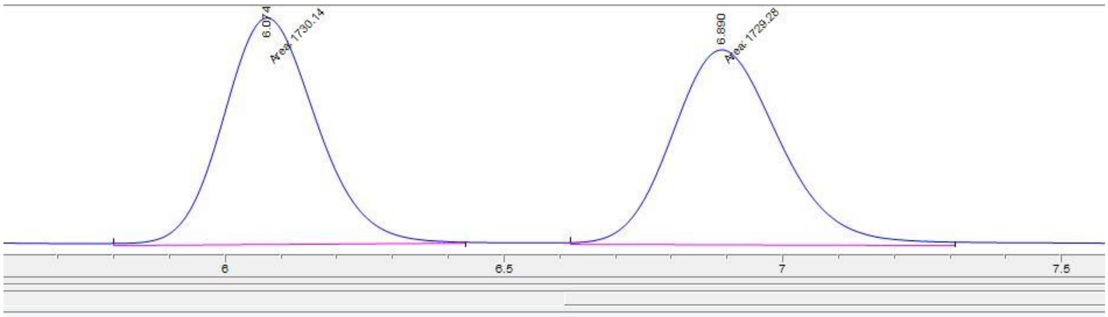

#	Time	Area	Height	Width	Area%	Symmetry
1	6.074	1730.1	146.7	0.1966	50.012	0.872
2	6.89	1729.3	126.3	0.2283	49.988	0.827

SFC analysis of the enantioenriched material, prepared using (R)-DM-SEGPHOS:

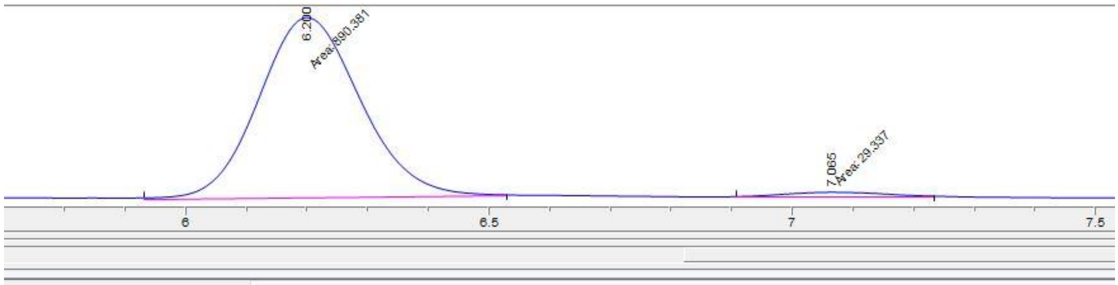

#	Time	Area	Height	Width	Area%	Symmetry
1	6.2	890.4	75.7	0.1961	96.810	0.891
2	7.065	29.3	2.3	0.2149	3.190	0.846

(*S*)-3-(Benzofuran-5-yl)-2-((4-hydroxyphenyl)amino)-3-methyl-1-(pyrrolidin-1-yl)butan-1-one (3in):

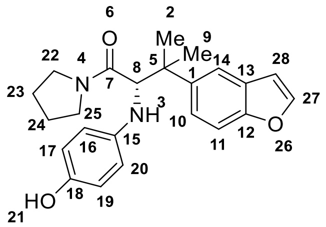

General procedure B: The reaction was carried out with substrate **1i** (22.0 mg, 0.10 mmol, 100 mol%) and 5-(prop-1-en-2-yl)benzofuran **2n** (63.2 mg, 0.40 mmol, 400 mol%). Purification of the residue by FCC (hexane/EtOAc 50:50) afforded the title compound (23.8 mg, 63%, >30:1 B:L, e.r. = 98:2) as a brown solid. ^{1}H NMR analysis of the crude material gave >30:1 B:L. **m.p.** = 154 – 156 °C (hexane/EtOAc); $[\boldsymbol{\alpha}]_D^{25}$ = -5.6 (c = 1.0, $CHCl_3$); **IR (thin film)** ν_{max}/cm^{-1}: 3237 (br), 2973 (s), 1611 (s), 1513 (s), 1450 (s), 1240 (s), 818 (s), 734 (s); **^{1}H NMR** (500 MHz, $CDCl_3$) δ 7.73 (d, *J* = 2.0 Hz, 1H, H^{14}), 7.62 (d, *J* = 2.5 Hz, 1H, H^{27}), 7.49 – 7.43 (m, 2H, H^{10} + H^{11}), 6.76 (d, *J* = 2.5 Hz, 1H, H^{28}), 6.63 (d, *J* = 8.5 Hz, 2H, H^{17} + H^{19}), 6.54 (d, *J* = 8.5 Hz, 2H, H^{16} + H^{20}), 5.88 (br. s, 1H, H^{21}), 4.26 (br. s, 1H, H^3), 4.00 (s, 1H, H^8), 3.31 – 3.26 (m, 1H, H^{22}), 3.15 – 3.10 (m, 1H, H^{22}), 2.97 – 2.93 (m, 1H, H^{25}), 2.26 – 2.20 (m, 1H, H^{25}), 1.68 (s, 3H, H^2), 1.60 (s, 3H, H^9), 1.55 – 1.46 (m, 2H, H^{23}), 1.38 – 1.31 (m, 1H, H^{24}), 1.16 – 1.10 (m, 1H, H^{24}); **^{13}C NMR** (126 MHz, $CDCl_3$) δ 171.5 (C^7), 153.6 (C^{12}), 149.0 (C^{15}), 145.4 (C^{27}), 141.8 (C^{18}), 140.9 (C^1), 127.1 (C^{13}), 123.4 (C^{10}), 119.5 (C^{14}), 116.4 (ArC), 116.2 (ArC), 110.4 (C^{11}), 106.7 (C^{28}), 66.7 (C^8), 46.4 (C^{22}), 45.5 (C^{25}), 42.3 (C^5), 26.9 (C^2), 25.6 (C^{23}), 23.7 (C^{24}), 23.5 (C^9); **HRMS** (ESl): calculated for $C_{23}H_{27}N_2O_3$ $[M+H]^+$ requires *m/z* 379.2016, found *m/z* 379.2022; **Chiral SFC:** YMC Chiral ART Cellulose-SC column (25 cm), CO_2:*i*-PrOH 75:25, 2 mL/min, 182 bar, r.t. Retention times: 7.5 mins (major), 7.8 mins (minor), e.r. = 98:2.

SFC analysis of the racemate, prepared using rac-BINAP:

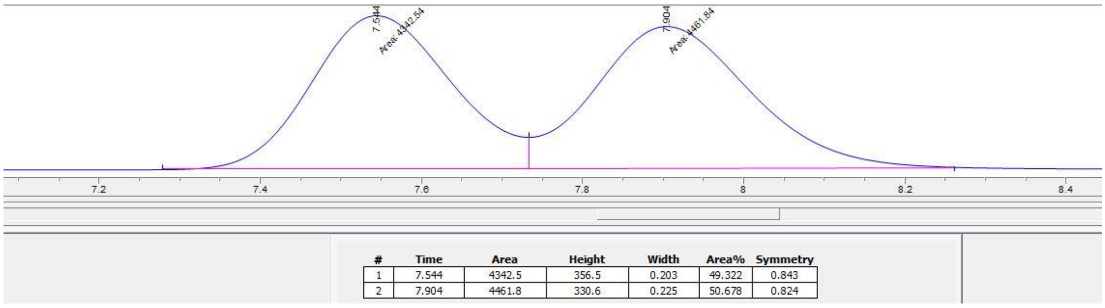

#	Time	Area	Height	Width	Area%	Symmetry
1	7.544	4342.5	356.5	0.203	49.322	0.843
2	7.904	4461.8	330.6	0.225	50.678	0.824

SFC analysis of the enantioenriched material, prepared using (R)-DM-SEGPHOS:

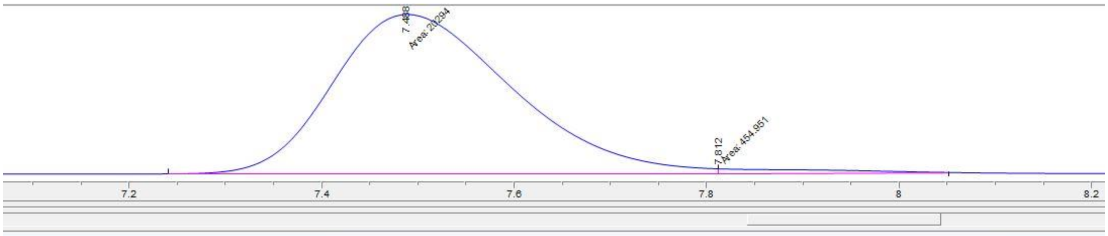

#	Time	Area	Height	Width	Area%	Symmetry
1	7.488	20294	1561.4	0.2166	97.807	0.695
2	7.812	455	49.2	0.1541	2.193	0

(*S*)-2-((4-Hydroxyphenyl)amino)-3-methyl-1-(pyrrolidin-1-yl)-3-(1-tosyl-1*H*-indol-3-yl)butan-1-one (3io):

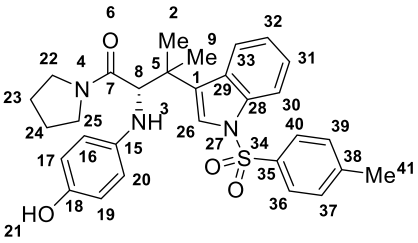

General procedure B: The reaction was carried out with substrate **1i** (22.0 mg, 0.10 mmol, 100 mol%) and 3-(prop-1-en-2-yl)-1-tosyl-1*H*-indole **2o** (124 mg, 0.40 mmol, 400 mol%). Purification of the residue by FCC (DCM/EtOAc 85:15) afforded the title compound (46.7 mg, 88%, >30:1 B:L, e.r. = 99:1) as a pale-yellow solid. ^{1}H NMR analysis of the crude material gave >30:1 B:L. **m.p.** = 222 – 224 °C (hexane/EtOAc); $[\boldsymbol{\alpha}]_{\boldsymbol{D}}^{\mathbf{25}}$ = +15.3 (c = 1.0, $CHCl_3$); **IR (thin film)** ν_{max}/cm^{-1}: 3242 (br), 2929 (s), 1595 (s), 1512 (s), 1355 (s), 1277 (s), 1124 (s), 745 (s), 682 (s); **^{1}H NMR** (500 MHz, DMSO-D$_6$) δ 8.49 (s, 1H, H^{21}), 7.93 – 7.89 (m, 3H, H^{33} + H^{36} + H^{40}), 7.75 (d, *J* = 8.0 Hz, 1H, H^{30}), 7.62 (s, 1H, H^{26}), 7.36 (d, *J* = 8.5 Hz, 2H, H^{37} + H^{39}), 7.31 – 7.28 (m, 1H, H^{31}), 7.24 – 7.21 (m, 1H, H^{32}), 6.53 – 6.48 (m, 4H, H^{16} + H^{17} + H^{19} + H^{20}), 4.76 (d, *J* = 10.5 Hz, 1H, H^3), 4.14 (d, *J* = 10.5 Hz, 1H, H^8), 2.98 – 2.89 (m, 2H, H^{22}), 2.70 – 2.65 (m, 1H, H^{25}), 2.32 (s, 3H, H^{41}), 2.23 – 2.18 (m, 1H, H^{25}), 1.60 (s, 3H, H^2), 1.53 (s, 3H, H^9), 1.31 – 1.26 (m, H, H^{23}), 1.15 – 1.10 (m, H, H^{23}), 0.84 – 0.76 (m, 1H, H^{24}), 0.50 – 0.42 (m, 1H, H^{24}); **^{13}C NMR** (126 MHz, DMSO-D$_6$) δ 170.9 (C^7), 149.6 (C^{15}), 145.8 (C^1), 141.3 (C^{18}), 134.9 (ArC), 134.6 (ArC), 130.6 (C^{36} + C^{40}), 129.7 (ArC), 128.8 (ArC), 127.4 (C^{37} + C^{39}), 124.7 (ArC), 124.5 (ArC), 123.1 (ArC), 122.7 (C^{32}), 116.0 (ArC), 115.4 (ArC), 113.5 (C^{33}), 63.0 (C^8), 46.5 (C^{22}), 45.7 (C^{25}), 39.9 (C^5), 27.2 (C^2), 25.3 (C^{23}), 23.4 (C^{24}), 23.1 (C^9), 21.5 (C^{41}); **HRMS** (ESl): calculated for $C_{30}H_{34}N_3O_4S$ [M+H]$^+$ requires *m/z* 532.2265, found *m/z* 532.2264; **Chiral SFC:** YMC Chiral ART Cellulose-SC column (25 cm), CO_2:*i*-PrOH 75:25, 3 mL/min, 228 bar, r.t. Retention times: 8.1 mins (major), 10.8 mins (minor), e.r. = 99:1.

SFC analysis of the racemate, prepared using rac-BINAP:

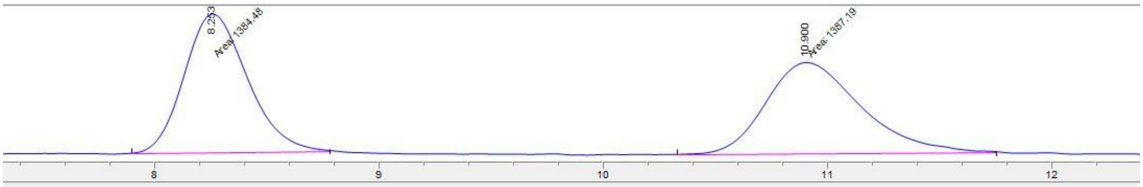

#	Time	Area	Height	Width	Area%	Symmetry
1	8.253	1384.5	69.6	0.3316	49.951	0.797
2	10.9	1387.2	46	0.5029	50.049	0.722

SFC analysis of the enantioenriched material, prepared using (R)-DM-SEGPHOS:

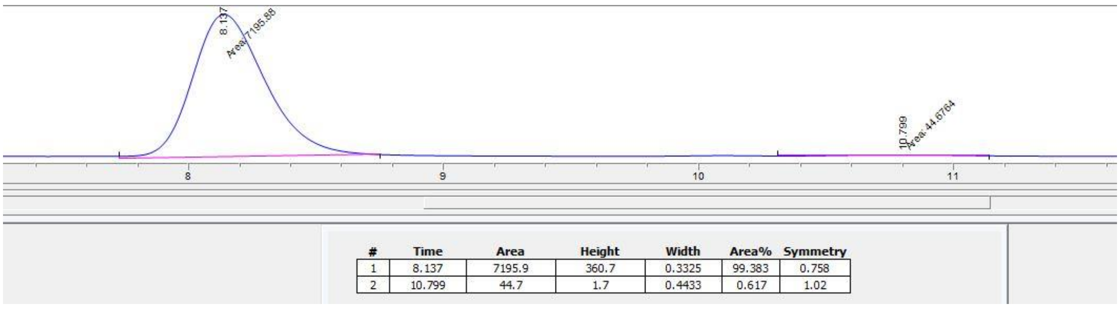

#	Time	Area	Height	Width	Area%	Symmetry
1	8.137	7195.9	360.7	0.3325	99.383	0.758
2	10.799	44.7	1.7	0.4433	0.617	1.02

(*S*)-3-(Benzofuran-2-yl)-2-((4-hydroxyphenyl)amino)-3-methyl-1-(pyrrolidin-1-yl)butan-1-one (3ip):

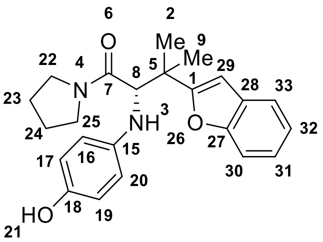

General procedure B: The reaction was carried out with substrate **1i** (22.0 mg, 0.10 mmol, 100 mol%) and 2-(prop-1-en-2-yl)benzofuran **2p** (63.2 mg, 0.40 mmol, 400 mol%). Purification of the residue by FCC (hexane/EtOAc 50:50) afforded the title compound (25.3 mg, 67%, >30:1 B:L, e.r. = 88:12) as a pale-yellow oil. ^{1}H NMR analysis of the crude material gave >30:1 B:L. $[\boldsymbol{\alpha}]_{\boldsymbol{D}}^{\mathbf{25}}$ = -5.1 (c = 1.0, $CHCl_3$); **IR (thin film)** ν_{max}/cm^{-1}: 3253 (br), 2974 (s), 1617 (s), 1514 (s), 1452 (s), 1241 (s), 822 (s), 736 (s); **^{1}H NMR** (500 MHz, $CDCl_3$) δ 7.50 (d, *J* = 9.0 Hz, 1H, H^{33}), 7.45 (d, *J* = 9.0 Hz, 1H, H^{30}), 7.26 – 7.23 (m, 1H, H^{31}), 7.22 – 7.19 (m, 1H, H^{32}), 6.65 (d, *J* = 9.0 Hz, 2H, H^{17} + H^{19}), 6.59 (d, *J* = 9.0 Hz, 2H, H^{16} + H^{20}), 6.53 (s, 1H, H^{29}), 5.67 (br. s, 1H, H^{21}), 4.39 (s, 1H, H^8), 4.29 (br. s, 1H, H^3), 3.36 – 3.31 (m, 1H, H^{22}), 3.21 – 3.16 (m, 2H, H^{22} + H^{25}), 3.07 – 3.03 (m, 1H, H^{25}), 1.63 (s, 3H, H^2), 1.61 – 1.55 (m, 2H, H^{23}), 1.54 (s, 3H, H^9), 1.43 – 1.36 (m, 1H, H^{24}), 1.33 – 1.28 (m, 1H, H^{24}); **^{13}C NMR** (126 MHz, $CDCl_3$) δ 171.0 (C^7), 163.4 (C^1), 154.4 (C^{27}), 149.0 (C^{15}), 141.6 (C^{18}), 128.6 (C^{28}), 123.7 (C^{31}), 122.7 (C^{32}), 120.8 (C^{33}), 116.5 (ArC), 116.2 (ArC), 110.7 (C^{30}), 102.4 (C^{29}), 64.1 (C^8), 46.7 (C^{22}), 45.6 (C^{25}), 40.8 (C^5), 25.8 (C^{23}), 24.2 (C^2), 23.9 (C^{24}), 21.7 (C^9); **HRMS** (ESI): calculated for $C_{23}H_{27}N_2O_3$ [M+H]$^+$ requires *m/z* 379.2016, found *m/z* 379.2022; **Chiral SFC:** YMC Chiral ART Cellulose-SC column (25 cm), CO_2:*i*-PrOH 75:25, 3 mL/min, 228 bar, r.t. Retention times: 4.0 mins (minor), 4.5 mins (major), e.r. = 88:12.

SFC analysis of the racemate, prepared using rac-BINAP:

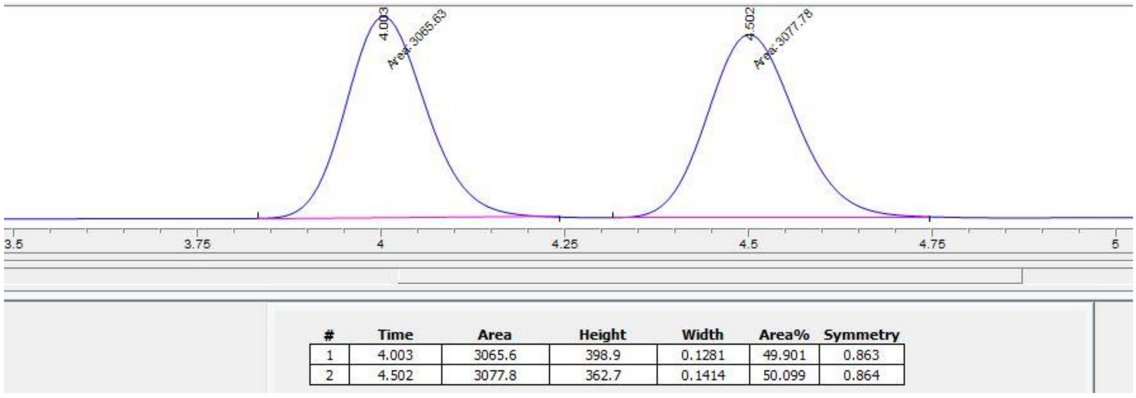

#	Time	Area	Height	Width	Area%	Symmetry
1	4.003	3065.6	398.9	0.1281	49.901	0.863
2	4.502	3077.8	362.7	0.1414	50.099	0.864

SFC analysis of the enantioenriched material, prepared using (R)-DM-SEGPHOS:

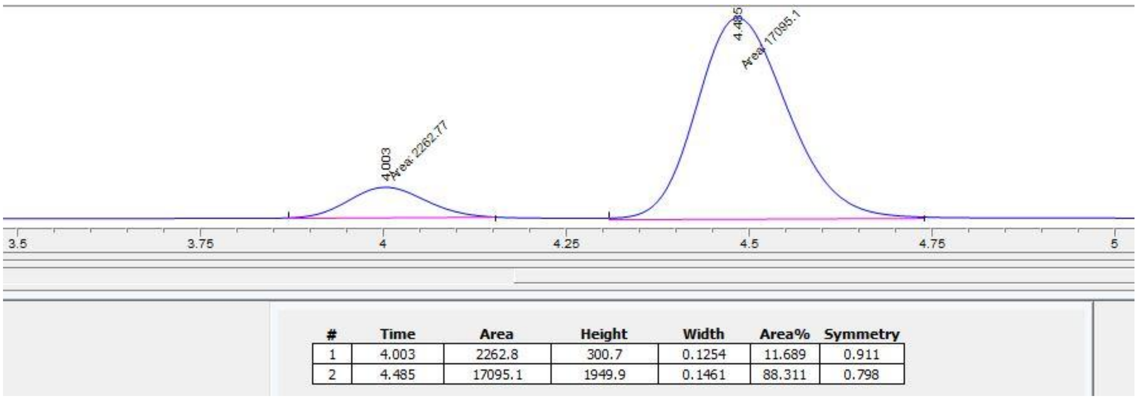

#	Time	Area	Height	Width	Area%	Symmetry
1	4.003	2262.8	300.7	0.1254	11.689	0.911
2	4.485	17095.1	1949.9	0.1461	88.311	0.798

(*S*)-2-((4-Hydroxyphenyl)amino)-3-methyl-1-(pyrrolidin-1-yl)-3-(thiophen-3-yl)butan-1-one (3iq):

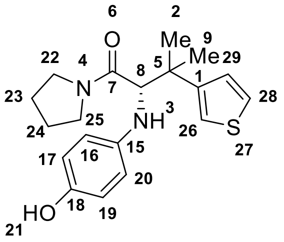

General procedure B: The reaction was carried out with substrate **1i** (22.0 mg, 0.10 mmol, 100 mol%) and 3-(prop-1-en-2-yl)thiophene **2q** (50.0 µL, 0.40 mmol, 400 mol%). Purification of the residue by FCC (hexane/EtOAc 50:50) afforded the title compound (24.1 mg, 70%, >30:1 B:L, e.r. = 98:2) as a pale-yellow oil. ^{1}H NMR analysis of the crude material gave >30:1 B:L. $[\alpha]_D^{25}$ = -4.9 (c = 1.0, $CHCl_3$); **IR (thin film)** ν_{max}/cm^{-1}: 3252 (br), 2971 (s), 1612 (s), 1514 (s), 1449 (s), 1240 (s), 788 (s), 735 (s); **^{1}H NMR** (500 MHz, $CDCl_3$) δ 7.26 – 7.24 (m, 1H, H^{28}), 7.18 – 7.17 (m, 1H, H^{26}), 7.15 – 7.13 (m, 1H, H^{29}), 6.65 (d, *J* = 9.0 Hz, 2H, H^{17} + H^{19}), 6.55 (d, *J* = 9.0 Hz, 2H, H^{16} + H^{20}), 5.39 (br. s, 1H, H^{21}), 4.26 (br. s, 1H, H^3), 3.94 (s, 1H, H^8), 3.37 – 3.31 (m, 1H, H^{22}), 3.25 – 3.20 (m, 1H, H^{22}), 3.08 – 3.03 (m, 1H, H^{25}), 2.51 – 2.46 (m, 1H, H^{25}), 1.66 – 1.56 (m, 6H, H^2 + H^{23} + H^{24}), 1.54 – 1.45 (m, 4H, H^9 + H^{24}); **^{13}C NMR**

(126 MHz, $CDCl_3$) δ 171.3 (C^7), 148.8 (C^{15}), 148.4 (C^1), 141.9 (C^{18}), 127.4 (C^{29}), 124.7 (C^{28}), 120.8 (C^{26}), 116.3 (ArC), 116.2 (ArC), 65.9 (C^8), 46.5 (C^{22}), 45.6 (C^{25}), 40.7 (C^5), 27.0 (C^{23}), 25.9 (C^2), 23.9 (C^{24}), 23.4 (C^9); **HRMS** (ESI): calculated for $C_{19}H_{25}N_2O_2S$ $[M+H]^+$ requires *m/z* 345.1631, found *m/z* 345.1635; **Chiral SFC:** YMC Chiral ART Cellulose-SC column (25 cm), CO_2:*i*-PrOH 75:25, 3 mL/min, 228 bar, r.t. Retention times: 4.5 mins (minor), 7.5 mins (major), e.r. = 98:2.

SFC analysis of the racemate, prepared using rac-BINAP:

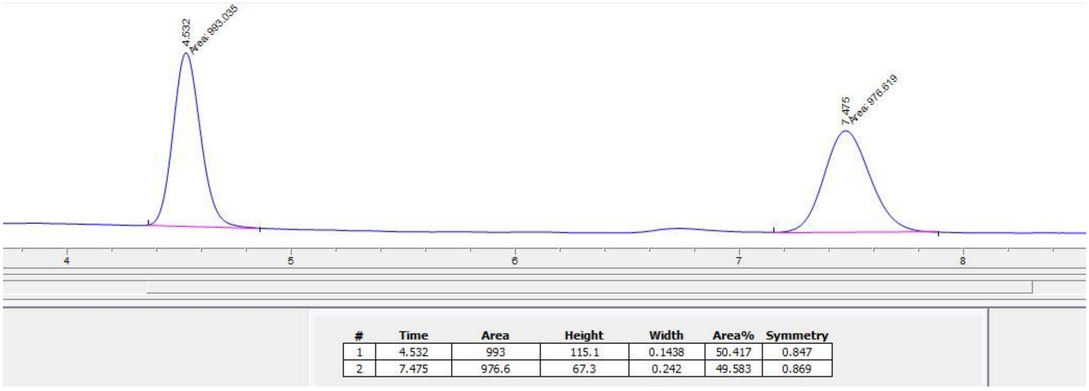

#	Time	Area	Height	Width	Area%	Symmetry
1	4.532	993	115.1	0.1438	50.417	0.847
2	7.475	976.6	67.3	0.242	49.583	0.869

SFC analysis of the enantioenriched material, prepared using (R)-DM-SEGPHOS:

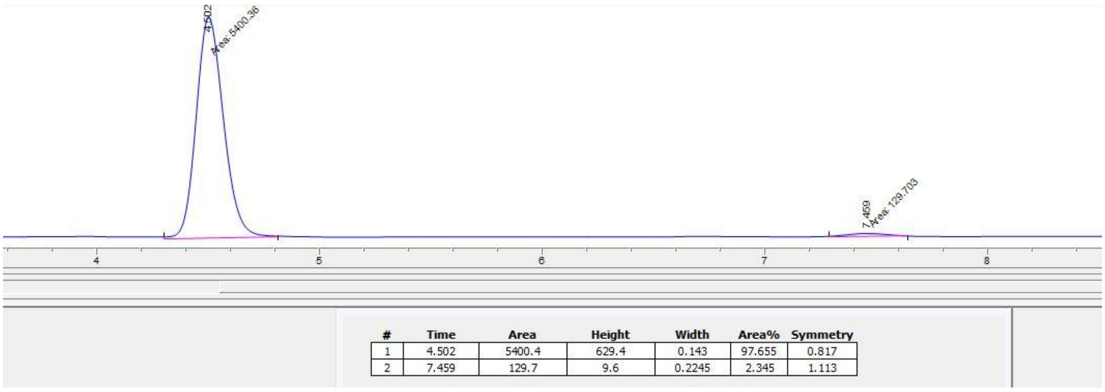

#	Time	Area	Height	Width	Area%	Symmetry
1	4.502	5400.4	629.4	0.143	97.655	0.817
2	7.459	129.7	9.6	0.2245	2.345	1.113

(8*R*,9*S*,13*R*,14*S*)-3-((*S*)-3-((4-Hydroxyphenyl)amino)-2-methyl-4-oxo-4-(pyrrolidin-1-yl)butan-2-yl)-13-methyl-6,7,8,9,11,12,13,14,15,16-decahydro-17*H*-cyclopenta[*a*]phenanthren-17-one (3ir):

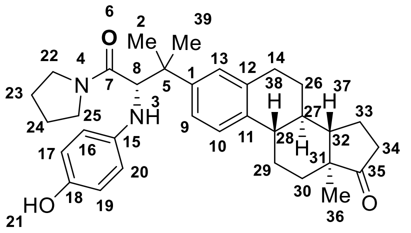

General procedure B: The reaction was carried out with substrate **1i** (22.0 mg, 0.10 mmol, 100 mol%), (8*R*,9*S*,13*R*,14*S*)-13-methyl-3-(prop-1-en-2-yl)-6,7,8,9,11,12,13,14,15,16-

decahydro-17*H*-cyclopenta[*a*]phenanthren-17-one **2r** (118 mg, 0.40 mmol, 400 mol%) and was run in *t*-BuOH (0.2 mL). Purification of the residue by FCC (hexane/EtOAc 60:40) afforded the title compound (25.7 mg, 50%, >30:1 B:L, >20:1 d.r.) as a brown solid. ^{1}H NMR analysis of the crude material gave >30:1 B:L and >20:1 d.r. **m.p.** = 191 – 193 °C (hexane/EtOAc); **IR (thin film)** ν_{max}/cm^{-1}: 3242 (br), 2974 (s), 2920 (s), 1680 (s), 1645 (s), 1516 (s), 1450 (s), 1240 (s), 826 (s), 734 (s); **^{1}H NMR** (500 MHz, $CDCl_3$) δ 7.31 (d, *J* = 8.5 Hz, 1H, H^9), 7.24 (d, *J* = 8.5 Hz, 1H, H^{10}), 7.20 (s, 1H, H^{13}), 6.68 (d, *J* = 8.5 Hz, 2H, H^{17} + H^{19}), 6.56 (d, *J* = 8.5 Hz, 2H, H^{16} + H^{20}), 6.14 (br. s, 1H, H^{21}), 4.24 (br. s, 1H, H^3), 3.93 (s, 1H, H^8), 3.33 – 3.27 (m, 1H, H^{22}), 3.22 – 3.17 (m, 1H, H^{22}), 2.99 – 2.90 (m, 3H, H^{25}), 2.56 – 2.51 (m, 1H), 2.48 – 2.41 (m, 1H), 2.35 – 2.26 (m, 2H), 2.21 – 2.03 (m, 3H), 2.00 – 1.98 (m, 1H), 1.70 – 1.40 (m, 15H), 1.35 – 1.29 (m, 1H), 0.92 (s, 3H, H^{36}); **^{13}C NMR** (126 MHz, $CDCl_3$) δ 208.1 (C^{35}), 171.4 (C^7), 149.1 (C^{15}), 143.6 (C^1), 141.8 (C^{18}), 137.9 (C^{12}), 135.8 (C^{11}), 127.7 (C^{13}), 124.9 (C^{10}), 124.3 (C^9), 116.3 (C^{17} + C^{19}), 116.2 (C^{16} + C^{20}), 66.4 (C^8), 50.5, 48.0, 46.4 (C^{22}), 45.6 (C^{25}), 44.3, 41.9, 38.3, 35.9, 31.6, 29.7, 26.7 (C^2), 26.2, 25.82, 25.80, 23.8, 23.0 (C^{39}), 21.6, 13.8 (C^{36}); **HRMS** (ESl): calculated for $C_{33}H_{43}N_2O_3$ $[M+H]^+$ requires *m/z* 515.3268, found *m/z* 515.3277.

(*2S,3R*)-3-Cyclobutyl-2-((4-hydroxyphenyl)amino)-3-phenyl-1-(pyrrolidin-1-yl)butan-1-one (3is):

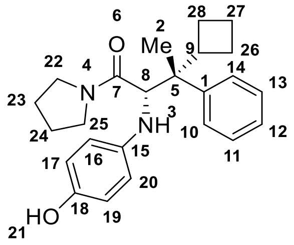

General procedure B: The reaction was carried out with substrate **1i** (22.0 mg, 0.10 mmol, 100 mol%), (1-cyclobutylvinyl)benzene **2s** (160 µL, 1.00 mmol, 1000 mol%), Ir(cod)$_2$BARF (12.7 mg, 10.0 µmol, 10 mol%) and (*R*)-DM-SEGPHOS (7.23 mg, 10.0 µmol, 10 mol%). Purification of the residue by FCC (hexane/EtOAc 50:50) afforded the title compound (13.6 mg, 36%, >30:1 B:L, d.r. 2:1 *a:b*, e.r. = 95:5) as a pale-yellow oil. ^{1}H NMR analysis of the crude material gave >30:1 B:L and 2:1 d.r. $[\boldsymbol{\alpha}]_{D}^{25}$ = +28.2 (c = 1.0, $CHCl_3$); **IR (thin film)** ν_{max}/cm^{-1}: 3245 (br), 2927 (s), 1612 (s), 1514 (s), 1445 (s), 1241 (s), 824 (s), 736 (s), 701 (s); **^{1}H NMR** (500 MHz, $CDCl_3$) Data for the major diastereomer *a*: δ 7.45 (d, *J* = 7.5 Hz, 2H, H^{10} + H^{14}), 7.32 – 7.30 (m, 2H, H^{11} + H^{13}), 7.22 – 7.19 (m, 1H, H^{12}), 6.67 (d, *J* = 8.5 Hz, 2H, H^{17} + H^{19}), 6.56 (d, *J* = 8.5 Hz, 2H, H^{16} + H^{20}), 5.43 (br. s, 1H, H^{21}), 4.18 – 3.96 (m, 2H, H^3 + H^8),

3.31 – 3.21 (m, 1.8H, H^9 + H^{22}), 3.07 – 3.01 (m, 1H, H^{22}), 2.91 – 2.86 (m, 0.7H, H^{25}), 2.33 – 2.28 (m, 1H, H^{25}), 2.26 – 2.18 (m, 1H, $H^{26\text{-}28}$), 2.09 – 2.00 (m, 1H, $H^{26\text{-}28}$), 1.88 – 1.73 (m, 2H, $H^{26\text{-}28}$), 1.68 – 1.63 (m, 2H, $H^{26\text{-}28}$), 1.60 (s, 3H, H^2), 1.56 – 1.48 (m, 2H, H^{23}), 1.44 – 1.39 (m, 1H, H^{24}), 1.34 – 1.29 (m, 1H, H^{24}). Characteristic signals for the minor diastereomer *b*: 3.67 – 3.60 (m, 0.4H, H^9), 3.38 – 3.33 (m, 0.4H, H^{22}), 2.33 – 2.28 (m, 0.3H, H^{25}); **^{13}C NMR** (126 MHz, $CDCl_3$) Data for the major diastereomer *a*: δ 170.9 (C^7), 148.5 (C^{15}), 144.0 (C^1), 141.5 (C^{18}), 127.9 (C^{11} + C^{13}), 127.6 (C^{10} + C^{14}), 126.4 (ArC), 116.2 (C^{17} + C^{19}), 115.8 (C^{16} + C^{20}), 66.3 (C^8), 46.3 (C^{22}), 46.0 (C^5), 45.4 (C^{25}), 44.6 (C^9), 25.8 (C^{23}), 25.4 ($C^{26\text{-}28}$), 24.8 ($C^{26\text{-}28}$), 18.1 (C^2), 23.7 (C^{24}), 14.8 ($C^{26\text{-}28}$). Data for the minor diastereomer *b*: δ 143.5 (C^1), 127.8 (C^{11} + C^{13}), 126.4 (ArC), 46.5 (C^{22}), 45.7 (C^{25}), 39.7 (C^9), 25.9 (C^{23}), 25.1 ($C^{26\text{-}28}$), 24.2 ($C^{26\text{-}28}$), 23.8 (C^{24}), 18.0 (C^2), 17.5 ($C^{26\text{-}28}$); **HRMS** (ESl): calculated for $C_{24}H_{31}N_2O_2$ $[M+H]^+$ requires *m/z* 379.2380, found *m/z* 379.2385; **Chiral SFC:** YMC Chiral ART Cellulose-SC column (25 cm), CO_2:*i*-PrOH 75:25, 3 mL/min, 228 bar, r.t. Retention times: 6.2 mins (major), 7.1 mins (minor), e.r. = 95:5.

SFC analysis of the racemates, prepared using rac-BINAP:

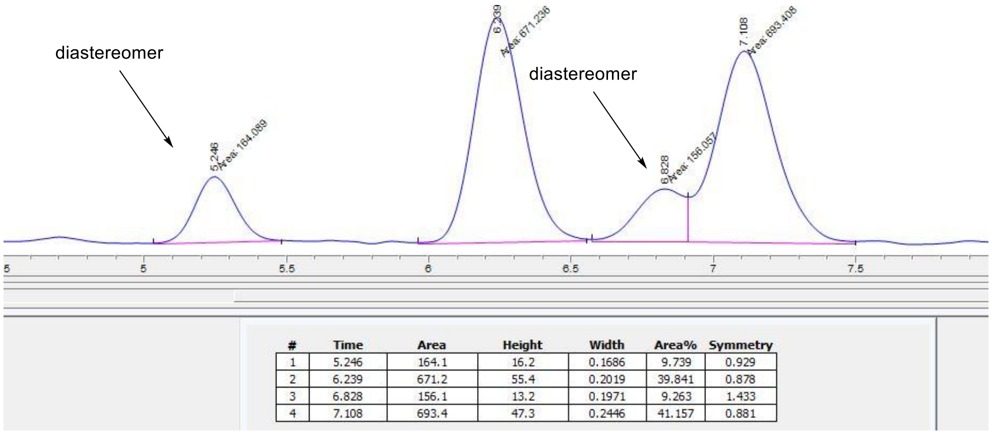

#	Time	Area	Height	Width	Area%	Symmetry
1	5.246	164.1	16.2	0.1686	9.739	0.929
2	6.239	671.2	55.4	0.2019	39.841	0.878
3	6.828	156.1	13.2	0.1971	9.263	1.433
4	7.108	693.4	47.3	0.2446	41.157	0.881

SFC analysis of the major diastereomer of enantioenriched material, prepared using (R)-DM-SEGPHOS:

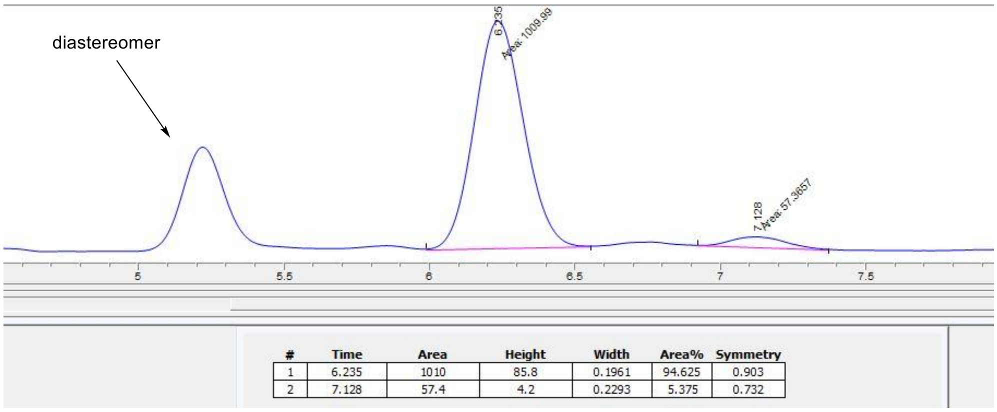

#	Time	Area	Height	Width	Area%	Symmetry
1	6.235	1010	85.8	0.1961	94.625	0.903
2	7.128	57.4	4.2	0.2293	5.375	0.732

(*2S*,*3R*)-4,4,4-Trifluoro-2-((4-hydroxyphenyl)amino)-3-methyl-3-phenyl-1-(pyrrolidin-1-yl)butan-1-one (3it):

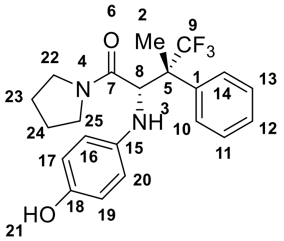

General procedure B: The reaction was carried out with substrate **1i** (22.0 mg, 0.10 mmol, 100 mol%), (3,3,3-trifluoroprop-1-en-2-yl)benzene **2t** (148 µL, 1.00 mmol, 1000 mol%), Ir(cod)$_2$BARF (12.7 mg, 10.0 µmol, 10 mol%) and (*R*)-DM-SEGPHOS (7.23 mg, 10.0 µmol, 10 mol%). Purification of the residue by FCC (hexane/EtOAc 60:40) afforded the title compound (30.6 mg, 78%, >30:1 B:L, d.r. 4:1 *a*:*b*, e.r. = 87:13) as a pale-yellow oil. ^{1}H NMR analysis of the crude material gave >30:1 B:L and 4:1 d.r. $[\boldsymbol{\alpha}]_{\boldsymbol{D}}^{\mathbf{25}}$ = -23.3 (c = 1.0, CHCl$_3$); **IR (thin film)** ν_{max}/cm^{-1}: 3337 (br), 2976 (s), 1618 (s), 1514 (s), 1446 (s), 1261 (s), 1150 (s), 827 (s), 735 (s), 699 (s); **^{1}H NMR** (500 MHz, CDCl$_3$) Data for the major diastereomer *a*: δ 7.59 (d, *J* = 7.5 Hz, 1.6H, H^{10} + H^{14}), 7.38 – 7.30 (m, 2.6H, H^{11} + H^{12} + H^{13}), 6.73 – 6.65 (m, 3.3H, H^{16} + H^{17} + H^{19} + H^{20}), 5.63 (br. s, 0.9H, H^{21}), 4.66 (d, *J* = 10.5 Hz, 1H, H^8), 4.17 – 4.10 (m, 0.9H, H^3), 3.21 – 3.14 (m, 0.8H, H^{22}), 2.98 – 2.93 (m, 0.8H, H^{22}), 2.90 – 2.85 (m, 0.8H, H^{25}), 2.69 – 2.64 (m, 0.8H, H^{25}), 1.94 (s, 2.5H, H^2), 1.86 – 1.76 (m, 0.6H, H^{23}), 1.58 – 1.48 (m, 1.6H, H^{23}), 1.43 – 1.27 (m, 2H, H^{24}). Characteristic signals for the minor diastereomer *b*: δ 7.65 (d, *J* = 7.5 Hz, 0.4H, H^{10} + H^{14}), 7.44 – 7.41 (m, 0.4H, H^{11} + H^{13}), 6.59 (d, *J* = 9.0 Hz, 0.4H, H^{17} + H^{19}), 6.41 (d, *J* = 9.0 Hz, 0.4H, H^{16} + H^{20}), 3.69 – 3.64 (m, 0.2H, H^{22}), 3.51 – 3.40 (m, 0.5H, H^{22} + H^{25}), 3.34 – 3.30 (m, 0.2H, H^{25}), 1.88 (s, 0.5H, H^2); **^{13}C NMR** (126 MHz, CDCl$_3$) Data for the major diastereomer *a*: δ 169.2 (C^7), 149.7 (C^{15}), 140.6 (C^{18}), 136.9 (C^1), 128.3 (ArC), 128.2 (q, *J* = 283.8 Hz, C^9), 128.2 (ArC), 128.1 (ArC), 128.0 (ArC), 127.9 (ArC), 127.5 (q, *J* = 2.5 Hz, ArC), 117.5 (ArC), 116.3 (ArC), 116.2 (ArC), 61.1 (C^8), 52.4 (q, *J* = 22.5 Hz, C^5), 46.6 (C^{22}),

45.4 (C^{25}), 25.6 (C^{23}), 23.7 (C^{24}), 14.3 (q, J = 2.5 Hz, C^{2}). Data for the minor diastereomer *b*: δ 169.9 (C^{7}), 150.0 (C^{15}), 140.4 (C^{18}), 137.4 (C^{1}), 118.0 (ArC), 116.0 (ArC), 61.4 (C^{8}), 46.9 (C^{22}), 46.1 (C^{25}), 26.0 (C^{23}), 24.0 (C^{24}), 14.5 (C^{2}); ^{19}F NMR (471 MHz, $CDCl_3$) Data for the major diastereomer *a*: δ -67.0. Data for the minor diastereomer *b*: δ -69.3; **HRMS** (ESI): calculated for $C_{21}H_{24}F_3N_2O_2$ $[M+H]^+$ requires *m/z* 393.1784, found *m/z* 393.1791; **Chiral SFC:** YMC Chiral ART Cellulose-SC column (25 cm), CO_2:*i*-PrOH 90:10, 2 mL/min, 177 bar, r.t. Retention times: 13.2 mins (minor), 14.9 mins (major), e.r. = 87:13.

SFC analysis of the racemate, prepared using rac-BINAP:

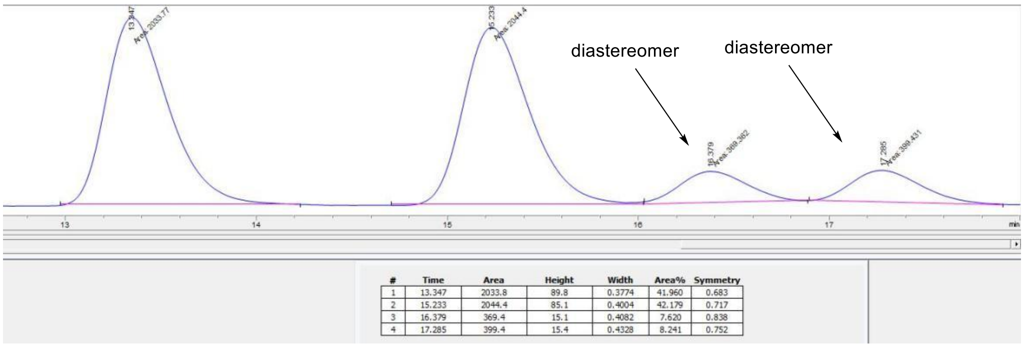

#	Time	Area	Height	Width	Area%	Symmetry
1	13.347	2033.8	89.8	0.3774	41.960	0.683
2	15.233	2044.4	85.1	0.4004	42.179	0.717
3	16.379	369.4	15.1	0.4082	7.620	0.838
4	17.285	399.4	15.4	0.4328	8.241	0.752

SFC analysis of the major diastereomer of enantioenriched material, prepared using (R)-DM-SEGPHOS:

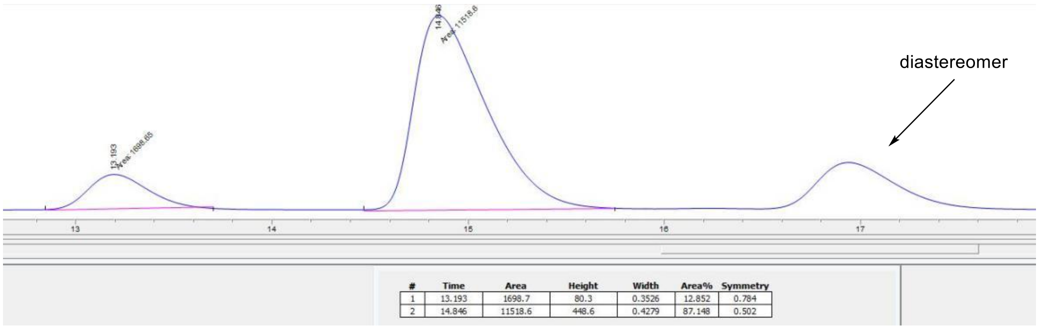

#	Time	Area	Height	Width	Area%	Symmetry
1	13.193	1698.7	80.3	0.3526	12.852	0.784
2	14.846	11518.6	448.6	0.4279	87.148	0.502

(*S*)-2-((4-Hydroxyphenyl)amino)-2-((*R*)-7-methylbicyclo[4.2.0]octa-1(6),2,4-trien-7-yl)-1-(pyrrolidin-1-yl)ethan-1-one (3iu):

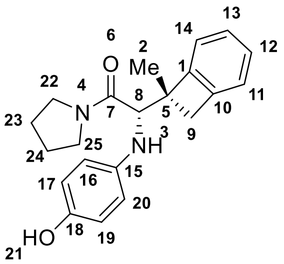

General procedure B: The reaction was carried out with substrate **1i** (22.0 mg, 0.10 mmol, 100 mol%), 7-methylenebicyclo[4.2.0]octa-1(6),2,4-triene **2u** (116 μL, 1.00 mmol, 1000

mol%), Ir(cod)$_2$BARF (12.7 mg, 10.0 μmol, 10 mol%) and (*R*)-DM-SEGPHOS (7.23 mg, 10.0 μmol, 10 mol%). Purification of the residue by FCC (hexane/EtOAc 60:40) afforded the title compound (11.8 mg, 35%, >30:1 B:L, >20:1 d.r., e.r. = 98:2) as a pale-yellow oil. ^{1}H NMR analysis of the crude material gave >30:1 B:L and >20:1 d.r. $[\boldsymbol{\alpha}]_{\boldsymbol{D}}^{\mathbf{25}}$ = -24.7 (c = 1.0, $CHCl_3$); **IR (thin film)** ν_{max}/cm^{-1}: 3231 (br), 2961 (s), 1619 (s), 1515 (s), 1457 (s), 1241 (s), 828 (s), 735 (s); **^{1}H NMR** (500 MHz, $CDCl_3$) δ 7.27 – 7.24 (m, 1H, H^{13}), 7.19 – 7.16 (m, 2H, H^{12} + H^{14}), 6.93 (d, *J* = 7.0 Hz, 1H, H^{11}), 6.69 – 6.64 (m, 4H, H^{16} + H^{17} + H^{19} + H^{20}), 6.16 (br. s, 1H, H^{21}), 4.26 (s, 1H, H^8), 3.58 – 3.53 (m, 1H, H^{22}), 3.49 – 3.44 (m, 1H, H^{22}), 3.36 (d, *J* = 14.5 Hz, 1H, H^9), 3.17 – 3.13 (m, 1H, H^{25}), 3.05 (d, *J* = 14.5 Hz, 1H, H^9), 2.80 – 2.76 (m, 1H, H^{25}), 1.85 – 1.64 (m, 4H, H^{23} + H^{24}), 1.60 (s, 3H, H^2); **^{13}C NMR** (126 MHz, $CDCl_3$) δ 170.8 (C^7), 150.1 (C^{15}), 142.2 (C^1), 128.2 (C^{11}), 126.8 (C^{14}), 124.1 (C^{12}), 121.0 (C^{13}), 117.4 (C^{17} + C^{19}), 116.3 (C^{16} + C^{20}), 63.8 (C^8), 50.8 (C^5), 47.2 (C^{22}), 46.0 (C^{25}), 44.8 (C^9), 25.8 (C^{23}), 24.1 (C^{24}), 19.5 (C^2); **HRMS** (ESl): calculated for $C_{21}H_{25}N_2O_2$ $[M+H]^+$ requires *m/z* 337.1911, found *m/z* 337.1917; **Chiral SFC:** YMC Chiral ART Cellulose-SC column (25 cm), CO_2:*i*-PrOH 75:25, 3 mL/min, 231 bar, r.t. Retention times: 4.9 mins (major), 5.8 mins (minor), e.r. = 98:2.

SFC analysis of the racemate, prepared using rac-BINAP:

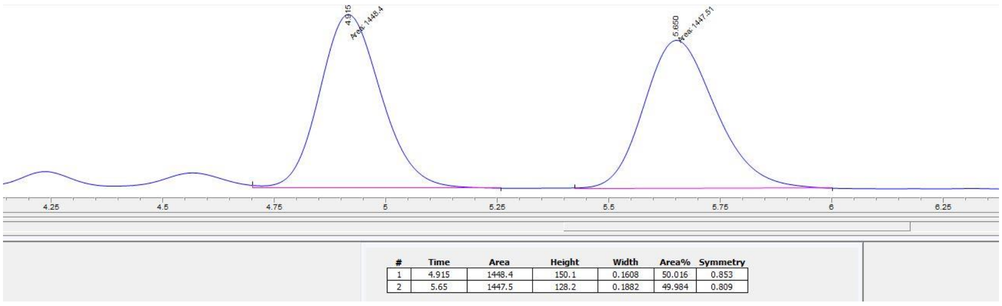

#	Time	Area	Height	Width	Area%	Symmetry
1	4.915	1448.4	150.1	0.1608	50.016	0.853
2	5.65	1447.5	128.2	0.1882	49.984	0.809

SFC analysis of the enantioenriched material, prepared using (R)-DM-SEGPHOS:

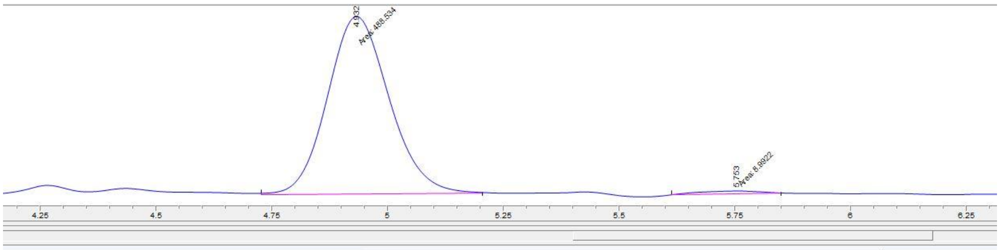

#	Time	Area	Height	Width	Area%	Symmetry
1	4.932	488.5	52.6	0.1547	98.193	0.872
2	5.753	9	9.2E-1	0.163	1.807	1.71

(*2S,3R*)-2-((4-Hydroxyphenyl)amino)-3-methyl-3,5-diphenyl-1-(pyrrolidin-1-yl)pentan-1-one (3iv):

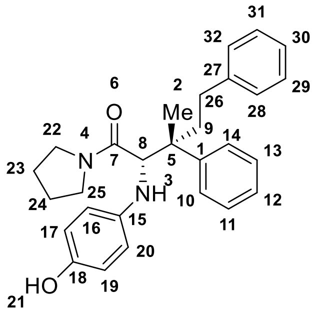

General procedure B: The reaction was carried out with substrate **1i** (22.0 mg, 0.10 mmol, 100 mol%), but-3-ene-1,3-diyldibenzene **2v** (208 mg, 1.00 mmol, 1000 mol%), Ir(cod)$_2$BARF (12.7 mg, 10.0 μmol, 10 mol%) and (*R*)-DM-SEGPHOS (7.23 mg, 10.0 μmol, 10 mol%). Purification of the residue by FCC (hexane/EtOAc 50:50) afforded the title compound (19.7 mg, 46%, >30:1 B:L, 9:1 d.r. *a:b*, e.r. = 95:5) as a brown oil. ^{1}H NMR analysis of the crude material gave >30:1 B:L and 5:1 d.r. $[\alpha]_D^{25}$ = -11.9 (c = 1.0, CHCl$_3$); **IR (thin film)** ν_{max}/cm^{-1}: 3260 (br), 2925 (s), 1610 (s), 1514 (s), 1448 (s), 1241 (s), 828 (s), 701 (s); **^{1}H NMR** (500 MHz, CDCl$_3$) Data for the major diastereomer *a* only: δ 7.55 (d, *J* = 8.0 Hz, 2H, ArH), 7.39 – 7.36 (m, 2H, ArH), 7.31 – 7.25 (m, 3H, ArH), 7.21 – 7.15 (m, 3H, ArH), 6.62 (d, *J* = 8.5 Hz, 2H, H^{17} + H^{19}), 6.54 (d, *J* = 8.5 Hz, 2H, H^{16} + H^{20}), 5.79 (br. s, 1H, H^{21}), 4.27 (br. s, 1H, H^3), 3.95 (s, 1H, H^8), 3.24 – 3.19 (m, 1H, H^{22}), 3.01 – 2.96 (m, 1H, H^{22}), 2.91 – 2.87 (m, 1H, H^{25}), 2.66 – 2.55 (m, 2H, H^{26}), 2.43 – 2.35 (m, 3H, H^9 + H^{25}), 1.71 (s, 3H, H^2), 1.56 – 1.46 (m, 2H, H^{23}), 1.40 – 1.35 (m, 1H, H^{24}), 1.27 – 1.22 (m, 1H, H^{24}); **^{13}C NMR** (126 MHz, CDCl$_3$) Data for the major diastereomer *a* only: δ 171.1 (C^7), 149.2 (C^{15}), 143.5 (C^1), 143.0 (C^{27}), 128.40 (ArC), 128.37 (ArC), 128.2 (ArC), 127.6 (ArC), 126.6 (ArC), 125.7 (ArC), 116.7 (C^{17} + C^{19}), 116.2 (C^{16} + C^{20}), 67.3 (C^8), 46.4 (C^{22}), 45.8 (C^5), 45.3 (C^{25}), 40.1 (C^{26}), 30.2 (C^9), 25.6 (C^{23}), 23.7 (C^{24}), 18.6 (C^2); **HRMS** (ESl): calculated for $C_{28}H_{33}N_2O_2$ [M+H]$^+$ requires *m/z* 429.2537, found *m/z* 429.2545; **Chiral SFC:** YMC Chiral ART Cellulose-SC column (25 cm), CO$_2$:*i*-PrOH 80:20, 2 mL/min, 180 bar, r.t. Retention times: 15.5 mins (minor), 16.2 mins (major), e.r. = 95:5.

SFC analysis of the major diastereomer of racemate, prepared using rac-BINAP:

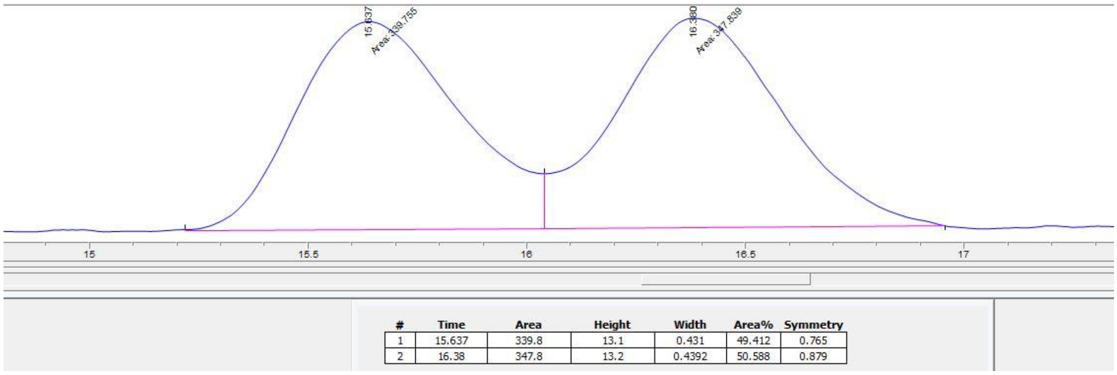

#	Time	Area	Height	Width	Area%	Symmetry
1	15.637	339.8	13.1	0.431	49.412	0.765
2	16.38	347.8	13.2	0.4392	50.588	0.879

SFC analysis of the major diastereomer of enantioenriched material, prepared using (R)-DM-SEGPHOS:

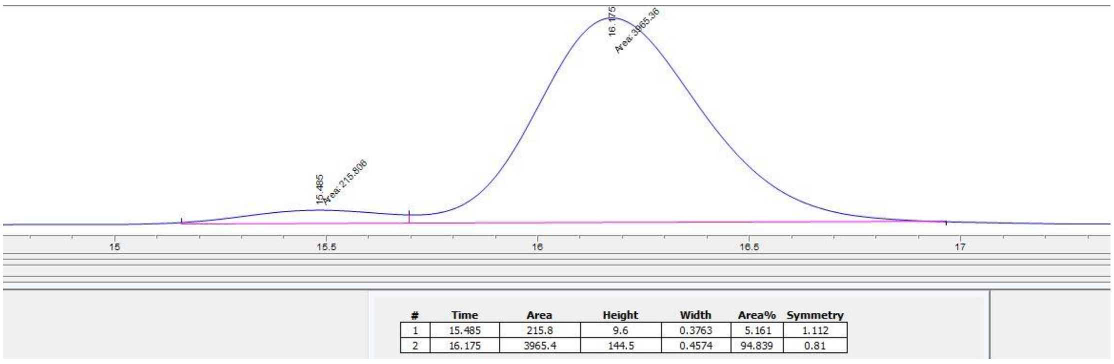

#	Time	Area	Height	Width	Area%	Symmetry
1	15.485	215.8	9.6	0.3763	5.161	1.112
2	16.175	3965.4	144.5	0.4574	94.839	0.81

(*2S,3R*)-2-((4-Hydroxyphenyl)amino)-3-methyl-3-phenyl-1-(pyrrolidin-1-yl)pentan-1-one (3iw):

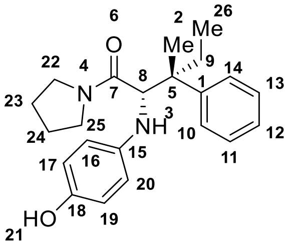

General procedure B: The reaction was carried out with substrate **1i** (22.0 mg, 0.10 mmol, 100 mol%), but-1-en-2-ylbenzene **2w** (147 µL, 1.00 mmol, 1000 mol%), Ir(cod)$_2$BARF (12.7 mg, 10.0 µmol, 10 mol%) and (*R*)-DM-SEGPHOS (7.23 mg, 10.0 µmol, 10 mol%). Purification of the residue by FCC (hexane/EtOAc 50:50) afforded the title compound (27.1 mg, 77%, >30:1 B:L, 7:1 d.r. *a*:*b*, e.r. = 97:3) as a pale-yellow oil. ^{1}H NMR analysis of the crude material gave >30:1 B:L and 7:1 d.r. $[\boldsymbol{\alpha}]_{\boldsymbol{D}}^{\mathbf{25}}$ = -11.8 (c = 1.0, CHCl$_3$); **IR (thin film)** ν_{max}/cm^{-1}: 3246 (br), 2971 (s), 1611 (s), 1514 (s), 1449 (s), 1240 (s), 824 (s), 735 (s), 702 (s); **^{1}H NMR** (500 MHz, CDCl$_3$) Data for the major diastereomer *a*: δ 7.45 (d, *J* = 7.5 Hz, 2H, H^{10} + H^{14}), 7.33 – 7.30 (m, 2H, H^{11} + H^{13}), 7.23 – 7.20 (m, 1H, H^{12}), 6.68 (d, *J* = 8.5 Hz, 2H, H^{17} + H^{19}), 6.59 (d, *J* = 8.5 Hz, 2H, H^{16} + H^{20}), 5.78 (br. s, 1H, H^{21}), 4.31 (br. s, 1H, H^3), 3.95 (s,

1H, H[8]), 3.24 – 3.19 (m, 1H, H[22]), 3.03 – 2.87 (m, 2H, H[22] + H[25]), 2.39 – 2.35 (m, 1H, H[25]), 2.30 – 2.14 (m, 2H, H[9]), 1.57 (s, 3H, H[2]), 1.54 – 1.48 (m, 2H, H[23]), 1.42 – 1.36 (m, 1H, H[24]), 1.29 – 1.22 (m, 1H, H[24]), 0.79 (t, *J* = 7.5 Hz, 2.6H, H[26]). Characteristic signals for the minor diastereomer *b*: 6.52 (d, *J* = 8.5 Hz, 0.3H, H[16] + H[20]), 3.35 – 3.30 (m, 0.2H, H[22]), 2.52 – 2.45 (m, 0.2H, H[25]), 1.61 (s, 0.5H, H[2]), 0.69 (t, *J* = 7.5 Hz, 0.4H, H[26]); **^{13}C NMR** (126 MHz, $CDCl_3$) Data for the major diastereomer *a*: δ 171.0 (C[7]), 149.1 (C[15]), 143.4 (C[1]), 141.3 (C[18]), 127.95 (C[11] + C[13]), 127.72 (C[10] + C[14]), 116.5 (ArC), 116.2 (ArC), 67.2 (C[8]), 46.43 (C[22]), 46.1 (C[5]), 45.3 (C[25]), 30.4 (C[2]), 25.7 (C[23]), 23.7 (C[24]), 18.1 (C[9]), 8.1 (C[26]). Data for the minor diastereomer *b*: δ 127.98 (C[11] + C[13]), 127.70 (C[10] + C[14]), 46.39 (C[22]), 45.6 (C[25]), 26.9 (C[2]), 25.8 (C[23]), 23.8 (C[24]), 21.8 (C[9]), 8.2 (C[26]); **HRMS** (ESl): calculated for $C_{22}H_{29}N_2O_2$ $[M+H]^+$ requires *m/z* 353.2224, found *m/z* 353.2228; **Chiral SFC:** YMC Chiral ART Cellulose-SC column (25 cm), CO_2:*i*-PrOH 80:20, 2 mL/min, 180 bar, r.t. Retention times: 11.3 mins (major), 15.2 mins (minor), e.r. = 97:3.

SFC analysis of the major diastereomer of racemate, prepared using rac-BINAP:

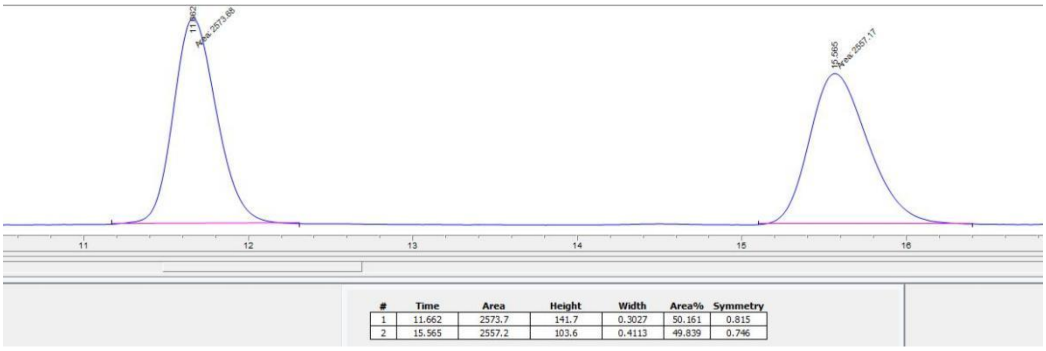

SFC analysis of the major diastereomer of enantioenriched material, prepared using (R)-DM-SEGPHOS:

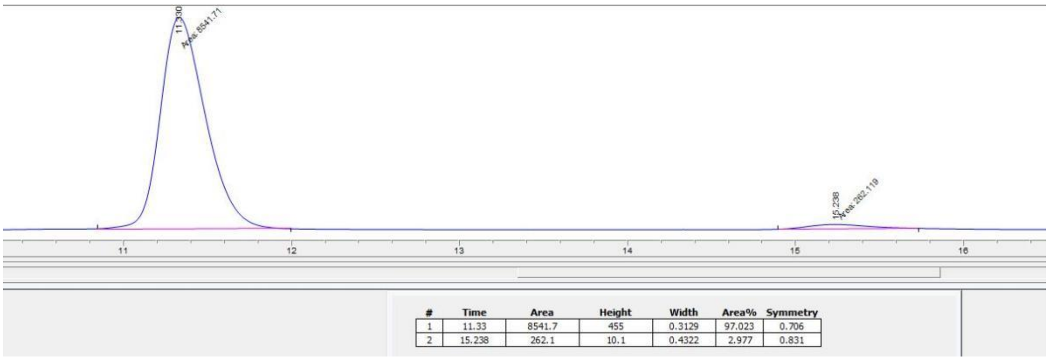

(*2S,3R*)-2-((4-Hydroxyphenyl)amino)-3-methyl-3-phenyl-1-(pyrrolidin-1-yl)nonan-1-one (3ix):

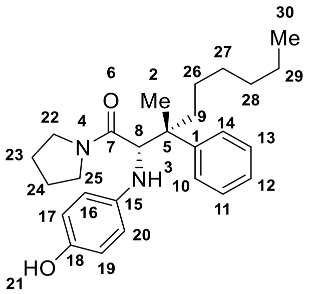

General procedure B: The reaction was carried out with substrate **1i** (22.0 mg, 0.10 mmol, 100 mol%), oct-1-en-2-ylbenzene **2x** (218 µL, 1.00 mmol, 1000 mol%), Ir(cod)$_2$BARF (12.7 mg, 10.0 µmol, 10 mol%) and (*R*)-DM-SEGPHOS (7.23 mg, 10.0 µmol, 10 mol%). Purification of the residue by FCC (hexane/EtOAc 50:50) afforded the title compound (15.5 mg, 38%, >30:1 B:L, >10:1 d.r. *a:b*, e.r. = 90:10) as a pale-yellow oil. ^{1}H NMR analysis of the crude material gave >30:1 B:L and 4:1 d.r. $[\boldsymbol{\alpha}]_{\boldsymbol{D}}^{\mathbf{25}}$ = -8.2 (c = 1.0, CHCl$_3$); **IR (thin film)** ν_{max}/cm^{-1}: 3259 (br), 2926 (s), 1612 (s), 1514 (s), 1445 (s), 1240 (s), 824 (s), 701 (s); **^{1}H NMR** (500 MHz, CDCl$_3$) Data for the major diastereomer *a* only: δ 7.45 (d, *J* = 7.5 Hz, 2H, H^{10} + H^{14}), 7.33 – 7.30 (m, 2H, H^{11} + H^{13}), 7.23 – 7.20 (m, 1H, H^{12}), 6.65 (d, *J* = 9.0 Hz, 2H, H^{17} + H^{19}), 6.58 (d, *J* = 8.5 Hz, 2H, H^{16} + H^{20}), 5.03 (br. s, 1H, H^{21}), 4.25 (br. s, *J* = 7.0 Hz, 1H, H^3), 3.92 (d, *J* = 7.0 Hz, 1H, H^8), 3.25 – 3.20 (m, 1H, H^{22}), 3.01 – 2.96 (m, 1H, H^{22}), 2.93 – 2.89 (m, 1H, H^{25}), 2.41 – 2.36 (m, 1H, H^{25}), 2.20 – 2.14 (m, 1H, H^9), 2.10 – 2.04 (m, 1H, H^9), 1.57 (s, 3H, H^2), 1.55 – 1.48 (m, 2H, H^{23}), 1.43 – 1.37 (m, 1H, H^{24}), 1.33 – 1.30 (m, 2H, H^{24} + H$^{26-29}$), 1.27 – 1.20 (m, 5H, H$^{26-29}$), 1.12 – 1.05 (m, 1H, H$^{26-29}$), 0.91 – 0.84 (m, 4H, H$^{26-30}$); **^{13}C NMR** (126 MHz, CDCl$_3$) Data for the major diastereomer *a* only: δ 171.1 (C^7), 148.6 (C^{15}), 144.0 (C^1), 142.2 (C^{18}), 127.9 (C^{11} + C^{13}), 127.6 (C^{10} + C^{14}), 126.3 (C^{12}), 116.3 (C^{17} + C^{19}), 116.1 (C^{16} + C^{20}), 67.1 (C^8), 46.3 (C^{22}), 45.8 (C^{25}), 45.2 (C^5), 37.9 (C^9), 31.8 (C$^{26-29}$), 30.2 (C$^{26-29}$), 25.7 (C^{23}), 23.7 (C^{24}), 23.6 (C$^{26-29}$), 22.7 (C$^{26-29}$), 18.7 (C^2), 14.1 (C^{30}); **HRMS** (ESI): calculated for $C_{26}H_{37}N_2O_2$ $[M+H]^+$ requires *m/z* 409.2850, found *m/z* 409.2858; **Chiral SFC:** YMC Chiral ART Cellulose-SC column (25 cm), CO$_2$:*i*-PrOH 75:25, 2 mL/min, 182 bar, r.t. Retention times: 7.4 mins (major), 9.4 mins (minor), e.r. = 90:10.

SFC analysis of the major diastereomer of racemate, prepared using rac-BINAP:

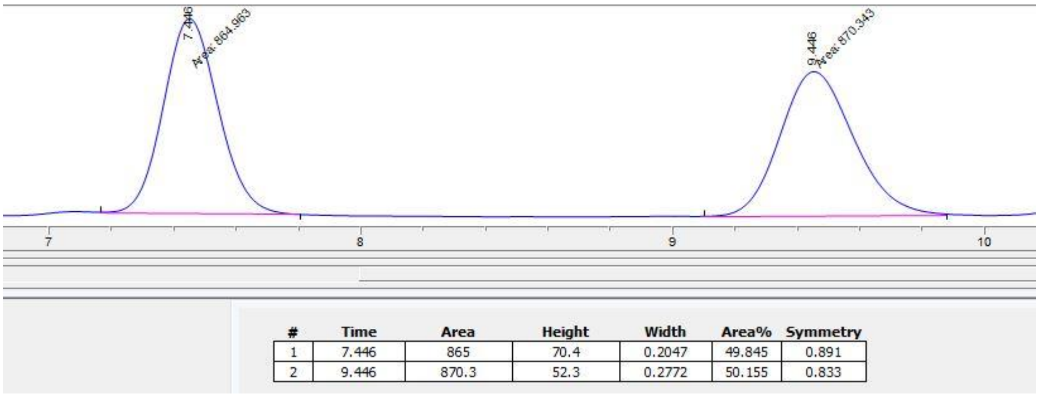

#	Time	Area	Height	Width	Area%	Symmetry
1	7.446	865	70.4	0.2047	49.845	0.891
2	9.446	870.3	52.3	0.2772	50.155	0.833

SFC analysis of the major diastereomer of enantioenriched material, prepared using (R)-DM-SEGPHOS:

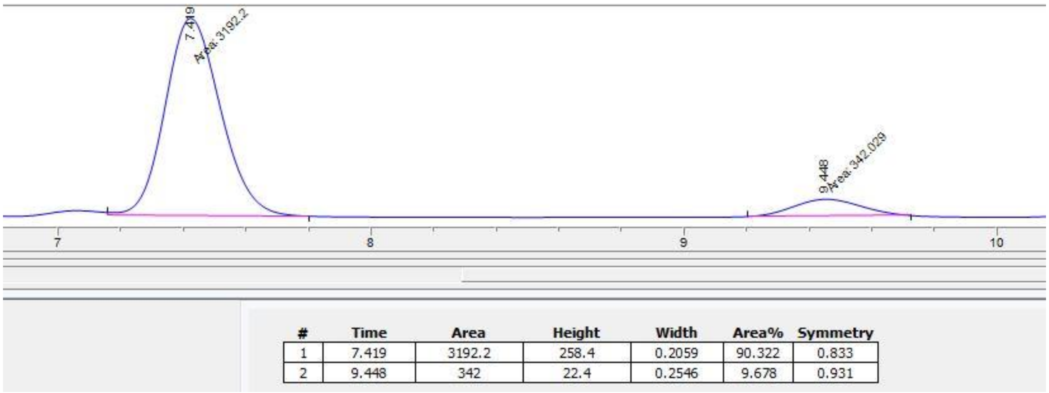

#	Time	Area	Height	Width	Area%	Symmetry
1	7.419	3192.2	258.4	0.2059	90.322	0.833
2	9.448	342	22.4	0.2546	9.678	0.931

Evaluation of the optimized protocol on a monosubstituted styrene

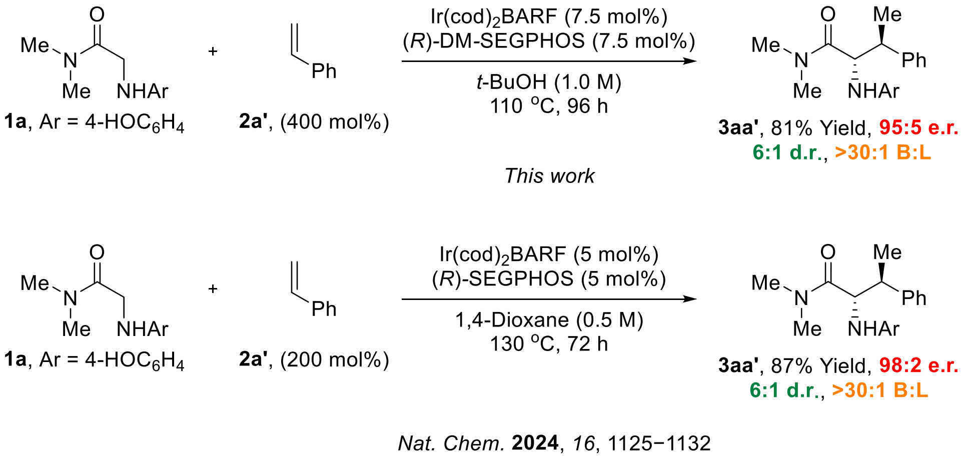

Scheme S1

Other alkenes, including methyl 4-(prop-1-en-2-yl)benzoate, 4-(prop-1-en-2-yl)aniline, 2-(4-(prop-1-en-2-yl)phenyl)isoindoline-1,3-dione, 1-(prop-1-en-2-yl)naphthalene, 2-methylhex-1-ene, ethene-1,1-diyldibenzene and (*E*)-prop-1-ene-1,2-diyldibenzene, have been evaluated and only led to the formation of the corresponding products in <5% ^{1}H NMR yields yet.

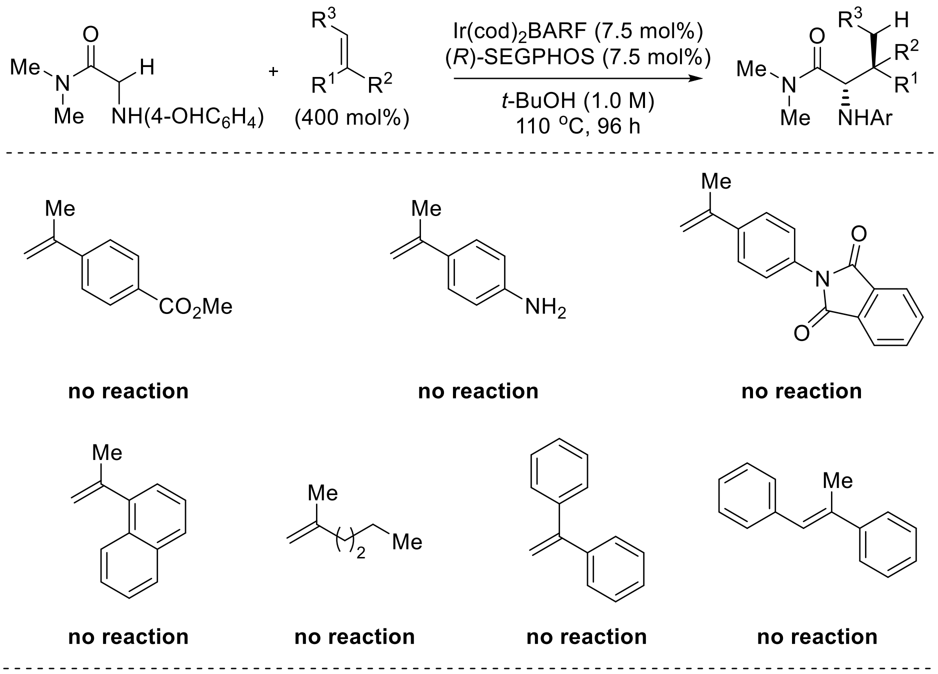

Our attempt to extend the reaction to the ester **1n** only led to 80% conversion and <5% ^{1}H NMR yield of **3na**.

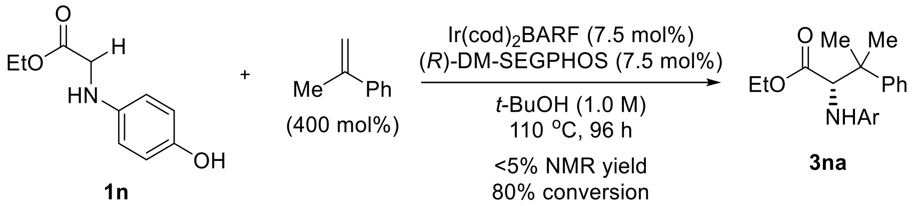

Applications and derivatizations

Synthesis of **3aa** on 2.00 mmol scale:

A Schlenk tube was charged with amide **1a** (388 mg, 2.00 mmol, 100 mol%), $[Ir(cod)_2]$BARF (191 mg, 0.15 mmol, 7.5 mol%) and (*R*)-DM-SEGPHOS (108 mg, 0.15 mmol, 7.5 mol%). The Schlenk tube was evacuated and refilled with N_2 (three cycles), then prop-1-en-2-ylbenzene **2a** (1.04 mL, 8.00 mmol, 400 mol%) was added followed by *t*-BuOH (2 mL). The tube was sealed and heated at 110 °C for 96 hours. After cooling to r.t., the solvent was removed under reduced pressure and the crude reaction mixture was purified by FCC (hexane/EtOAc 55:45) to afford the desired product **3aa** (549 mg, 88%, >30:1 B:L, e.r. = 97:3) as a pale-yellow solid. ^{1}H NMR analysis of the crude material gave >30:1 B:L.

4-Bromo-*N*-((*2S,3R*)-3-methyl-1-oxo-3-phenyl-1-(pyrrolidin-1-yl)pentan-2-yl)benzamide (5):

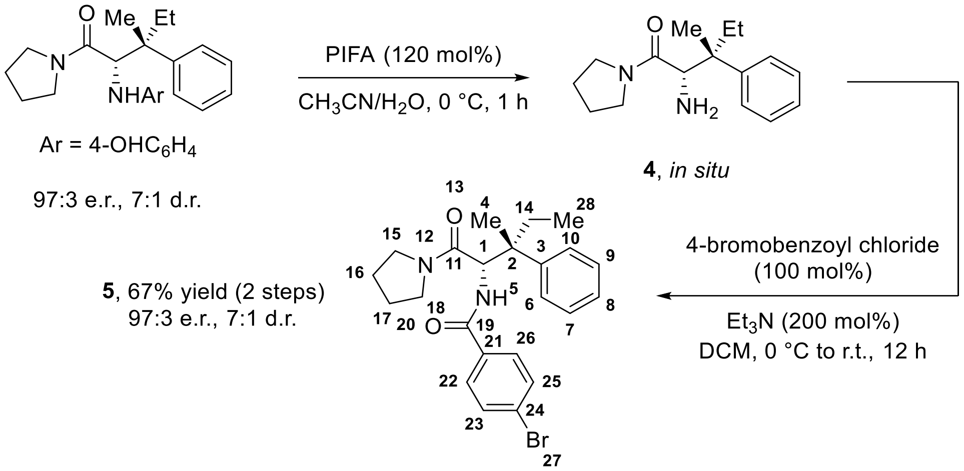

To a solution of **3iw** (70.4 mg, 0.20 mmol, 100 mol%) in CH_3CN (2 mL) and water (1 mL), was added (bis(trifluoroacetoxy)iodo)benzene (103 mg, 0.24 mmol, 120 mol%) portion-wise at 0 °C. The mixture was then stirred at 0 °C for 1 hour and the progress of the reaction was monitored by TLC. Upon completion, the mixture was cooled to 0 °C and a saturated aq. $Na_2S_2O_3$ solution (approx. 0.3 mL) was slowly added to quench excess PIFA. The mixture was extracted with CH_2Cl_2 (approx. 5 × 5 mL). The combined organic phases were dried over anhydrous $MgSO_4$. Concentration of the filtrate *in vacuo* was followed by FCC (basic Al_2O_3, CH_2Cl_2/MeOH 90:10) to afford the free amine **4**. The crude material was employed without any further purification on the next step.

4-Bromobenzoyl chloride (43.6 mg, 0.20 mmol, 100 mol%) was slowly added, portion-wise, to the solution of **4**, Et_3N (56.0 µL, 0.40 mmol, 200 mol%) and CH_2Cl_2 (2 mL) at 0 °C. The reaction was stirred at ambient temperature for 12 hours. Upon completion, the reaction mixture was diluted with CH_2Cl_2 (10 mL) and transferred to a separatory funnel. The mixture was washed with aq. 1N HCl (10 mL). The organic phase was dried over anhydrous $MgSO_4$. Concentration of the filtrate *in vacuo* was followed by FCC (EtOAc/hexane 40:60) to afford the desired product **5** (59.2 mg, 67%, d.r. 7:1, e.r. = 97:3) as a pale-yellow solid. **m.p.** = 152 – 154 °C (hexane/EtOAc); $[\alpha]_D^{25}$ = +14.0 (c = 1.0 $CHCl_3$); **IR (thin film)** ν_{max}/cm^{-1}: 3326 (s), 2877 (s), 1727 (s), 1588 (s), 1483 (s), 1260 (s), 1072 (s), 1011 (s), 847 (s), 746 (s); **^{1}H NMR** (500 MHz, $CDCl_3$) δ 7.71 (d, *J* = 8.0 Hz, 2H, H^{22} + H^{26}), 7.60 (d, *J* = 8.0 Hz, 2H, H^{23} + H^{25}), 7.47 (d, *J* = 8.0 Hz, 2H, H^6 + H^{10}), 7.36 – 7.33 (m, 2H, H^7 + H^9), 7.27 – 7.24 (m, 1H, H^8), 6.98 (d, *J* = 9.5 Hz, 1H, H^5), 5.05 (d, *J* = 9.5 Hz, 1H, H^1), 3.32 – 3.22 (m, 2H, H^{15}), 3.08 – 3.03 (m,

1H, H[18]), 2.34 – 2.23 (m, 2H, H[14] + H[18]), 1.80 – 1.73 (m, 1H, H[14]), 1.67 – 1.61 (m, 2H, H[16]), 1.57 (s, 3H, H[4]), 1.49 – 1.33 (m, 2H, H[17]), 0.75 (t, J = 7.5 Hz, 3H, H[28]); **^{13}C NMR** (126 MHz, $CDCl_3$) δ 168.8 (C[11]), 166.1 (C[19]), 142.4 (C[3]), 133.0 (C[21]), 131.8 (C[23] + C[25]), 128.8 (C[22] + C[26]), 128.1 (C[7] + C[9]), 127.8 (C[6] + C[10]), 126.7 (C[8]), 126.4 (C[24]), 59.1 (C[1]), 46.6 (C[15]), 46.5 (C[2]), 45.4 (C[18]), 30.3 (C[14]), 25.7 (C[16]), 23.8 (C[17]), 18.3 (C[4]), 8.0 (C[28]); **HRMS** (ESl): calculated for $C_{23}H_{28}BrN_2O_2$ $[M+H]^+$ requires m/z 443.1329, found m/z 443.1325; **Chiral SFC:** YMC Chiral ART Cellulose-SC column (25 cm), CO_2:*i*-PrOH 80:20 (with 0.5% TEA), 2 mL/min, 176 bar, r.t. Retention times: 3.6 mins (minor), 4.3 mins (major), e.r. = 97:3.

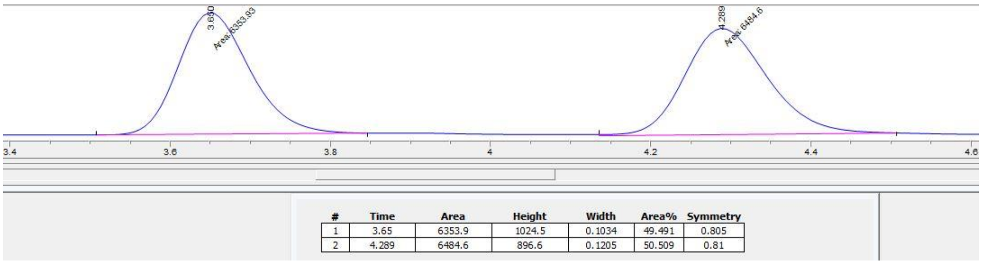

#	Time	Area	Height	Width	Area%	Symmetry
1	3.65	6353.9	1024.5	0.1034	49.491	0.805
2	4.289	6484.6	896.6	0.1205	50.509	0.81

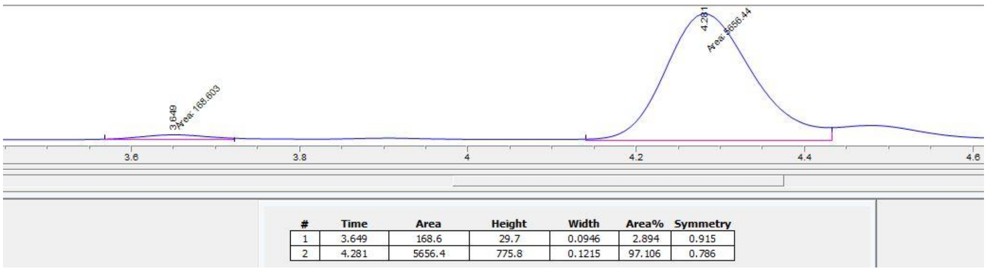

#	Time	Area	Height	Width	Area%	Symmetry
1	3.649	168.6	29.7	0.0946	2.894	0.915
2	4.281	5656.4	775.8	0.1215	97.106	0.786

The structure of compound **5** was determined by single crystal X-ray diffraction of crystals (>99:1 e.r.) grown from EtOAc/hexane. See CCDC 2408988.

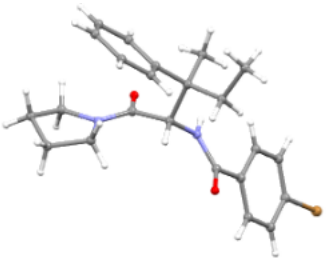

Synthesis of (*S*)-2-amino-*N*,*N*,3-trimethyl-3-phenylbutanamide 6, (*S*)-2-amino-3-methyl-3-phenylbutanoic acid hydrochloride 7, (*S*)-2-amino-3-methyl-3-phenylbutan-1-ol 8 and (*S*)-*N*-(1-hydroxy-3-methyl-3-phenylbutan-2-yl)-4-methylbenzenesulfonamide 8-1:

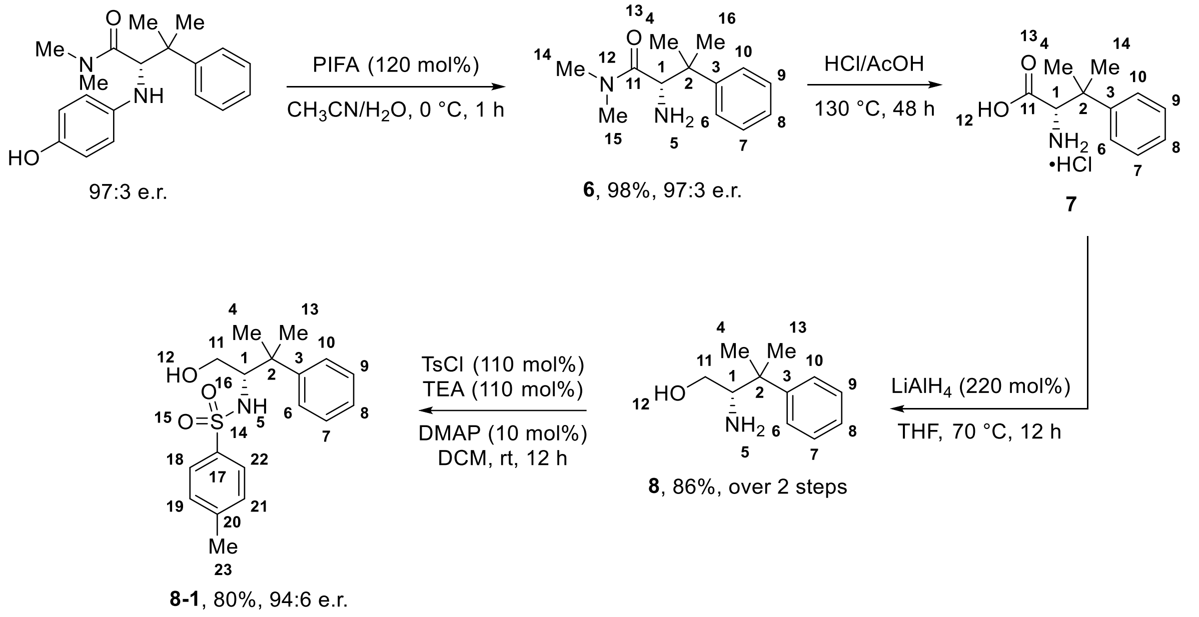

To the solution of **3aa** (250 mg, 0.80 mmol, 100 mol%) in CH_3CN (8 mL) and water (4 mL), was added (bis(trifluoroacetoxy)iodo)benzene (412 mg, 0.96 mmol, 120 mol%) portion-wise at 0 °C. The mixture was then stirred at 0 °C for 1 hour and the progress of the reaction was monitored by TLC. Upon completion, the mixture was cooled to 0 °C and a saturated aq. $Na_2S_2O_3$ solution (approx. 1 mL) was slowly added to quench excess PIFA. The mixture was extracted with CH_2Cl_2 (approx. 6 × 10 mL). The combined organic phases were dried over anhydrous $MgSO_4$. Concentration of the filtrate *in vacuo* was followed by FCC (basic Al_2O_3, CH_2Cl_2/MeOH 90:10) to afford the free amine **6** (173 mg, 98%, e.r. = 97:3) as a pale-yellow oil. $[\alpha]_D^{25}$ = +94.8 (c = 1.0 $CHCl_3$); **IR (thin film)** ν_{max}/cm^{-1}: 3362 (s), 2930 (br), 1617 (s), 1497 (s), 1363 (s), 1138 (s), 910 (s), 773 (s), 770 (s); **^{1}H NMR** (500 MHz, $CDCl_3$) δ 7.41 (d, *J* = 7.0 Hz, 2H, H^6 + H^{10}), 7.34 – 7.31 (m, 2H, H^7 + H^9), 7.24 – 7.22 (m, 1H, H^8), 3.85 (s, 1H, H^1), 2.80 (s, 3H, H^{14}), 2.49 (s, 3H, H^{15}), 1.89 (br. s, 2H, H^5), 1.50 (s, 3H, H^4), 1.42 (s, 3H, H^{16}); **^{13}C NMR** (126 MHz, $CDCl_3$) δ 173.3 (C^{11}), 146.8 (C^3), 128.1 (C^7 + C^9), 126.6 (C^6 + C^{10}), 126.4 (C^8), 58.8 (C^1), 42.5 (C^2), 37.1 (C^{14}), 35.4 (C^{15}), 24.7 (C^4), 23.1 (C^{16}); **HRMS** (ESl): calculated for $C_{13}H_{21}N_2O$ $[M+H]^+$ requires *m/z* 221.1648, found *m/z* 221.1650; **Chiral SFC:** DAICEL CHIRALCEL OD-H column (25 cm), CO_2:*i*-PrOH (with 0.5% TEA) 70:30, 2 mL/min, 191 bar, r.t. Retention times: 2.5 mins (major), 3.0 mins (minor), e.r. = 97:3.

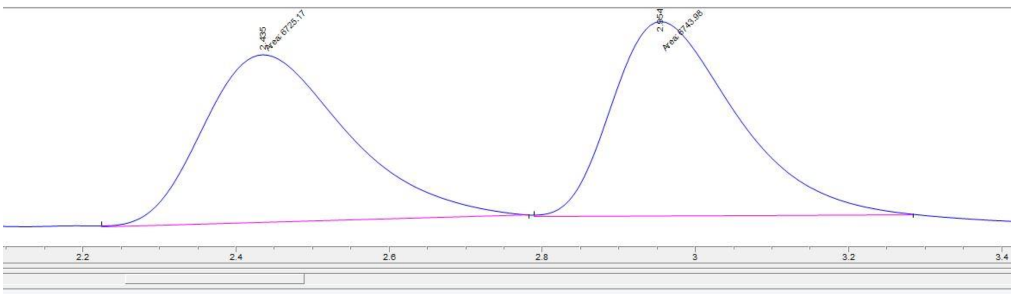

#	Time	Area	Height	Width	Area%	Symmetry
1	2.435	6725.2	513.7	0.2182	49.930	0.697
2	2.954	6744	595.7	0.1887	50.070	0.624

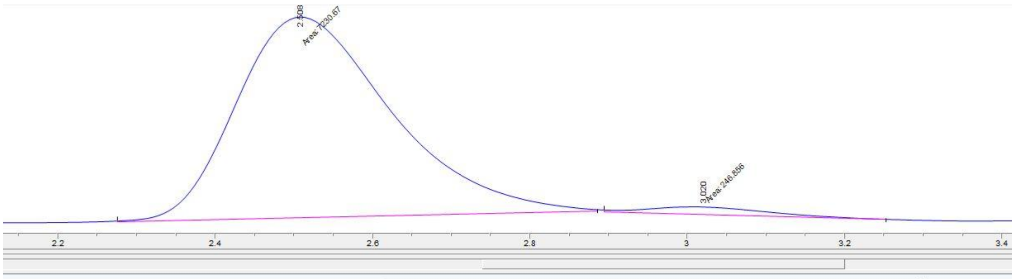

#	Time	Area	Height	Width	Area%	Symmetry
1	2.508	7230.7	525.3	0.2294	96.699	0.69
2	3.02	246.9	21.6	0.1907	3.301	0.54

To a solution of free amine **6** (44.0 mg, 0.20 mmol, 100 mol%) in AcOH (0.5 mL), was added concentrated hydrochloric acid (1 mL) dropwise over 2 minutes at r.t. The mixture was then stirred at 130 °C for 48 hours and the progress of the reaction was monitored by TLC. After cooling to r.t., the solvents were removed *in vacuo*, the solid was then washed with EtOAc and dried under vacuum to afford **7** as a brown solid. The solid was used in the next step without further purification. **IR (thin film)** ν_{max}/cm^{-1}: 3388 (br), 2976 (s), 1721 (s), 1644 (s), 1450 (s), 1475 (s), 1244 (s), 716 (s); **^{1}H NMR** (500 MHz, D_2O) δ 7.44 (d, *J* = 8.0 Hz, 2H, H^6 + H^{10}), 7.41 – 7.38 (m, 2H, H^7 + H^9), 7.32 – 7.29 (m, 1H, H^8), 4.20 (s, 1H, H^1), 1.47 (s, 3H, H^4), 1.41 (s, 3H, H^{14}); **^{13}C NMR** (126 MHz, D_2O) δ 170.8 (C^{11}), 143.2 (C^3), 129.2 (C^7 + C^9), 127.8 (C^8), 126.4 (C^6 + C^{10}), 62.3 (C^1), 34.5 (C^2), 26.3 (C^4), 22.2 (C^{14}). The crude material was employed without any further purification on the next step. The enantioselectivity was confirmed after subsequent Ts-protection.

To the above solid, a solution of $LiAlH_4$ in THF (1.0 M, 0.44 mL, 0.44 mmol, 220 mol%) was then added dropwise over 2 minutes under N_2 at r.t. The mixture was then stirred at 70 °C for 12 hours and the progress of the reaction was monitored by TLC. Upon completion, the mixture was cooled to 0 °C and 3 drops of water were slowly added. This was followed by addition of aq. 4M NaOH solution (3 drops) and water (3 drops). The resulting suspension was then warmed to r.t. and stirred for 15 minutes before anhydrous Na_2SO_4 was added. The mixture was stirred for additional 15 minutes before filtration. The concentration of the filtrate *in vacuo*

to afford the desired product **8** (30.8 mg, 86%, over 2 steps) as a colorless oil. **IR (thin film)** ν_{max}/cm^{-1}: 3348 (br), 2977 (s), 2942 (s), 1568 (s), 1492 (s), 1455 (s), 1038 (s), 770 (s), 705 (s); **^{1}H NMR** (500 MHz, $CDCl_3$) δ 7.38 – 7.33 (m, 4H, H^6 + H^7 + H^9 + H^{10}), 7.26 – 7.22 (m, 1H, H^8), 3.62 – 3.60 (m, 1H, H^{11}), 3.25 – 3.21 (m, 1H, H^{11}), 3.05 – 3.02 (m, 1H, H^1), 2.43 (br. s, 3H, H^5 + H^{12}), 1.35 (s, 6H, H^4 + H^{13}); **^{13}C NMR** (126 MHz, $CDCl_3$) δ 146.9 (C^3), 128.4 (C^7 + C^9), 126.3 (C^8), 126.1 (C^6 + C^{10}), 62.4 (C^1 or C^{11}), 62.2 (C^1 or C^{11}), 40.7 (C^2), 24.7 (C^4), 24.1 (C^{13}); **HRMS** (ESI): calculated for $C_{11}H_{18}NO$ $[M+H]^+$ requires m/z 180.1383, found m/z 180.1386. The crude material was employed without any further purification on the next step. The enantiopurity was measured after subsequent Ts-protection.

4-Methylbenzenesulfonyl chloride (21.0 mg, 0.11 mmol, 110 mol%) was slowly added to a solution of **8** (17.9 mg, 0.10 mmol, 100 mol%), Et_3N (15.0 µL, 0.11 mmol, 110 mol%), DMAP (1.3 mg, 11.0 µmol, 10 mol%) and CH_2Cl_2 (1 mL) at r.t. The reaction was stirred at ambient temperature for 12 hours. Upon completion, the reaction mixture was diluted with CH_2Cl_2 (5.mL) and transferred to a separatory funnel. The mixture was washed with aq. 1N HCl (10 mL). The organic phase was dried over anhydrous $MgSO_4$. Concentration of the filtrate *in vacuo* was followed by FCC (EtOAc/hexane 50:50) to afford the desired product **8-1** (26.7 mg, 80%, e.r. = 94:6) as a colorless oil; **IR (thin film)** ν_{max}/cm^{-1}: 3437 (br), 3161 (br), 2977 (s), 1597 (s), 1474 (s), 1447 (s), 1303 (s), 1081 (s), 699 (s), 675 (s); **^{1}H NMR** (500 MHz, $CDCl_3$) δ 7.70 (d, *J* = 8.5 Hz, 2H, H^{18} + H^{22}), 7.29 – 7.20 (m, 7H, H^{6-10} + H^{19} + H^{21}), 4.98 (d, *J* = 8.5 Hz, 1H, H^5), 3.45 – 3.37 (m, 3H, H^1 + H^{11}), 2.44 (s, 3H, H^{23}), 1.31 (s, 3H, H^4), 1.27 (s, 3H, H^4); **^{13}C NMR** (126 MHz, $CDCl_3$) δ 145.6 (C^3), 143.5 (C^{17}), 137.3 (C^{20}), 129.7 (C^{19} + C^{21}), 128.6 (C^7 + C^9), 127.2 (C^{18} + C^{22}), 126.6 (C^8), 126.0 (C^6 + C^{10}), 63.9 (C^1), 62.3 (C^{11}), 41.2 (C^2), 26.5 (C^4), 24.5 (C^{13}), 21.6 (C^{23}); **HRMS** (ESI): calculated for $C_{18}H_{23}NO_3SNa$ $[M+Na]^+$ requires m/z 356.1291, found m/z 356.1301; **Chiral SFC:** DAICEL CHIRALCEL OD-H column (25 cm), CO_2:*i*-PrOH (with 0.5% TEA) 90:10, 2 mL/min, 145 bar, r.t. Retention times: 19.4 mins (minor), 20.5 mins (major), e.r. = 94:6.

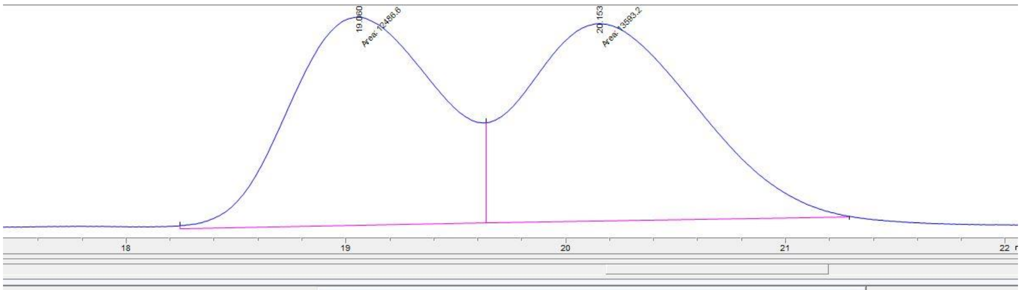

#	Time	Area	Height	Width	Area%	Symmetry
1	19.06	12486.6	262.8	0.7919	47.878	0.86
2	20.153	13593.2	249.4	0.9083	52.122	0.771

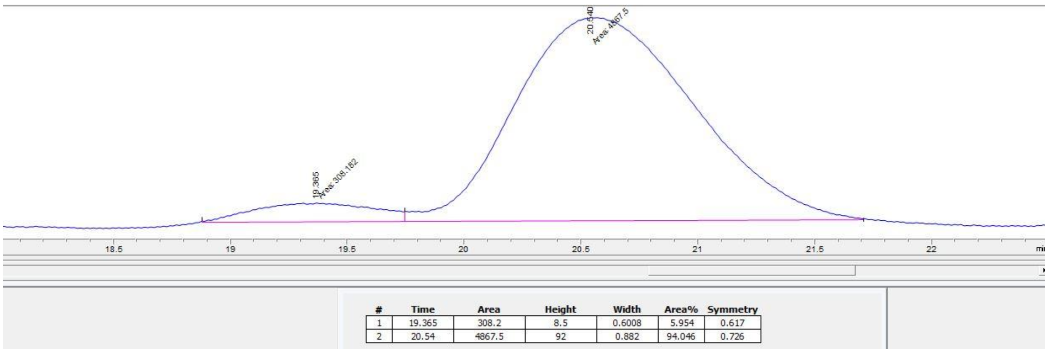

#	Time	Area	Height	Width	Area%	Symmetry
1	19.365	308.2	8.5	0.6008	5.954	0.617
2	20.54	4867.5	92	0.882	94.046	0.726

Synthetic approach to 7 from the literature

(*S*)-2-Amino-3-methyl-3-phenylbutanoic acid can be synthesized in 7 steps according to reported procedures versus the three step synthesis described here.[18,19]

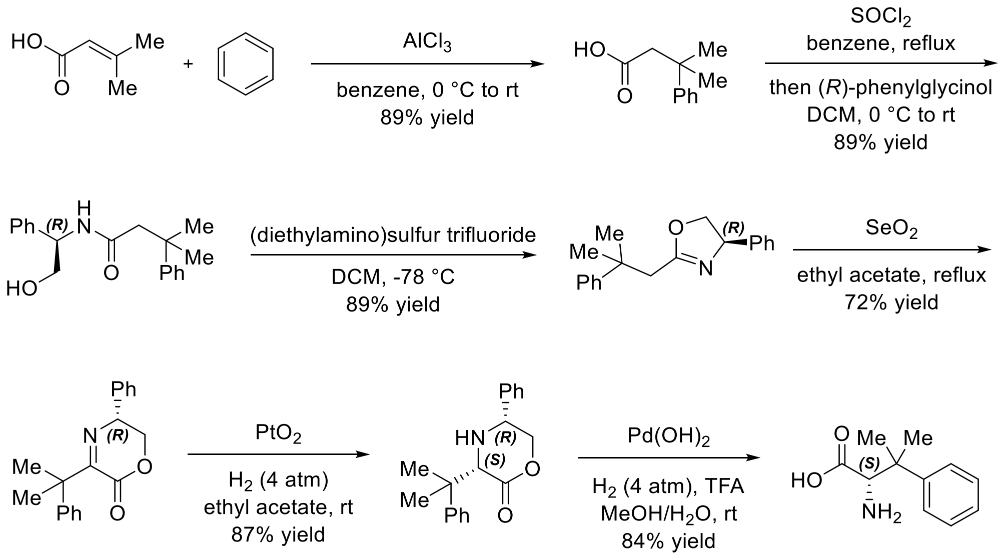

Streamlined access to the precursor of a chiral N,O-ligand (*S*)-2-amino-3-methyl-3-(naphthalen-2-yl)-1-(pyrrolidin-1-yl)butan-1-one 9:

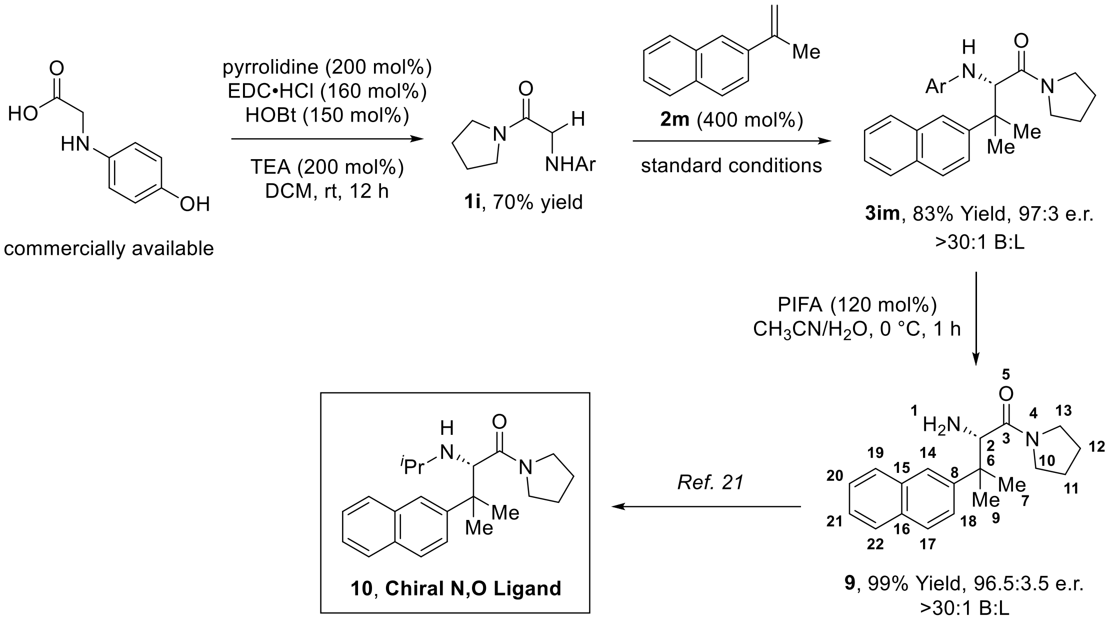

Amide **1i** was synthesized according to a modified procedure.[20]

To an oven-dried 100 mL round bottom flask, (4-hydroxyphenyl)glycine (1.67 g, 10.0 mmol, 100 mol%) and pyrrolidine (1.7 mL, 20.0 mmol, 200 mol%) were dissolved in 30 mL DCM, EDC•HCl (3.15 g, 16.0 mmol, 160 mol%), HOBt (2.03 g, 15.0 mmol, 150 mol%) and TEA (2.8 mL, 20.0 mmol, 200 mol%) were added. After the addition was complete, the reaction mixture was stirred for 22 hours at r.t. and the progress of the reaction was monitored by TLC. Upon completion, water (approx. 50 mL) was added to the mixture. The mixture was extracted with DCM (approx. 3 × 30 mL) and the combined organic layers were dried over Na_2SO_4 and concentrated *in vacuo*, which was purified by column chromatography (DCM/MeOH, 20:1) affording the title amide **1i** (1.54 g, 70%) as a pale yellow solid.

To the solution of **3im** (194 mg, 0.50 mmol, 100 mol%) in CH_3CN (5 mL) and water (2.5 mL), (bis(trifluoroacetoxy)iodo)benzene (258 mg, 0.60 mmol, 120 mol%) was added portion-wise at 0 °C. The mixture was then stirred at 0 °C for 1 hour and the progress of the reaction was monitored by TLC. Upon completion, the mixture was cooled to 0 °C and a saturated aq. $Na_2S_2O_3$ solution (approx. 0.7 mL) was slowly added to quench excess PIFA. The mixture was extracted with CH_2Cl_2 (approx. 6 × 10 mL). The combined organic phases were dried over anhydrous $MgSO_4$. The concentration of the filtrate *in vacuo* was followed by FCC (basic Al_2O_3, CH_2Cl_2/MeOH 95:5) to afford the free amine **9** (147 mg, 99%, e.r. = 96.5:3.5) as a pale-yellow solid. **m.p.** = 117 – 119 °C (hexane/EtOAc); $[\alpha]_D^{25}$ = +83.2 (c = 1.0 $CHCl_3$); **IR (thin**

film) ν_{max}/cm^{-1}: 3340 (br), 2971 (s), 1611 (s), 1448 (s), 823 (s), 753 (s), 733 (s); **^{1}H NMR** (500 MHz, $CDCl_3$) δ 7.86 – 7.79 (m, 4H, ArH), 7.59 – 7.57 (m, 1H, H^{14}), 7.50 – 7.45 (m, 2H, ArH), 3.72 (s, 1H, H^2), 3.46 – 3.40 (m, 1H, H^{10}), 3.27 – 3.20 (m, 2H, H^{10} + H^{13}), 2.54 – 2.49 (m, 1H, H^{13}), 2.12 (br. s, 2H, H^1), 1.67 – 1.58 (m, 5H, H^7 + H^{11}), 1.55 (s, 3H, H^9), 1.48 – 1.41 (m, 1H, H^{12}), 1.29 – 1.24 (m, 1H, H^{12}); **^{13}C NMR** (126 MHz, $CDCl_3$) δ 171.6 (C^3), 144.1 (C^8), 133.1 (C^{16}), 132.0 (C^{15}), 128.0 (ArC), 127.4 (ArC), 127.3 (ArC), 126.1 (ArC), 125.8 (ArC), 125.4 (ArC), 125.1 (ArC), 61.4 (C^2), 46.6 (C^{10}), 45.7 (C^{13}), 42.6 (C^6), 25.8 (C^{11}), 24.7 (C^7), 23.9 (C^{12}), 23.4 (C^9); **HRMS** (ESI): calculated for $C_{19}H_{25}N_2O$ $[M+H]^+$ requires *m/z* 297.1961, found *m/z* 297.1961; **Chiral SFC:** DAICEL CHIRALCEL OD-H column (25 cm), CO_2:*i*-PrOH (with 0.5% TEA) 70:30, 2 mL/min, 194 bar, r.t. Retention times: 3.1 mins (major), 3.8 mins (minor), e.r. = 96.5:3.5.

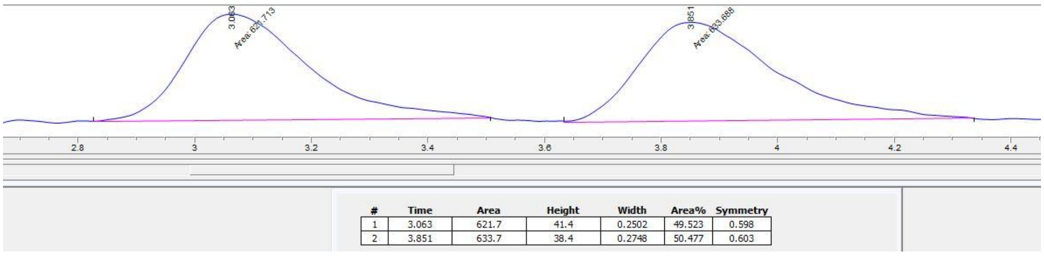

#	Time	Area	Height	Width	Area%	Symmetry
1	3.063	621.7	41.4	0.2502	49.523	0.598
2	3.851	633.7	38.4	0.2748	50.477	0.603

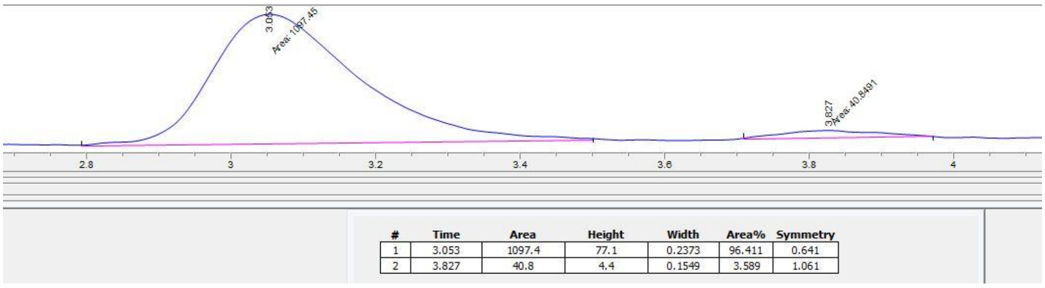

#	Time	Area	Height	Width	Area%	Symmetry
1	3.053	1097.4	77.1	0.2373	96.411	0.641
2	3.827	40.8	4.4	0.1549	3.589	1.061

Synthetic approach to chiral ligand 10 from the literature

(*R*)-2-(Isopropylamino)-3-methyl-3-(naphthalen-2-yl)-1-(pyrrolidin-1-yl)butan-1-one can be synthesized in 9 steps according to reported procedures versus the 4 step synthesis described here.[21,22]

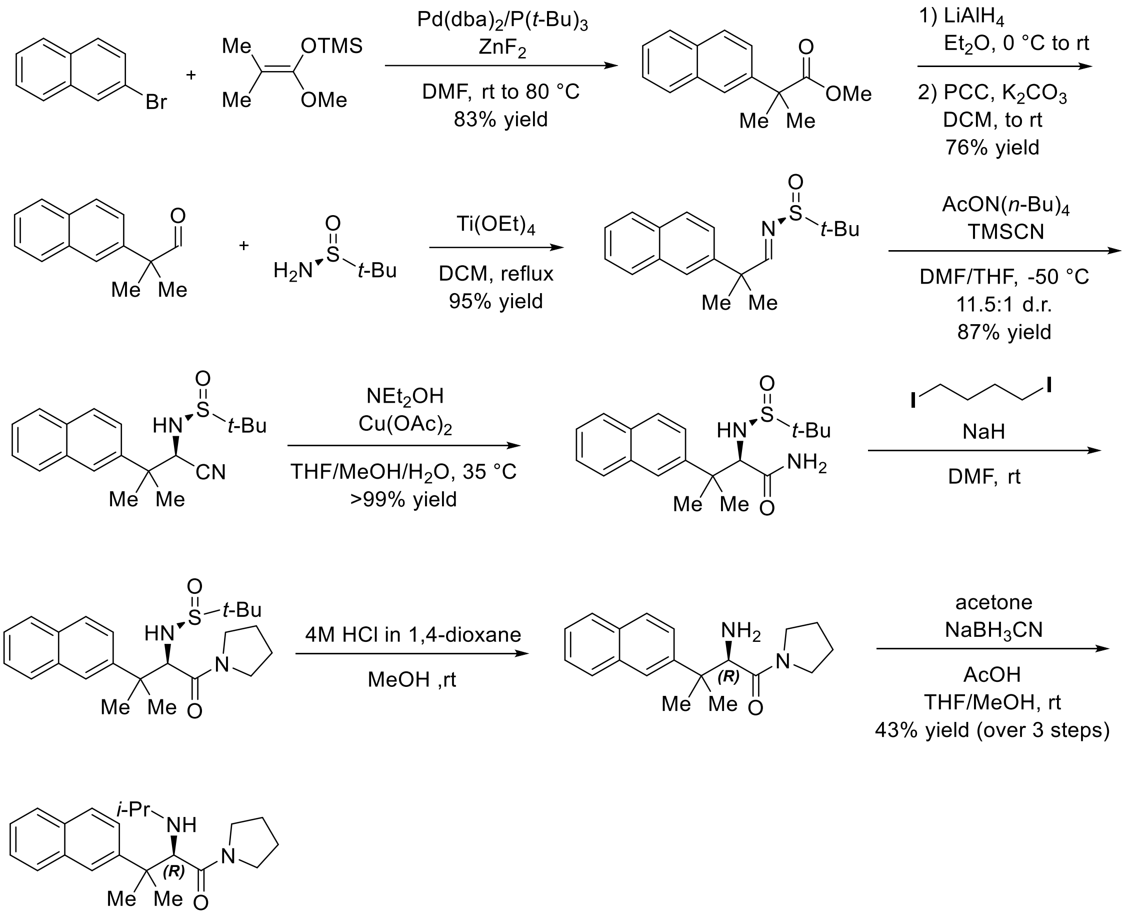
Br
+
Me
OTMS
Me
OMe
Pd(dba)2/P(t-Bu)3
ZnF2
DMF, rt to 80 °C
83% yield
O
OMe
Me Me
1) LiAlH4
Et2O, 0 °C to rt
2) PCC, K2CO3
DCM, to rt
76% yield
O
Me Me
+
O
H2N
S
t-Bu
Ti(OEt)4
DCM, reflux
95% yield
O
N
S
t-Bu
Me Me
AcON(n-Bu)4
TMSCN
DMF/THF, -50 °C
11.5:1 d.r.
87% yield
O
HN
S
t-Bu
CN
Me Me
NEt2OH
Cu(OAc)2
THF/MeOH/H2O, 35 °C
>99% yield
O
HN
S
t-Bu
NH2
Me Me
O
I
I
NaH
DMF, rt
O
S
t-Bu
HN
N
Me Me
O
4M HCl in 1,4-dioxane
MeOH ,rt
NH2
(R)
N
Me Me
O
acetone
NaBH3CN
AcOH
THF/MeOH, rt
43% yield (over 3 steps)
i-Pr
NH
(R)
N
Me Me
O

Mechanistic studies

Figure S1: Kinetic experiments

Procedure:

Two microwave vials in a nitrogen-filled glovebox were charged with amide **1d** (71.3 mg, 0.40 mmol, 100 mol%), [Ir(cod)$_2$]BARF (Vial 1: 50.9 mg, 0.04 mmol, 10.0 mol%; Vial 2: 25.4 mg, 0.02 mmol, 5.0 mol%), (*R*)-DM-SEGPHOS (Vial 1: 28.9 mg, 0.04 mmol, 10.0 mol%; Vial 2: 14.5 mg, 0.02 mmol, 5.0 mol%) and 1,4-dimethoxybenzene (internal standard, 55.3 mg, 0.40 mmol), respectively. Then, prop-1-en-2-ylbenzene **2a** (208 µL, 1.60 mmol, 400 mol%) and 3-ethyl-3-pentanol (0.4 mL) were added. These vials were capped with removable caps and placed into a preheated heating block at 110 °C inside the glovebox. Aliquots were taken at regular intervals (≈ 1-5 µL) using a spinal needle, removed from the glovebox, quenched by addition of MeCN (1 mL) and analysed by HPLC. The concentration of product **3da** was calculated by comparison to the internal standard.

Method:

The order in catalyst has been determined applying the Variable Time Normalization Graphical Analysis (VTNGA).[23] Different concentrations of catalyst (10.0 mol% and 5.0 mol%, respectively) were used and the concentration of substrate **3da** was plotted against t[Ir]^n where n is the partial order in iridium catalyst, and t is the reaction time. The order in iridium catalyst is that value of n that causes the curves to overlay. The overlap between the temporal reaction profiles with catalyst loadings of 10.0 mol% and 5.0 mol% indicates that the order in iridium catalyst is approximately 1 using (*R*)-DM-SEGPHOS as a ligand.

		t (h)	tcat	[P] (M)	[cat] (M)	t (h)	tcat	[P] (M)	[cat] (M)
		0.083333	0.00	0.000	0.05	0.083333	0.00	0.001	0.1
order in cat	0.5	0.5	0.09	0.001	0.05	0.5	0.13	0.001	0.1
		1	0.20	0.004	0.05	1	0.29	0.006	0.1
		2	0.43	0.016	0.05	2	0.61	0.031	0.1
		4	0.88	0.036	0.05	4	1.24	0.063	0.1
		6	1.32	0.054	0.05	6	1.87	0.093	0.1
		8	1.77	0.070	0.05	8	2.50	0.131	0.1
		10	2.22	0.087	0.05	10	3.14	0.158	0.1
		23.2	5.17	0.192	0.05	23.2	7.31	0.318	0.1
		24.8	5.53	0.199	0.05	24.8	7.82	0.342	0.1
		27.5	6.13	0.220937	0.05	27.5	8.67	0.35019	0.1
		29.5	6.58	0.238998	0.05	29.5	9.30	0.384286	0.1
		31.6	7.05	0.249983	0.05	31.6	9.97	0.387013	0.1
		47.33	10.56	0.344805	0.05	47.33	14.94	0.49102	0.1
		49.2	10.98		0.05	49.2	15.53	0.519868	0.1
		52.5	11.72	0.434308	0.05	52.5	16.58	0.521669	0.1
		54.5	12.17	0.392041	0.05	54.5	17.21	0.520819	0.1
		71	15.86	0.444286	0.05	71	22.43	0.601458	0.1
		72.5	16.19	0.449875	0.05	72.5	22.90	0.608183	0.1

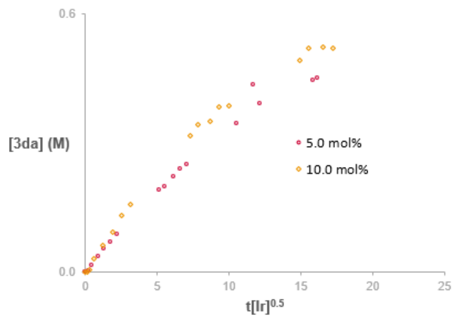

		t (h)	tcat	[P] (M)	[cat] (M)	t (h)	tcat	[P] (M)	[cat] (M)
		0.083333	0.00	0.000	0.05	0.083333	0.00	0.001	0.1
order in cat	1	0.5	0.02	0.001	0.05	0.5	0.04	0.001	0.1
		1	0.05	0.004	0.05	1	0.09	0.006	0.1
		2	0.10	0.016	0.05	2	0.19	0.031	0.1
		4	0.20	0.036	0.05	4	0.39	0.063	0.1
		6	0.30	0.054	0.05	6	0.59	0.093	0.1
		8	0.40	0.070	0.05	8	0.79	0.131	0.1
		10	0.50	0.087	0.05	10	0.99	0.158	0.1
		23.2	1.16	0.192	0.05	23.2	2.31	0.318	0.1
		24.8	1.24	0.199	0.05	24.8	2.47	0.342	0.1
		27.5	1.37	0.220937	0.05	27.5	2.74	0.35019	0.1
		29.5	1.47	0.238998	0.05	29.5	2.94	0.384286	0.1
		31.6	1.58	0.249983	0.05	31.6	3.15	0.387013	0.1
		47.33	2.36	0.344805	0.05	47.33	4.72	0.49102	0.1
		49.2	2.46		0.05	49.2	4.91	0.519868	0.1
		52.5	2.62	0.434308	0.05	52.5	5.24	0.521669	0.1
		54.5	2.72	0.392041	0.05	54.5	5.44	0.520819	0.1
		71	3.55	0.444286	0.05	71	7.09	0.601458	0.1
		72.5	3.62	0.449875	0.05	72.5	7.24	0.608183	0.1

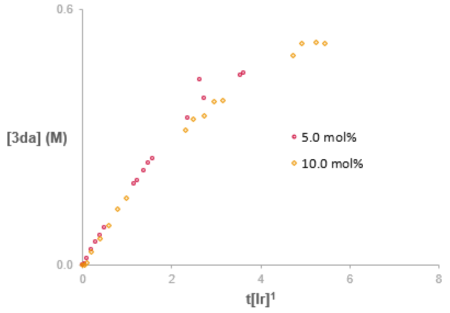

		t (h)	tcat	[P] (M)	[cat] (M)	t (h)	tcat	[P] (M)	[cat] (M)
		0.083333	0.00	0.000	0.05	0.083333	0.00	0.001	0.1
order in cat	2	0.5	0.00	0.001	0.05	0.5	0.00	0.001	0.1
		1	0.00	0.004	0.05	1	0.01	0.006	0.1
		2	0.00	0.016	0.05	2	0.02	0.031	0.1
		4	0.01	0.036	0.05	4	0.04	0.063	0.1
		6	0.01	0.054	0.05	6	0.06	0.093	0.1
		8	0.02	0.070	0.05	8	0.08	0.131	0.1
		10	0.02	0.087	0.05	10	0.10	0.158	0.1
		23.2	0.06	0.192	0.05	23.2	0.23	0.318	0.1
		24.8	0.06	0.199	0.05	24.8	0.25	0.342	0.1
		27.5	0.07	0.220937	0.05	27.5	0.27	0.35019	0.1
		29.5	0.07	0.238998	0.05	29.5	0.29	0.384286	0.1
		31.6	0.08	0.249983	0.05	31.6	0.32	0.387013	0.1
		47.33	0.12	0.344805	0.05	47.33	0.47	0.49102	0.1
		49.2	0.12		0.05	49.2	0.49	0.519868	0.1
		52.5	0.13	0.434308	0.05	52.5	0.52	0.521669	0.1
		54.5	0.14	0.392041	0.05	54.5	0.54	0.520819	0.1
		71	0.18	0.444286	0.05	71	0.71	0.601458	0.1
		72.5	0.18	0.449875	0.05	72.5	0.72	0.608183	0.1

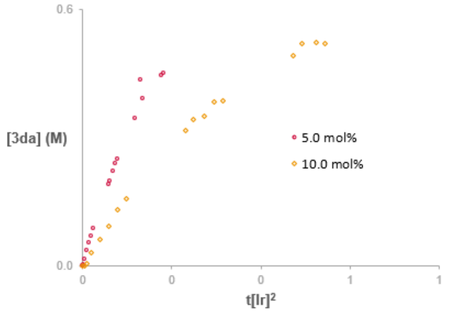

References

1. Tsuchikama, K.; Kasagawa, M.; Endo, K.; Shibata, T. Cationic Ir(I)-Catalyzed sp^3 C−H Bond Alkenylation of Amides with Alkynes. *Org. Lett.* **2009**, *11*, 1821−1823.
2. Dervisi, A.; Carcedo, C.; Ooi, L.-L. Chiral Diphosphine Ddppm-Iridium Complexes: Effective Asymmetric Imine Hydrogenations at Ambient Pressures. *Adv. Synth. Catal.* **2006**, *348*, 175−183.
3. Hong, F. L.; Aldhous, T. P.; Kemmitt, P. D.; Bower, J. F. A Directed Enolization Strategy Enables the Byproduct Free Construction of Contiguous Stereocenters en route to Complex Amino Acids. *Nat. Chem.* **2024**, *16*, 1125−1132.
4. Crapster, J. A.; Guzei, I. A.; Blackwell, H. E. A Peptoid Ribbon Secondary Structure. *Angew. Chem., Int. Ed.* **2013**, *52*, 5079−5084.
5. Chen, Z.; Yan, Q.; Liu, Z.; Xu, Y.; Zhang, Y. Copper-Mediated Synthesis of 1,2,3-Triazoles from *N*-Tosylhydrazones and Anilines. *Angew. Chem., Int. Ed.* **2013**, *52*, 13324−13328.
6. Grélaud, S.; Cooper, P.; Feron, L. J.; Bower, J. F. Branch-Selective and Enantioselective Iridium-Catalyzed Alkene Hydroarylation via Anilide-Directed C-H Oxidative Addition. *J. Am. Chem. Soc.* **2018**, *140*, 9351−9356.
7. Yamamoto, T.; Morita, T.; Takagi, J.; Yamakawa, T. $NiCl_2(PMe_3)_2$-Catalyzed Borylation of Chloroarenes. *Org. Lett.* **2011**, *13*, 5766−5769.
8. Pratsch, G.; Overman, L. E. Synthesis of 2,5-Diaryl-1,5-dienesfrom Allylic Bromides Using Visible-Light Photoredox Catalysis. *J. Org. Chem.* **2015**, *80*, 11388−11397.
9. Gulledge, Z, Z.; Pinson, C. C.; Stovall, A. M.; Dzeagu, F. O.; and Carrick, J. D. Chemoselective, Osmium-free, Dihydroxylation/Oxidative Cleavage of Heteroaryl Isoprenes by a Contemporary Malaprade Reaction. *Org. Biomol. Chem.* **2022**, *20*, 7916−7922.
10. Law, J. A.; Bartfield, N. M.; Frederich, J. H. Site-Specific Alkene Hydromethylation via Protonolysis of Titanacyclobutanes. *Angew. Chem., Int. Ed.* **2021**, *60*, 14360−14364.
11. Tang, H. J.; Zhang, B.; Xue, F.; Feng, C. Visible-Light-Induced Meerwein Fluoroarylation of Styrenes. *Org. Lett.* **2021**, *23*, 4040−4044.
12. Iron-Catalyzed Acyloxyalkylation of Styrenes Using Hypervalent Iodine Reagents. Wang, Z.; Kanai, M.; Kuninobu, Y. *Org. Lett.* **2017**, *19*, 2398−2401.

13. Xiong, B.; Zeng, X.; Geng, S.; Chen, S.; He, Y.; Feng, Z. Thiyl Radical Promoted Chemo- and Regioselective Oxidation of C=C Bonds Using Molecular Oxygen via Iron Catalysis. *Green Chem.* **2018**, *20*, 4521−4527.
14. Walker, J. C. L.; Oestreich, M. Regioselective Transfer Hydrodeuteration of Alkenes with a Hydrogen Deuteride Surrogate Using $B(C_6F_5)_3$ Catalysis. *Org. Lett.* **2018**, *20*, 6411−6414.
15. Matsuda, T.; Yuihara, I.; Kondo, K. Rhodium(I)-Catalysed Skeletal Reorganisation of Benzofused Spiro[3.3]Heptanes via Consecutive Carbon-Carbon Bond Cleavage. *Org. Biomol. Chem.* **2016**, *14*, 7024−7027.
16. Zhang, S.; Bedi, D.; Cheng, L.; Unruh, D. K.; Li, G.; Findlater, M. Cobalt(II)-Catalyzed Stereoselective Olefin Isomerization: Facile Access to Acyclic Trisubstituted Alkenes. *J. Am. Chem. Soc.* **2020**, *142*, 8910−8917.
17. Czyz, M. L.; Taylor, M. S.; Horngren, T. H.; Polyzos, A. Reductive Activation and Hydrofunctionalization of Olefins by Multiphoton Tandem Photoredox Catalysis. *ACS Catal.* **2021**, *11*, 5472−5480.
18. Crowley, D. C.; Lynch, D.; Maguire, A. R. Copper-Mediated, Heterogeneous, Enantioselective Intramolecular Buchner Reactions of α-Diazoketones Using Continuous Flow Processing. *J. Org. Chem.* **2018**, *83*, 3794−3805.
19. Liu, C.; Molinski T. F. Preparation of a-Amino Acids by Oxidative Oxazoline–Oxazinone Rearrangement–Hydrogenation (OOOH). Scope and Limitations. *Chem. Asian J.* **2011**, *6*, 2022−2027.
20. Tian, H.; Xu, W.; Liu, Y.; Wang, Q. Unnatural α-Amino Acid Synthesized through α-Alkylation of Glycine Derivatives by Diacyl Peroxides. *Org. Lett.* **2020**, *22*, 5005−5008.
21. Ishihara, K.; Nishimura, K.; Yamakawa, K. Enantio- and Site-selective α-Fluorination of *N*-Acyl 3,5-dimethylpyrazoles Catalyzed by Chiral π−Cu^{II} Complexes. *Angew. Chem., Int. Ed.* **2020**, *59*, 17641−17647.
22. Cai, X.; Keshavarz, A.; Omaque, J. D.; Stokes, B. J. Brønsted Acid-Catalyzed Intramolecular Hydroarylationof β-Benzylstyrenes. *Org. Lett.* **2017**, *19*, 2626−2629.
23. Burés, J. A Simple Graphical Method to Determine the Order in Catalyst. *Angew. Chem., Int. Ed.* **2016**, *55*, 2028−2031.

NMR Spectra

2-((3-Fluoro-4-hydroxyphenyl)amino)-*N*,*N*-dimethylacetamide (1g)

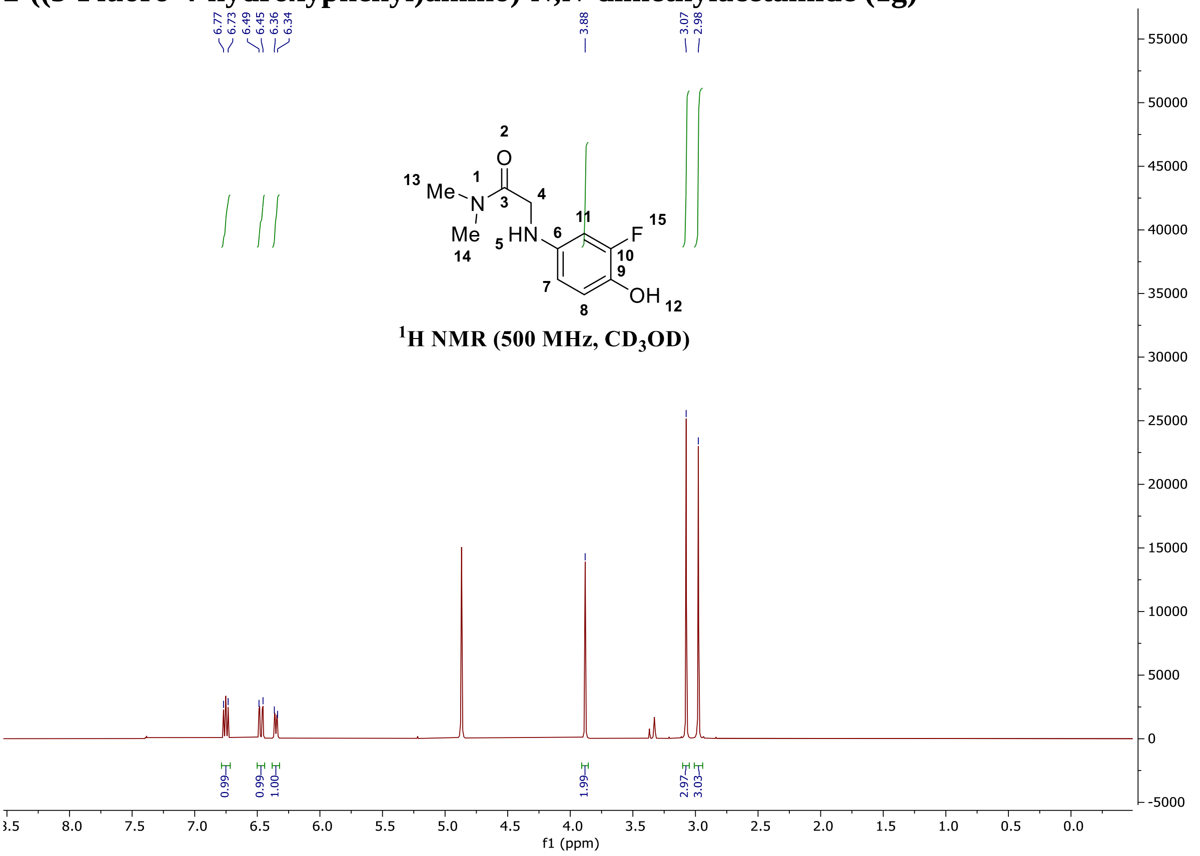

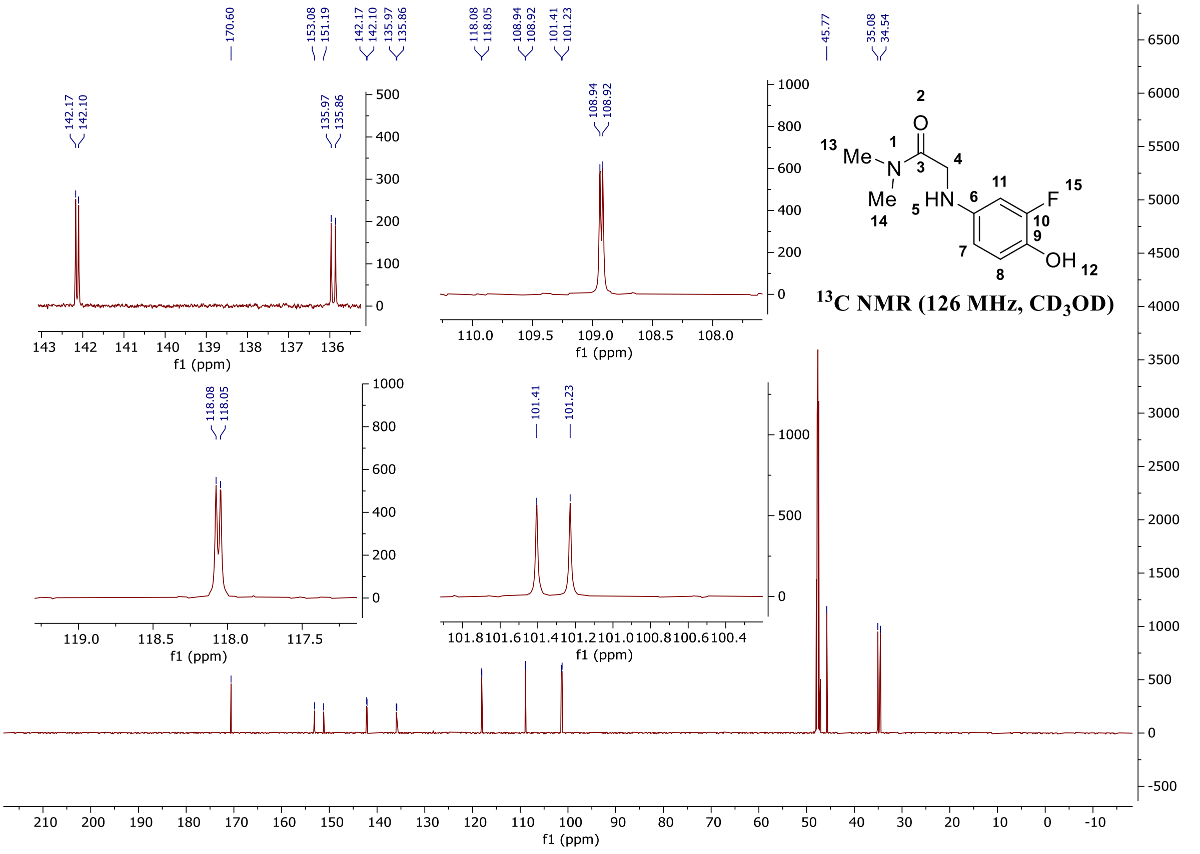

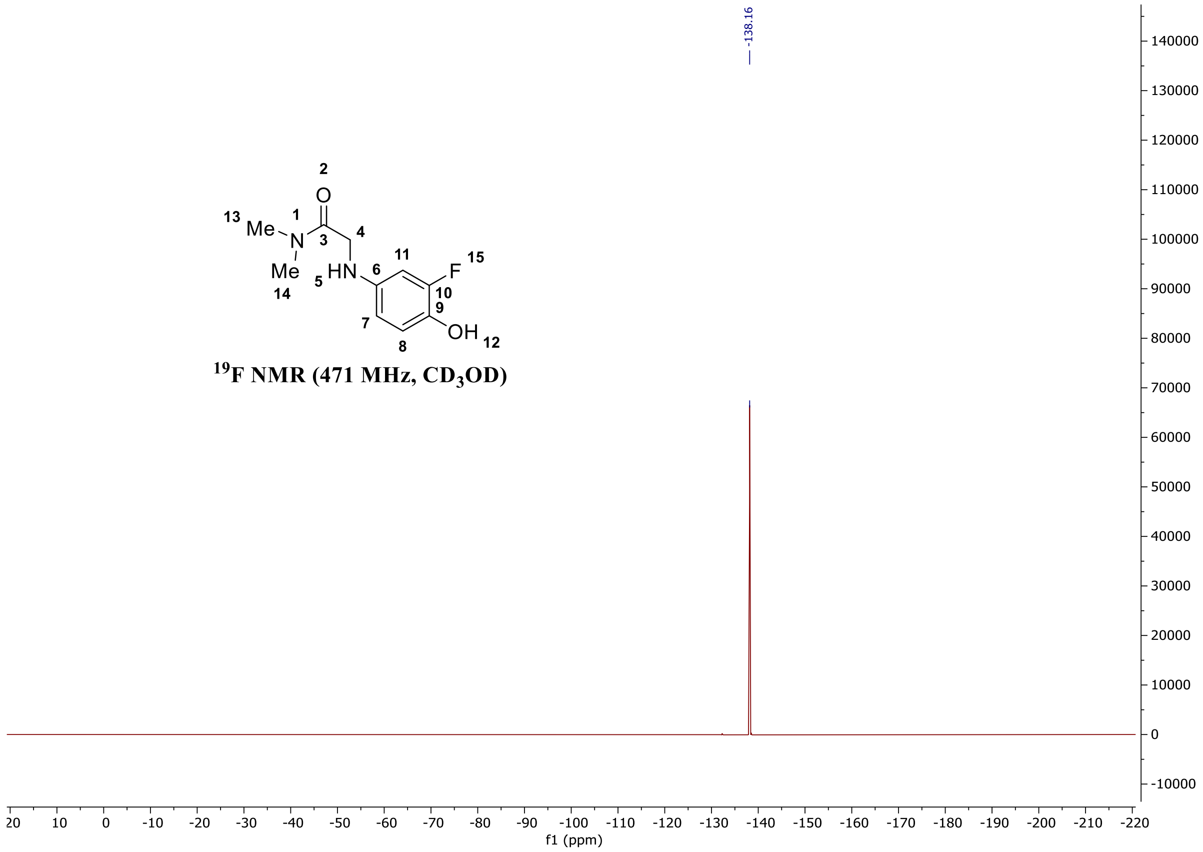

N,*N*-Dibenzyl-2-((4-hydroxyphenyl)amino)acetamide (1h)

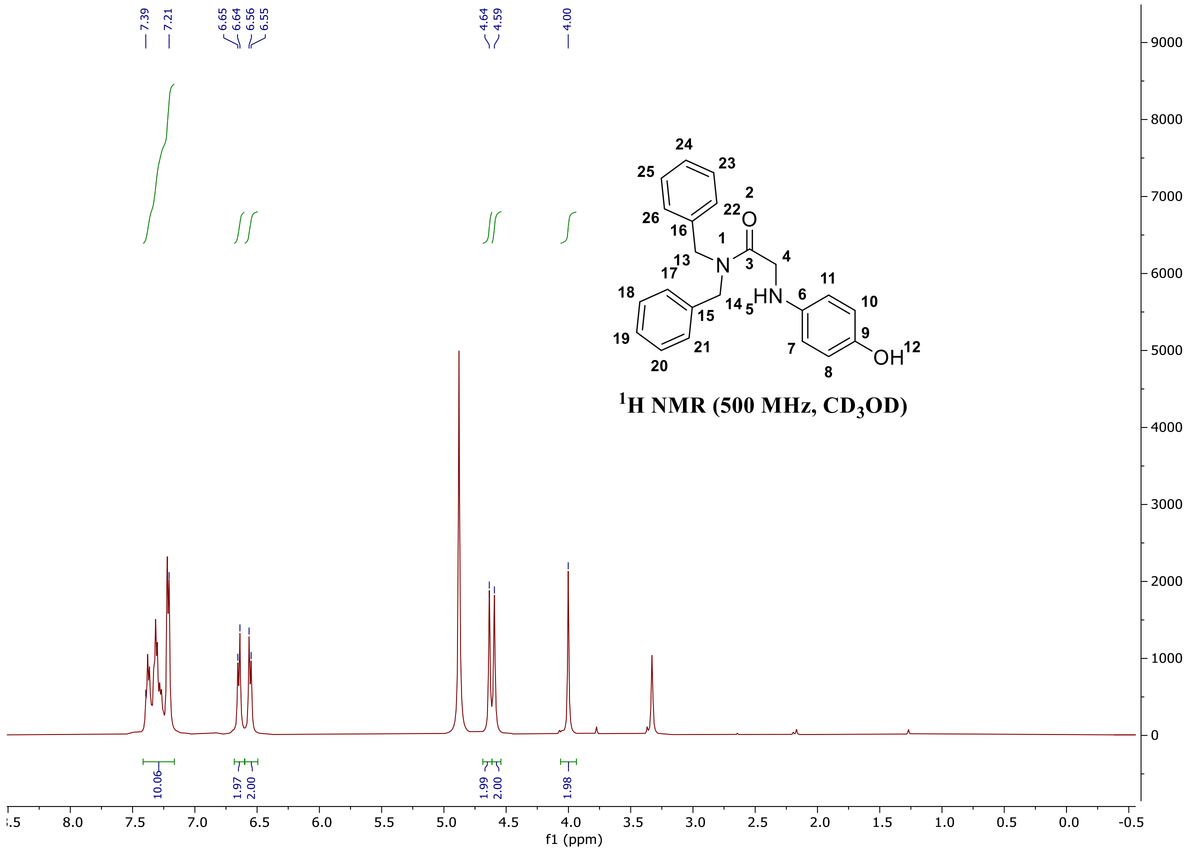

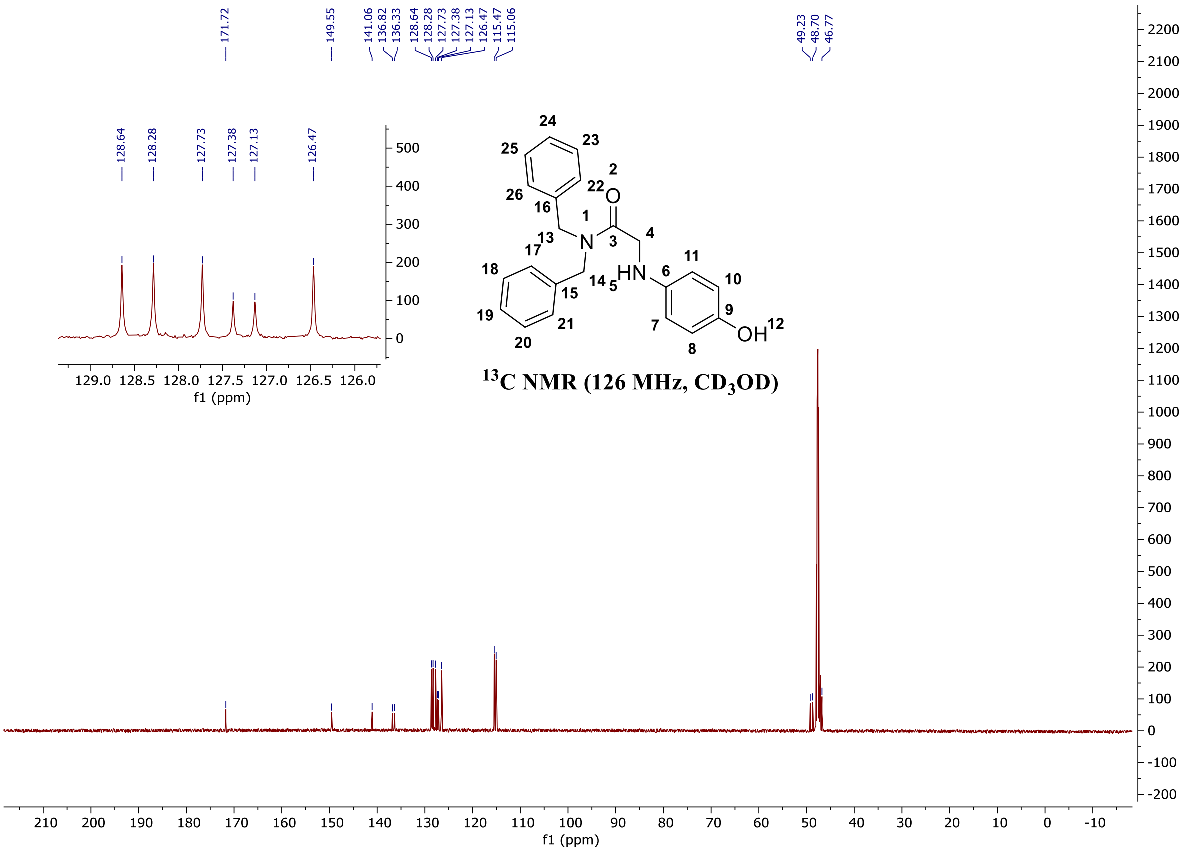

2-((4-Hydroxyphenyl)amino)-1-(pyrrolidin-1-yl)ethan-1-one (1i)

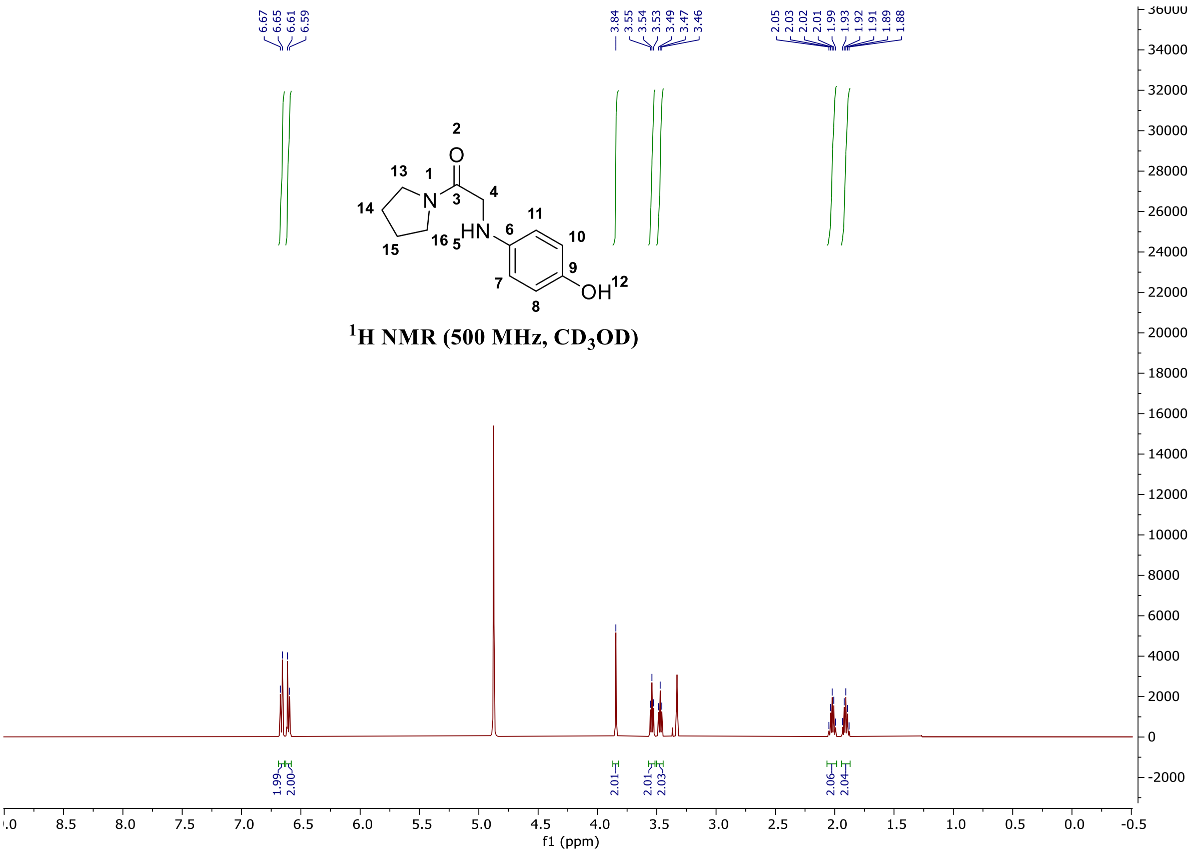

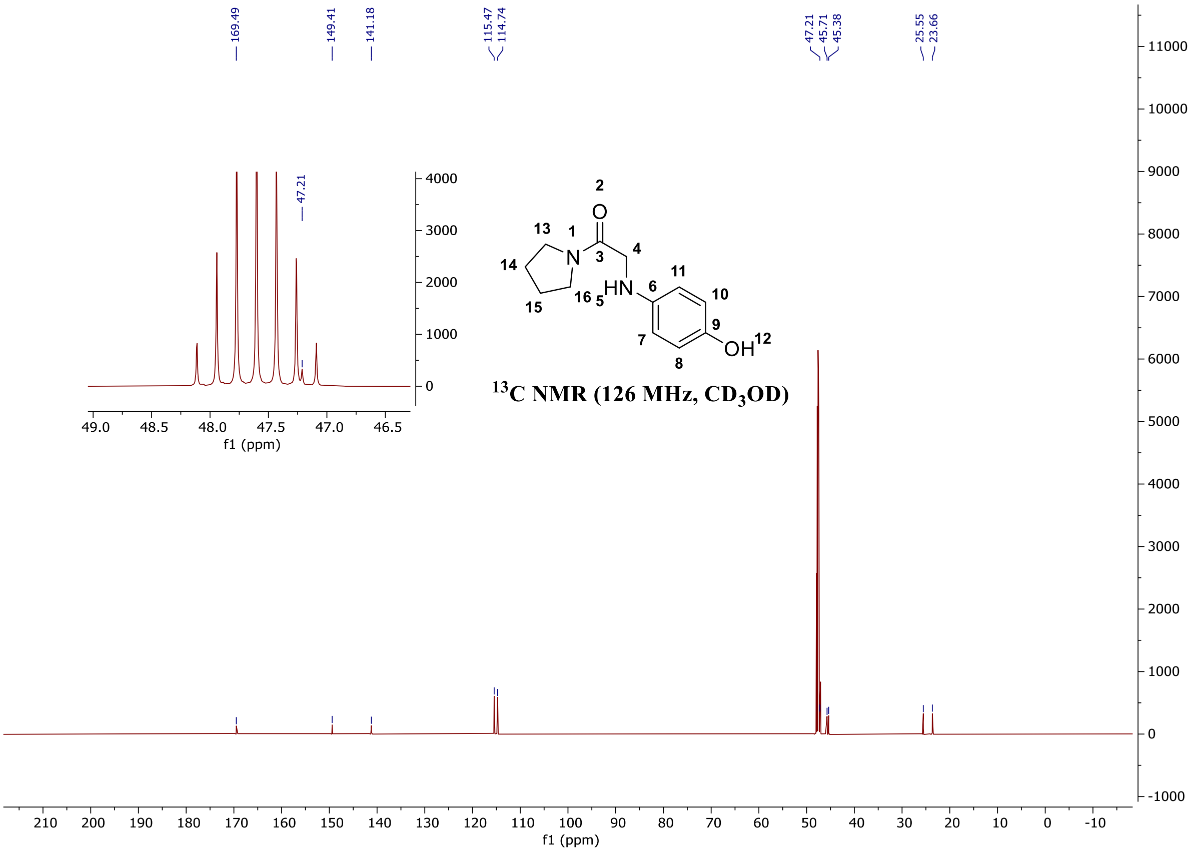

2-((4-Hydroxyphenyl)amino)-1-(piperidin-1-yl)ethan-1-one (1j)

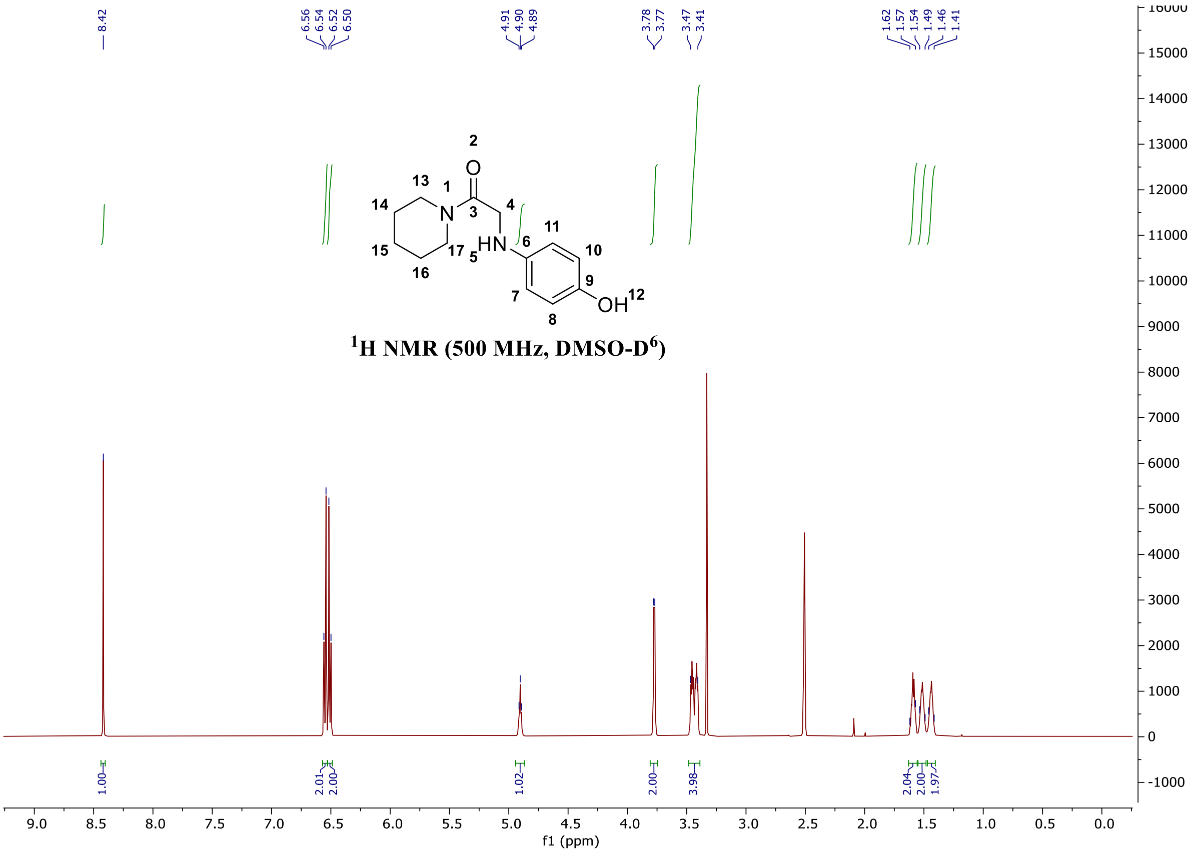

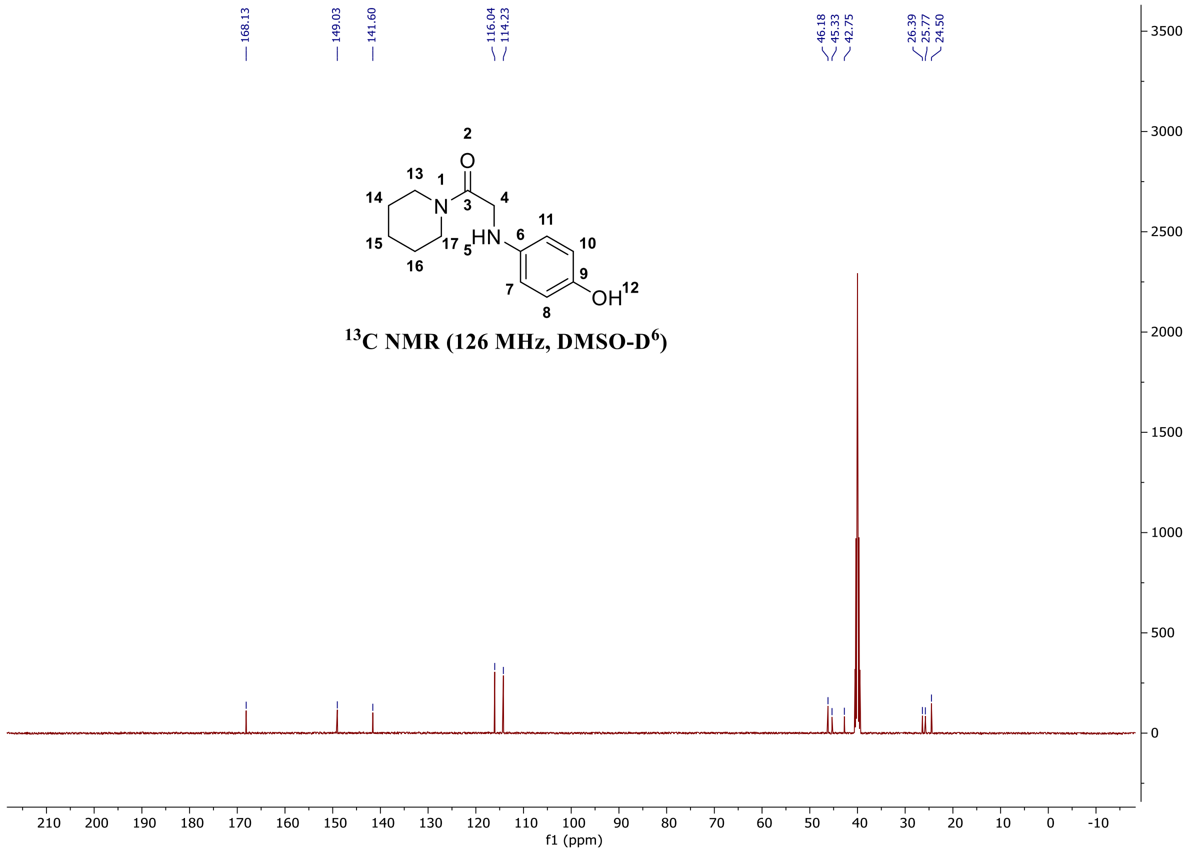

2-((4-Hydroxyphenyl)amino)-1-phenylethan-1-one (1m)

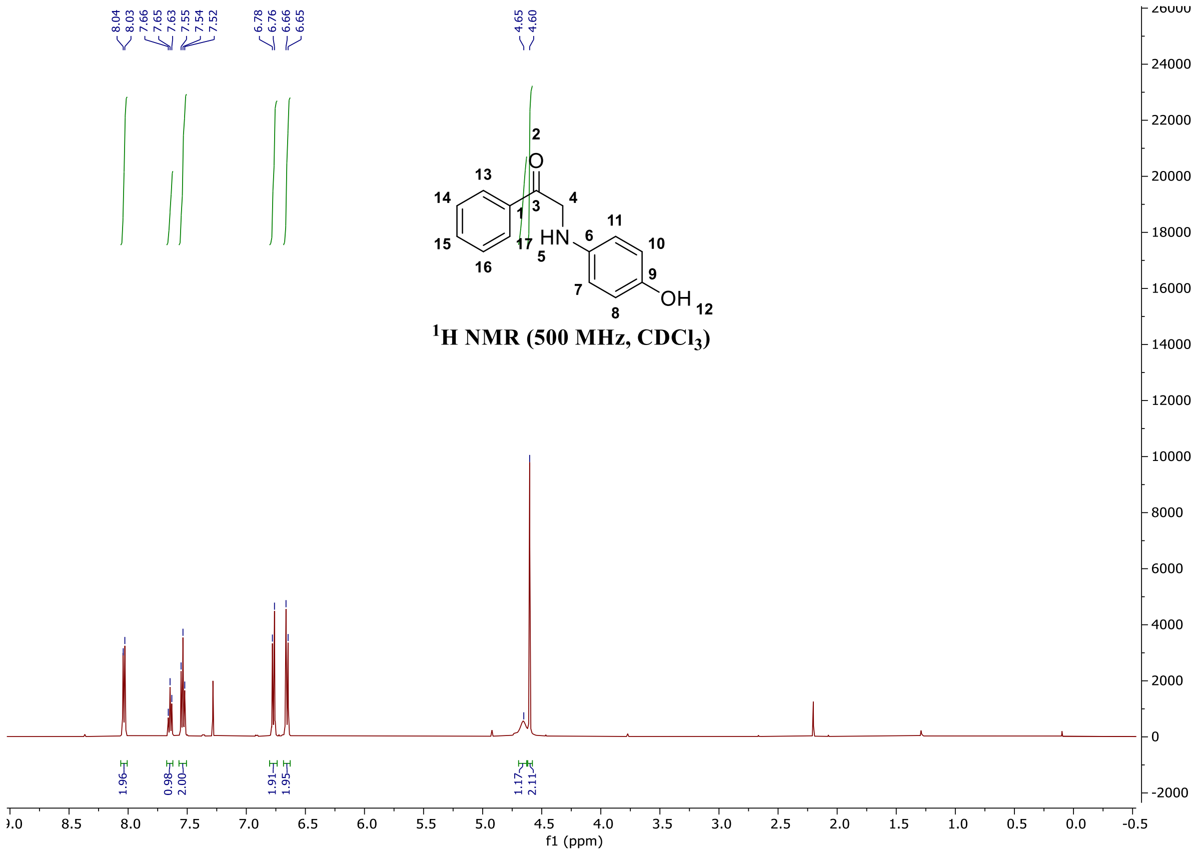

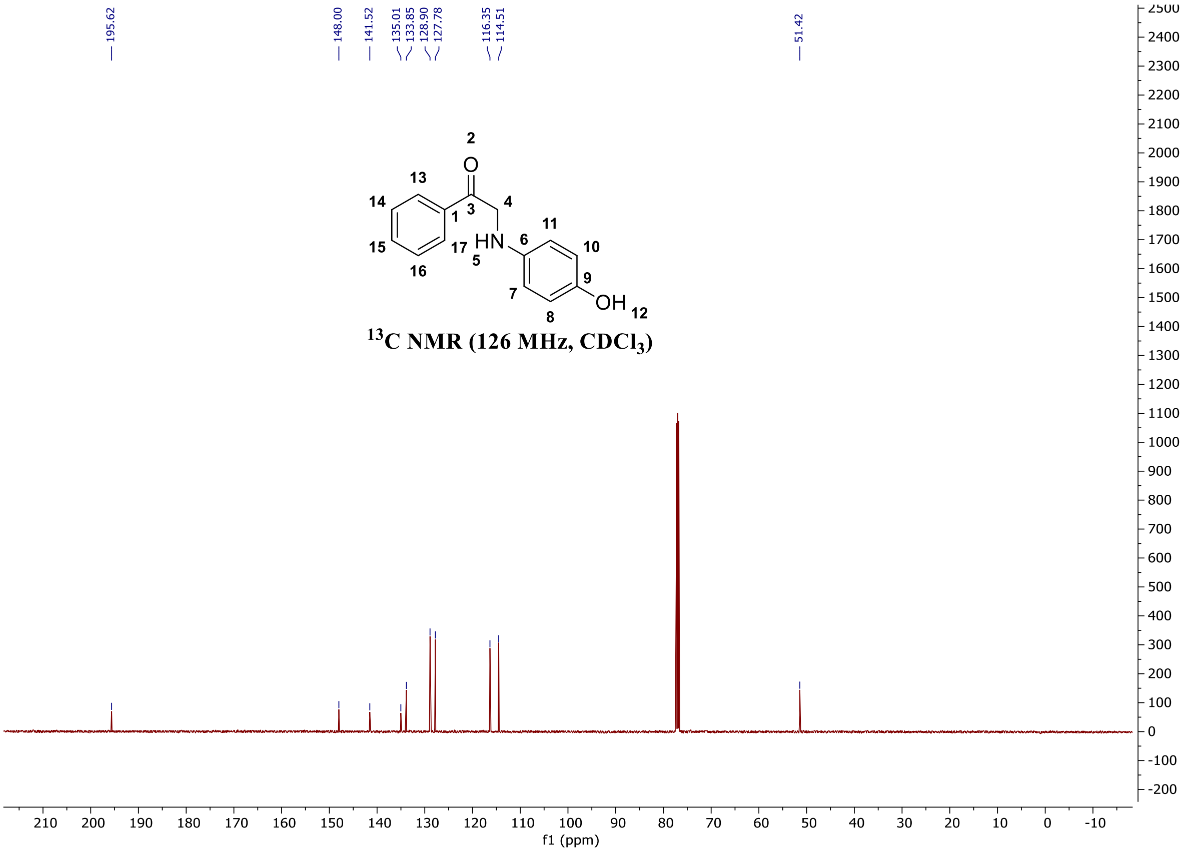

Ethyl (4-hydroxyphenyl)glycinate (1n)

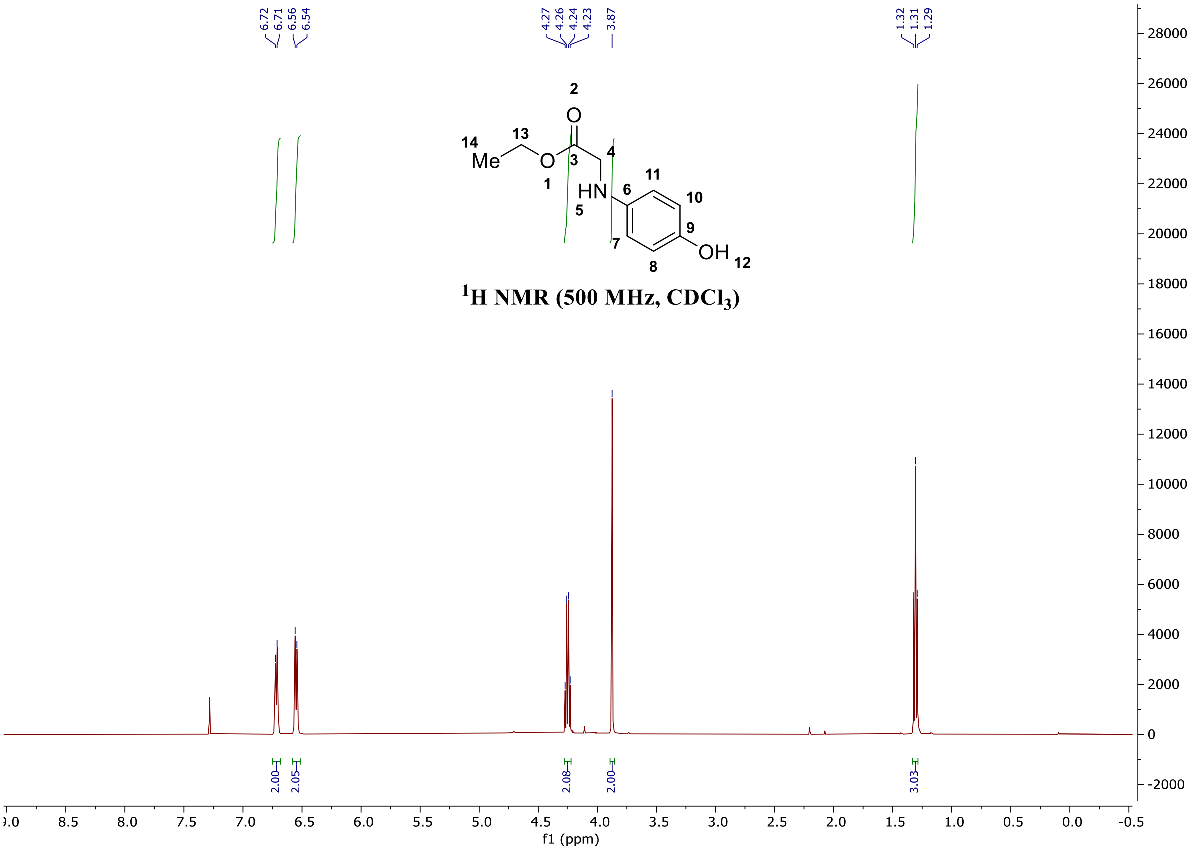

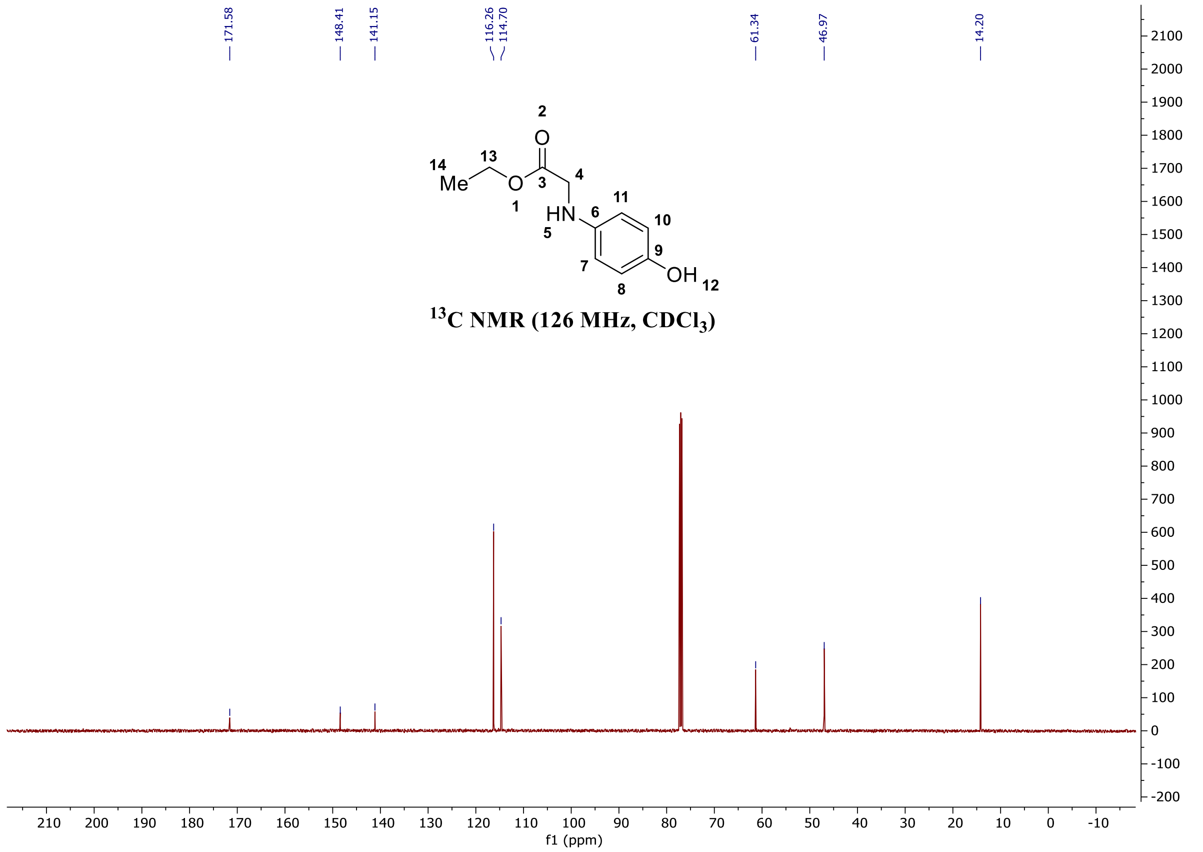

4,4,5,5-Tetramethyl-2-(4-(prop-1-en-2-yl)phenyl)-1,3,2-dioxaborolane (2i)

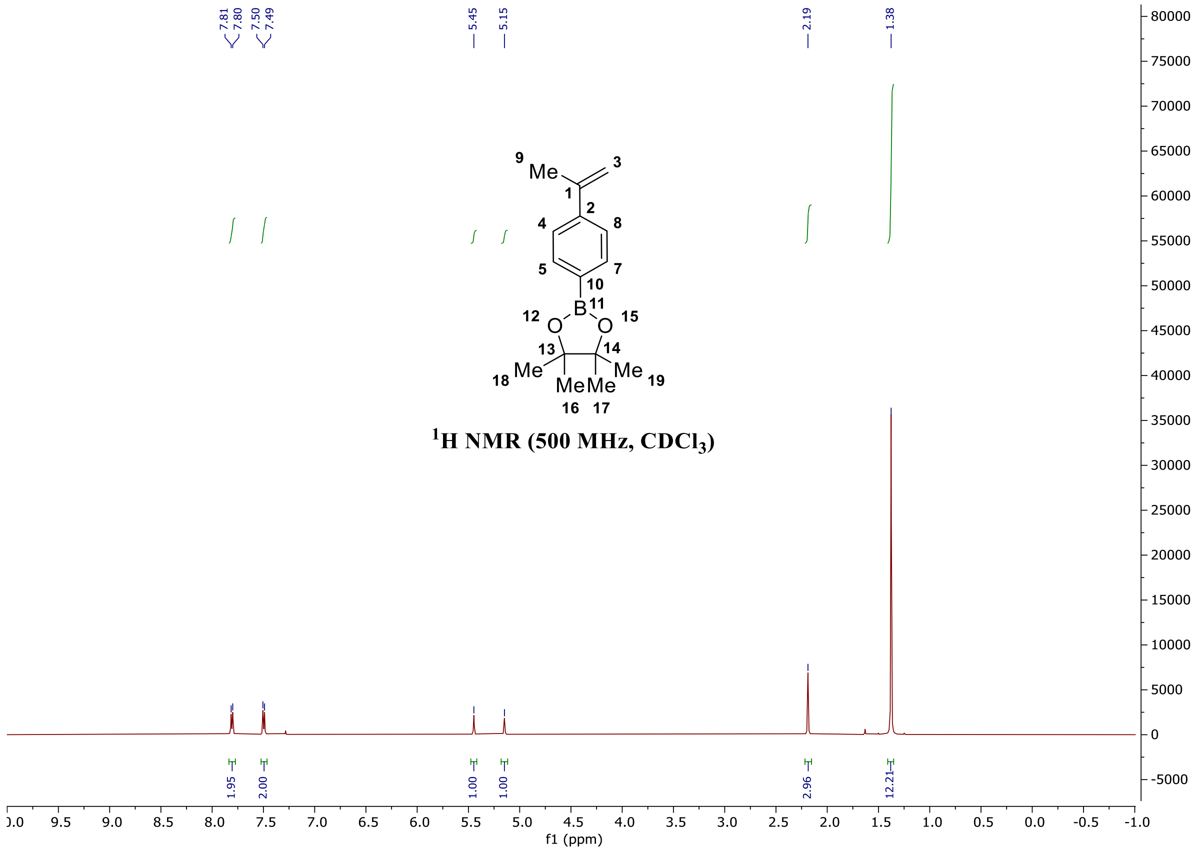

1-Fluoro-2-(prop-1-en-2-yl)benzene (2l)

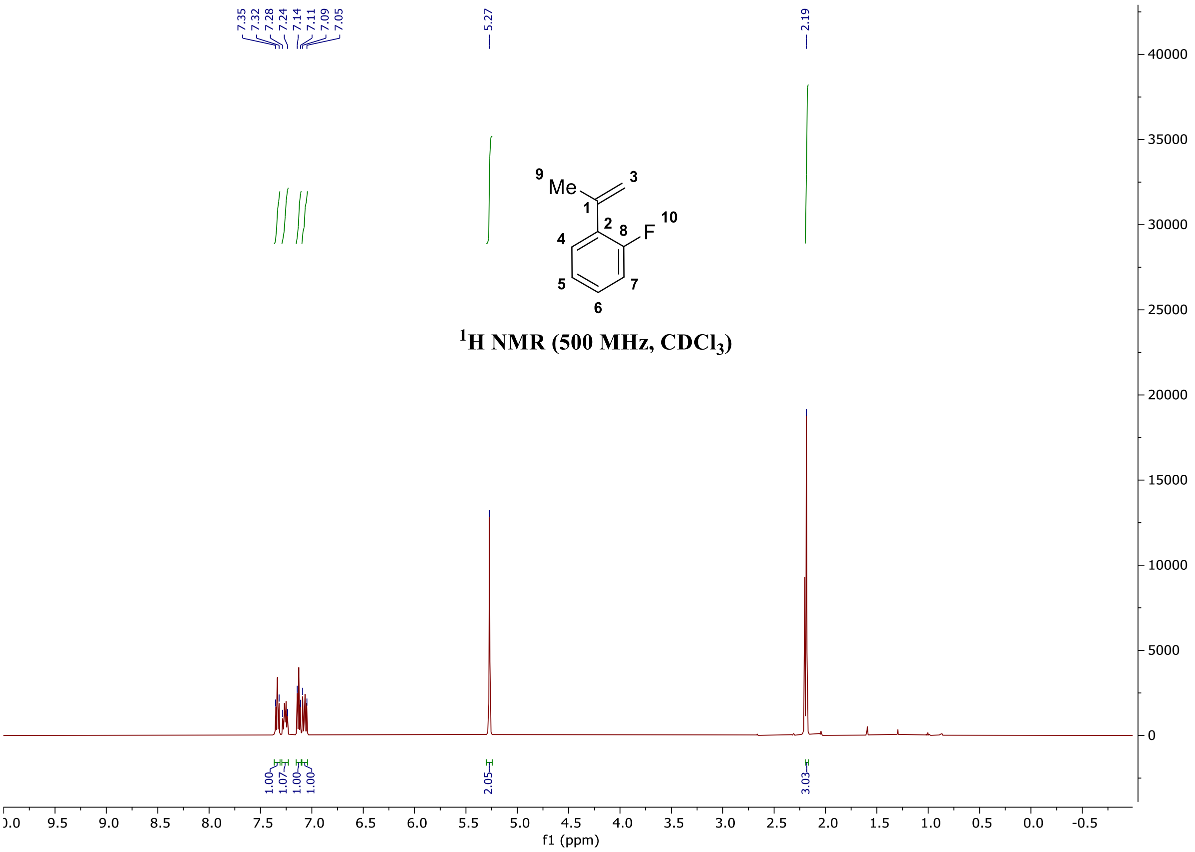

5-(Prop-1-en-2-yl)benzofuran (2n)

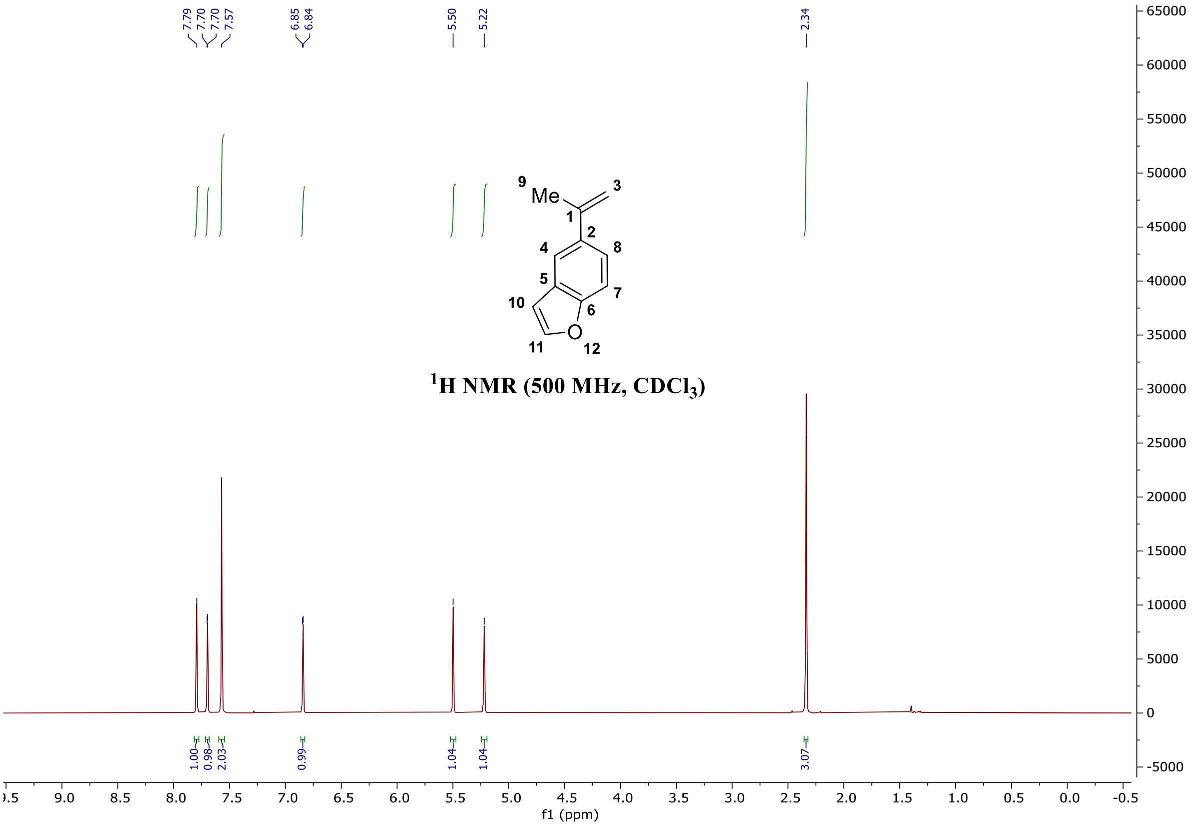

3-(Prop-1-en-2-yl)-1-tosyl-1*H*-indole (2o)

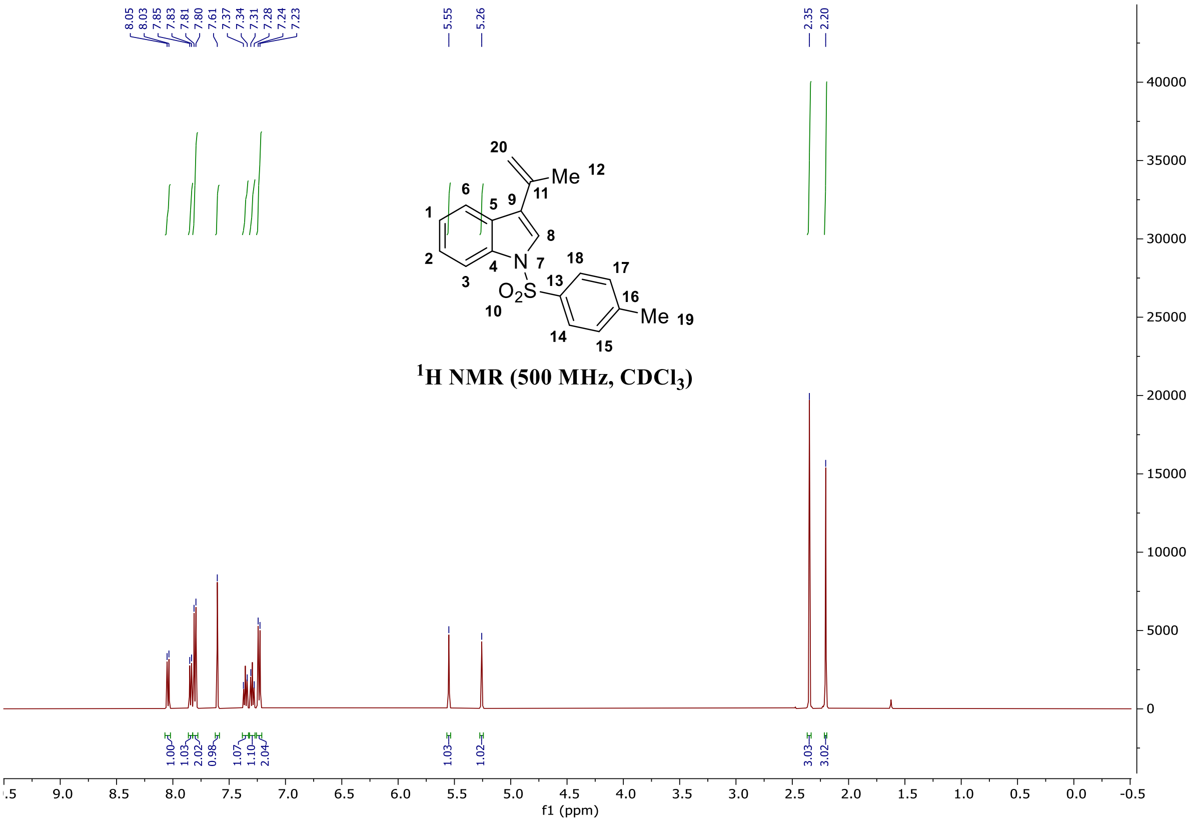

2-(Prop-1-en-2-yl)benzofuran (2p)

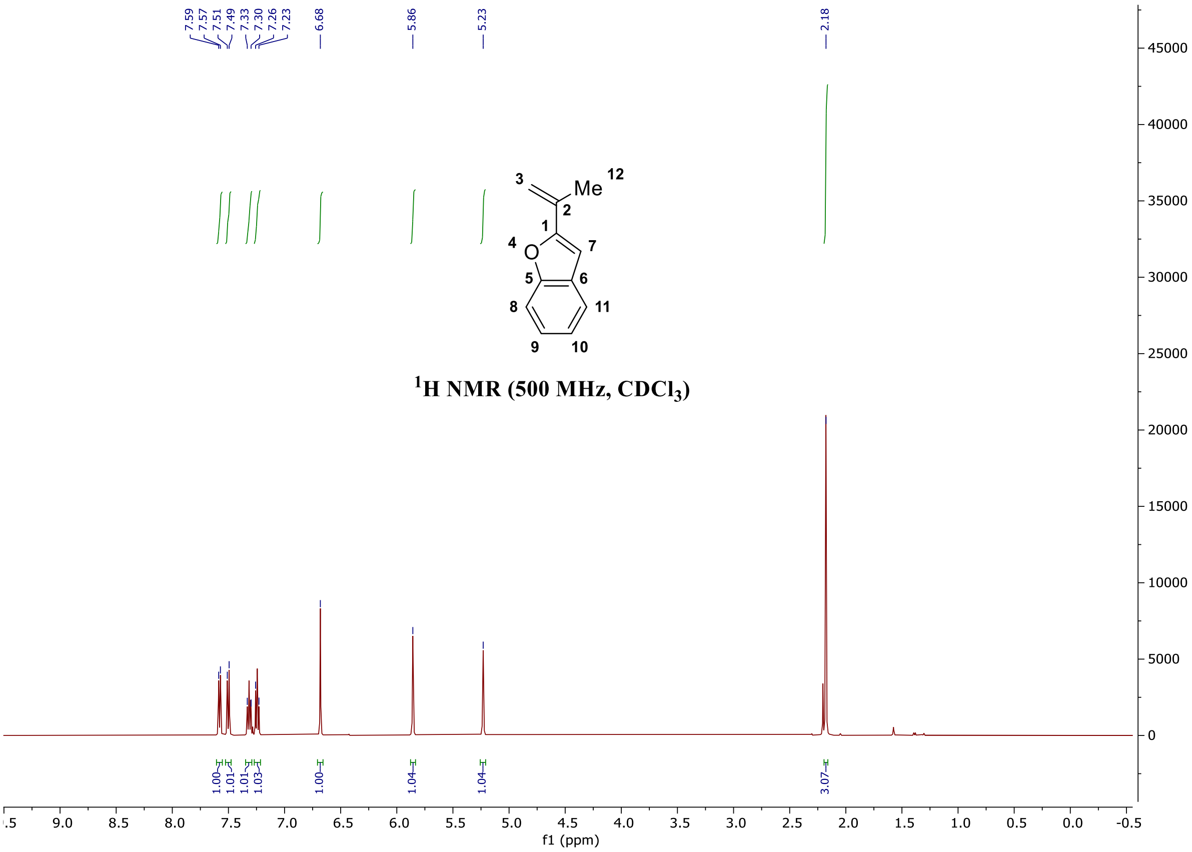

3-(Prop-1-en-2-yl)thiophene (2q)

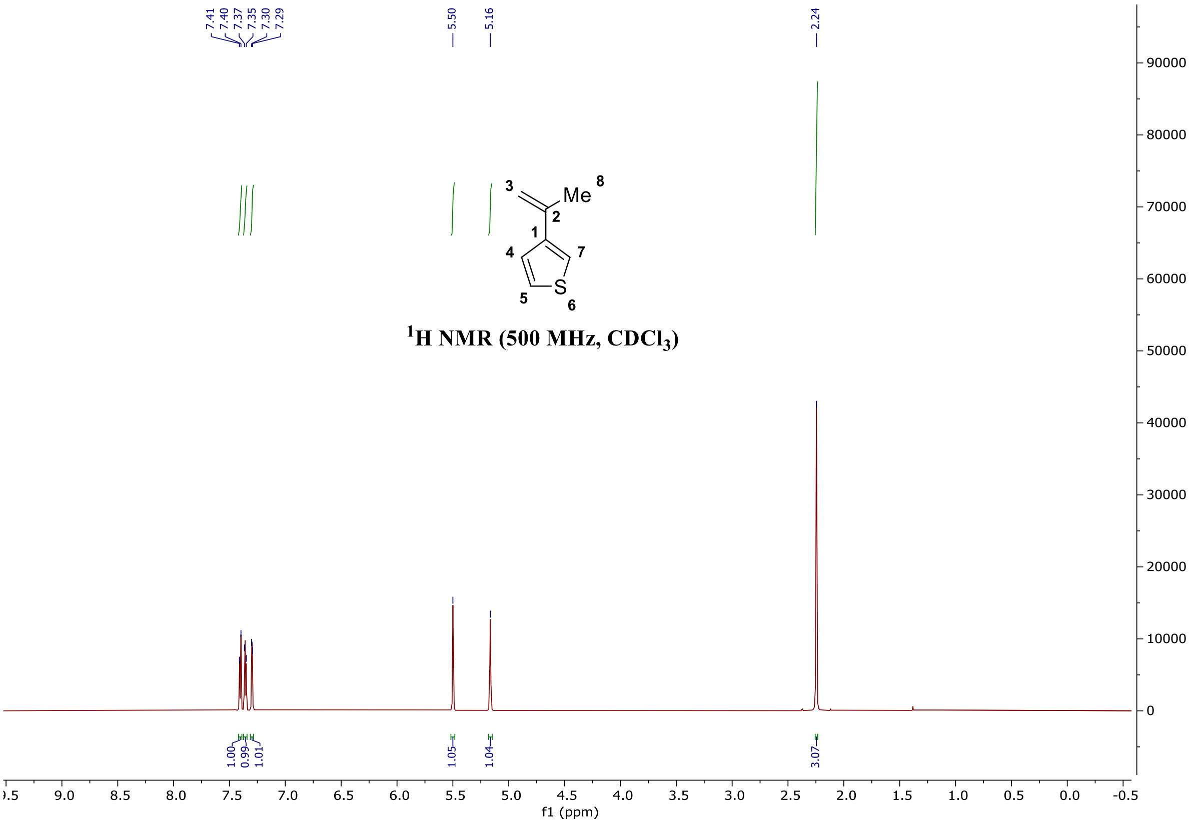

4-(*8R,9S,13S,14S*)-13-Methyl-3-(prop-1-en-2-yl)-6,7,8,9,11,12,13,14,15,16-decahydro-17*H*-cyclopenta[*a*]phenanthren-17-one (2r)

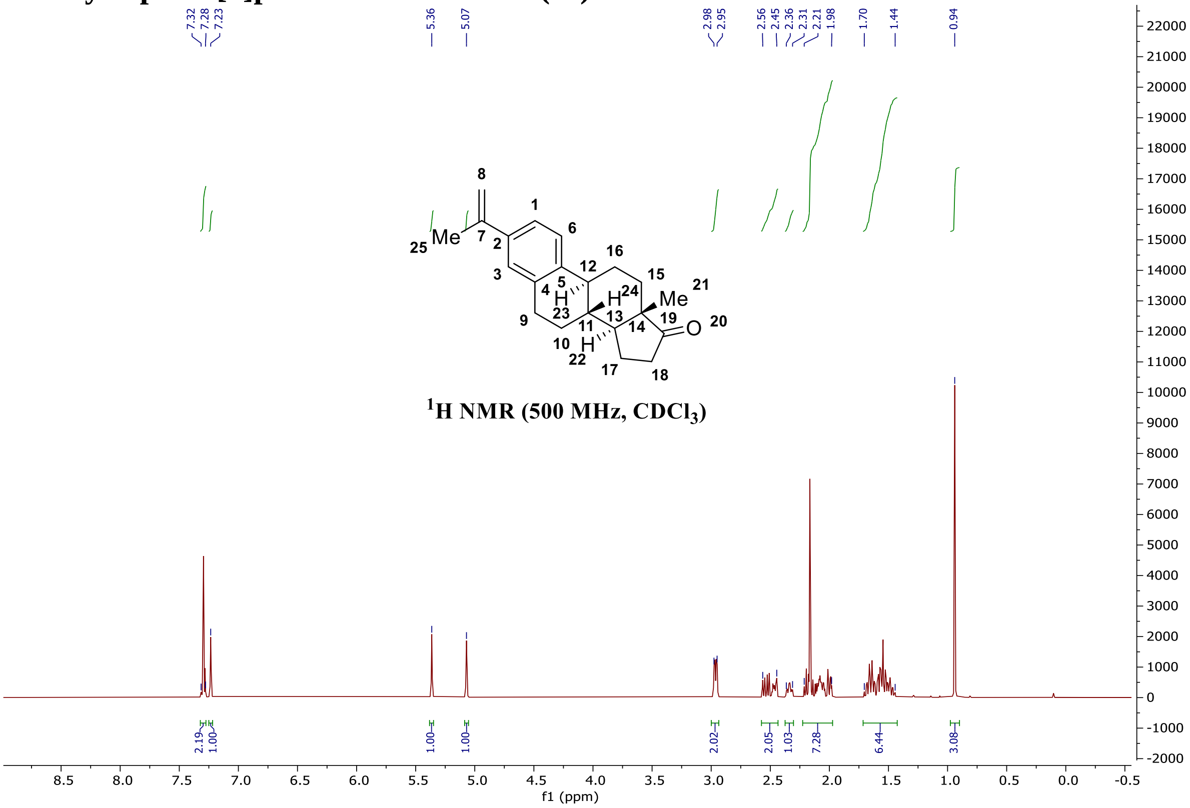

(1-Cyclobutylvinyl)benzene (2s)

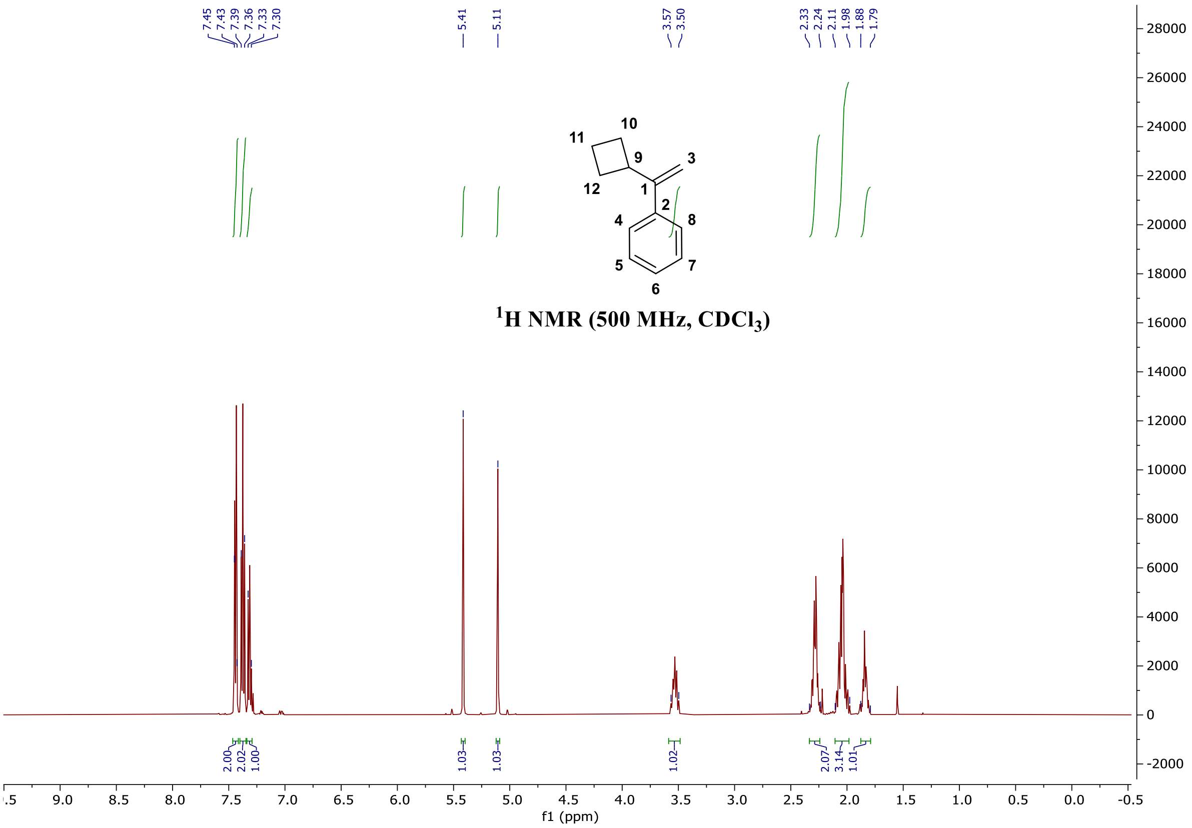

7-Methylenebicyclo[4.2.0]octa-1,3,5-triene (2u)

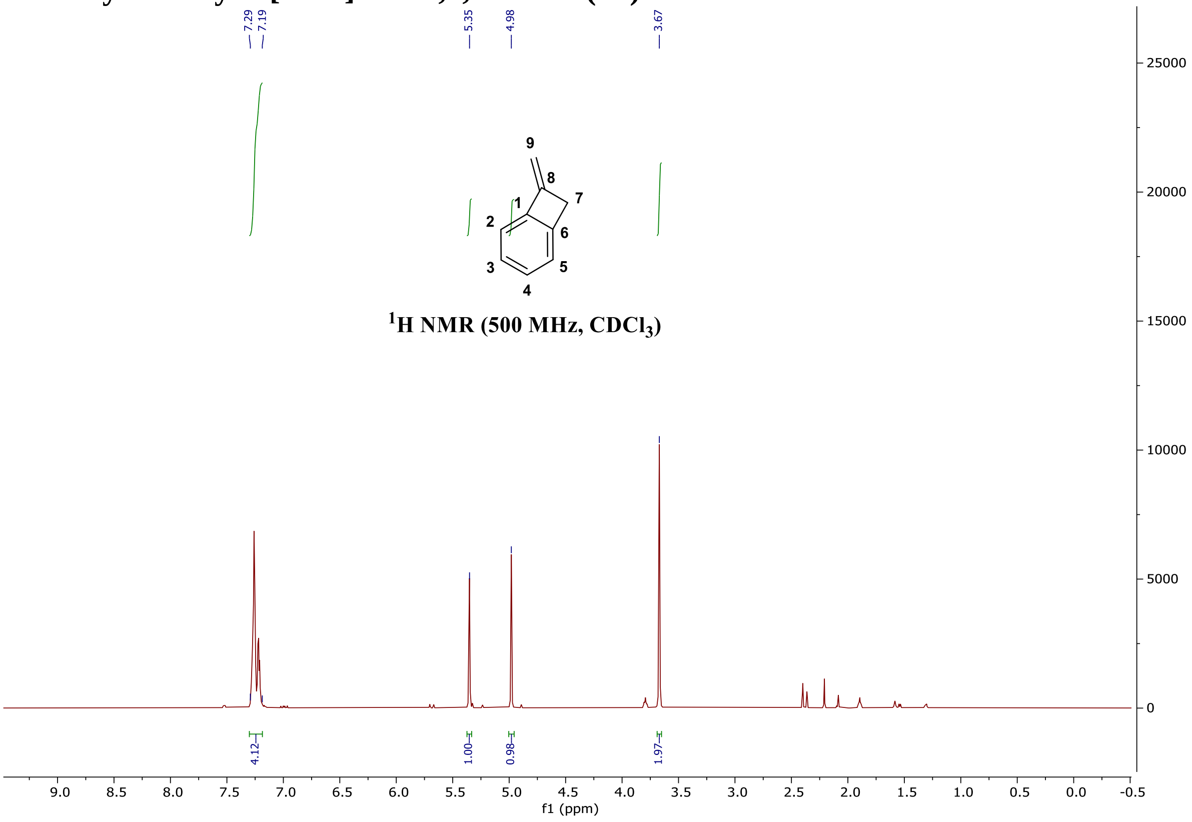

But-3-ene-1,3-diyldibenzene (2v)

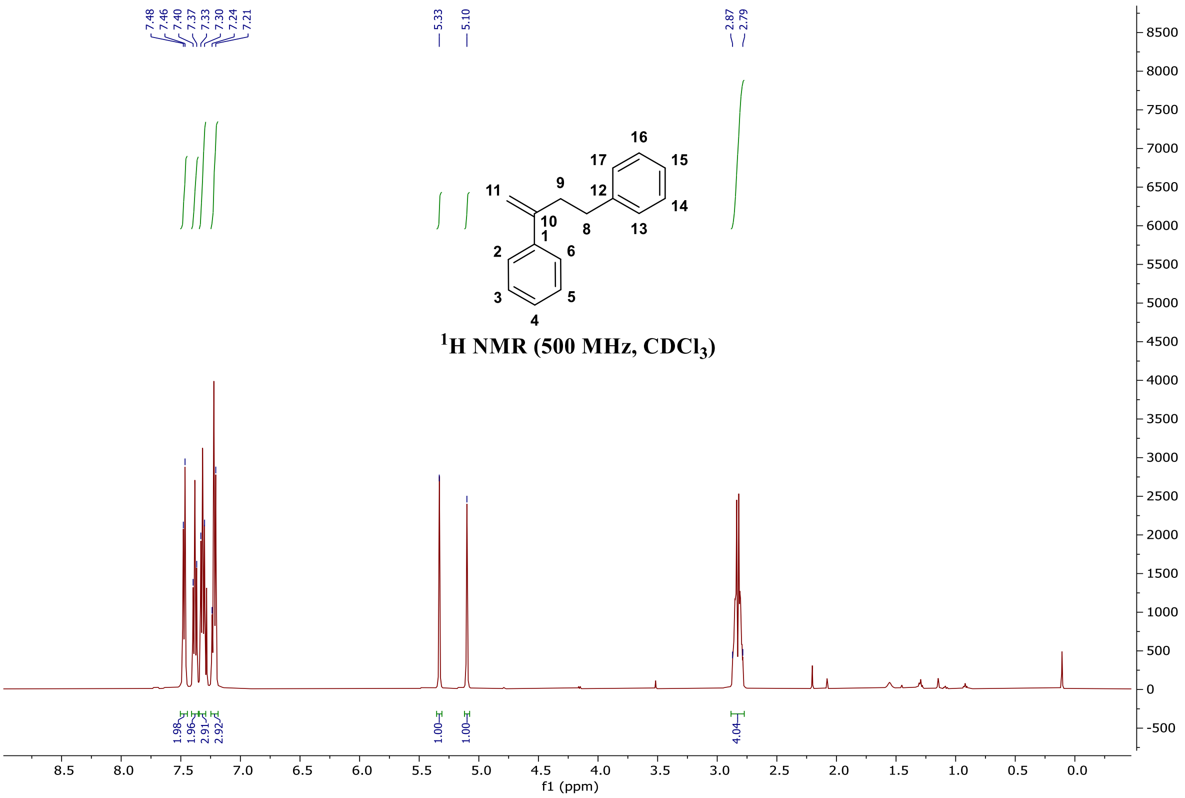

Oct-1-en-2-ylbenzene (2x)

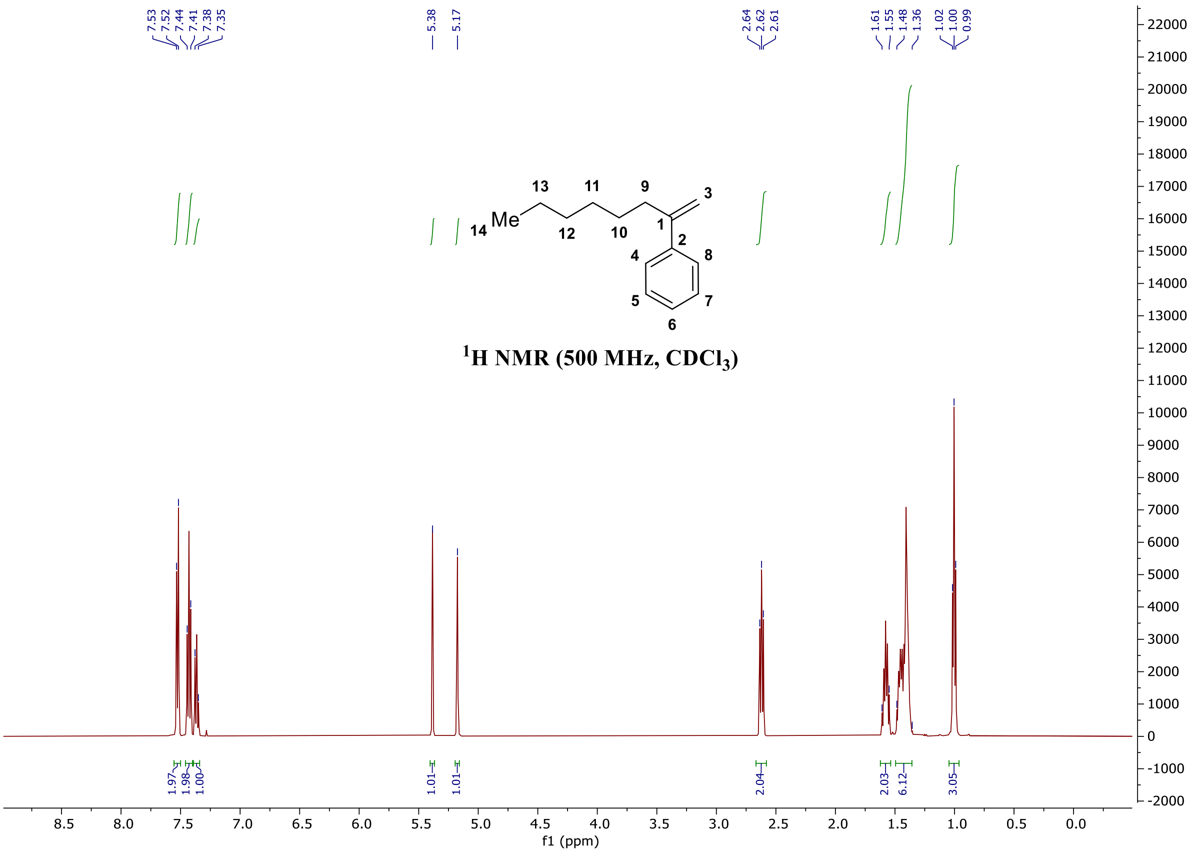

(*S*)-2-((4-Hydroxyphenyl)amino)-*N*,*N*,3-trimethyl-3-phenylbutanamide (3aa)

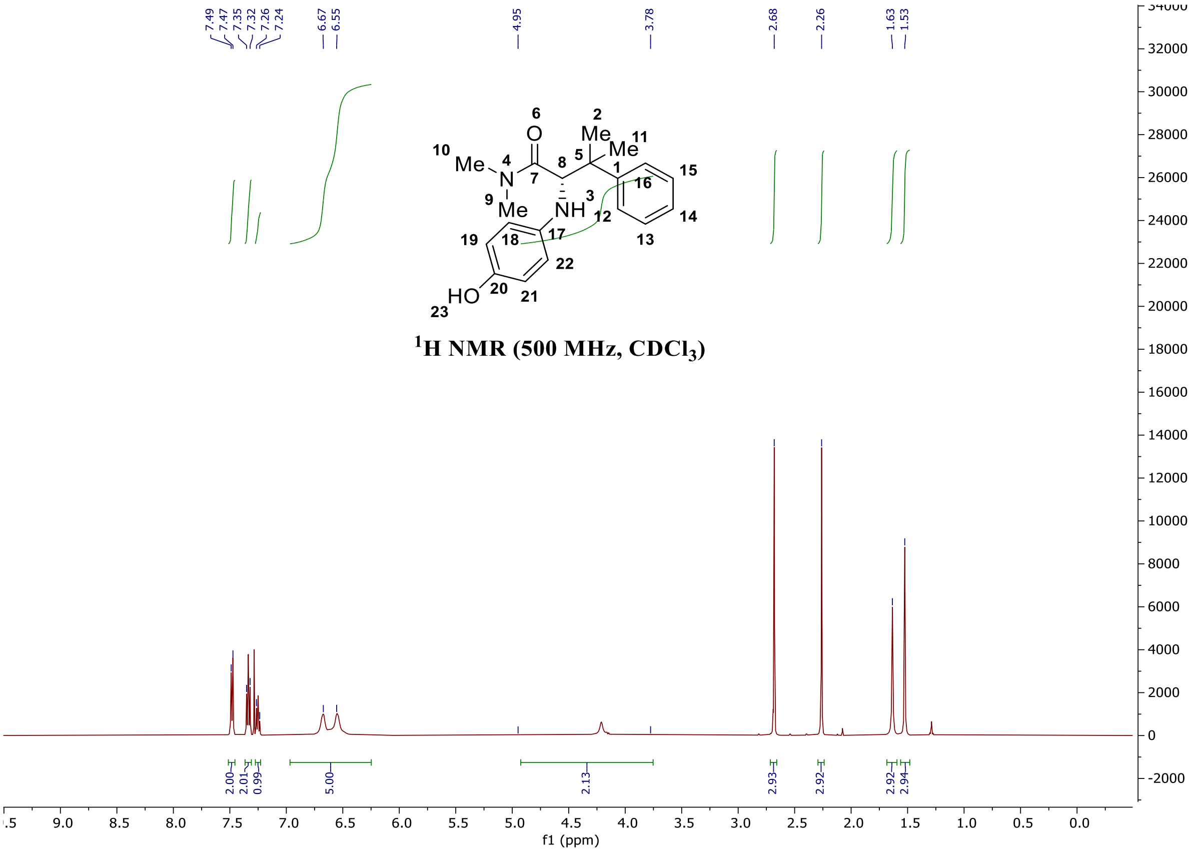

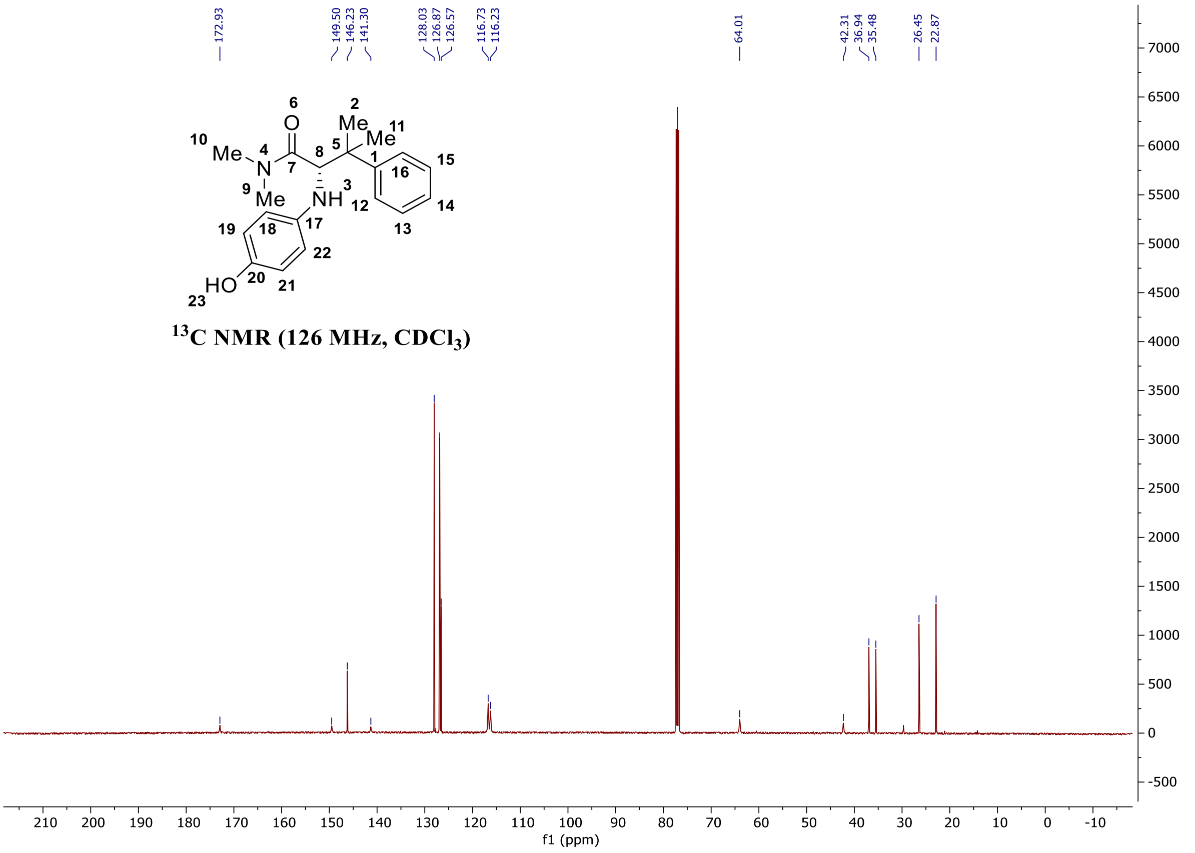

(*S*)-2-((4-Methoxyphenyl)amino)-*N*,*N*,3-trimethyl-3-phenylbutanamide (3ba)

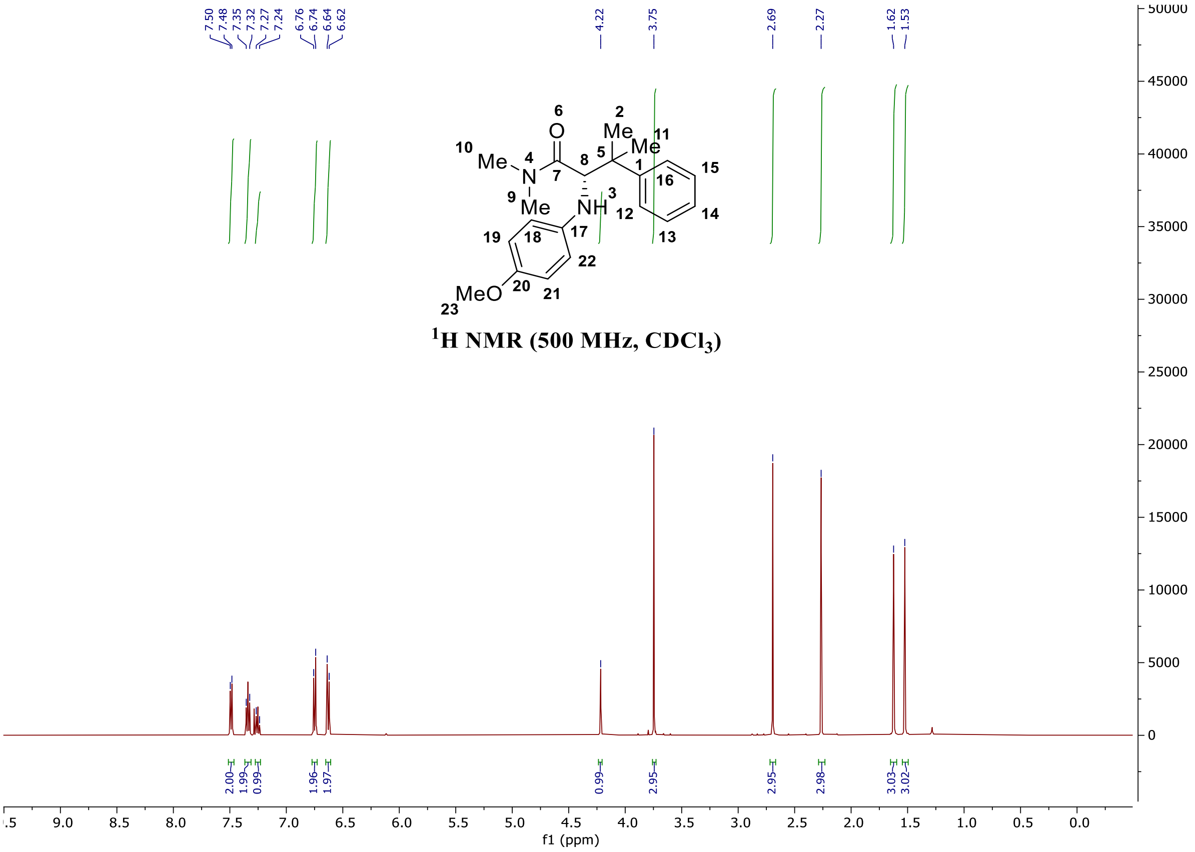

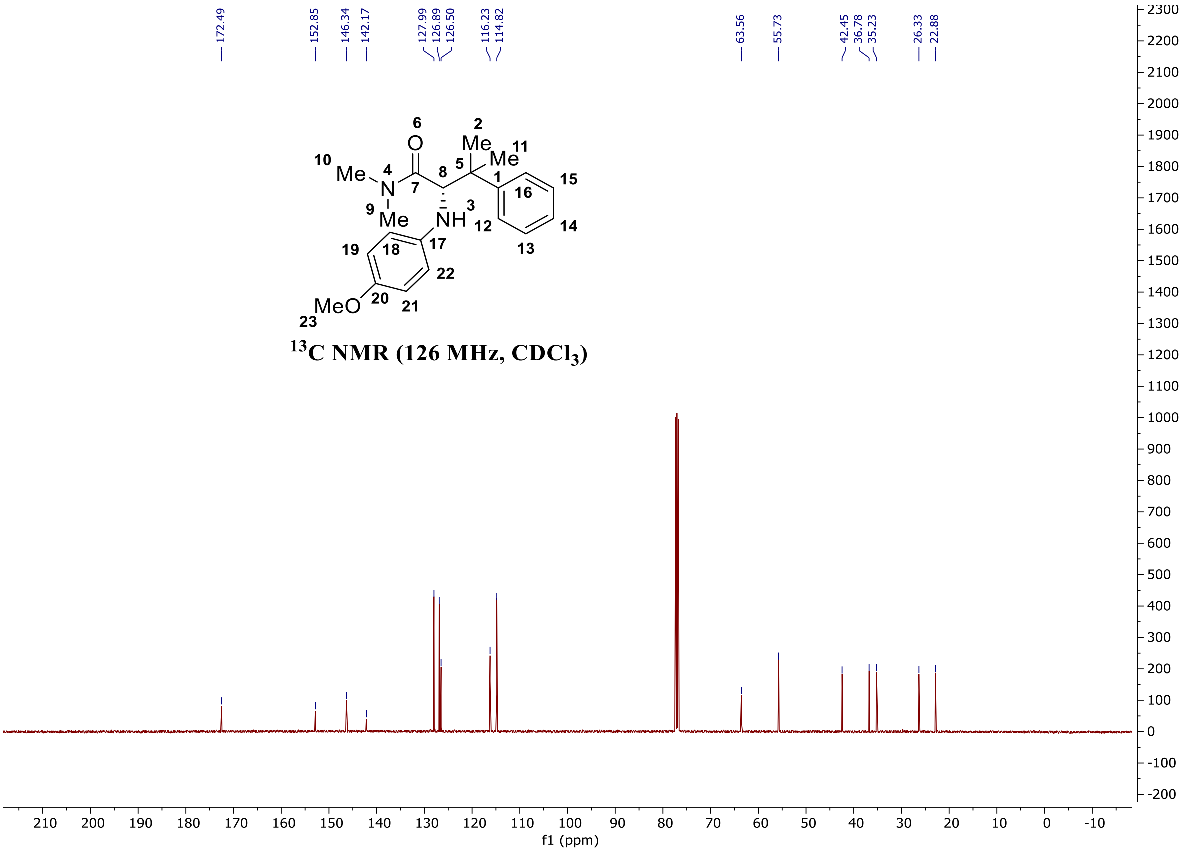

(S)-N,N,3-Trimethyl-3-phenyl-2-(p-tolylamino)butanamide (3ca)

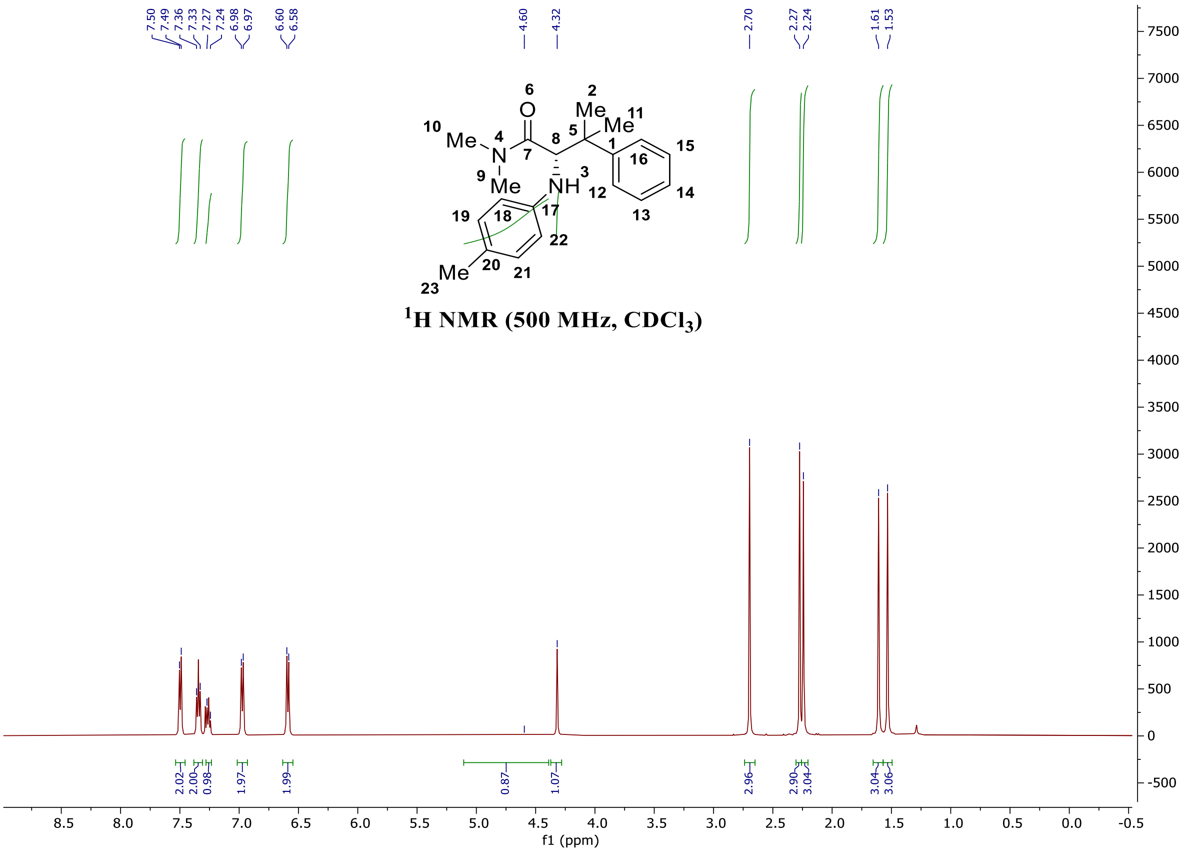

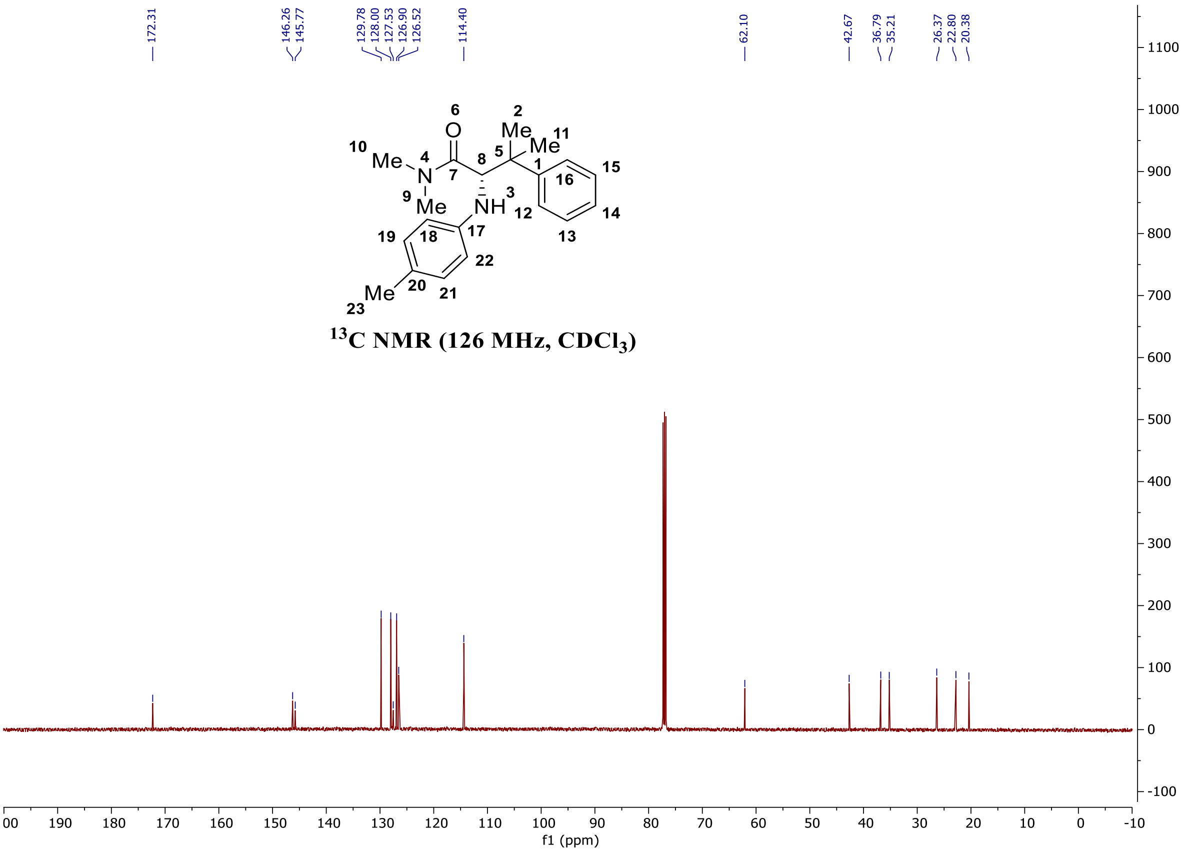

(*S*)-*N*,*N*,3-Trimethyl-3-phenyl-2-(phenylamino)butanamide (3da)

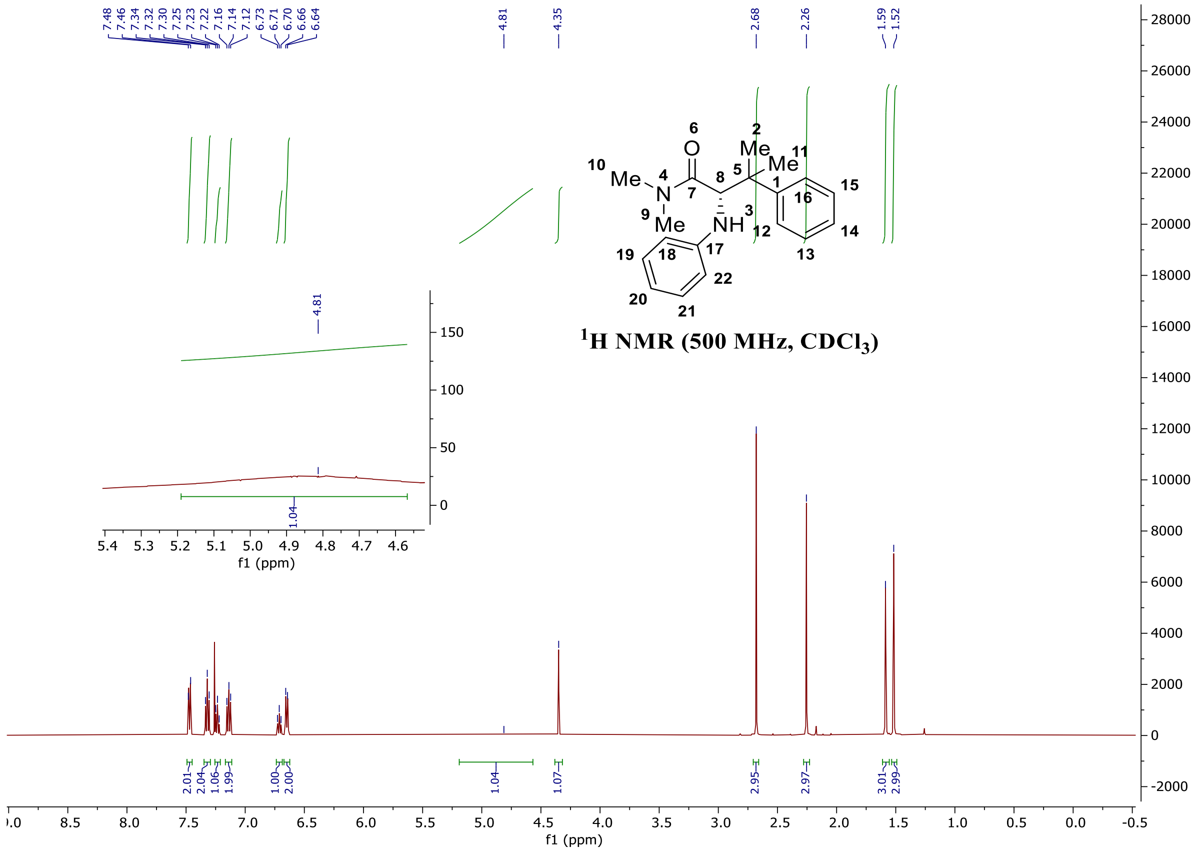

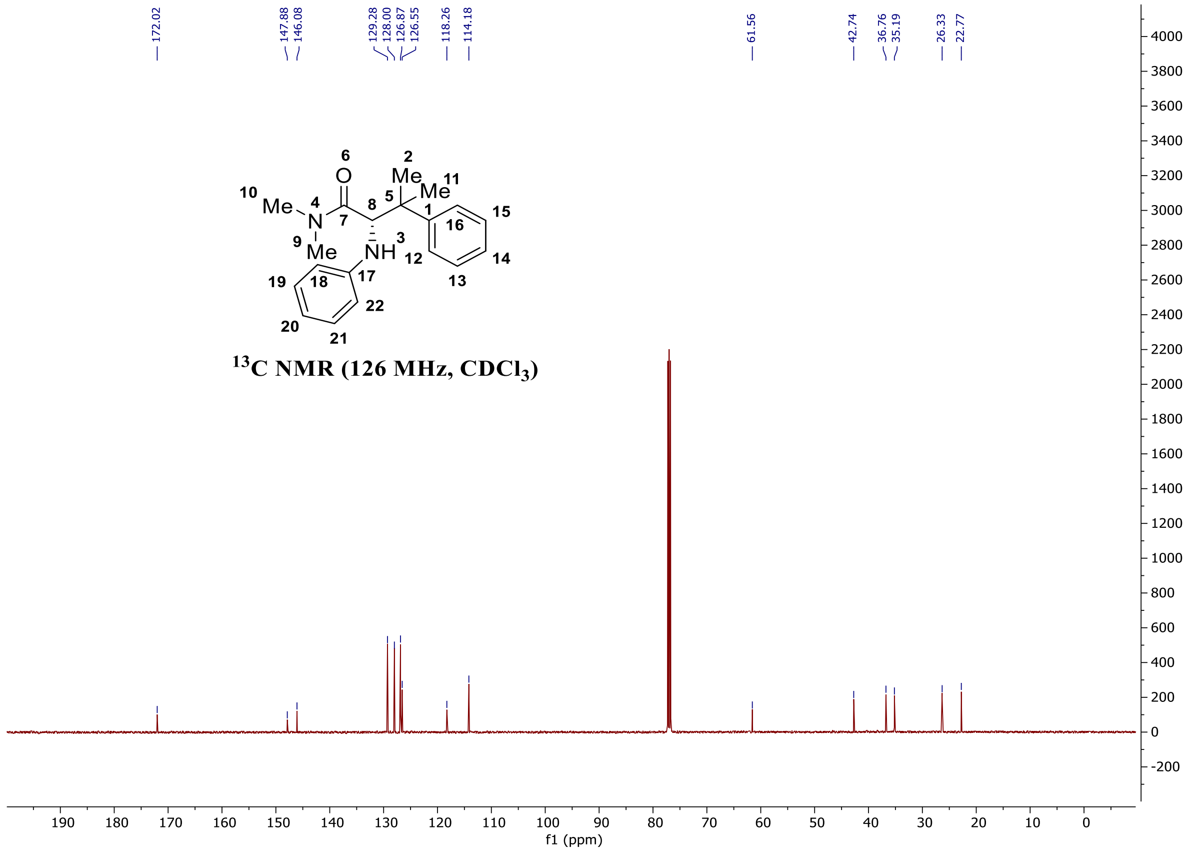

(*S*)-2-((4-Fluorophenyl)amino)-*N*,*N*,3-trimethyl-3-phenylbutanamide (3ea)

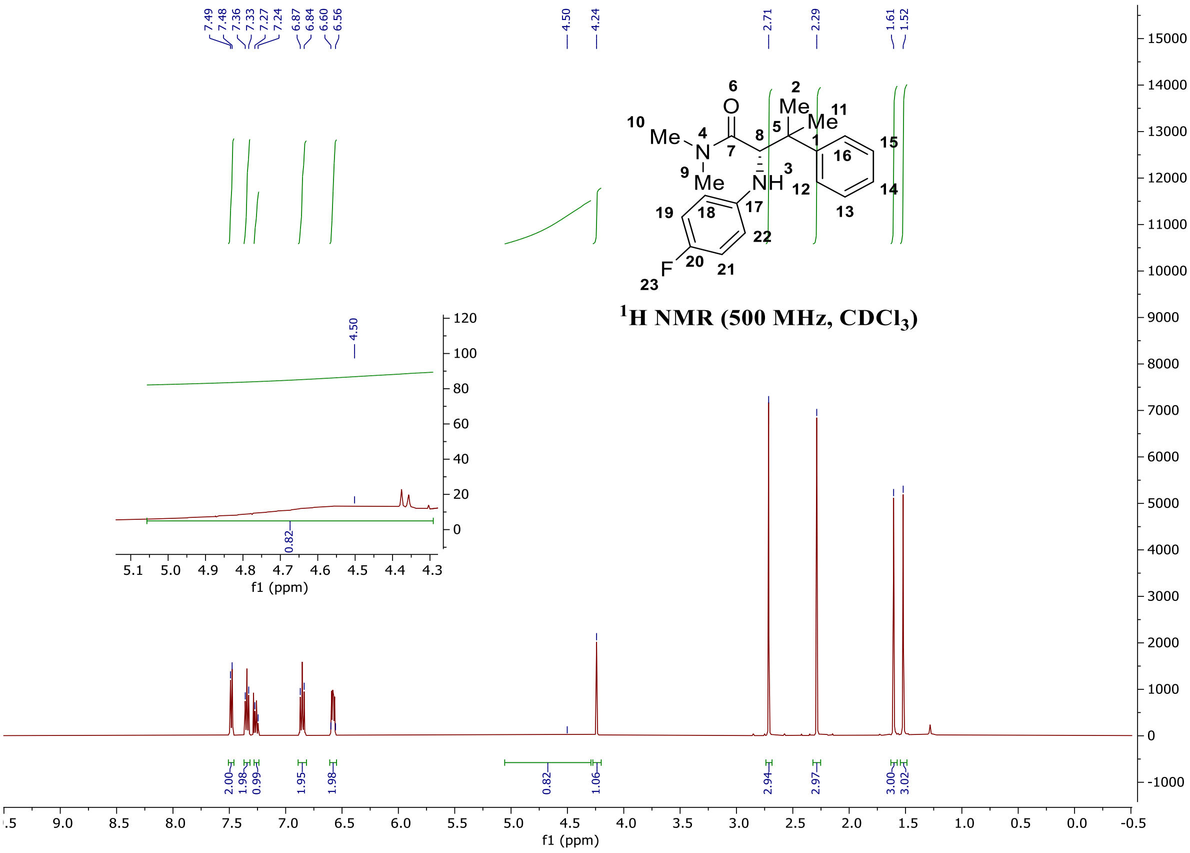

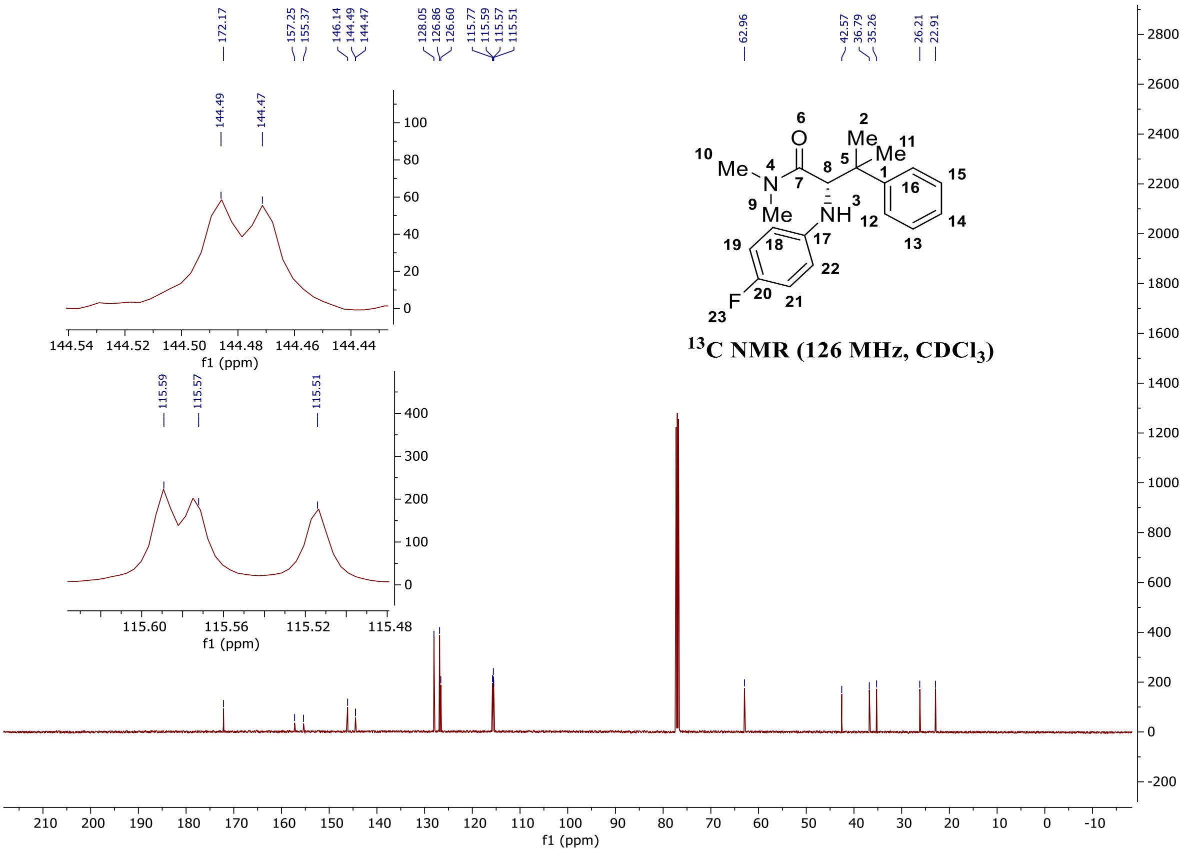

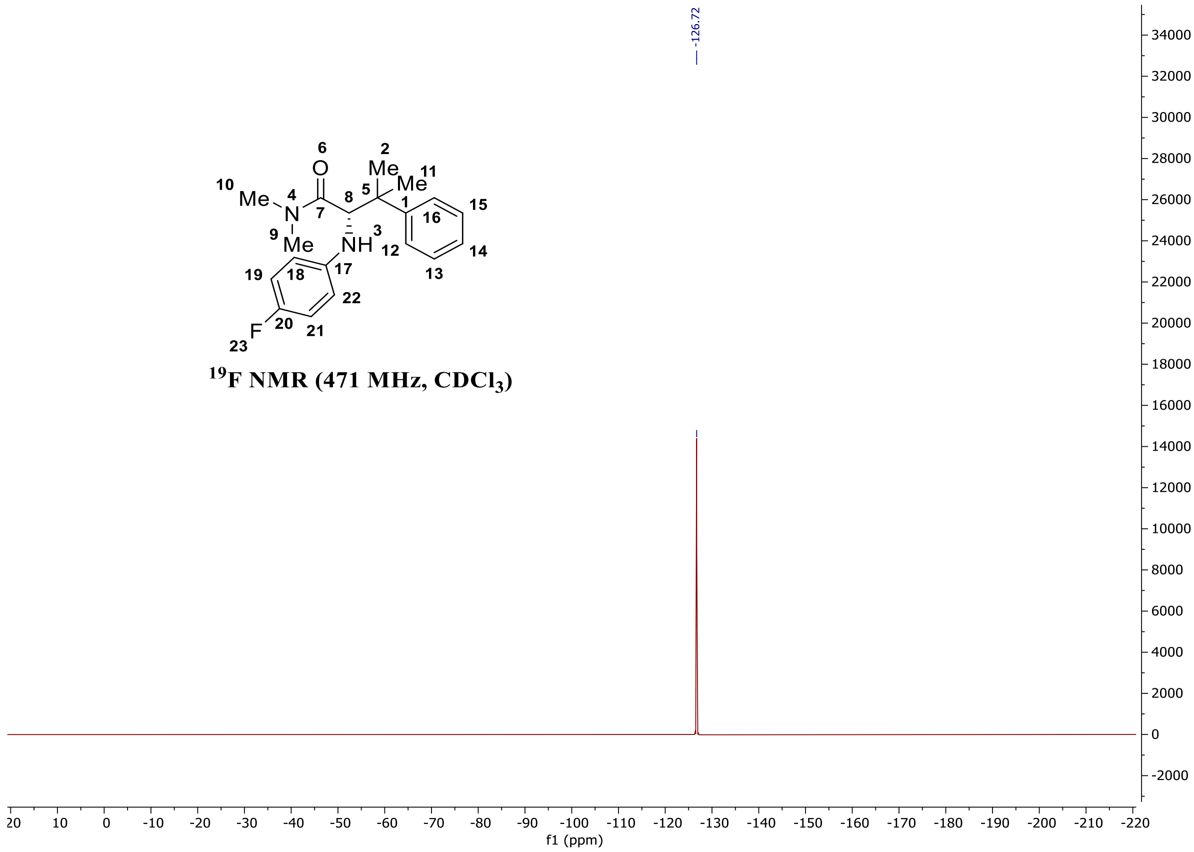

(*S*)-2-((4-Fluoro-3-methoxyphenyl)amino)-*N*,*N*,3-trimethyl-3-phenylbutanamide (3fa)

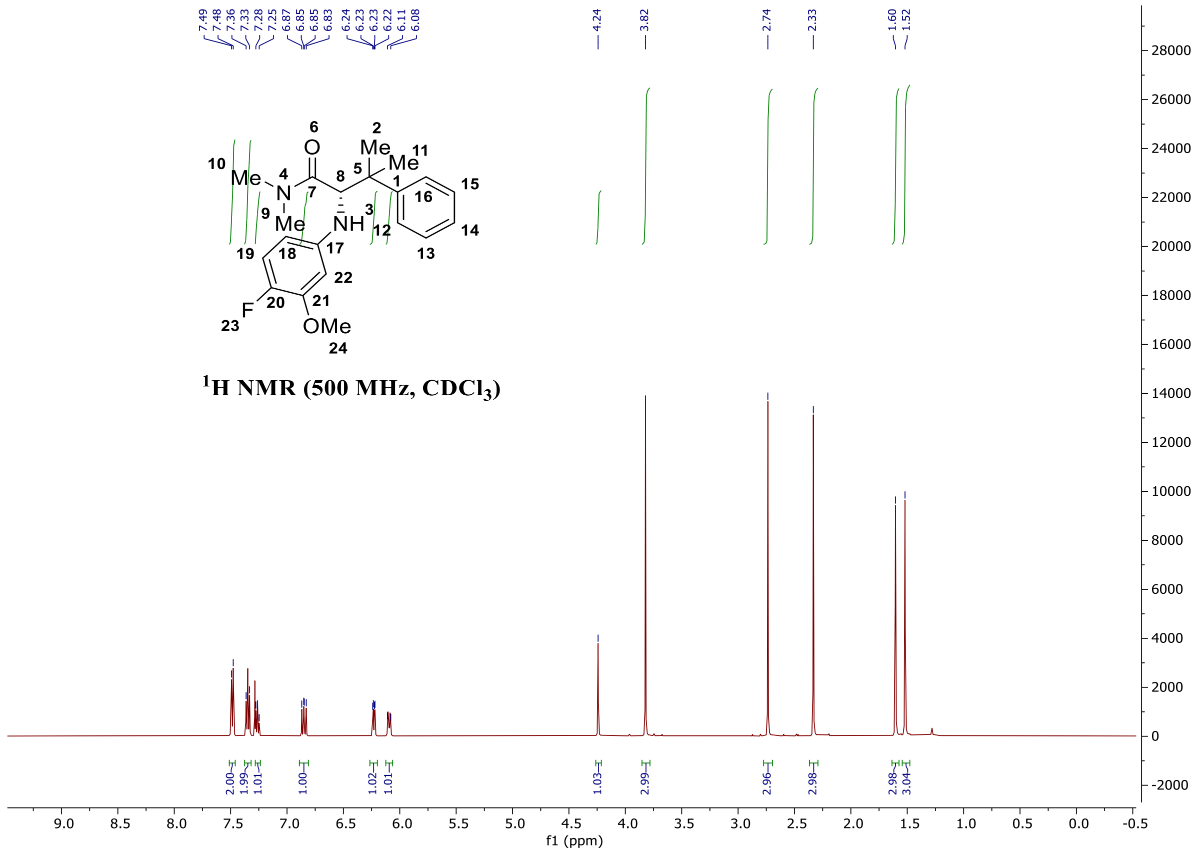

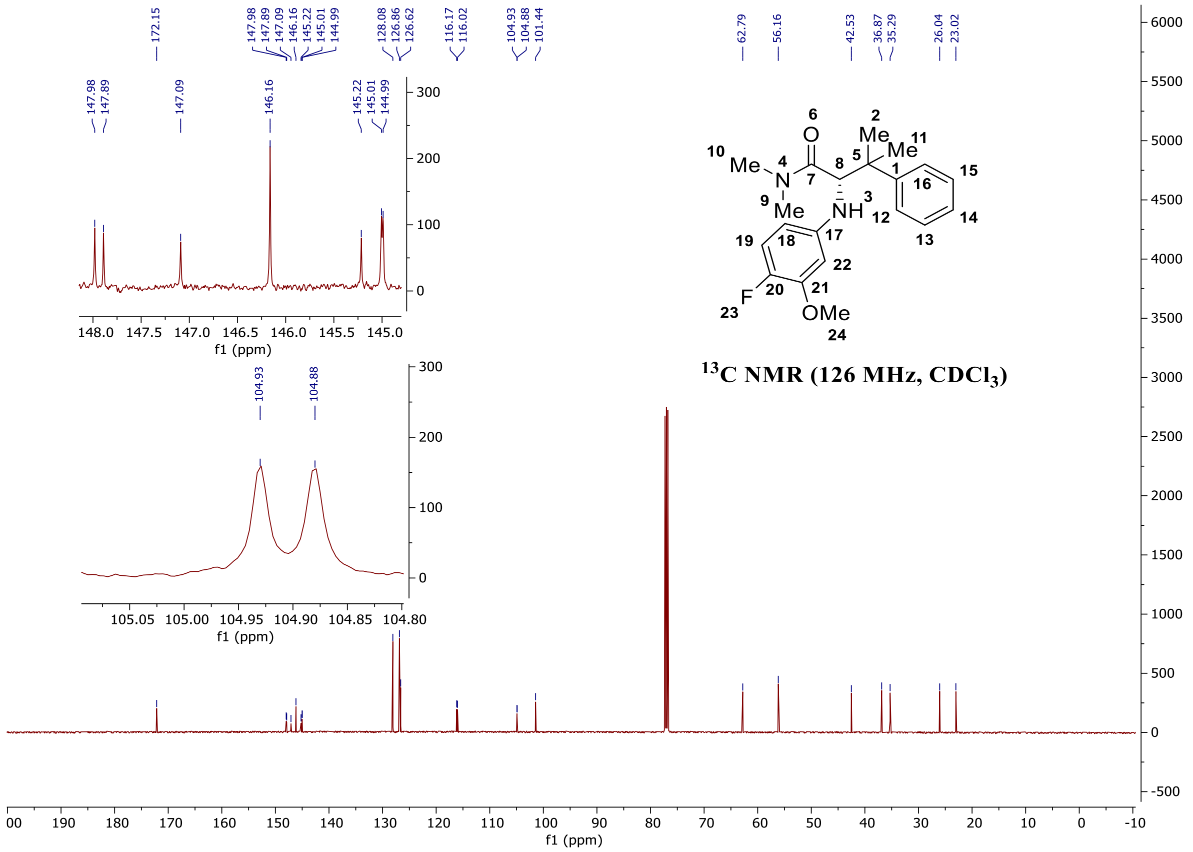

^{13}C NMR (126 MHz, CDCl$_3$)
f1 (ppm)

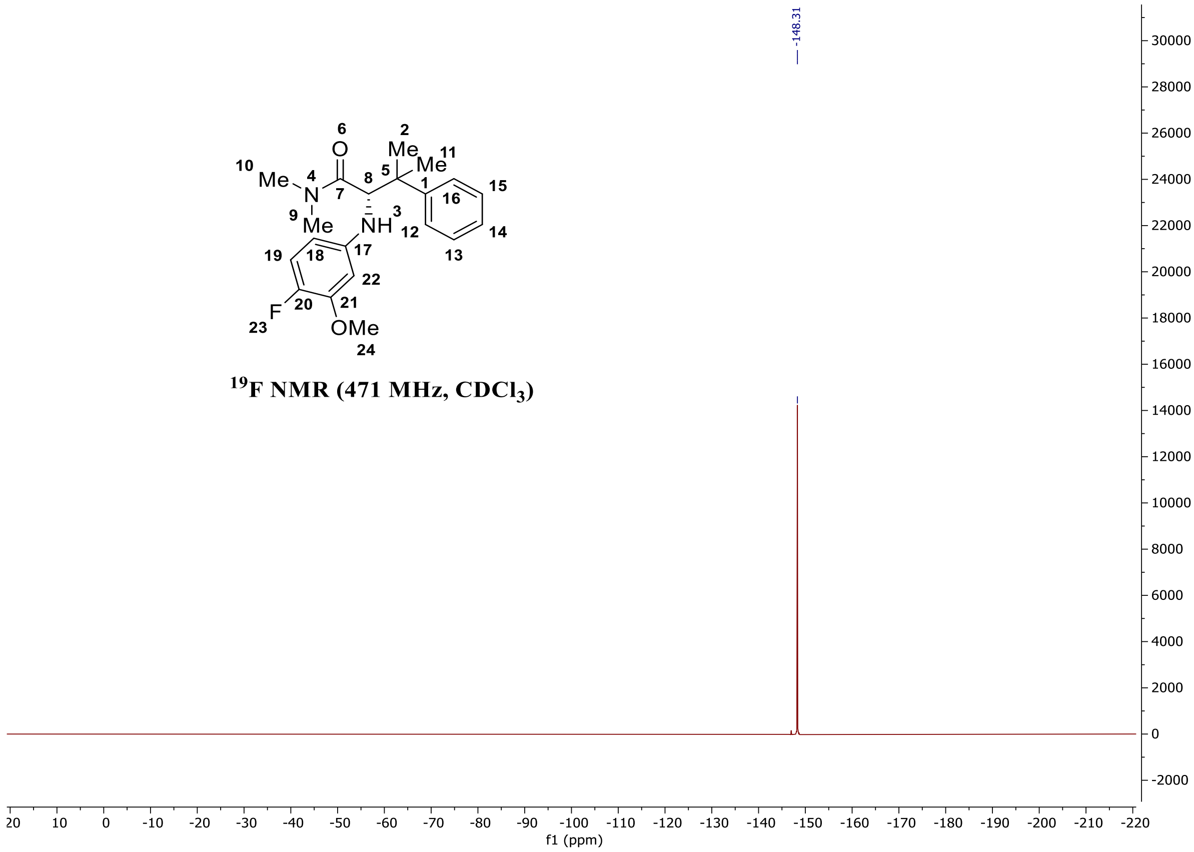

-148.31
^{19}F NMR (471 MHz, CDCl$_3$)
f1 (ppm)

(*S*)-2-((3-Fluoro-4-hydroxyphenyl)amino)-*N*,*N*,3-trimethyl-3-phenylbutanamide (3ga)

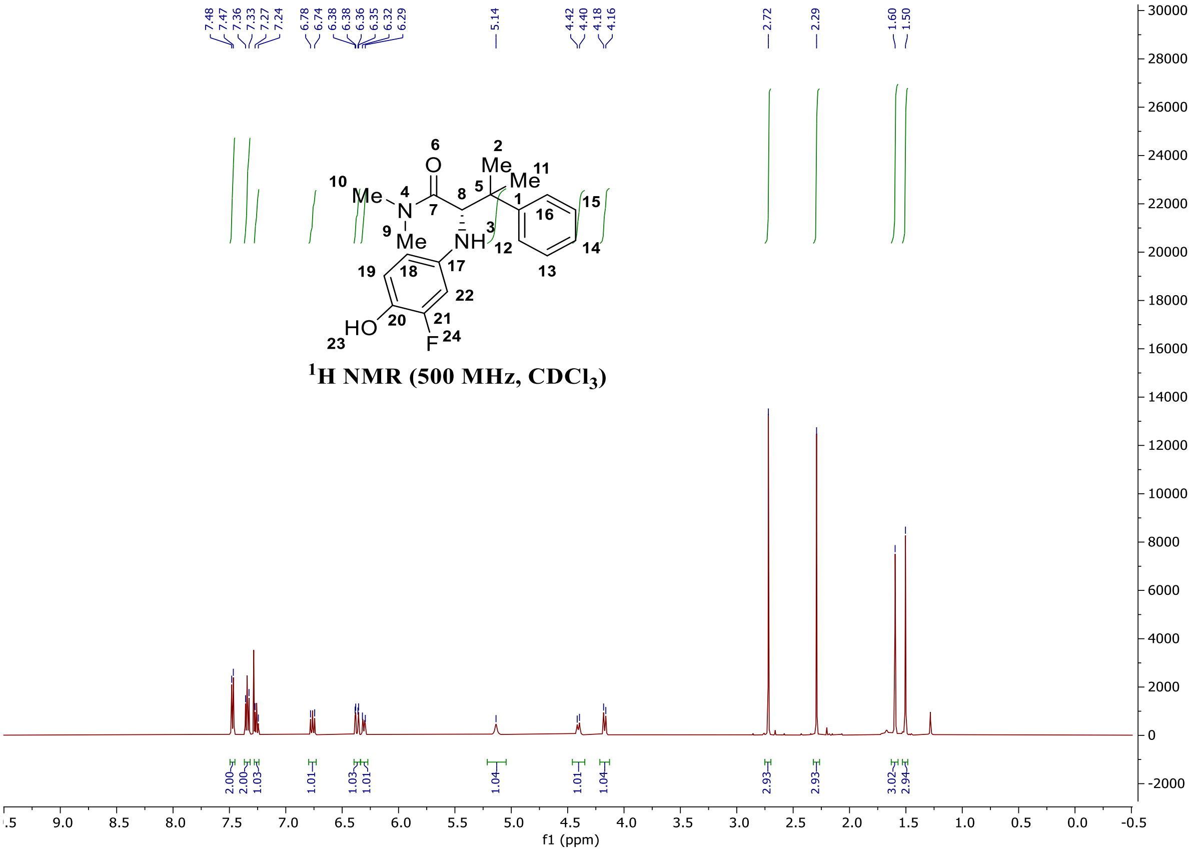

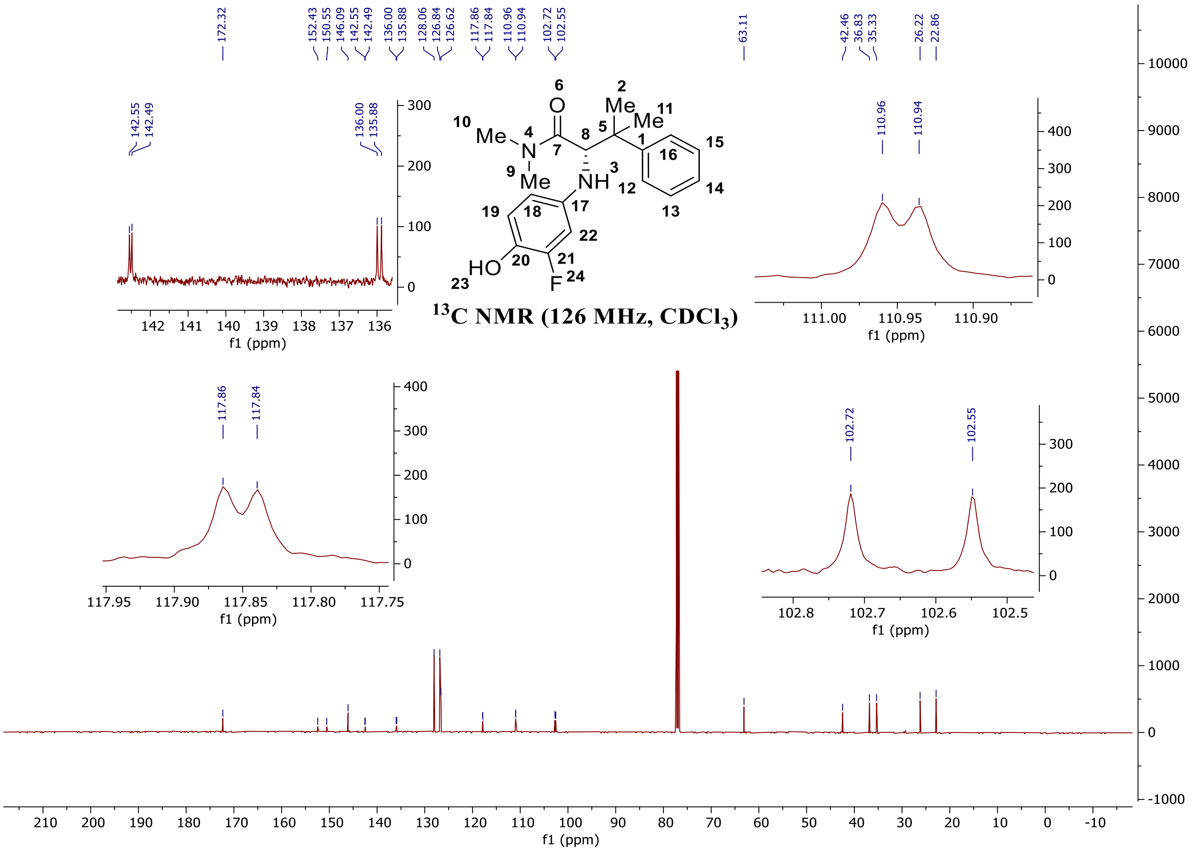

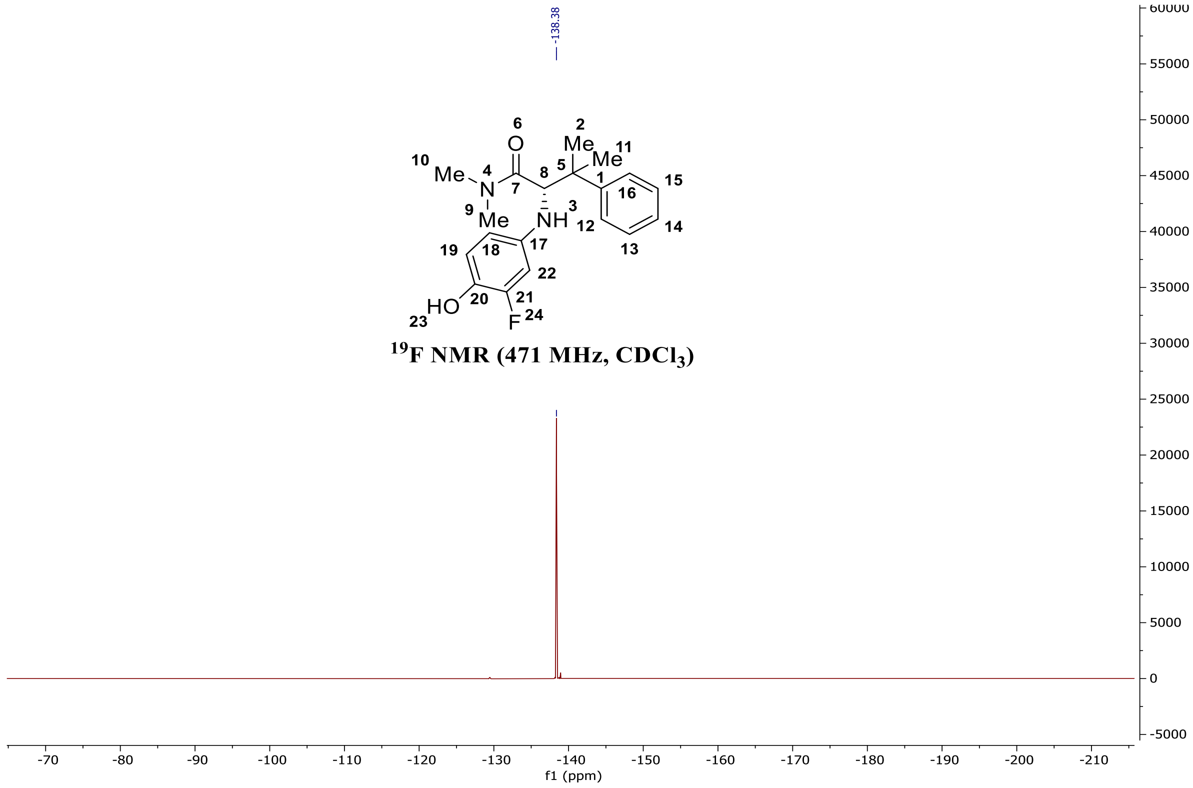

(*S*)-*N*,*N*-Dibenzyl-2-((4-hydroxyphenyl)amino)-3-methyl-3-phenylbutanamide (3ha)

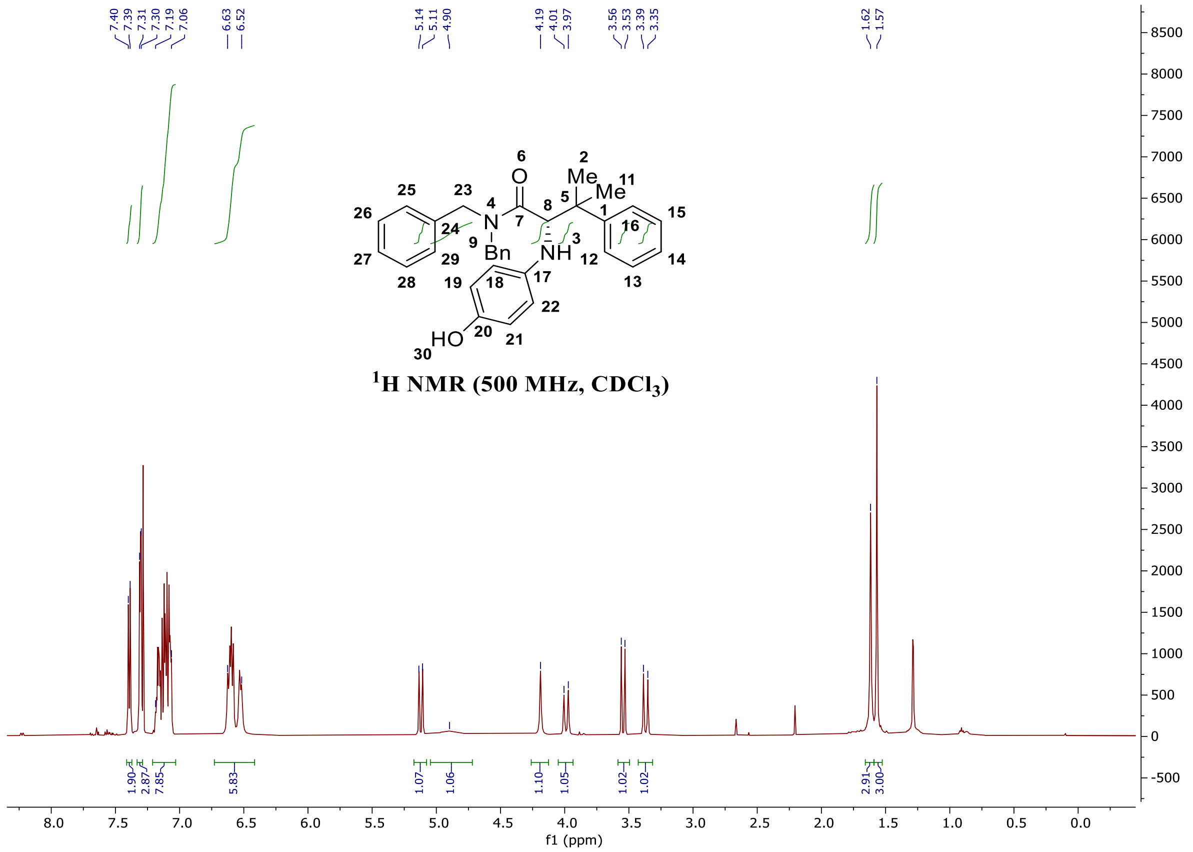

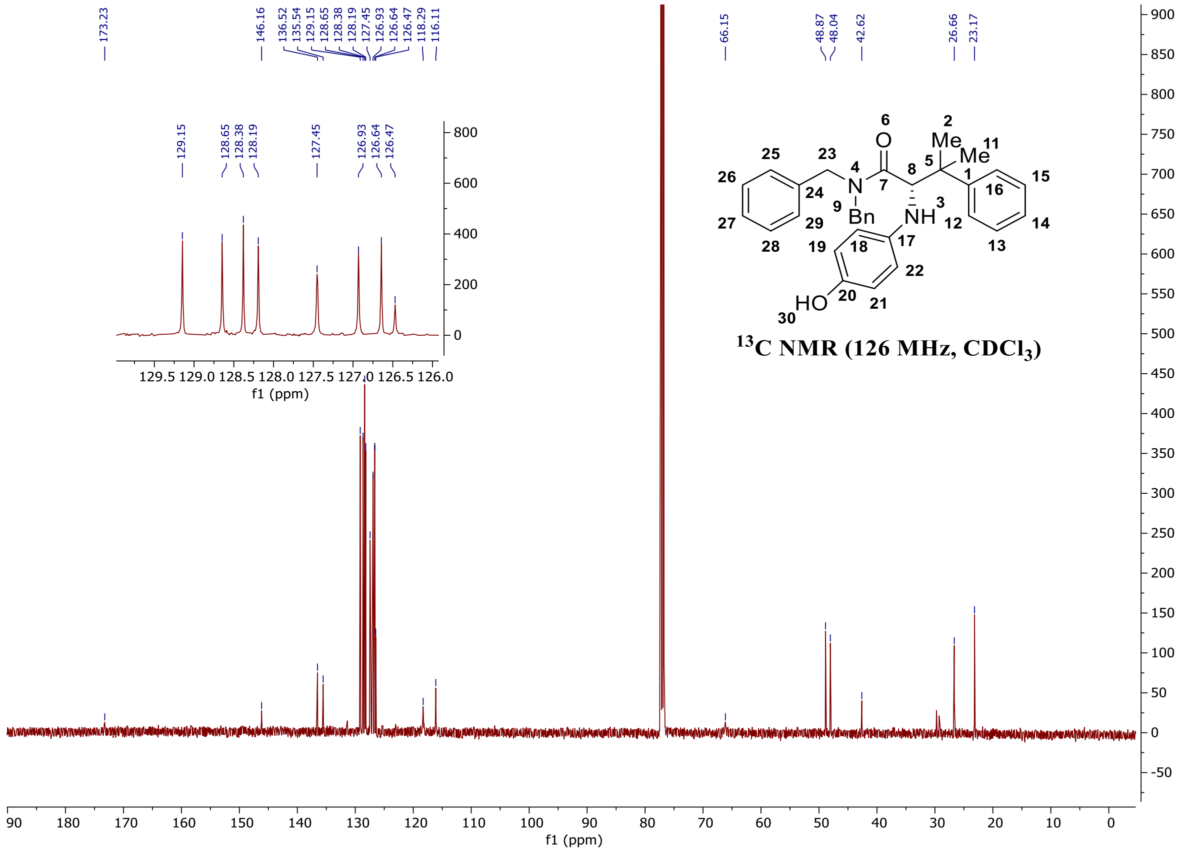

(*S*)-2-((4-Hydroxyphenyl)amino)-3-methyl-3-phenyl-1-(pyrrolidin-1-yl)butan-1-one (3ia)

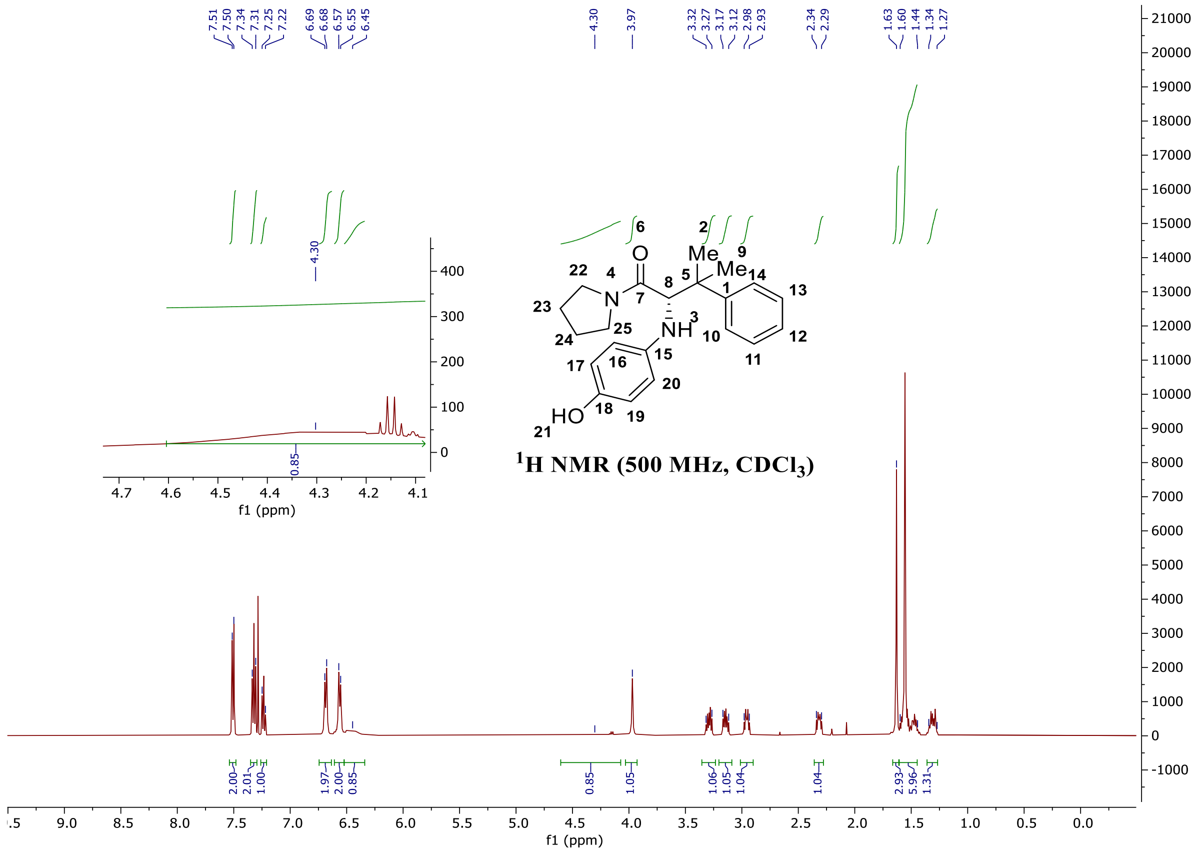

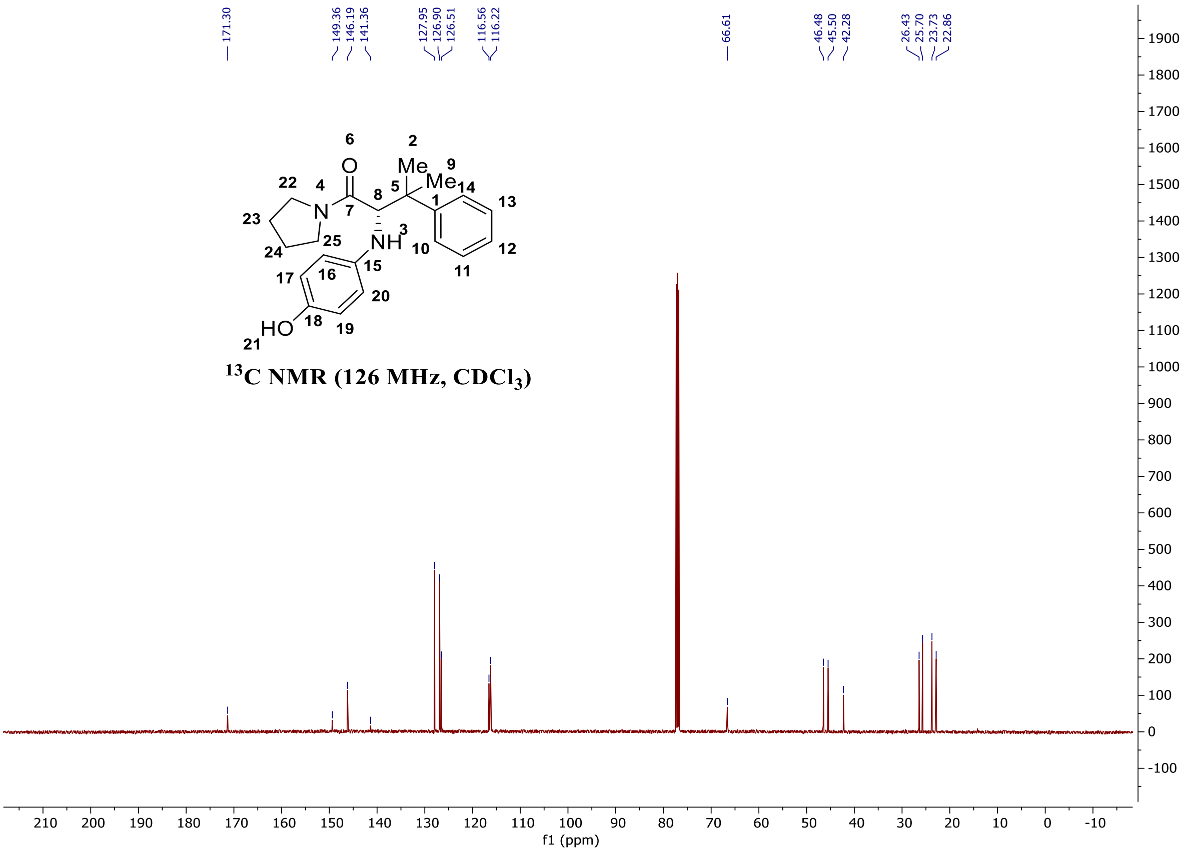

(*S*)-2-((4-Hydroxyphenyl)amino)-3-methyl-3-phenyl-1-(piperidin-1-yl)butan-1-one (3ja)

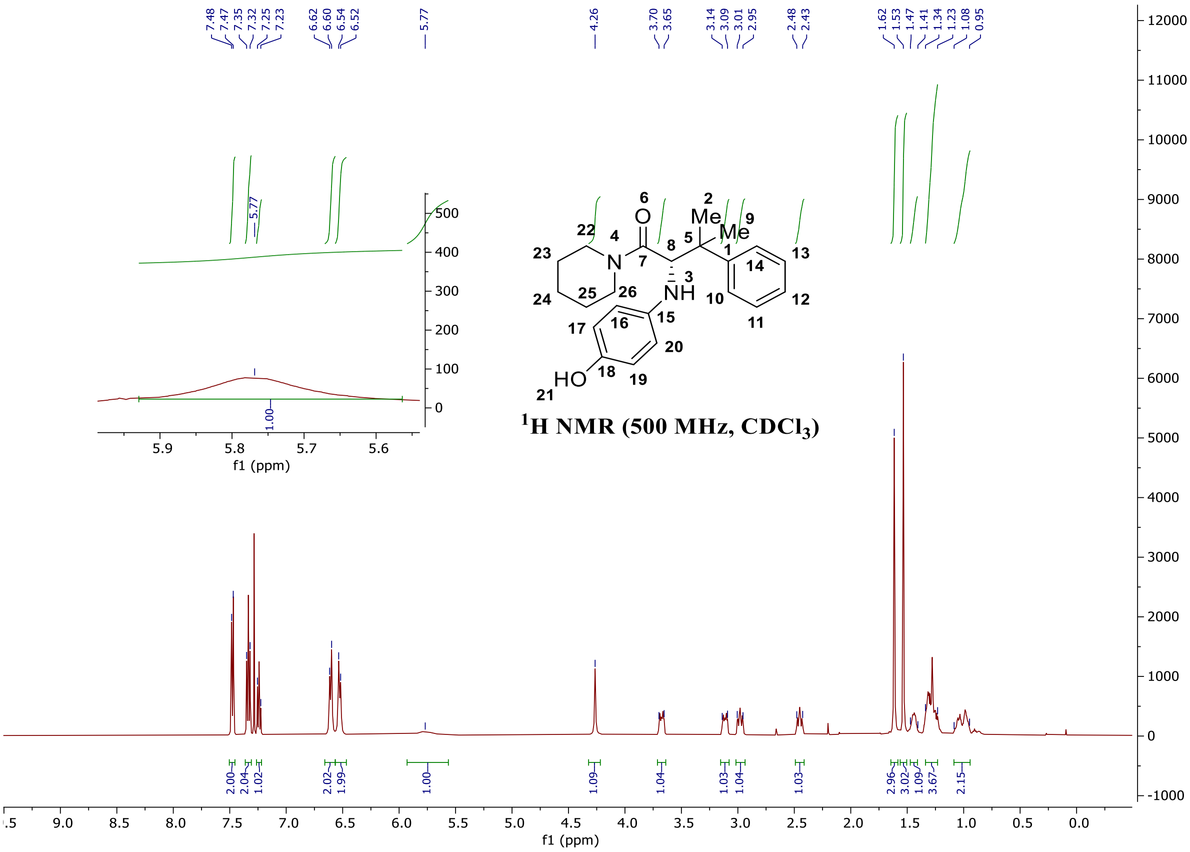

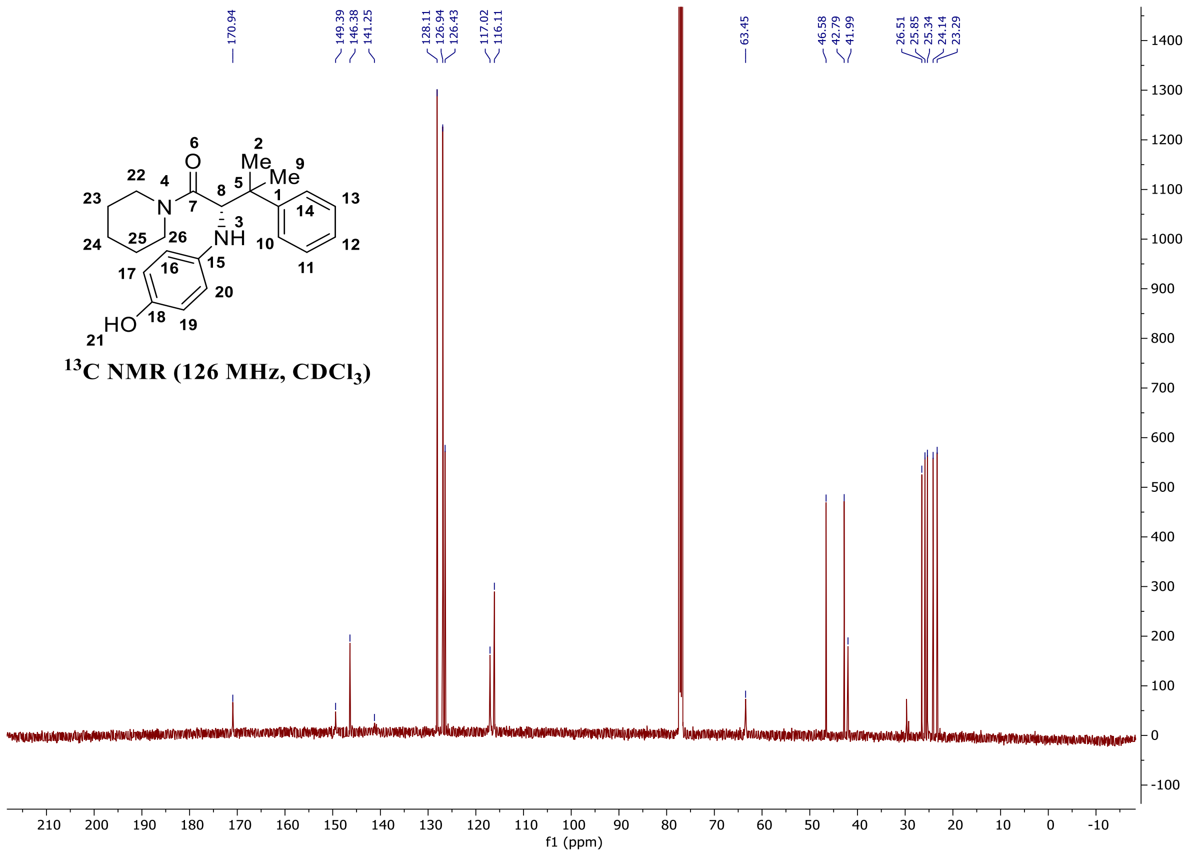

(*S*)-2-((4-Hydroxyphenyl)amino)-3-methyl-1-morpholino-3-phenylbutan-1-one (3ka)

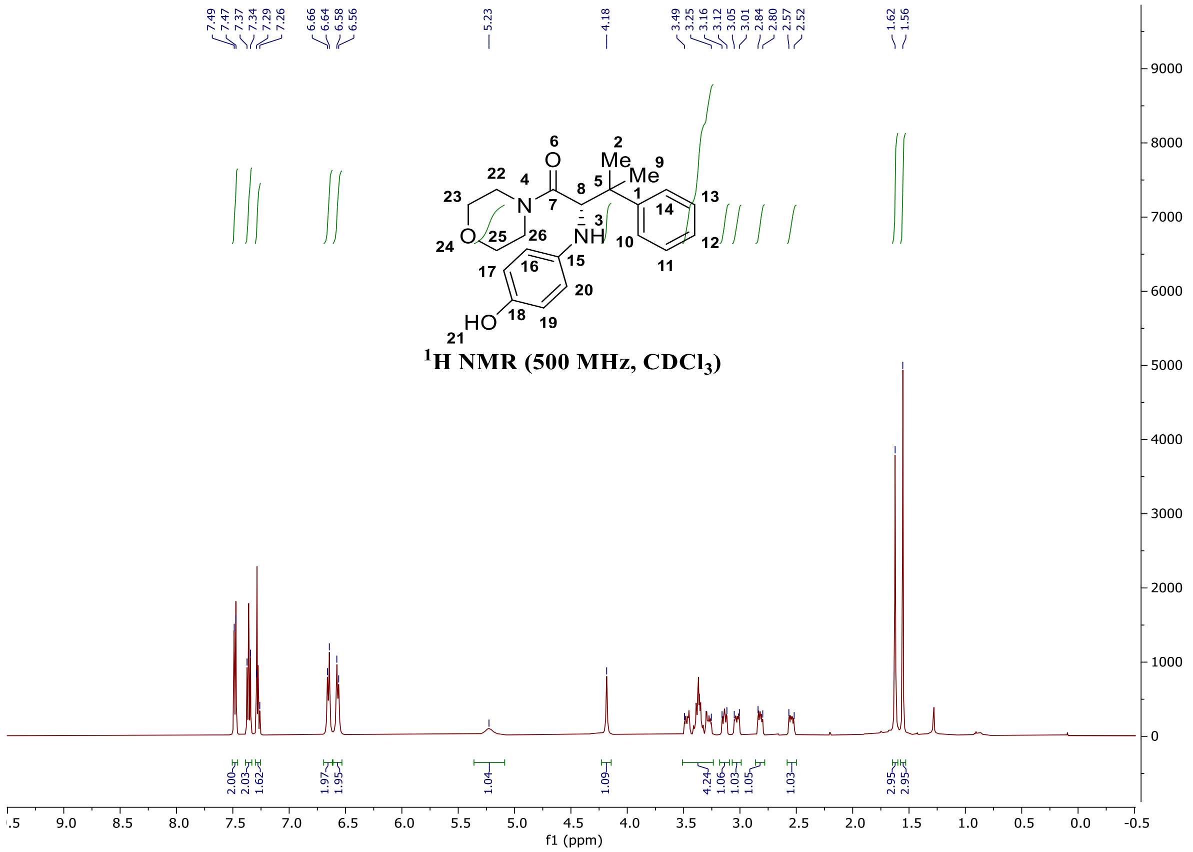

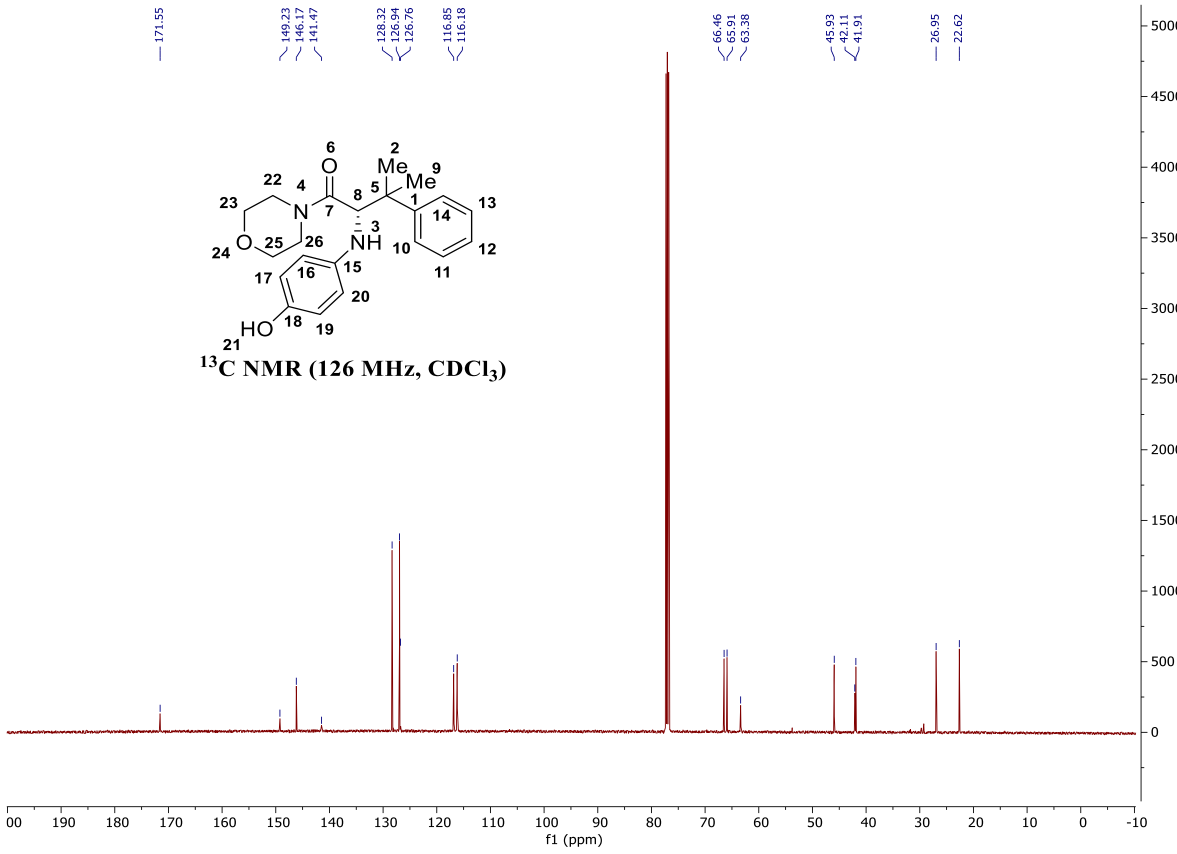

(*S*)-2-((4-Hydroxyphenyl)amino)-3-methyl-*N*,3-diphenylbutanamide (3la)

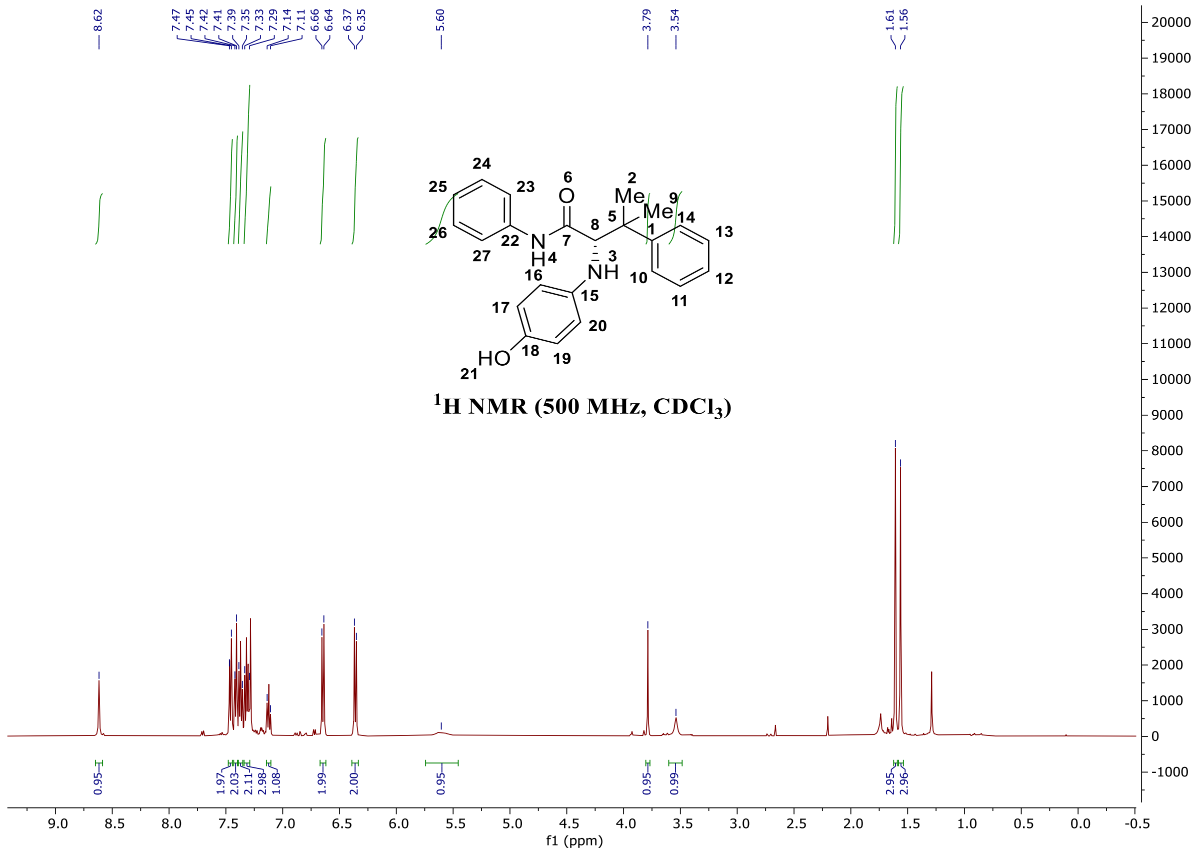

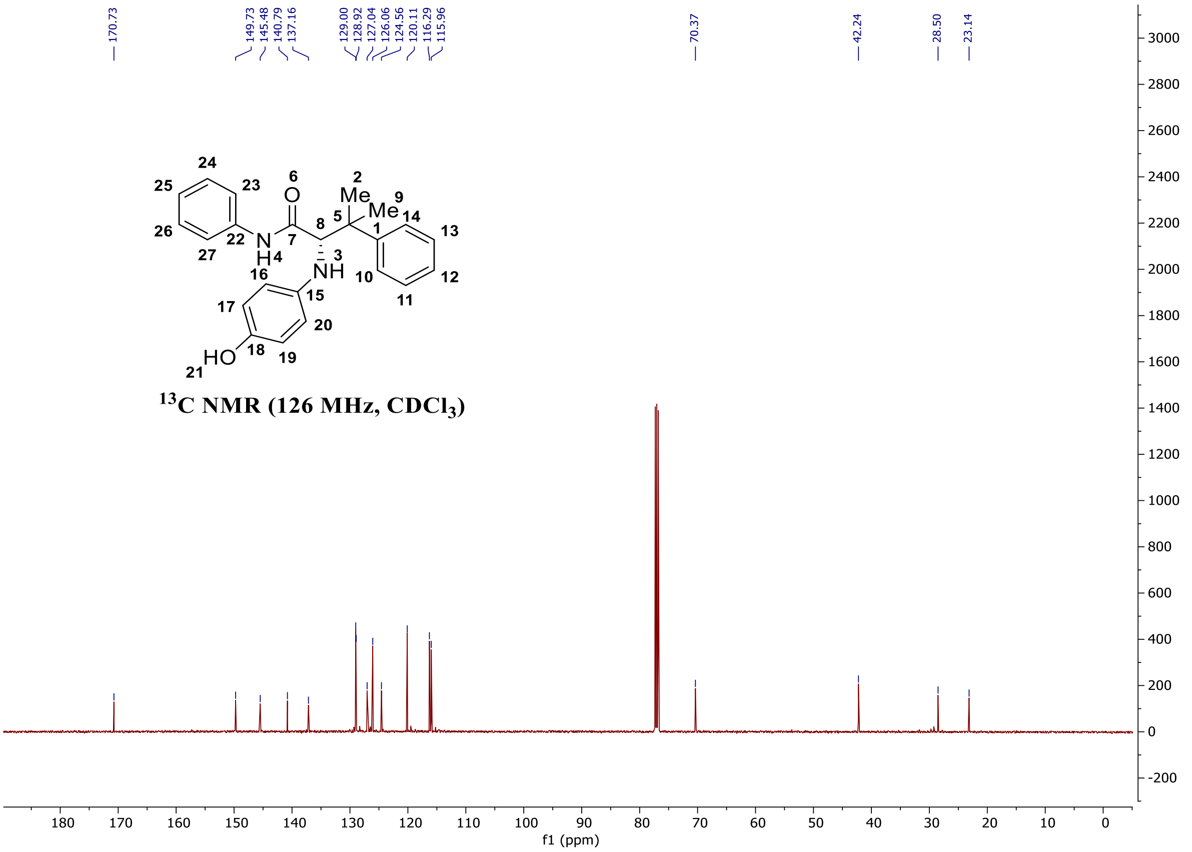

(*S*)-2-((4-Hydroxyphenyl)amino)-3-methyl-1,3-diphenylbutan-1-one (3ma)

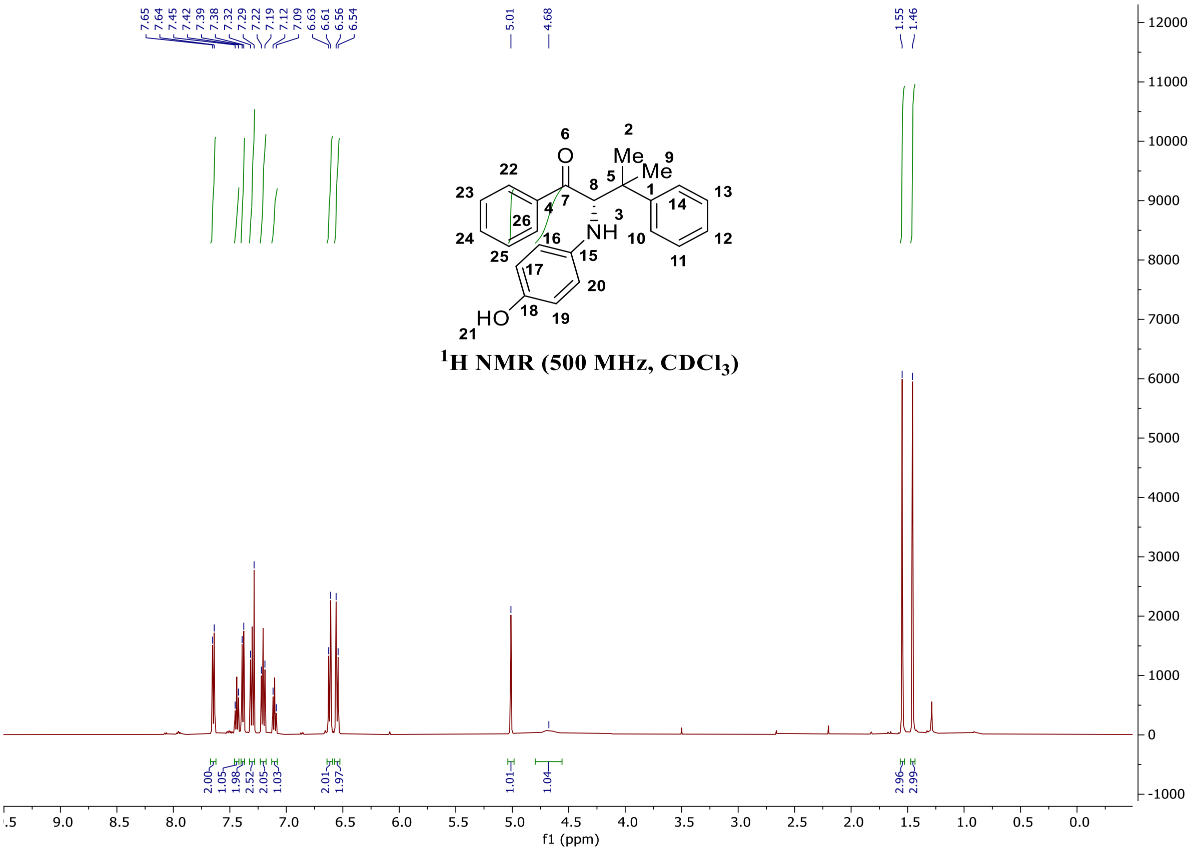

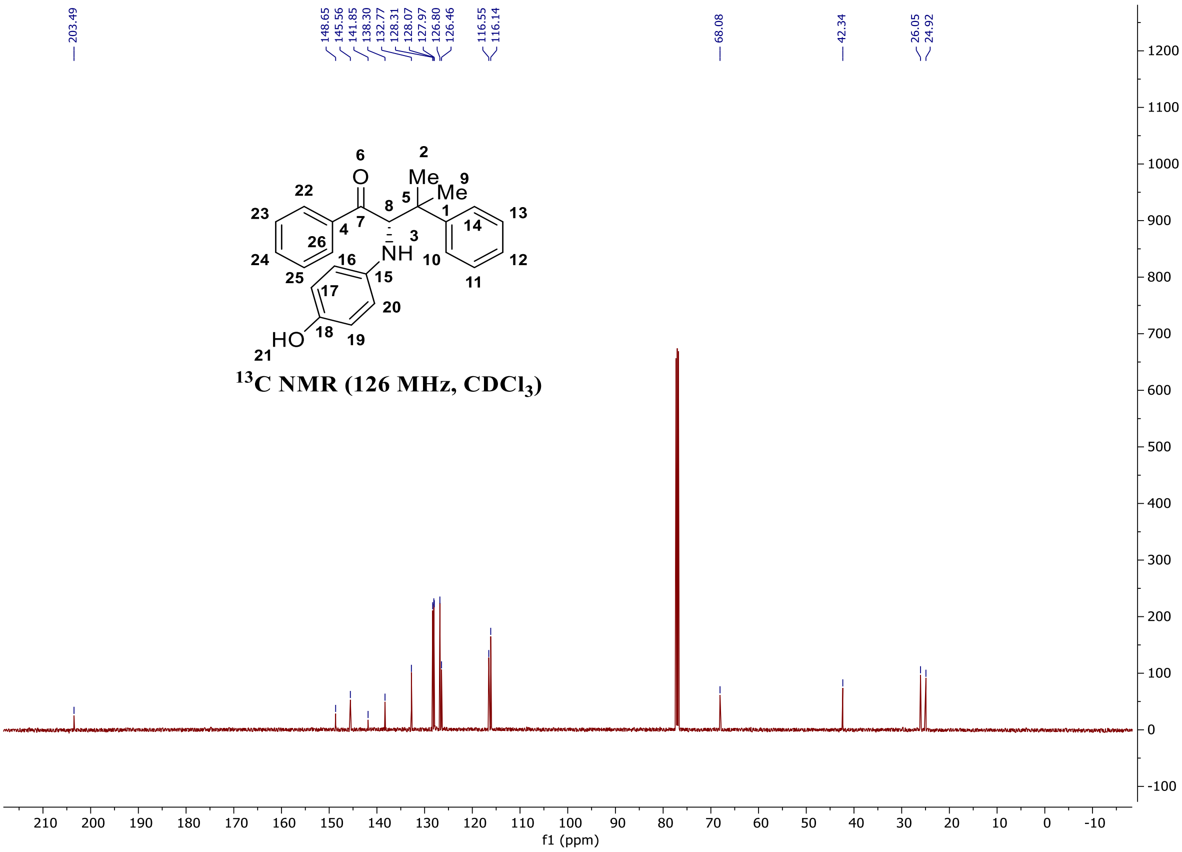

(*S*)-2-((4-Hydroxyphenyl)amino)-3-methyl-1-(pyrrolidin-1-yl)-3-(*p*-tolyl)butan-1-one (3ib)

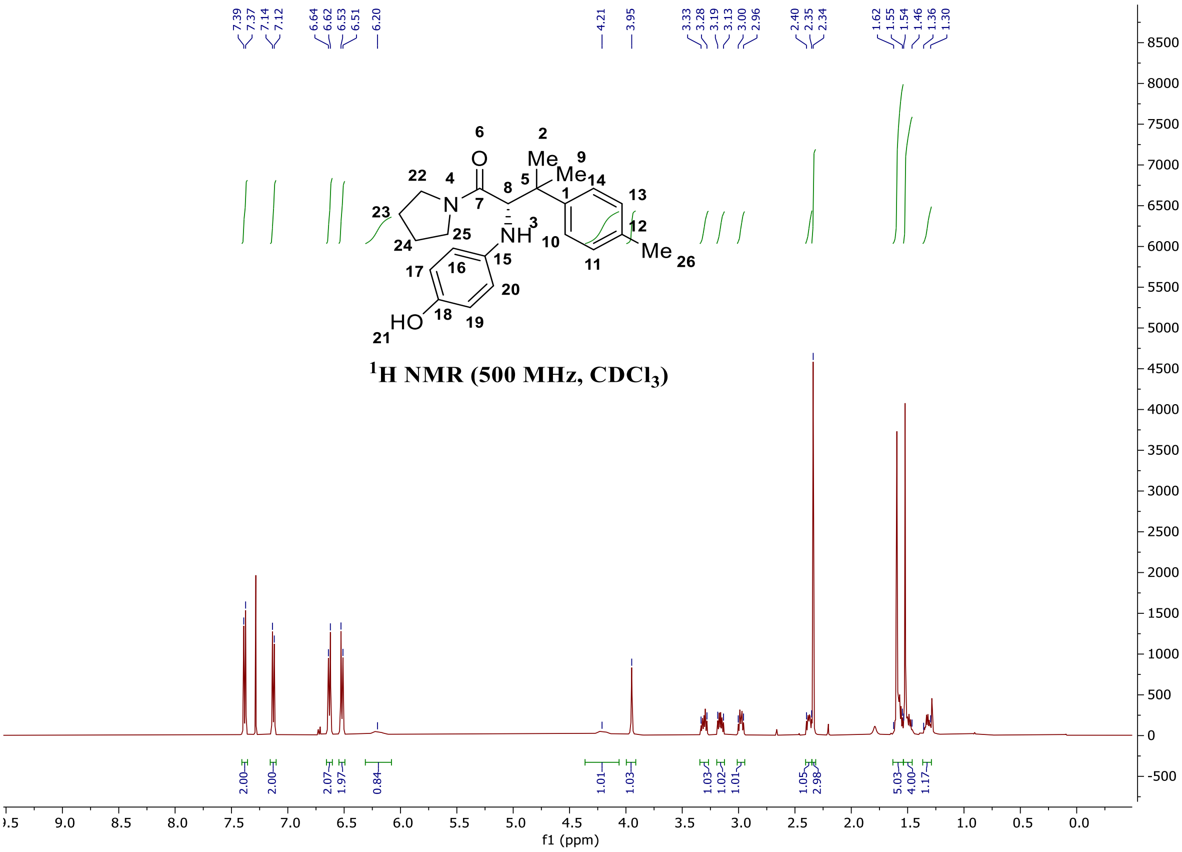

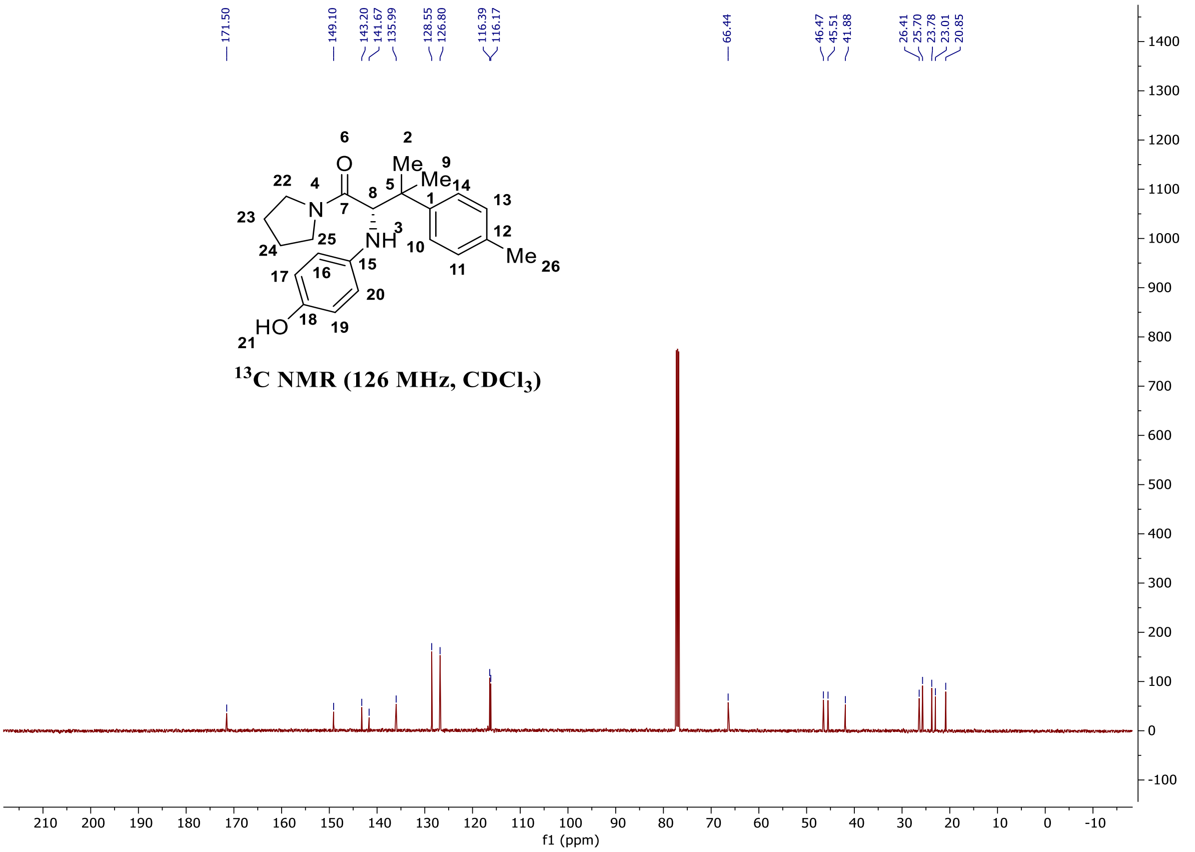

(*S*)-2-((4-Hydroxyphenyl)amino)-3-(4-methoxyphenyl)-3-methyl-1-(pyrrolidin-1-yl)butan-1-one (3ic)

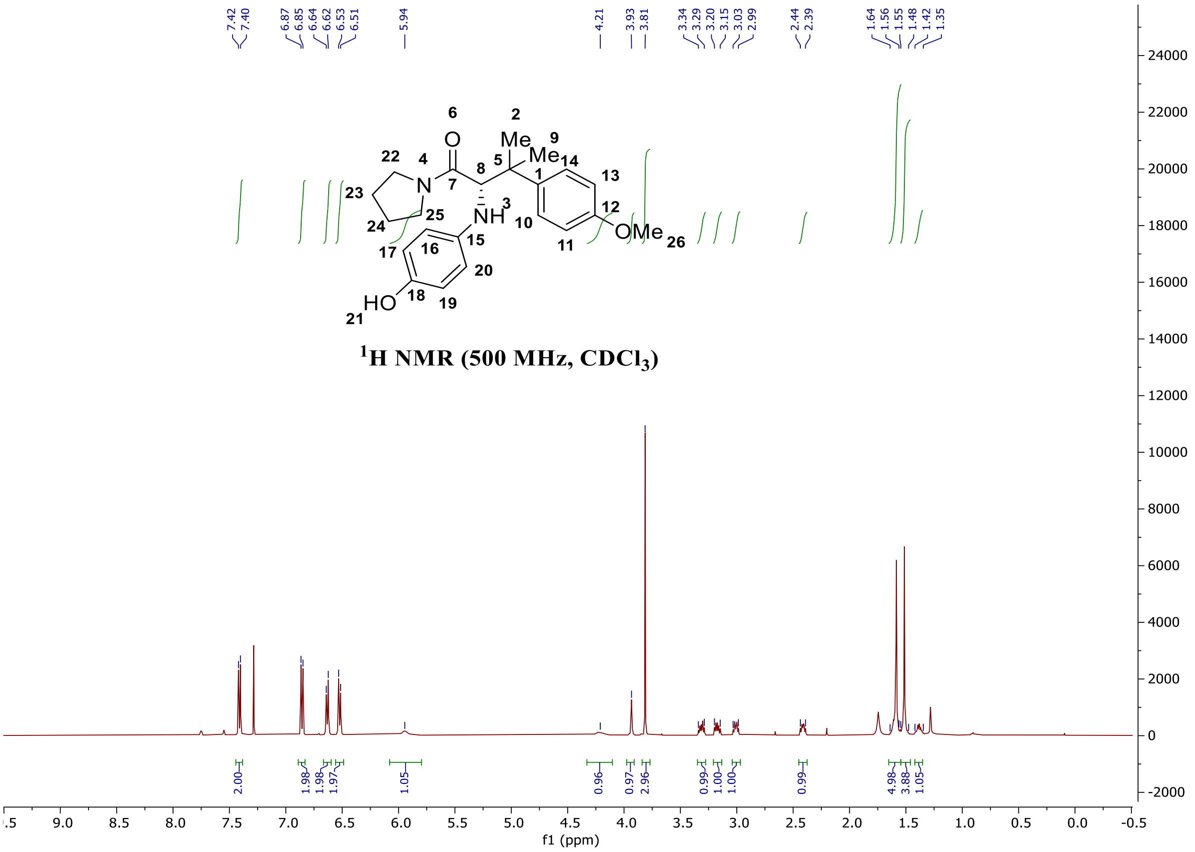

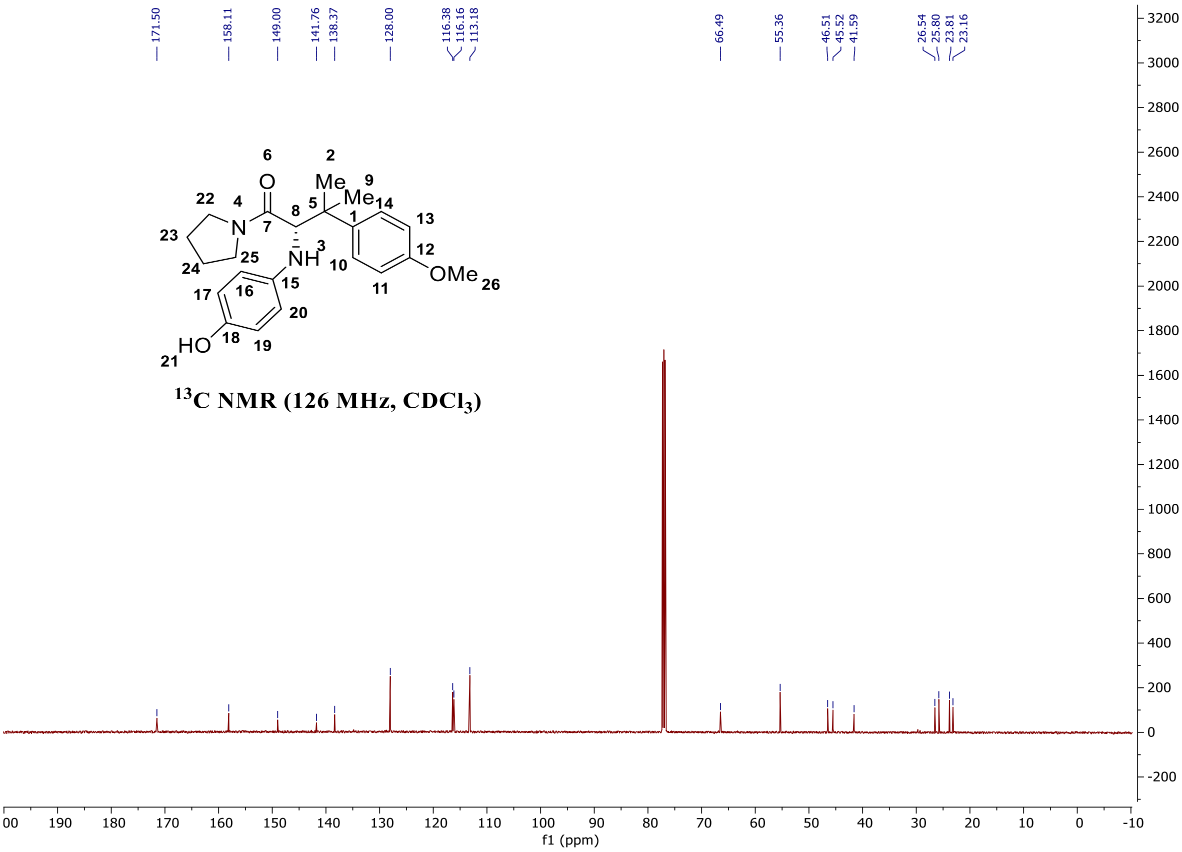

(*S*)-3-(4-Hydroxyphenyl)-2-((4-hydroxyphenyl)amino)-3-methyl-1-(pyrrolidin-1-yl)butan-1-one (3id)

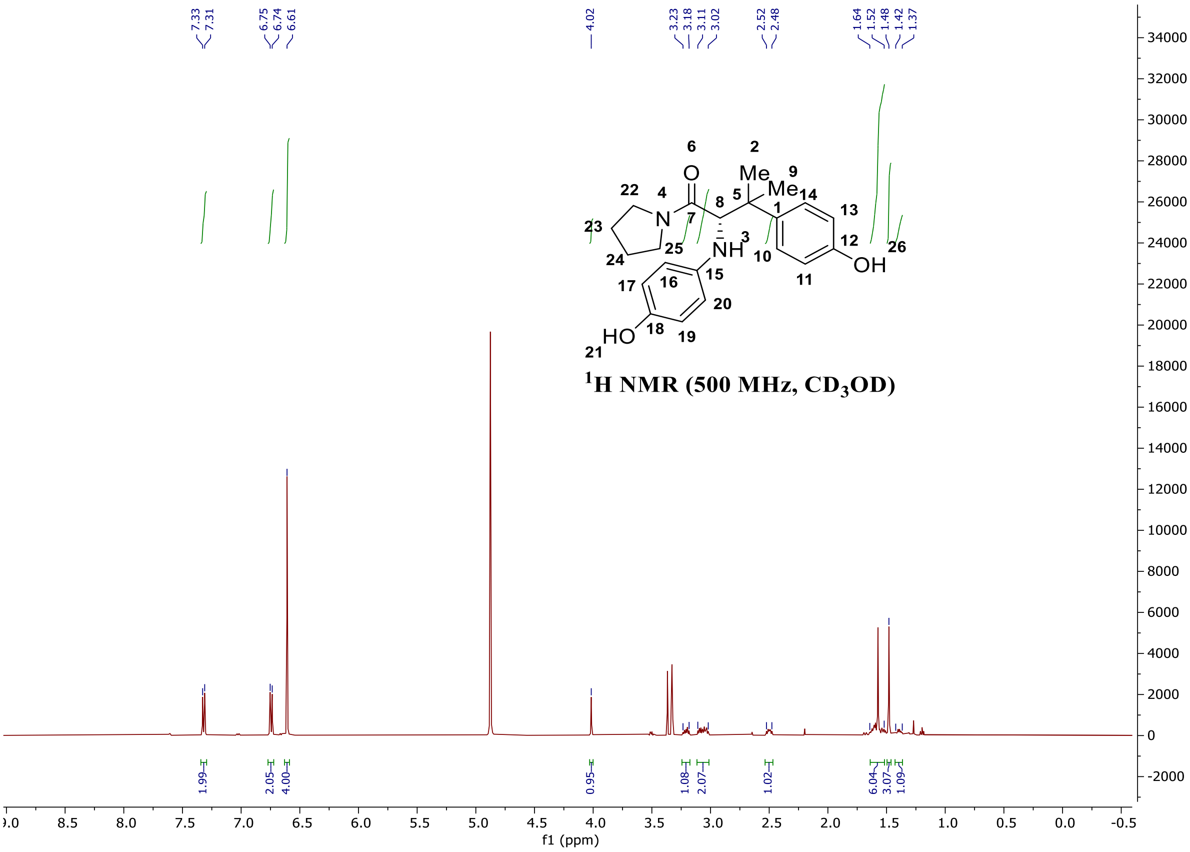

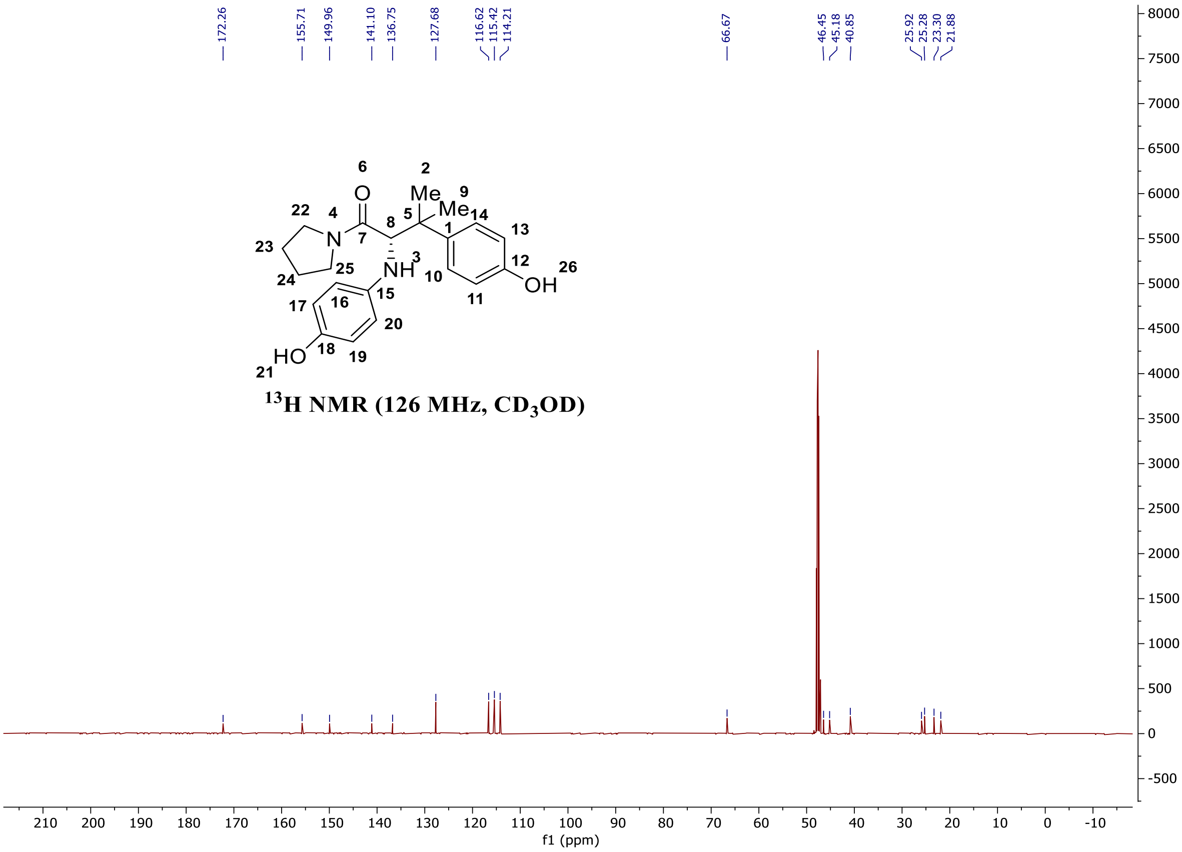

(*S*)-3-(4-Fluorophenyl)-2-((4-hydroxyphenyl)amino)-3-methyl-1-(pyrrolidin-1-yl)butan-1-one (3ie)

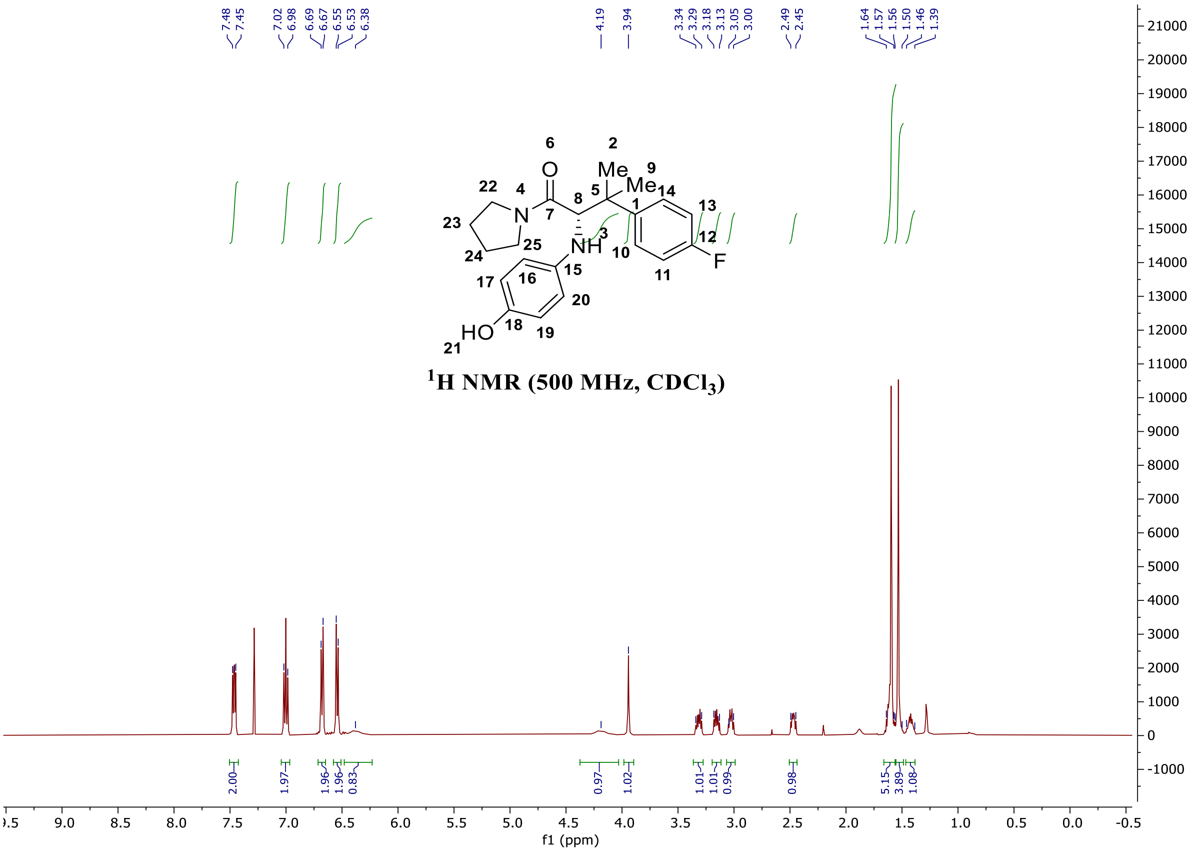

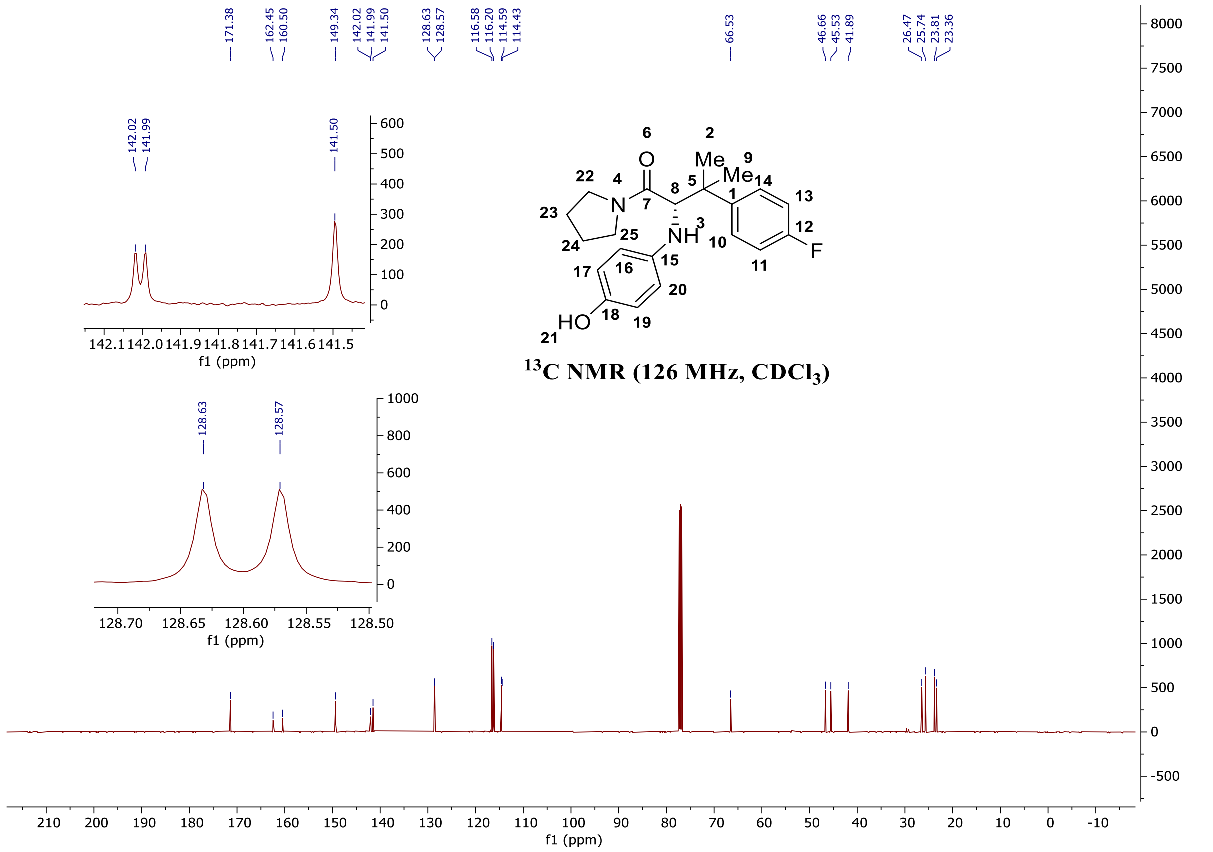
171.38
162.45
160.50
149.34
142.02
141.99
141.50
128.63
128.57
116.58
116.20
114.59
114.43
66.53
46.66
45.53
41.89
26.47
25.74
23.81
23.36
13C NMR (126 MHz, CDCl3)
f1 (ppm)

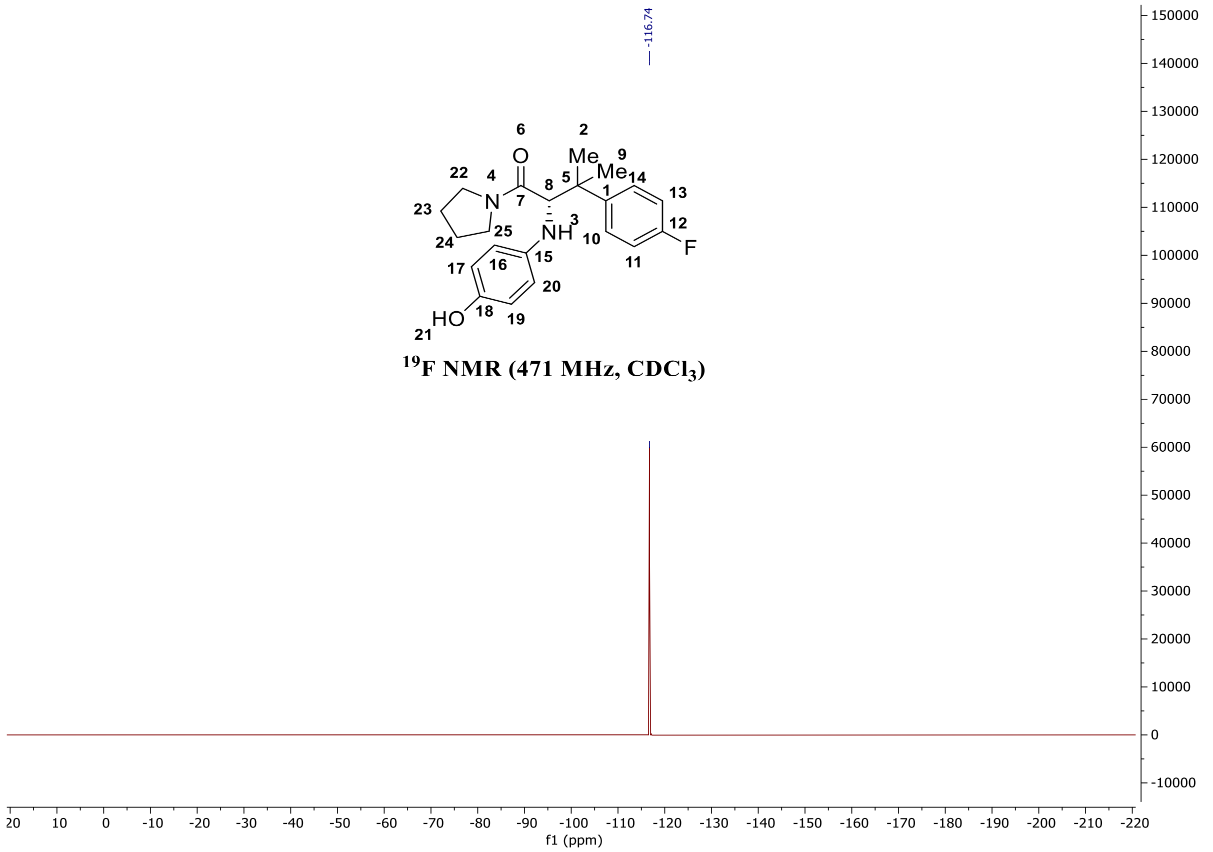
-116.74
19F NMR (471 MHz, CDCl3)
f1 (ppm)

(*S*)-3-(4-Chlorophenyl)-2-((4-hydroxyphenyl)amino)-3-methyl-1-(pyrrolidin-1-yl)butan-1-one (3if)

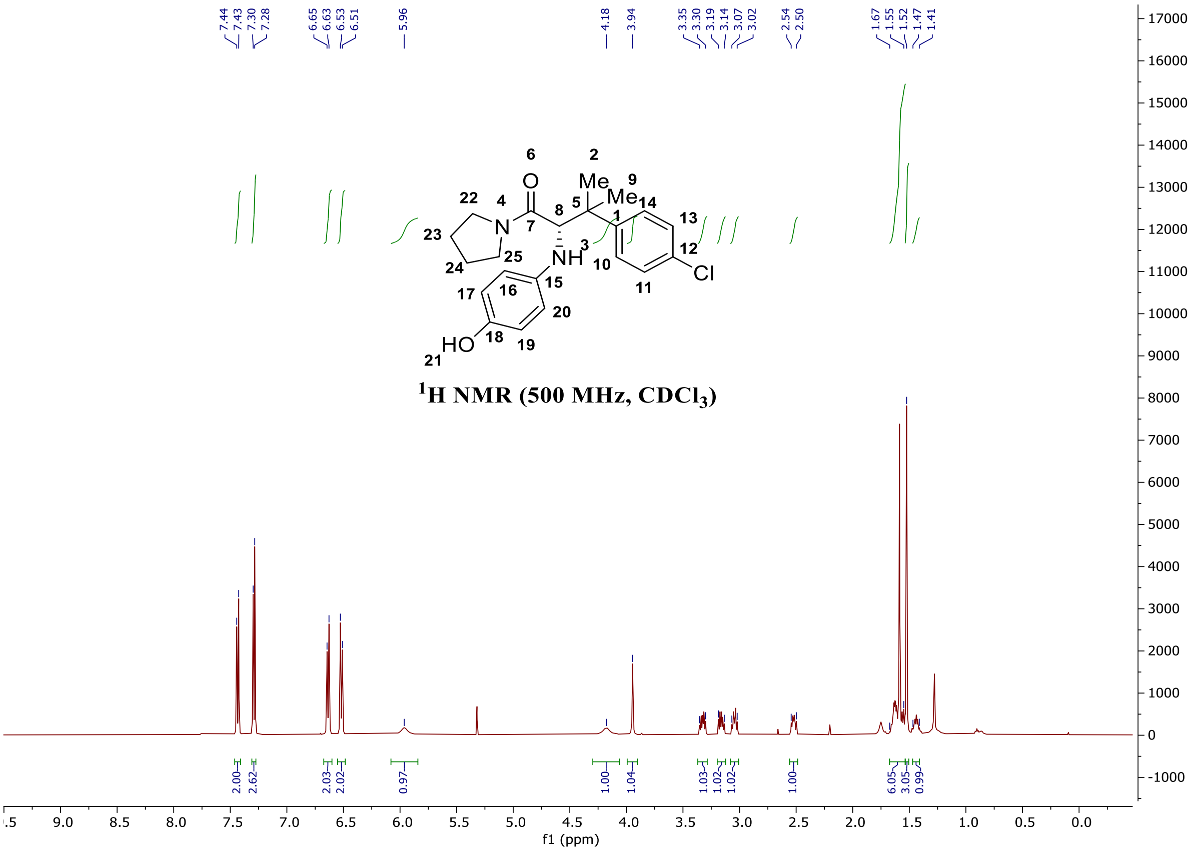

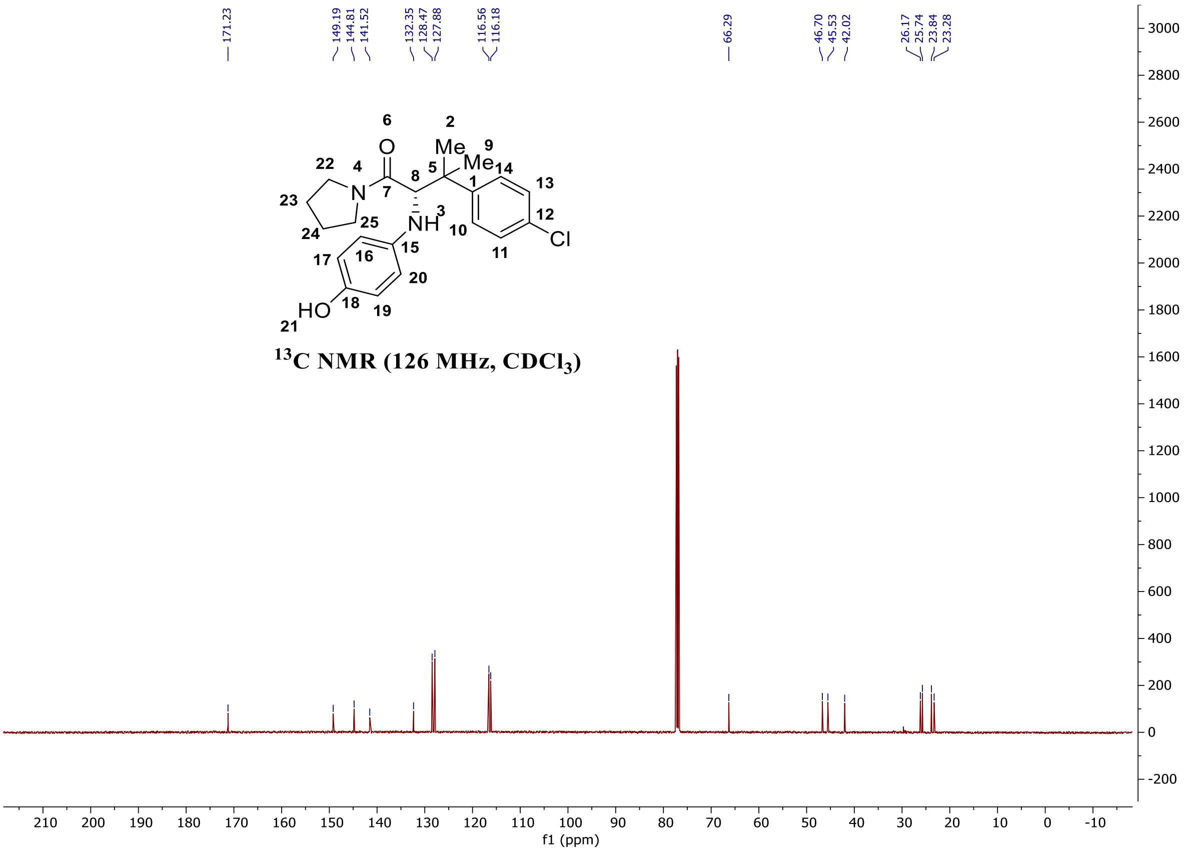

(*S*)-3-(4-Bromophenyl)-2-((4-hydroxyphenyl)amino)-3-methyl-1-(pyrrolidin-1-yl)butan-1-one (3ig)

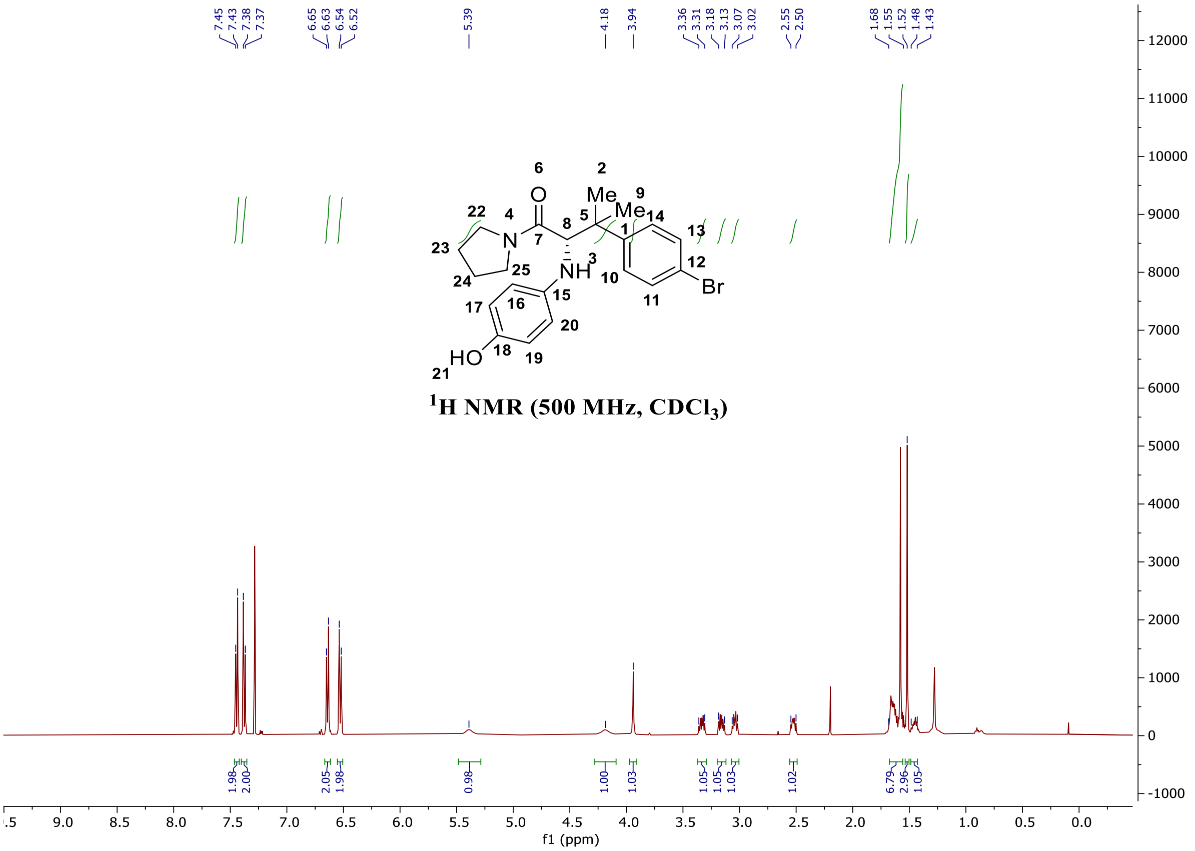

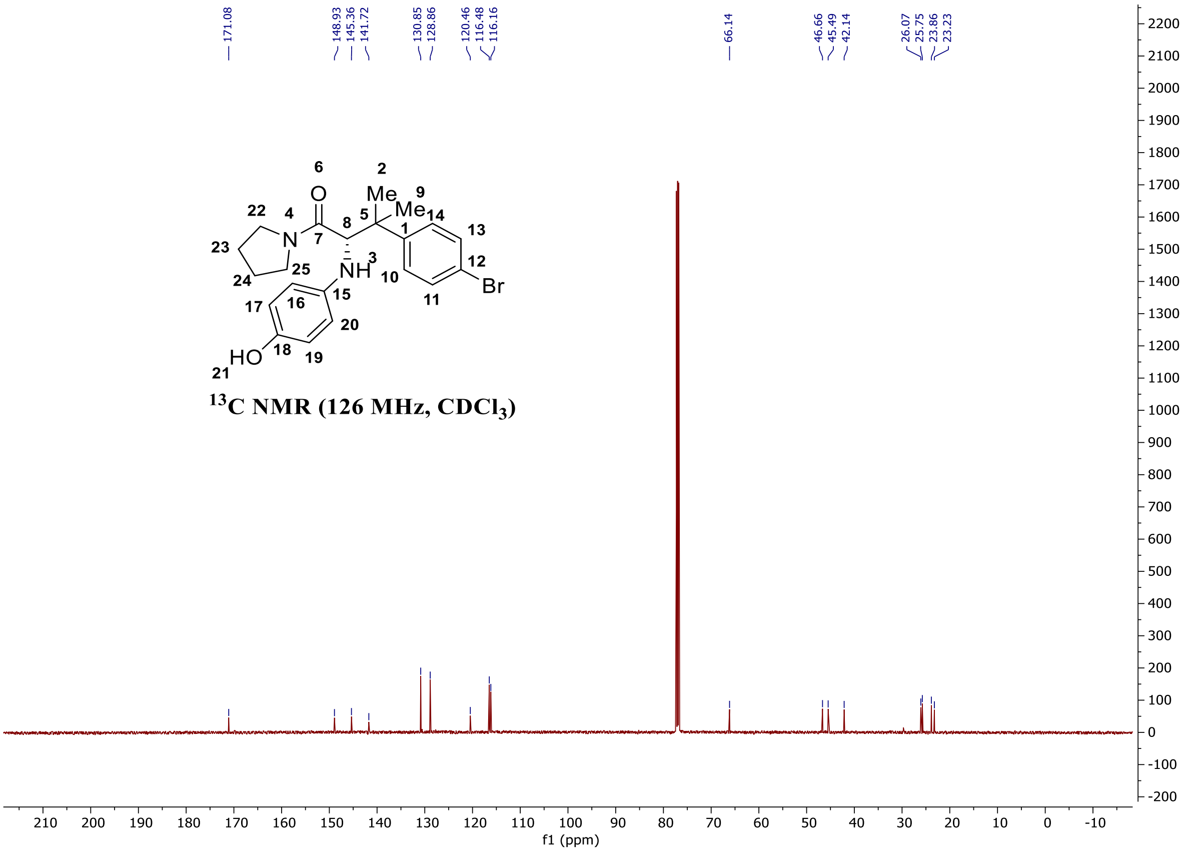

(*S*)-3-([1,1'-Biphenyl]-4-yl)-2-((4-hydroxyphenyl)amino)-3-methyl-1-(pyrrolidin-1-yl)butan-1-one (3ih)

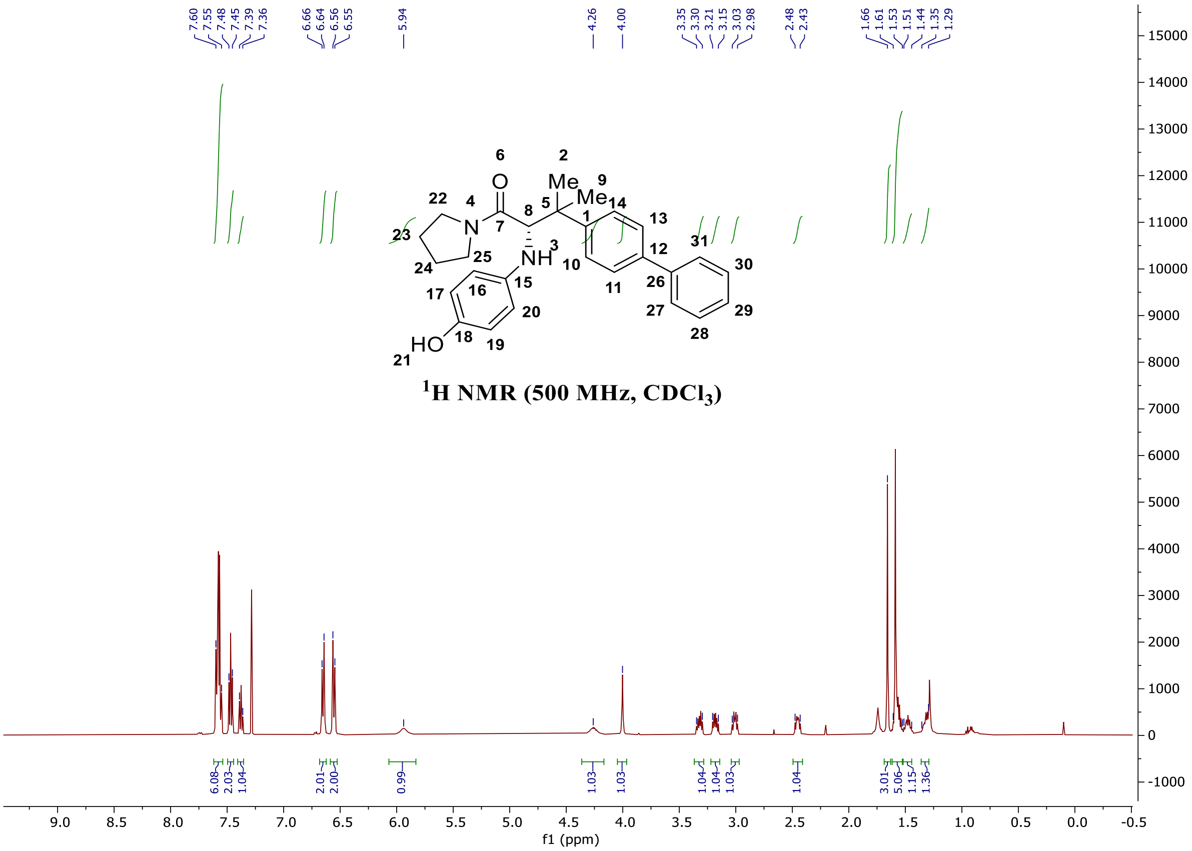

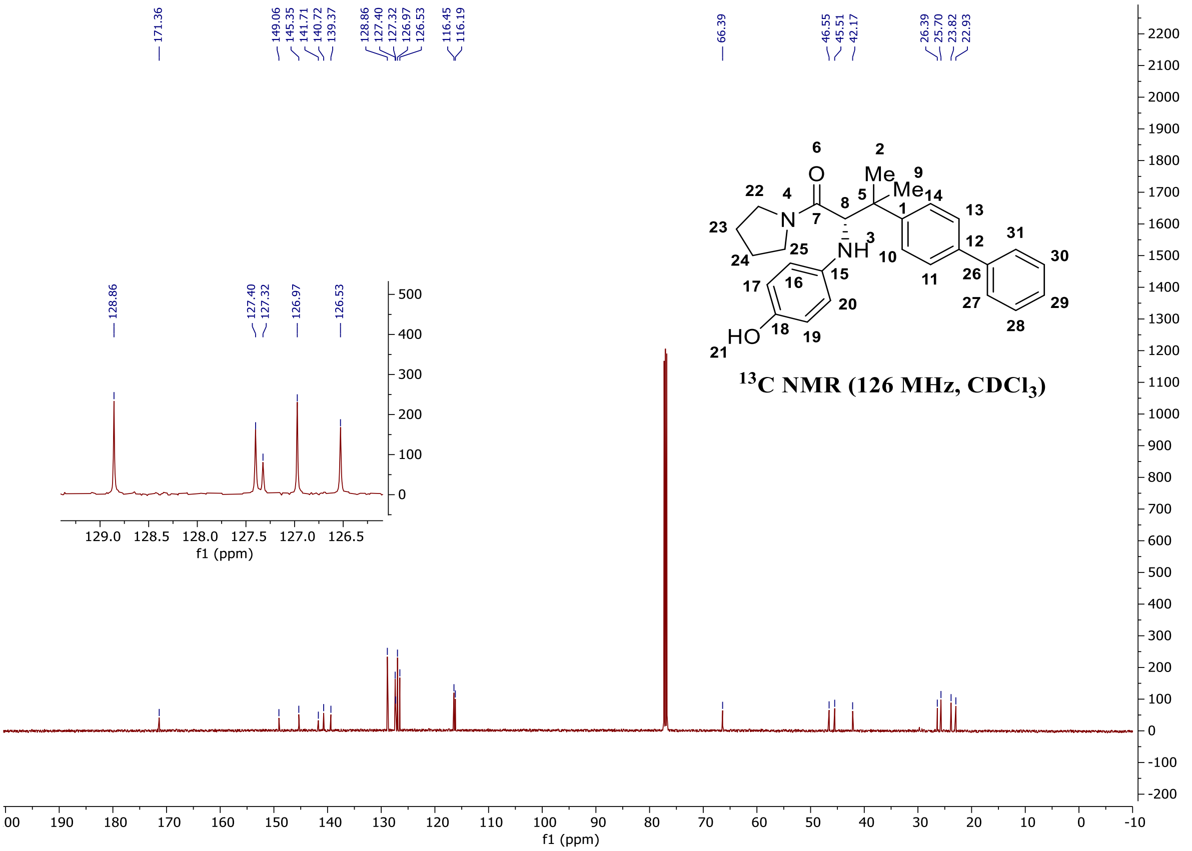

(*S*)-2-((4-Hydroxyphenyl)amino)-3-methyl-1-(pyrrolidin-1-yl)-3-(4-(4,4,5,5-tetramethyl-1,3,2-dioxaborolan-2-yl)phenyl)butan-1-one (3ii)

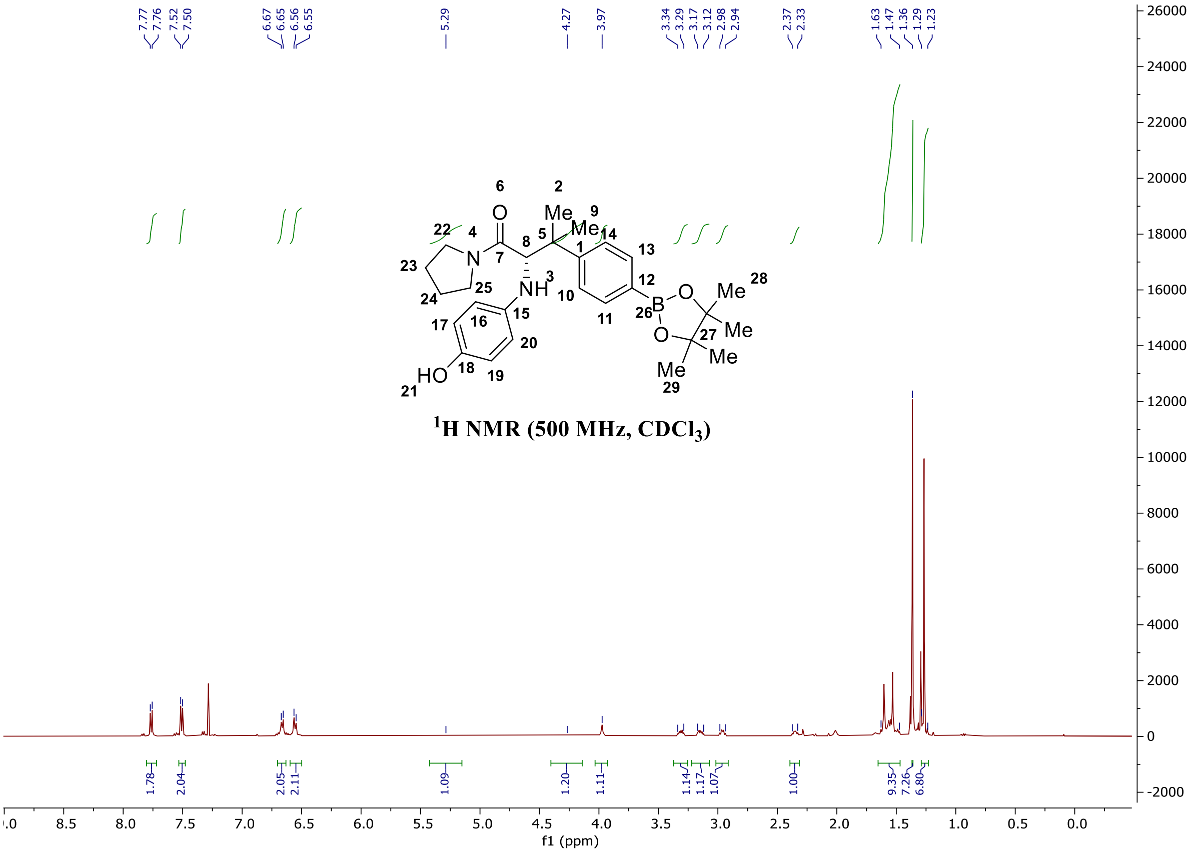

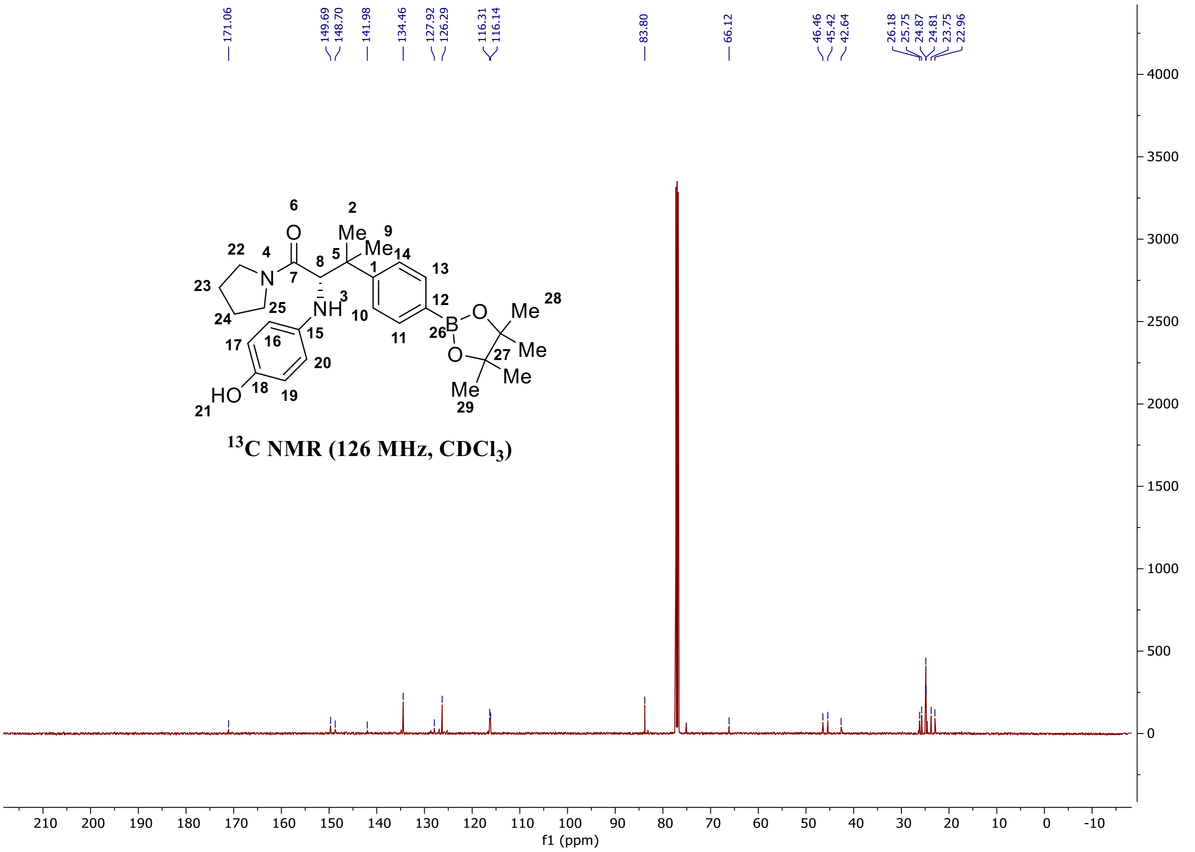

(*S*)-2-((4-Hydroxyphenyl)amino)-3-methyl-1-(pyrrolidin-1-yl)-3-(*m*-tolyl)butan-1-one (3ij)

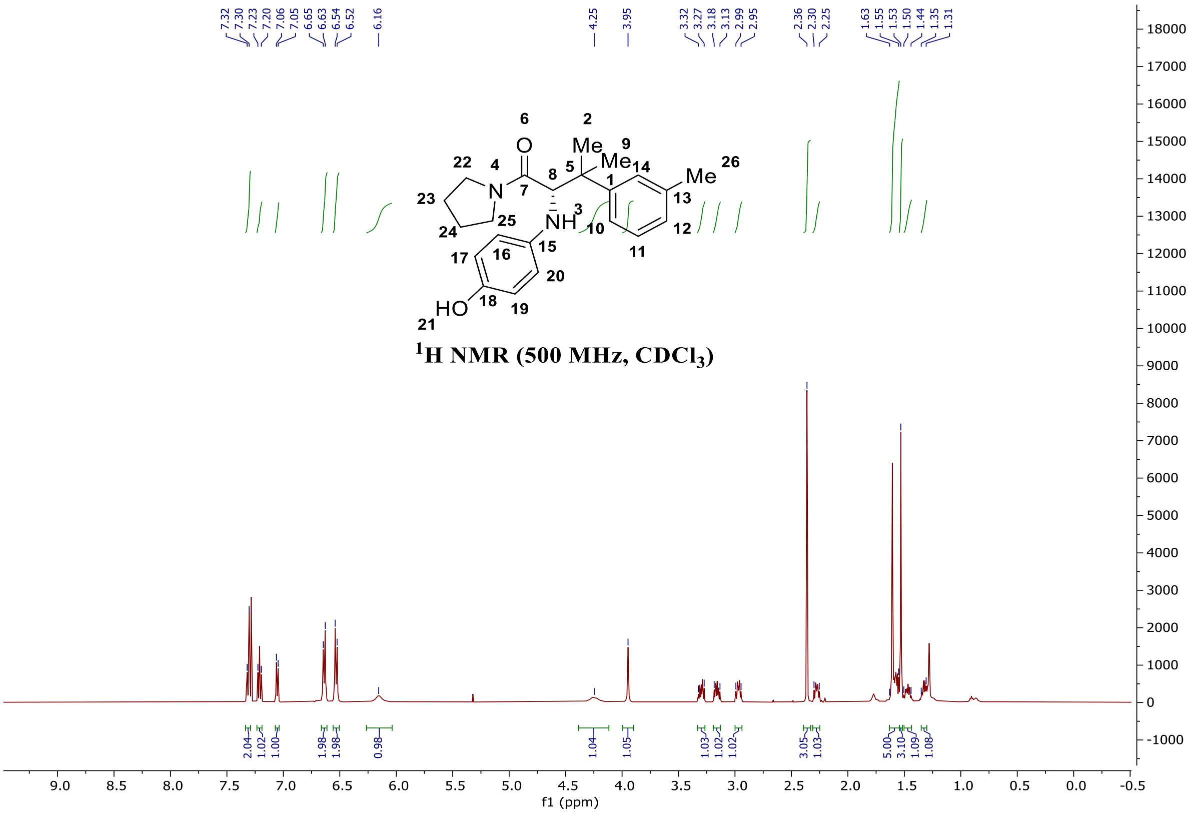

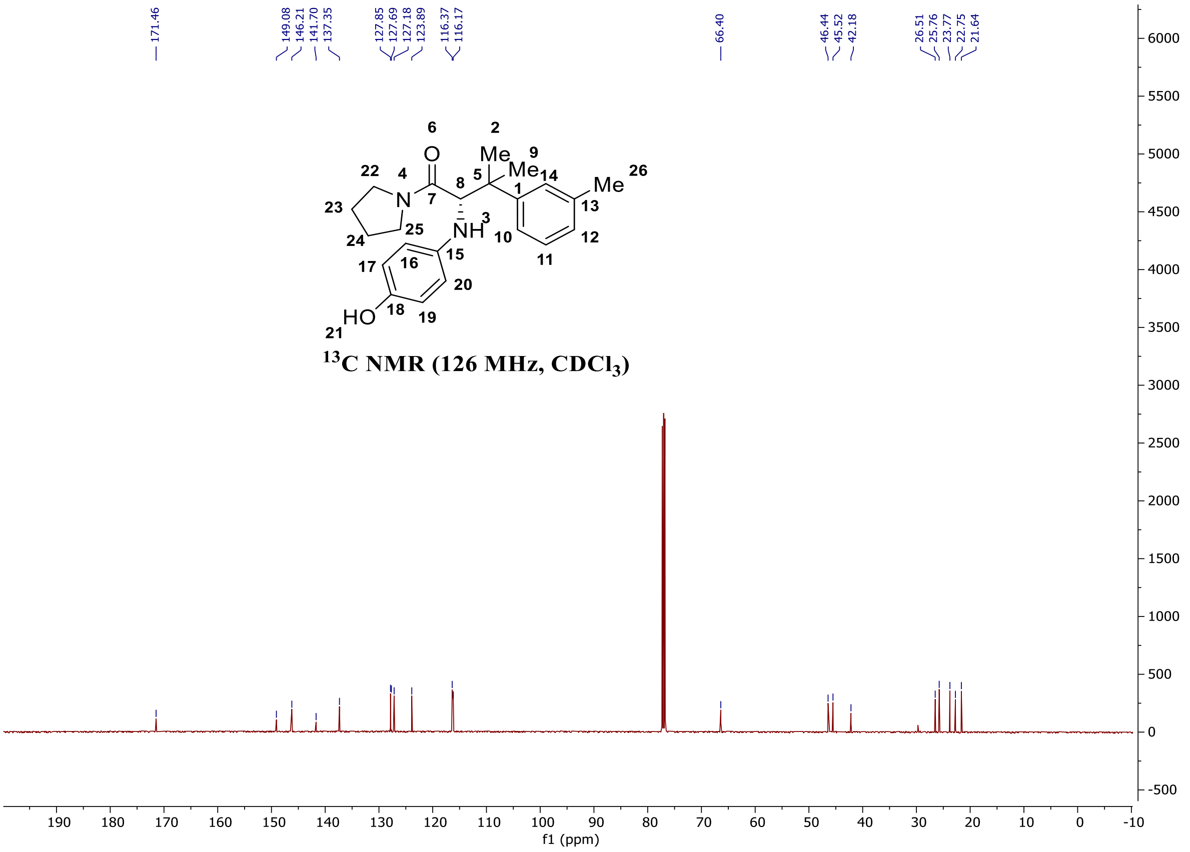

(*S*)-3-(3-Chlorophenyl)-2-((4-hydroxyphenyl)amino)-3-methyl-1-(pyrrolidin-1-yl)butan-1-one (3ik)

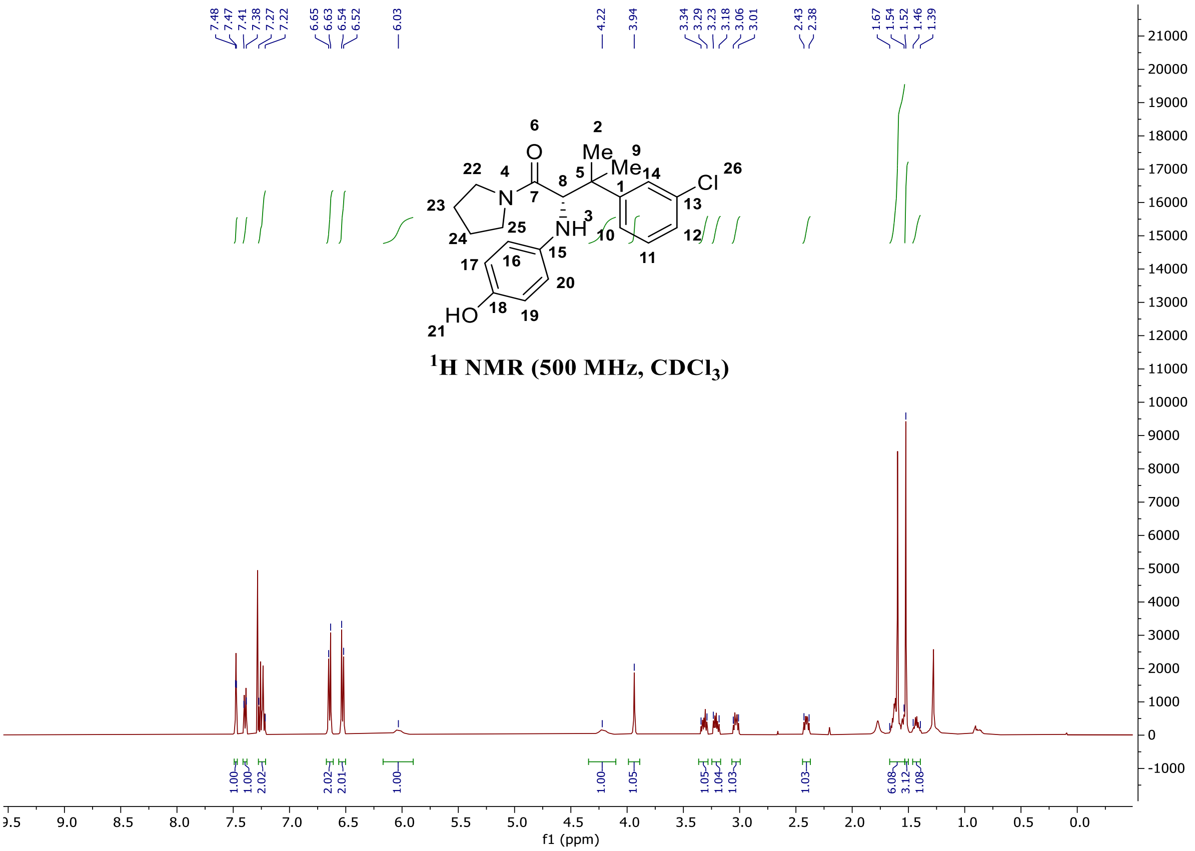

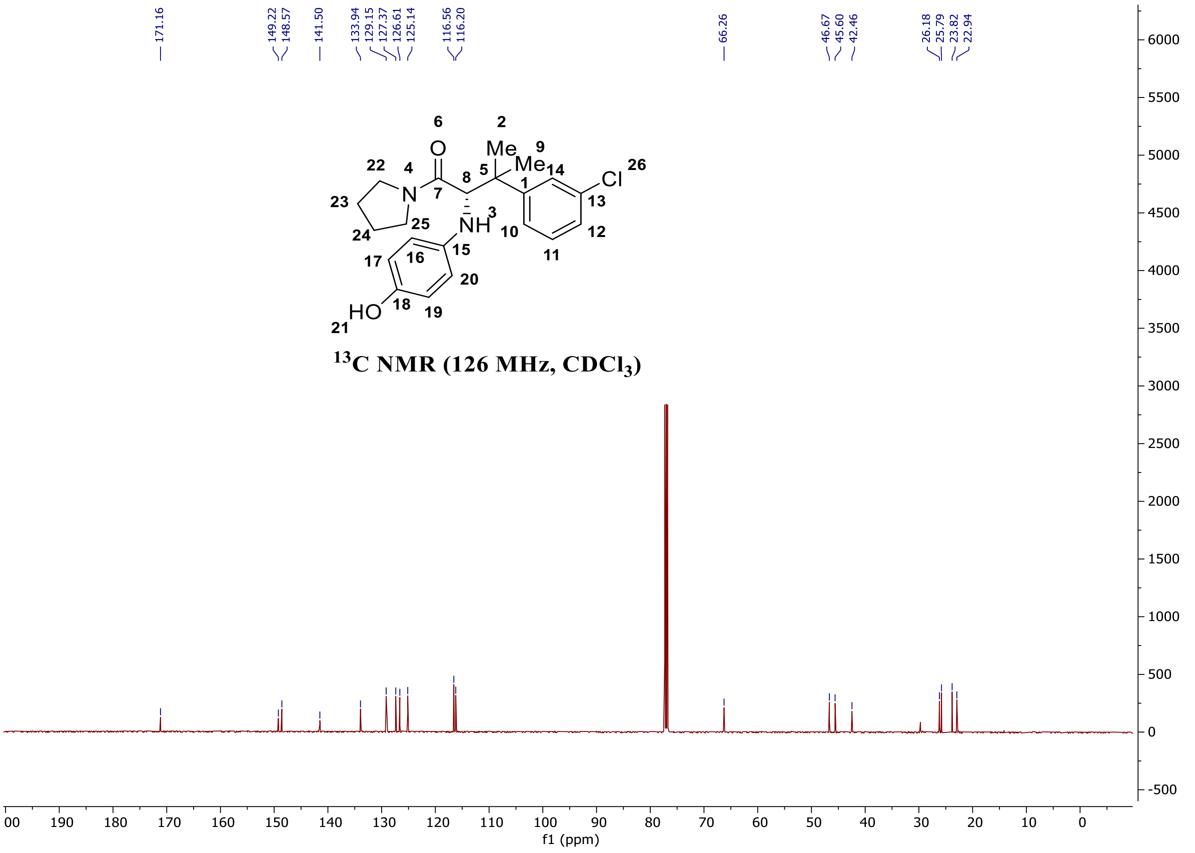

(*S*)-3-(2-Fluorophenyl)-2-((4-hydroxyphenyl)amino)-3-methyl-1-(pyrrolidin-1-yl)butan-1-one (3il)

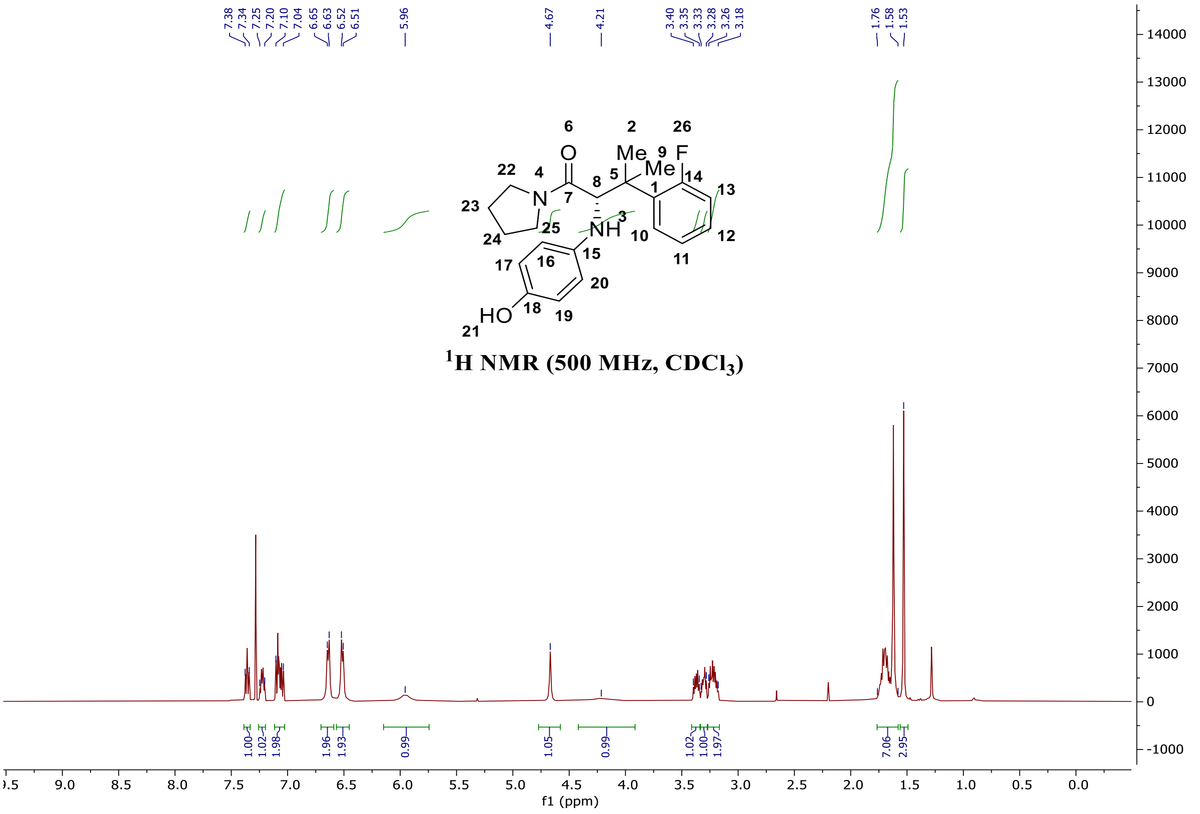

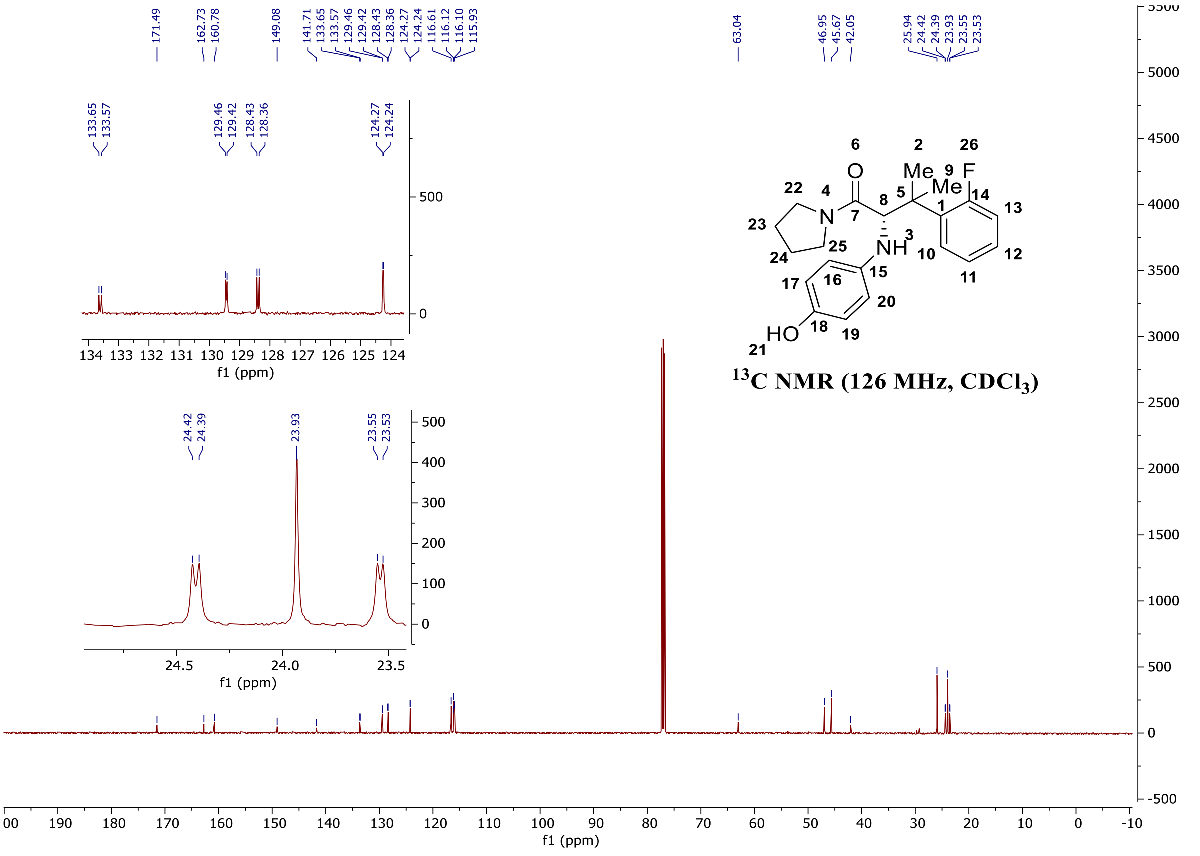

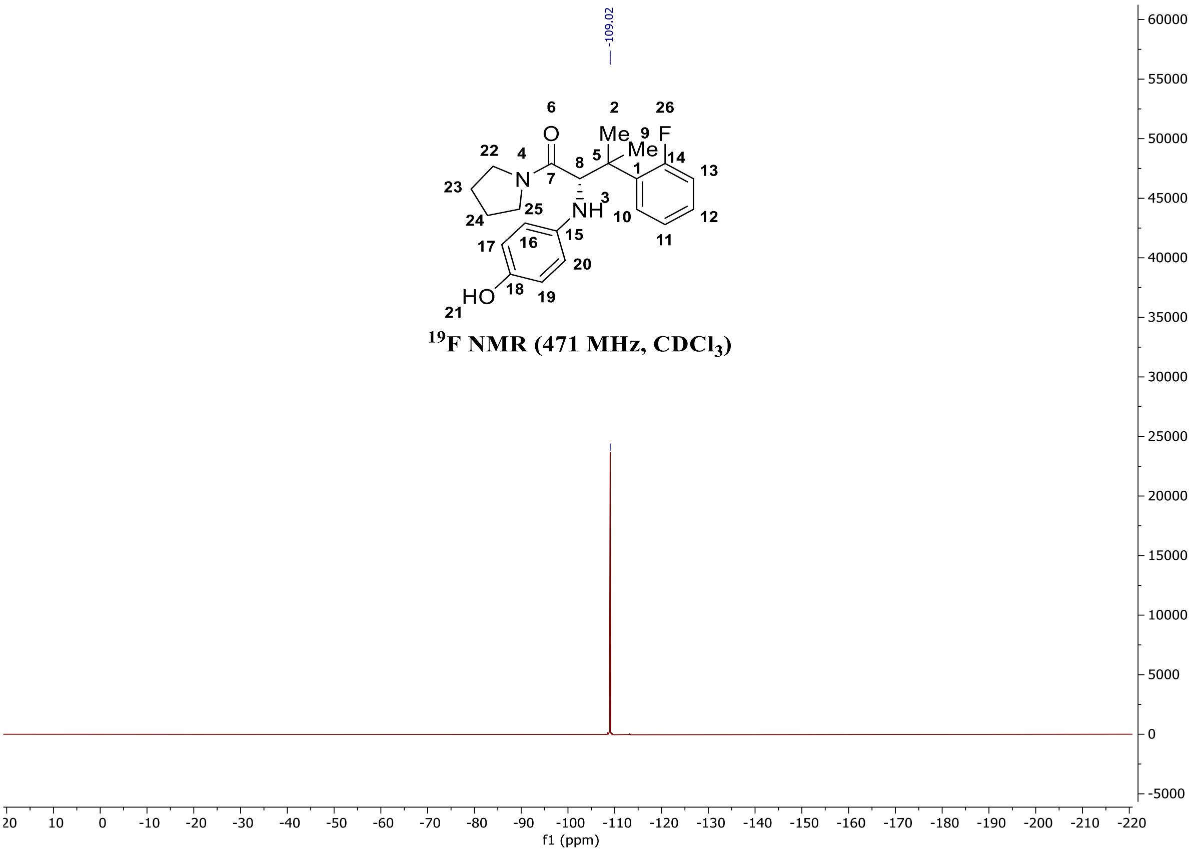

(*S*)-2-((4-Hydroxyphenyl)amino)-3-methyl-3-(naphthalen-2-yl)-1-(pyrrolidin-1-yl)butan-1-one (3im)

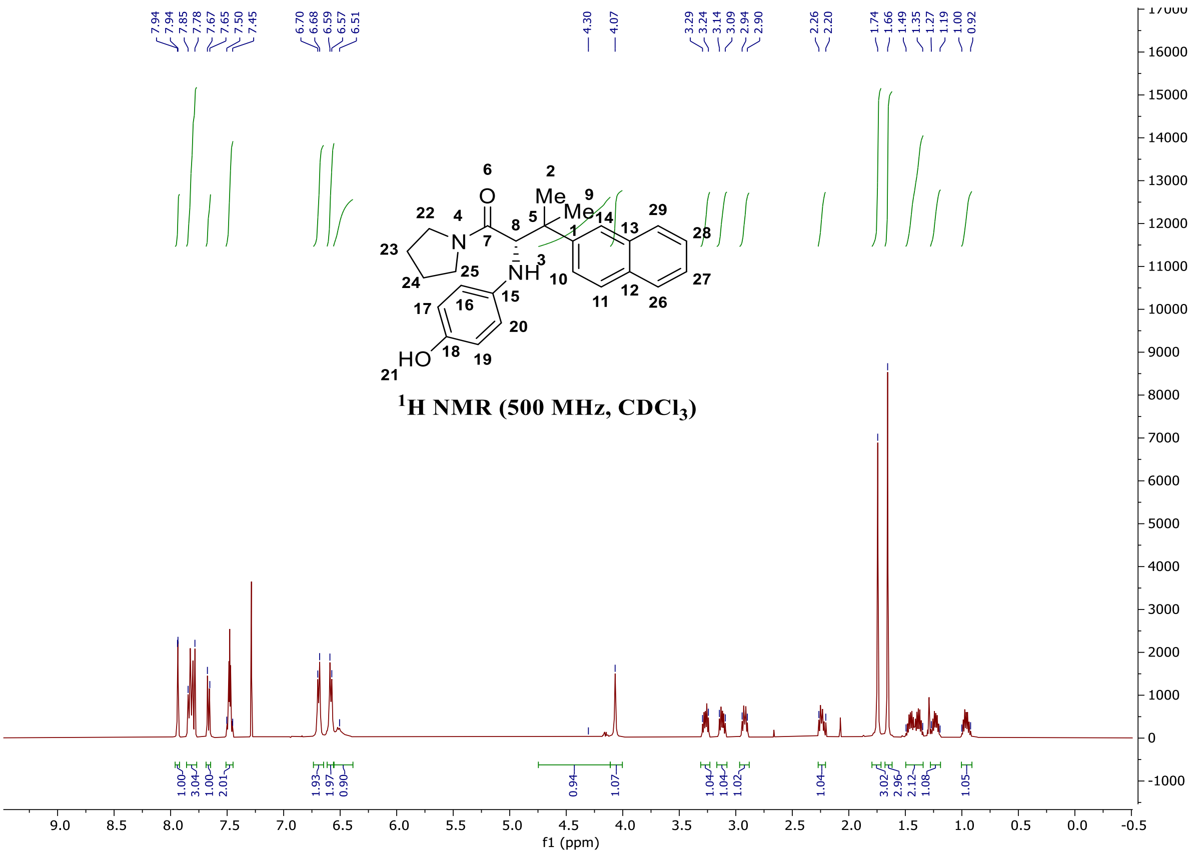

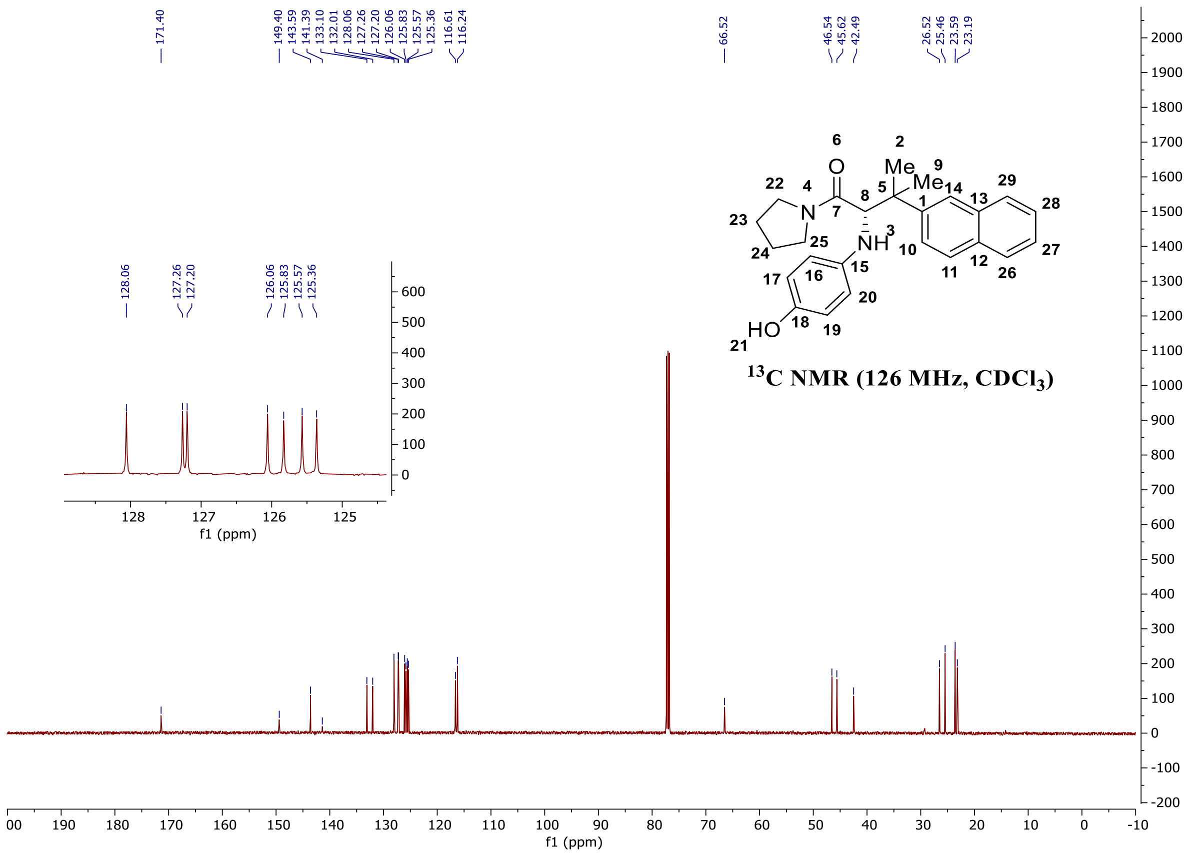

(*S*)-3-(Benzofuran-5-yl)-2-((4-hydroxyphenyl)amino)-3-methyl-1-(pyrrolidin-1-yl)butan-1-one (3in)

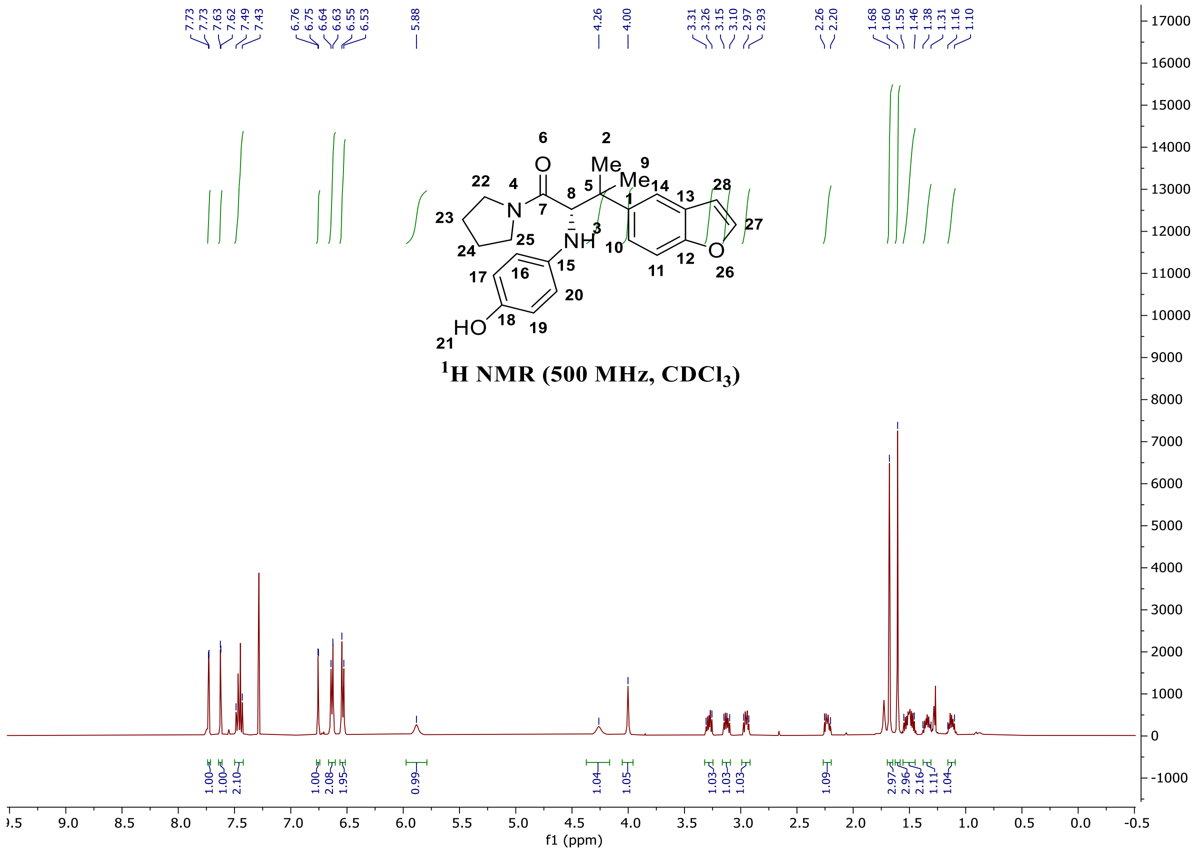

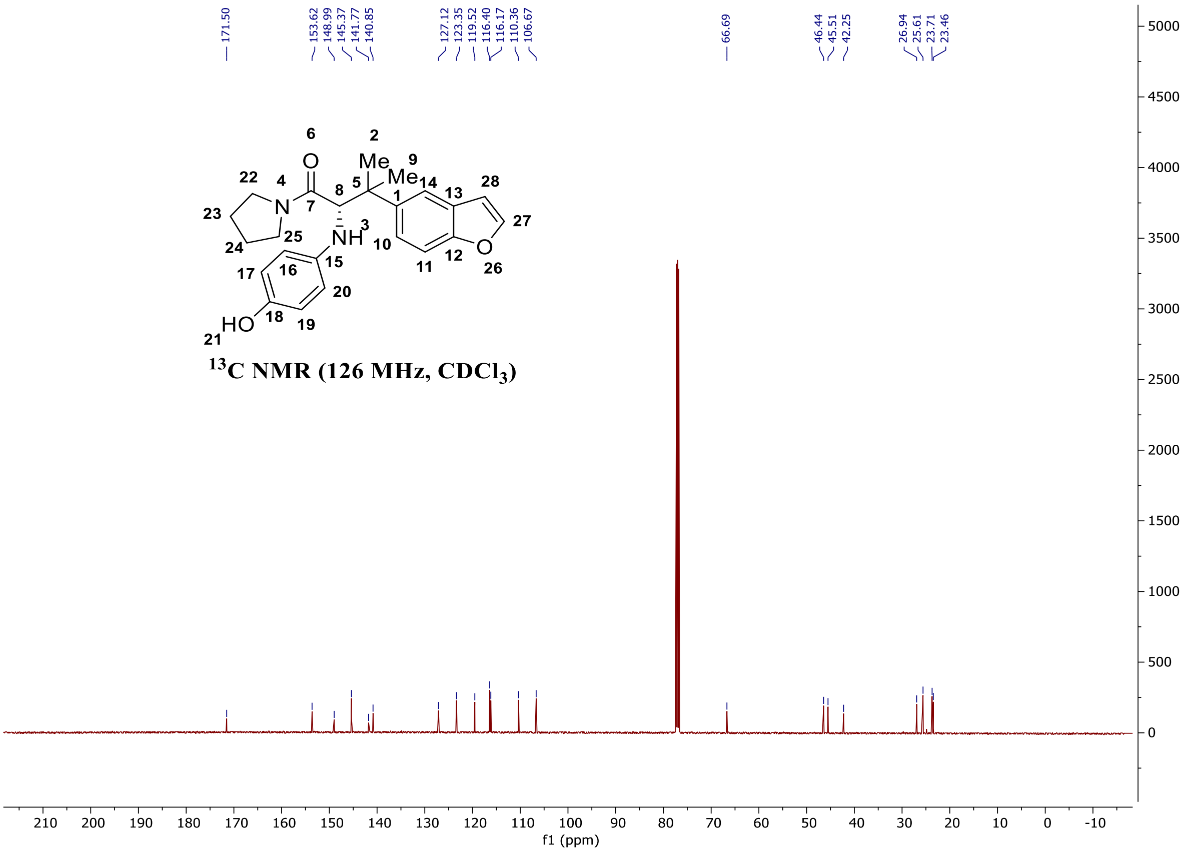

(*S*)-2-((4-Hydroxyphenyl)amino)-3-methyl-1-(pyrrolidin-1-yl)-3-(1-tosyl-1*H*-indol-3-yl)butan-1-one (3io)

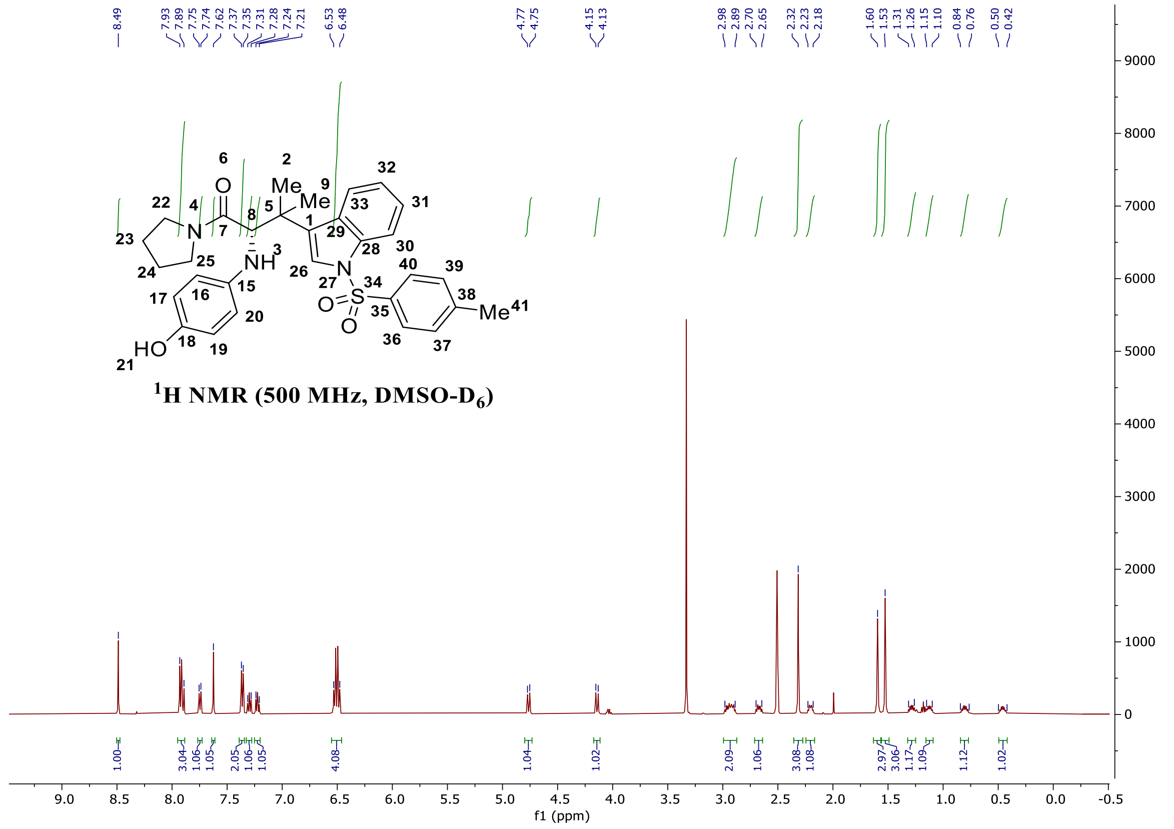

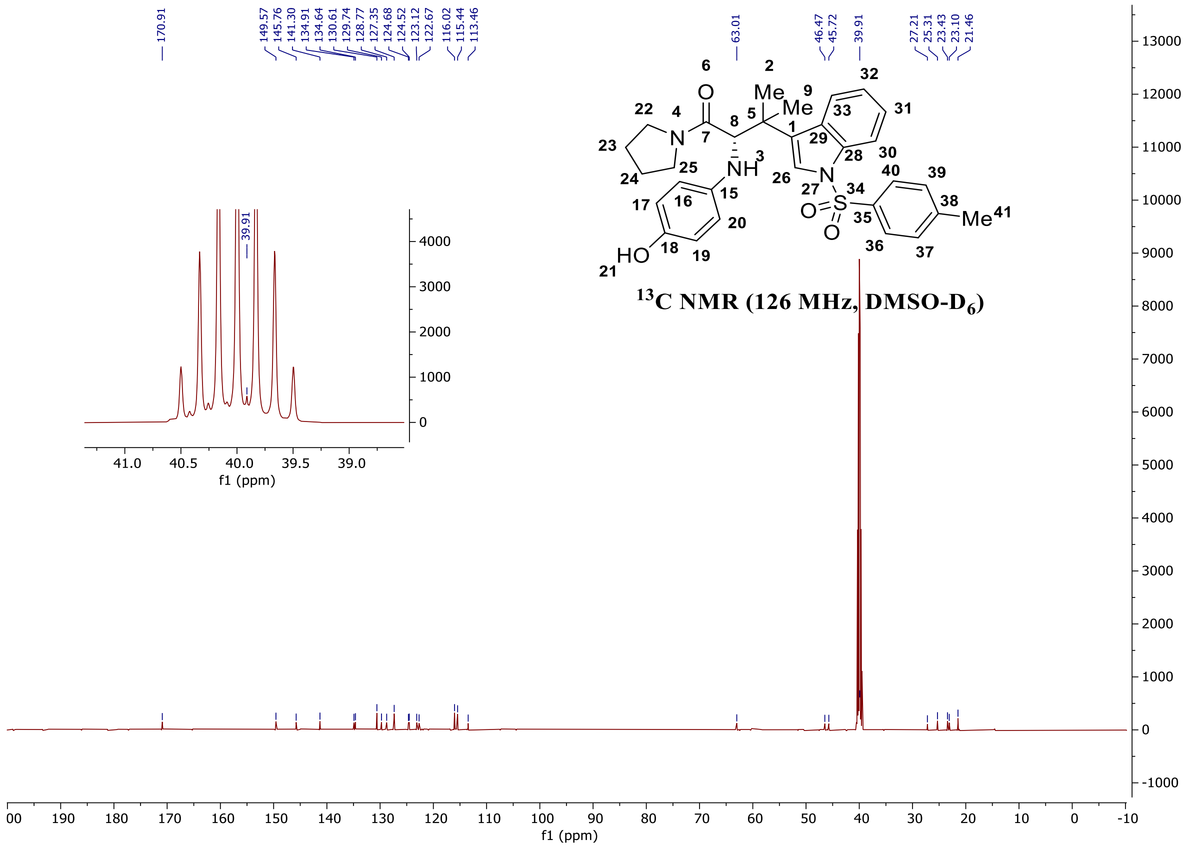

(*S*)-3-(Benzofuran-2-yl)-2-((4-hydroxyphenyl)amino)-3-methyl-1-(pyrrolidin-1-yl)butan-1-one (3ip)

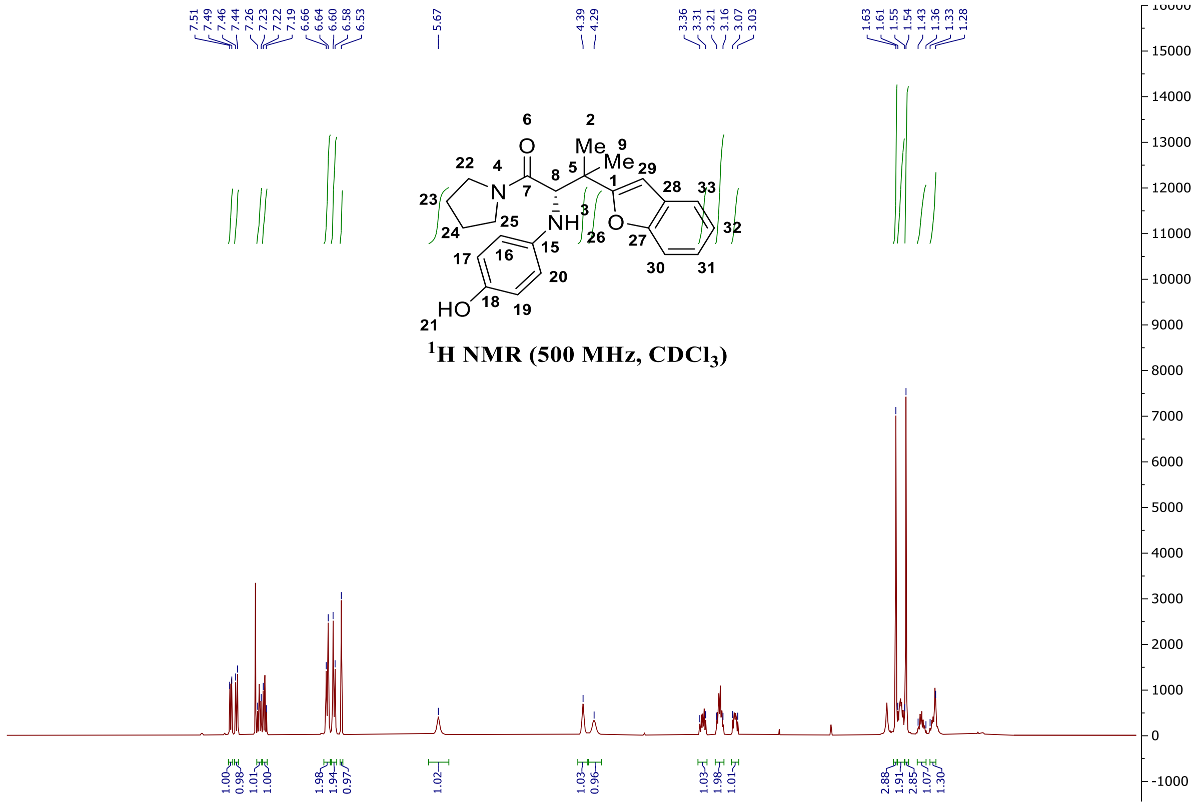

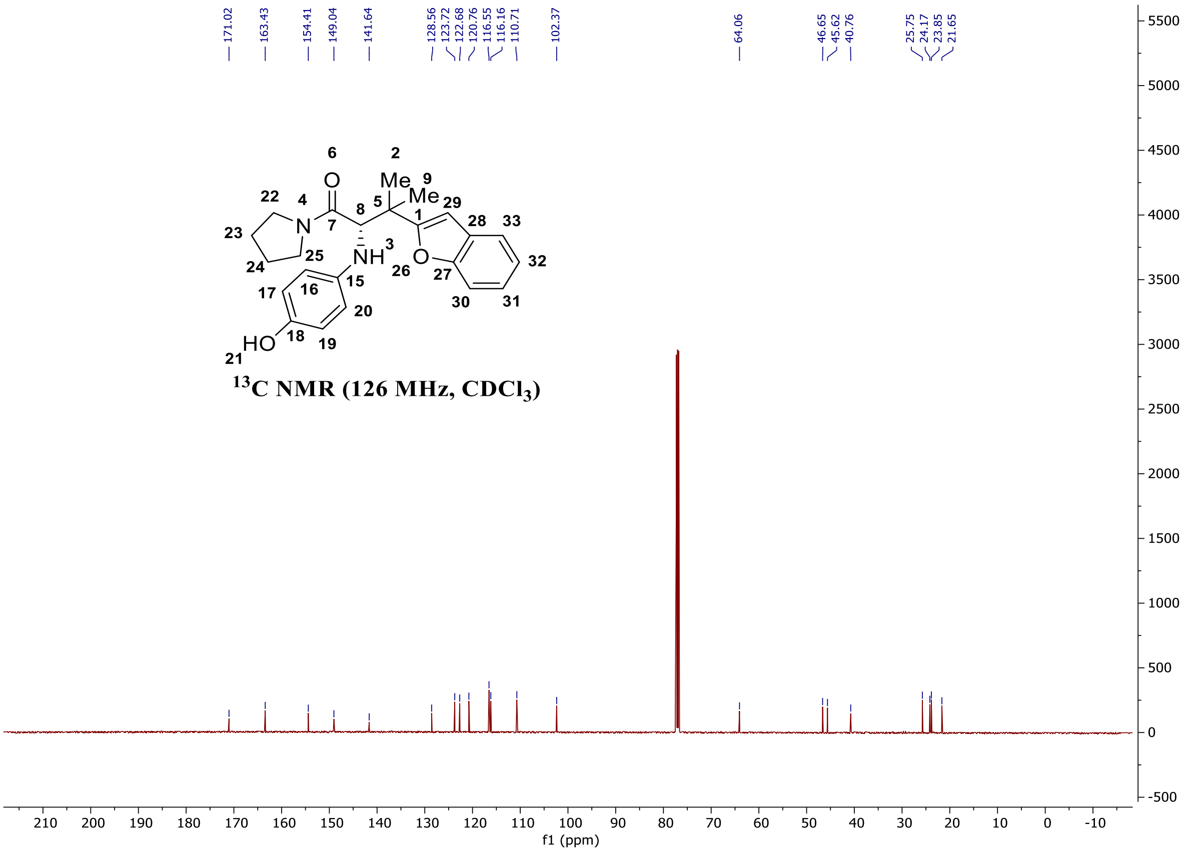

(*S*)-2-((4-Hydroxyphenyl)amino)-3-methyl-1-(pyrrolidin-1-yl)-3-(thiophen-3-yl)butan-1-one (3iq)

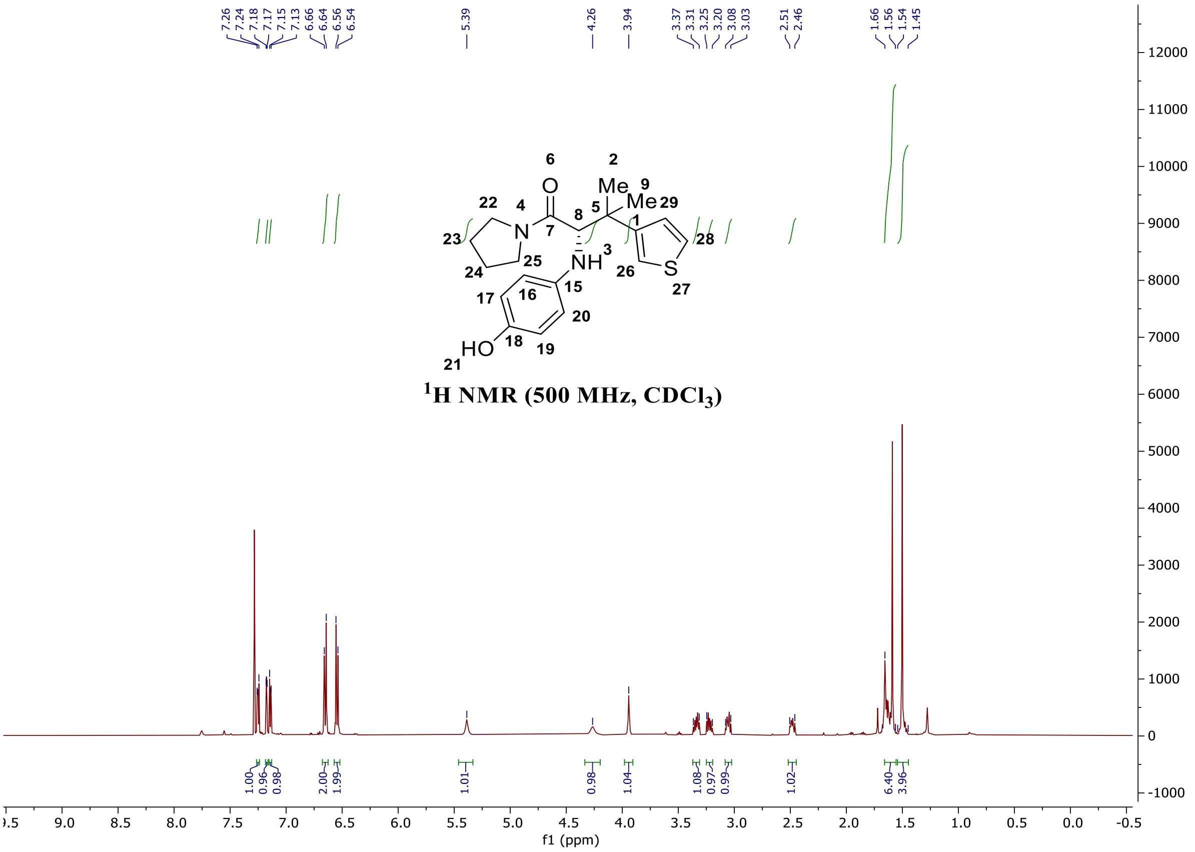

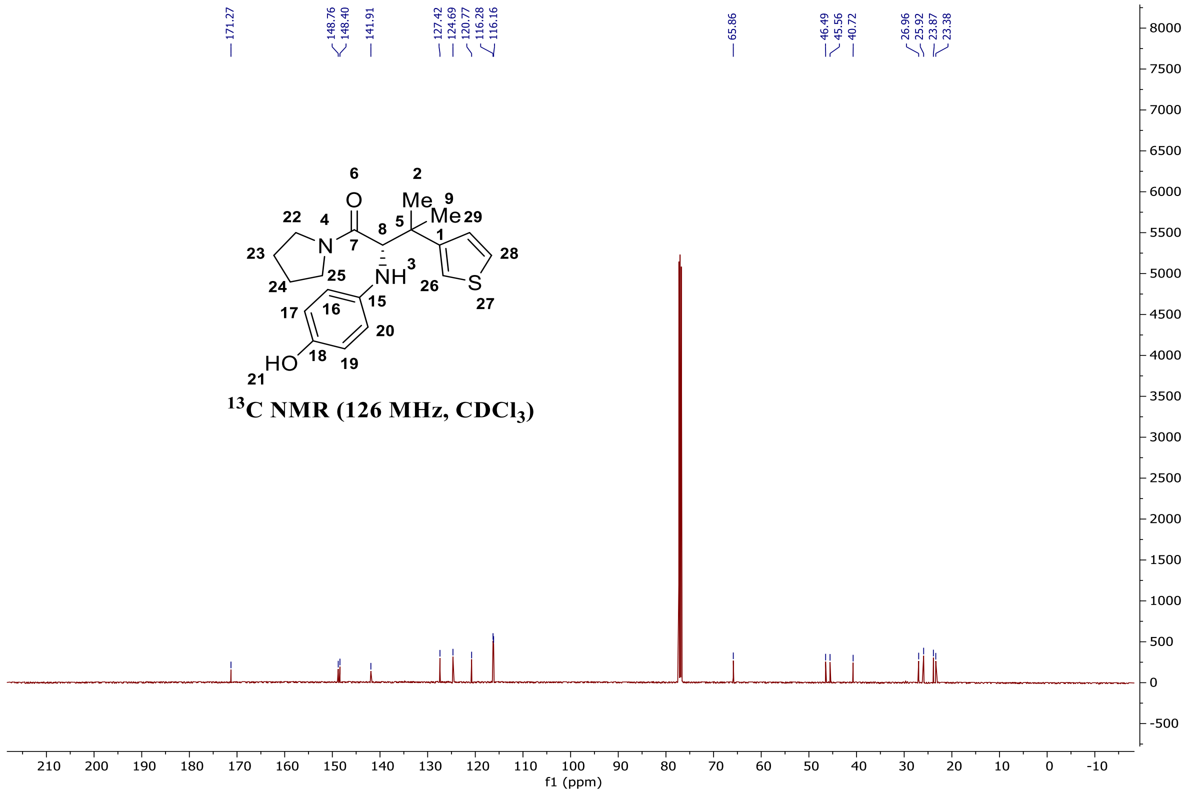

(8*R*,9*S*,13*R*,14*S*)-3-((*S*)-3-((4-Hydroxyphenyl)amino)-2-methyl-4-oxo-4-(pyrrolidin-1-yl)butan-2-yl)-13-methyl-6,7,8,9,11,12,13,14,15,16-decahydro-17*H*-cyclopenta[*a*]phenanthren-17-one (3ir)

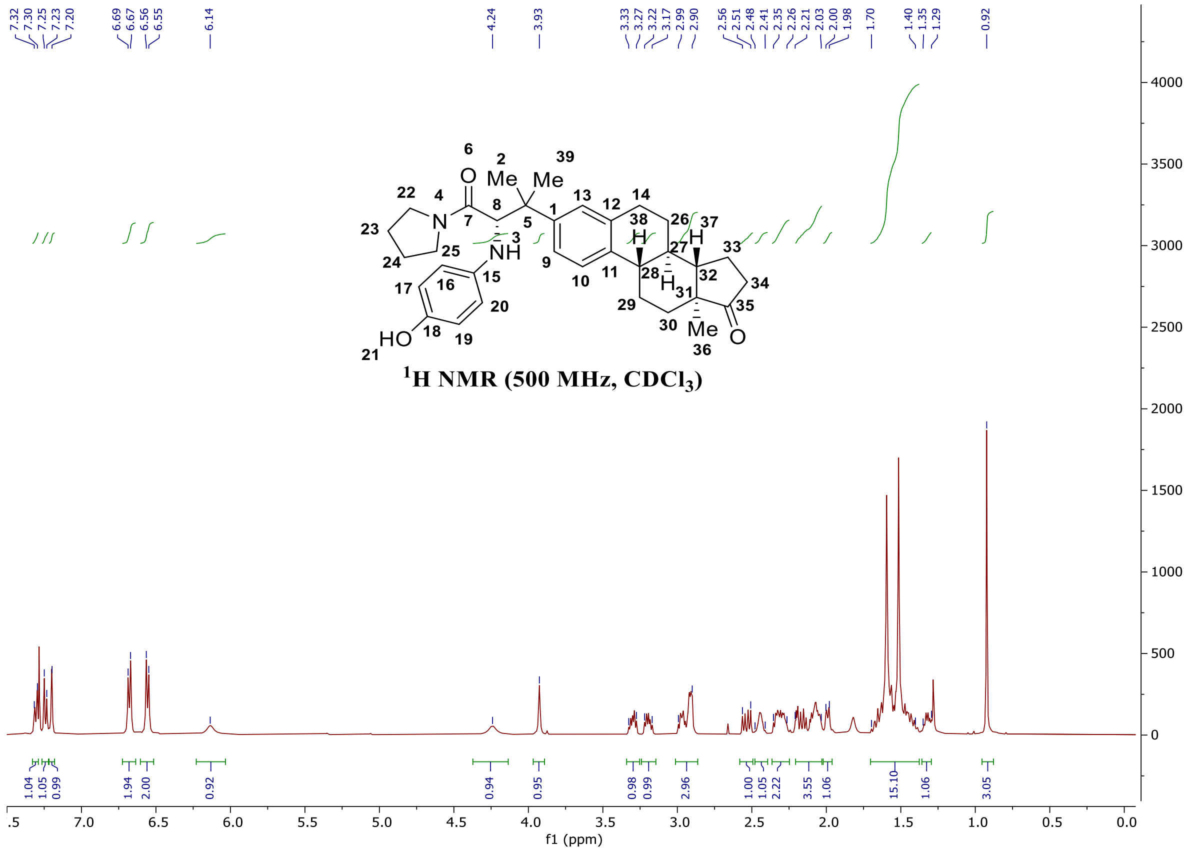

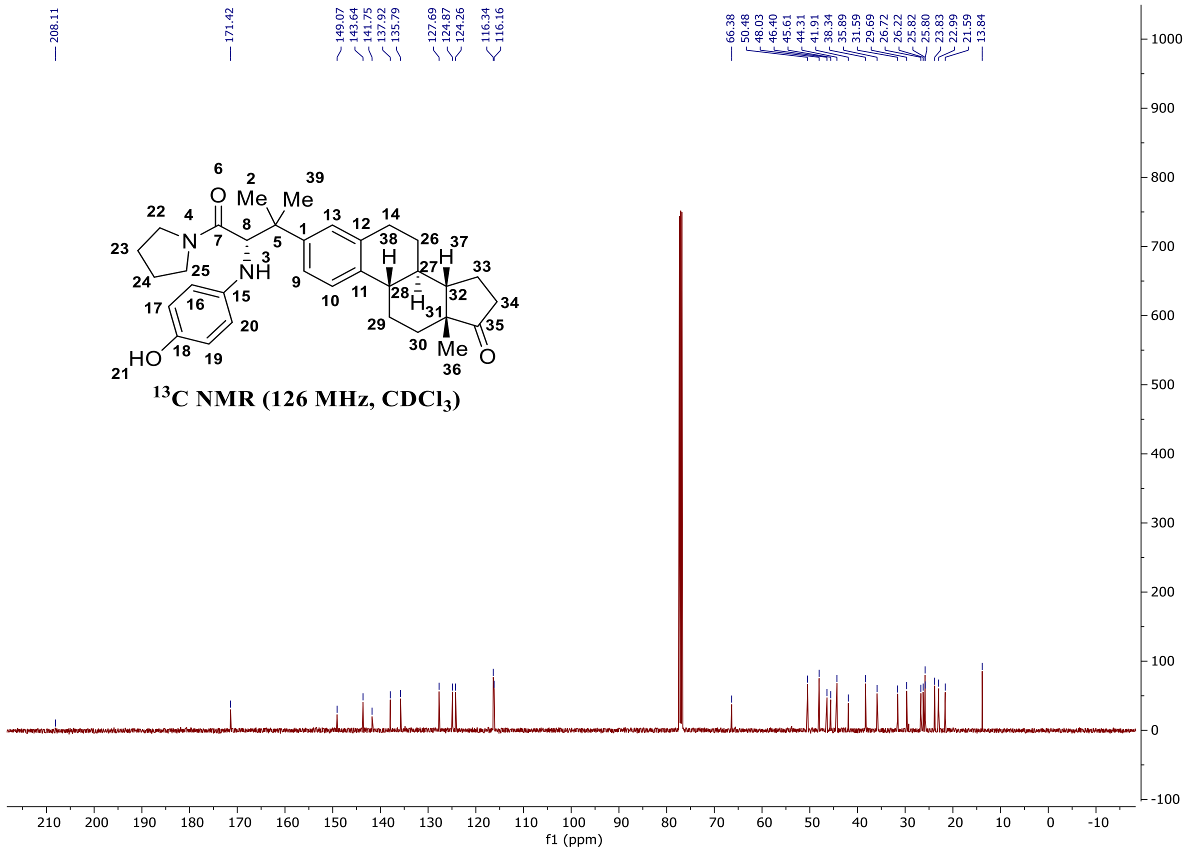

(*2S,3R*)-3-Cyclobutyl-2-((4-hydroxyphenyl)amino)-3-phenyl-1-(pyrrolidin-1-yl)butan-1-one (3is)

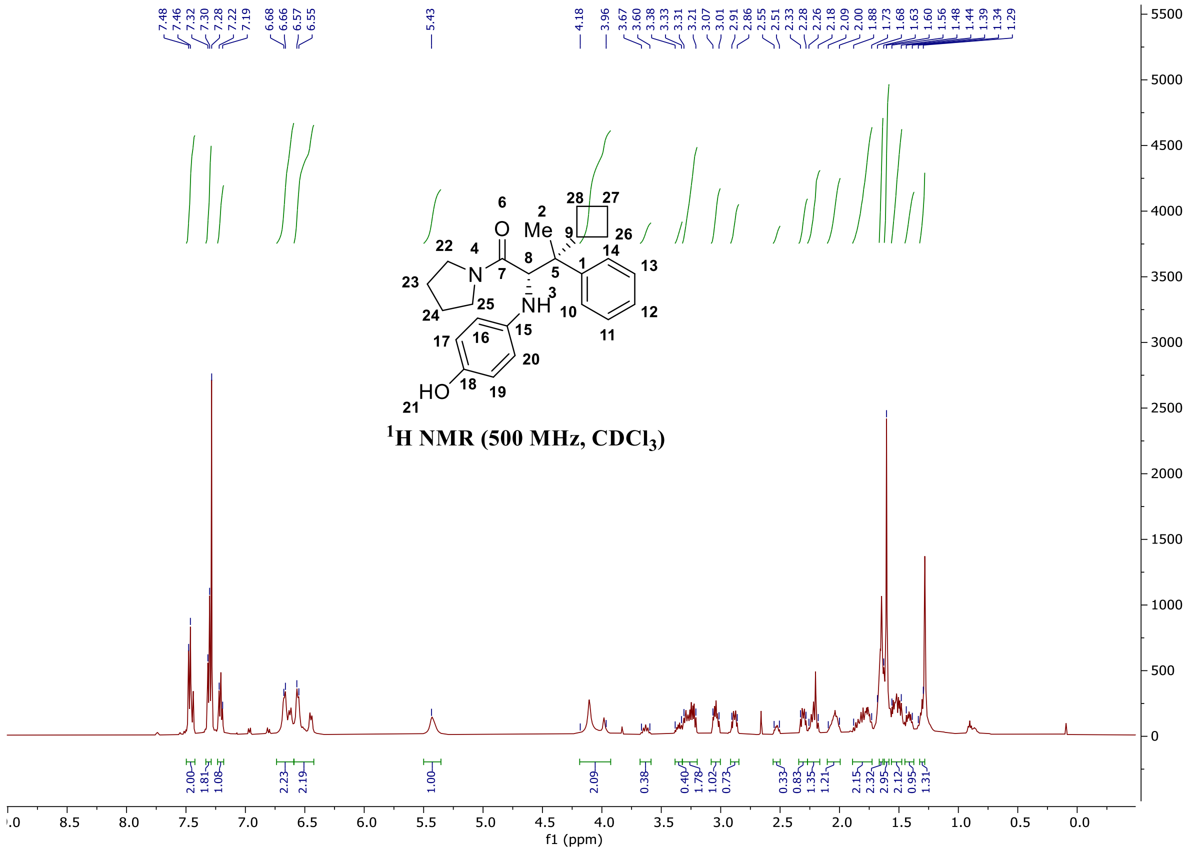

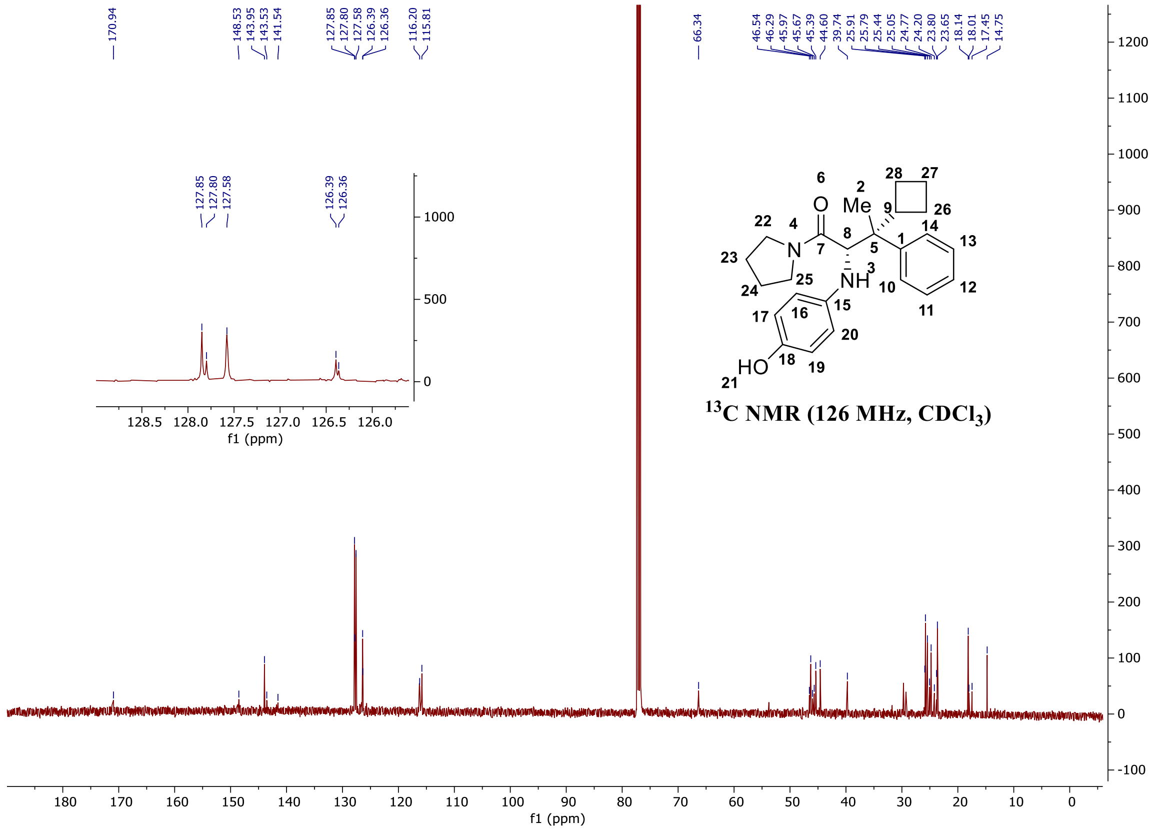

(2*S*,3*R*)-4,4,4-Trifluoro-2-((4-hydroxyphenyl)amino)-3-methyl-3-phenyl-1-(pyrrolidin-1-yl)butan-1-one (3it)

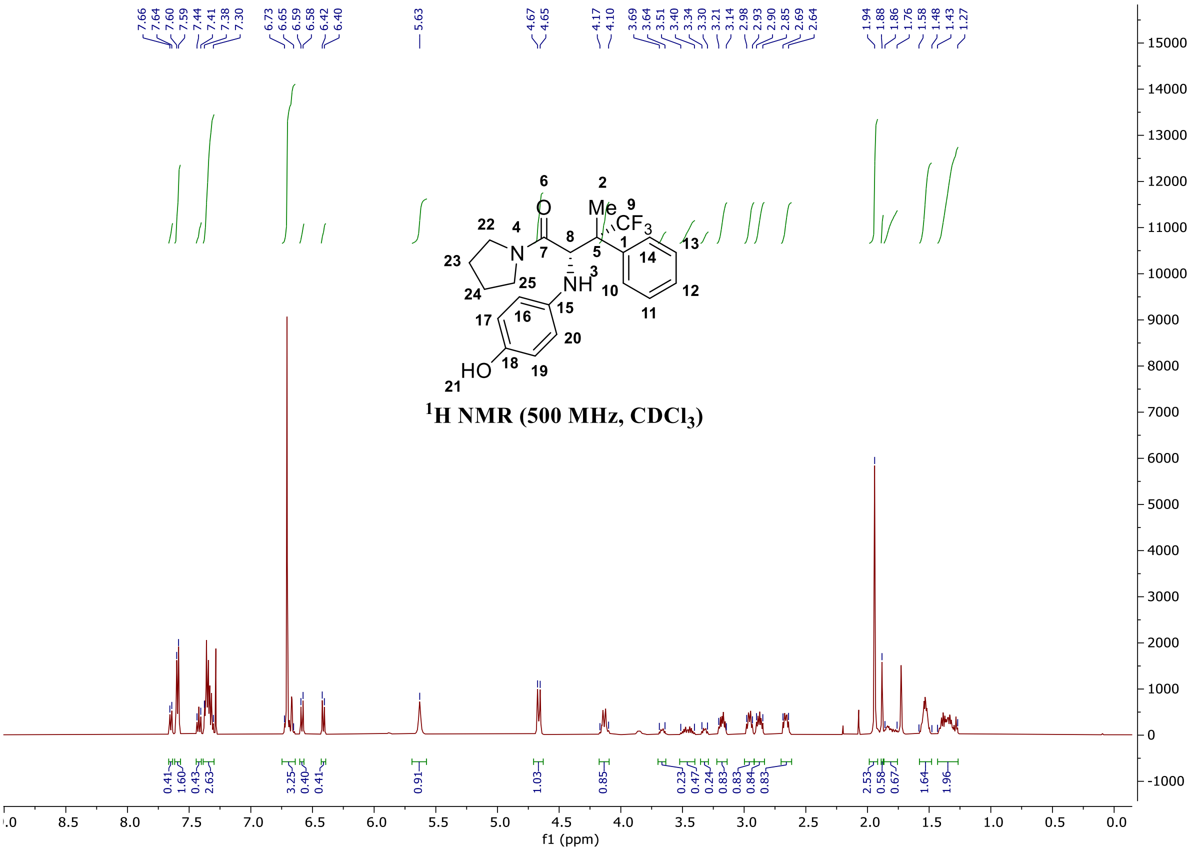

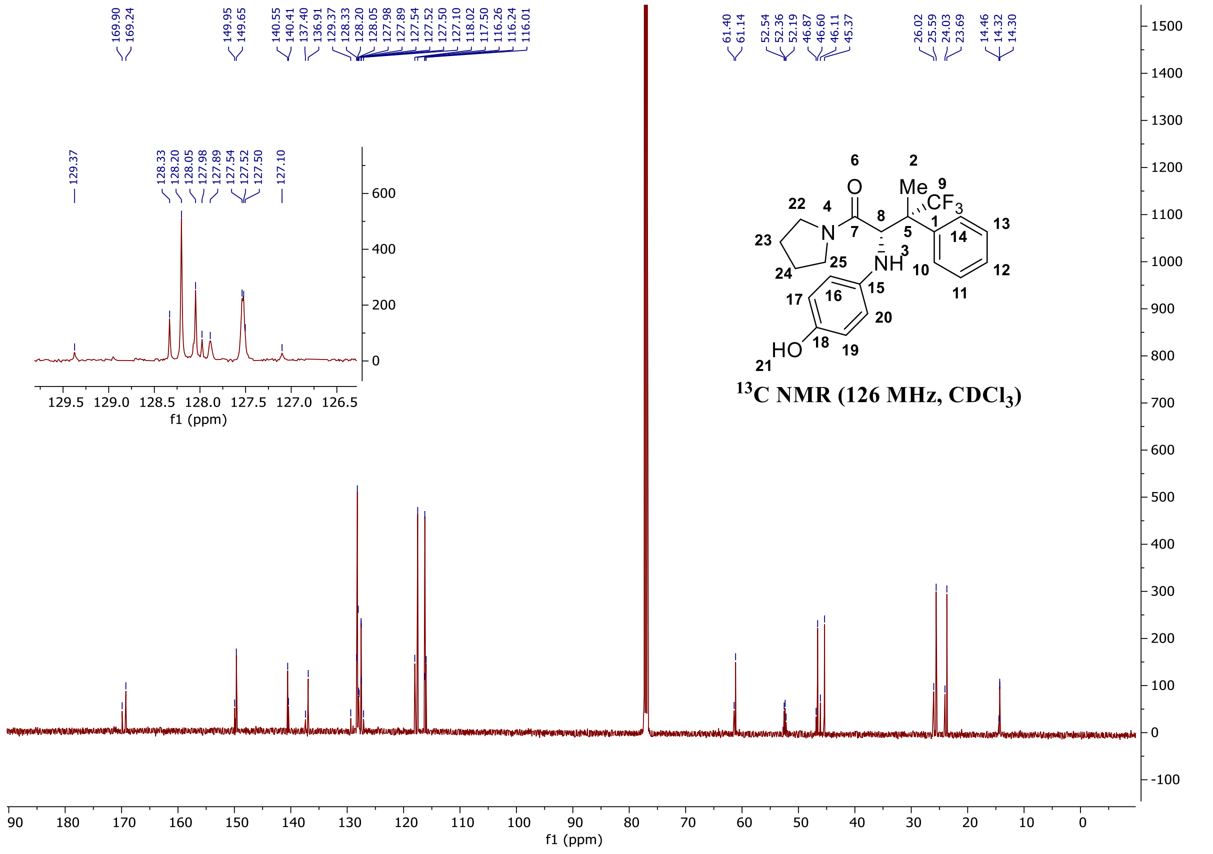
13C NMR (126 MHz, CDCl3)
f1 (ppm)

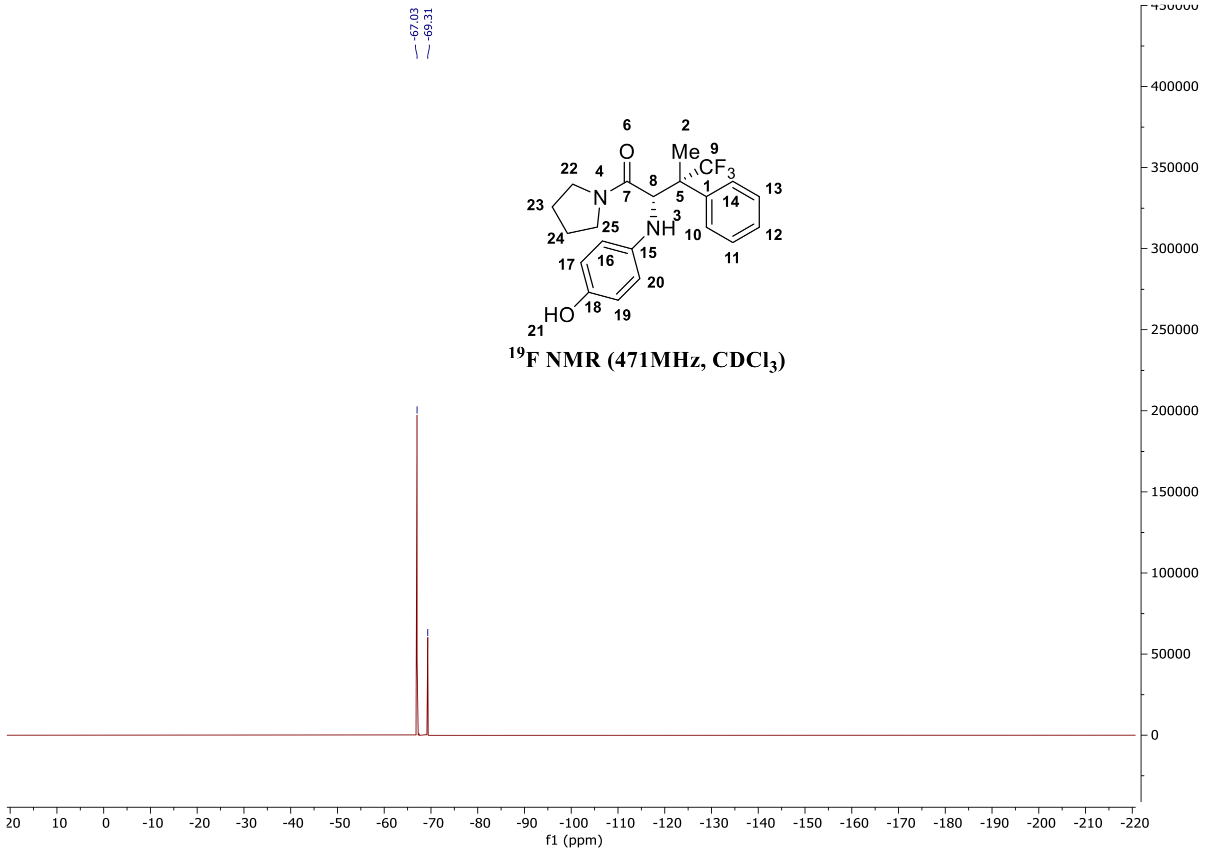
-67.03
-69.31
19F NMR (471MHz, CDCl3)
f1 (ppm)

(*S*)-2-((4-Hydroxyphenyl)amino)-2-((*R*)-7-methylbicyclo[4.2.0]octa-1(6),2,4-trien-7-yl)-1-(pyrrolidin-1-yl)ethan-1-one (3iu)

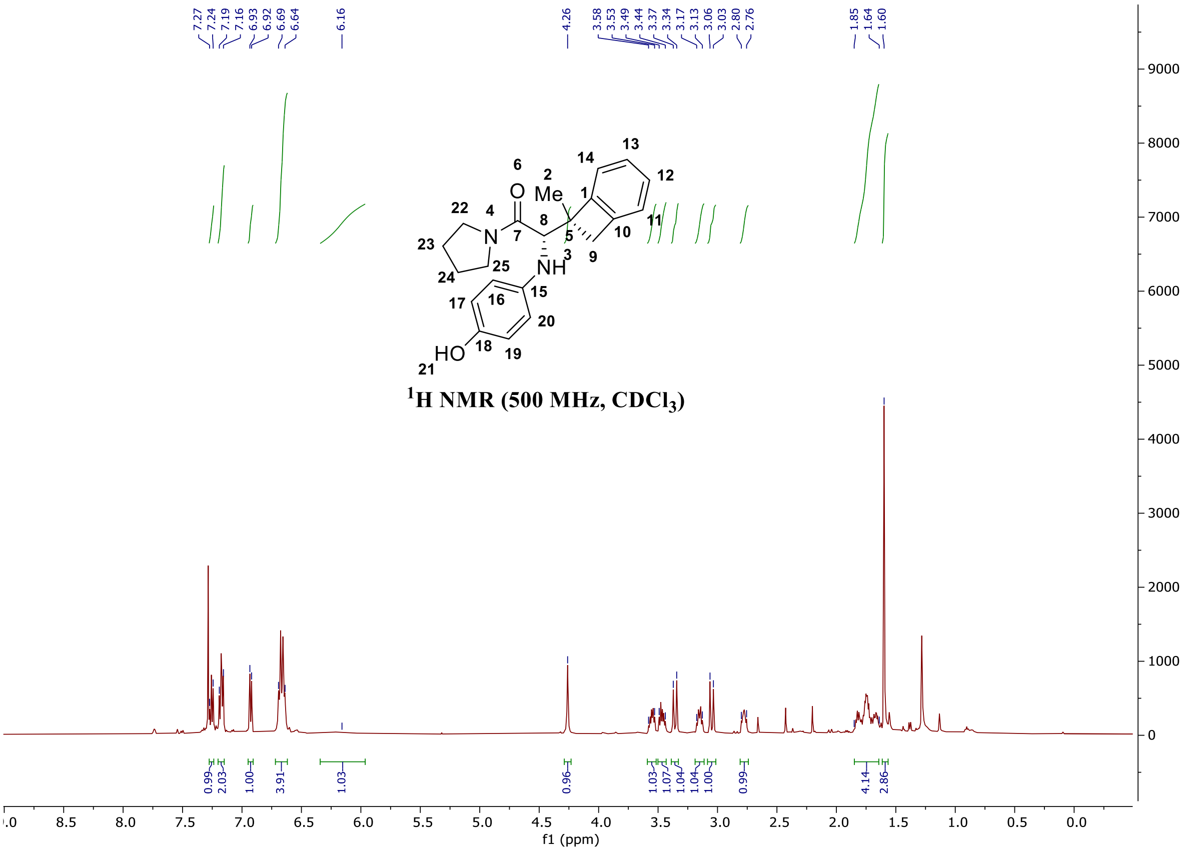

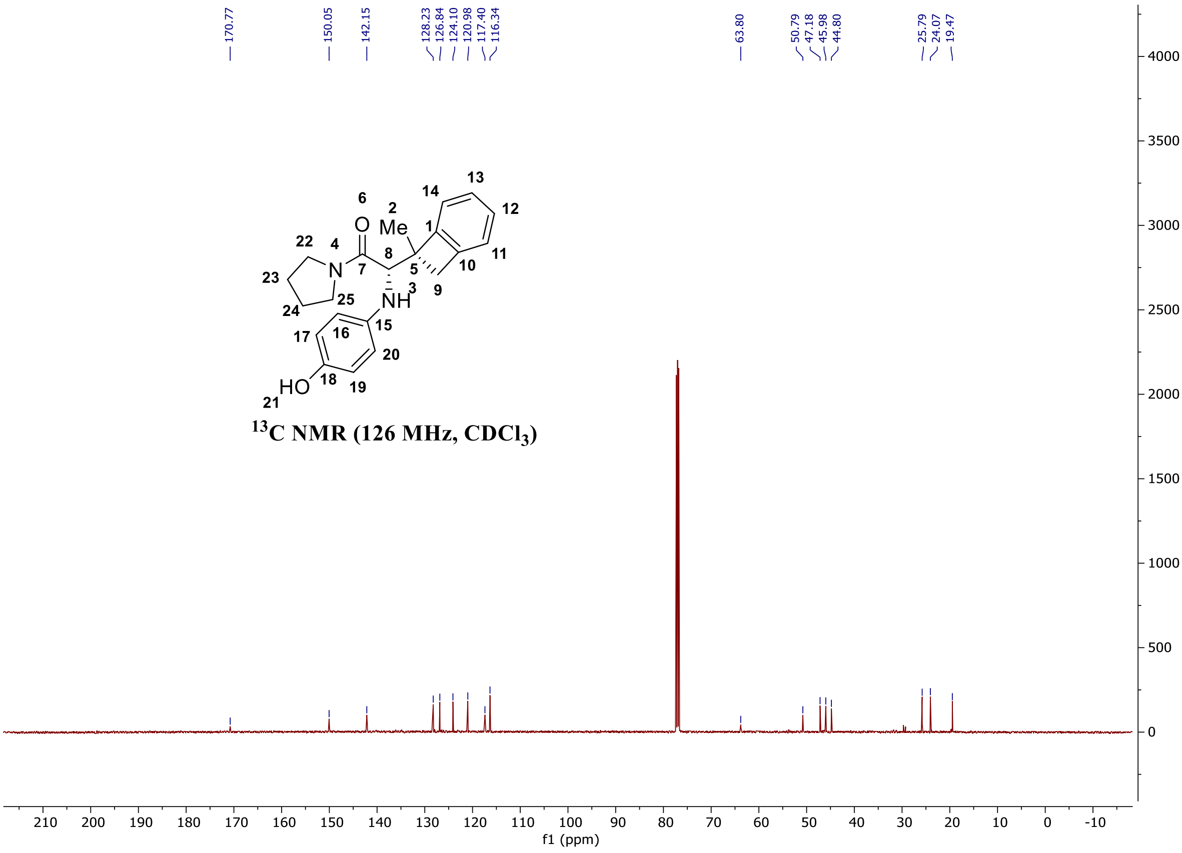

(2*S*,3*R*)-2-((4-Hydroxyphenyl)amino)-3-methyl-3,5-diphenyl-1-(pyrrolidin-1-yl)pentan-1-one (3iv)

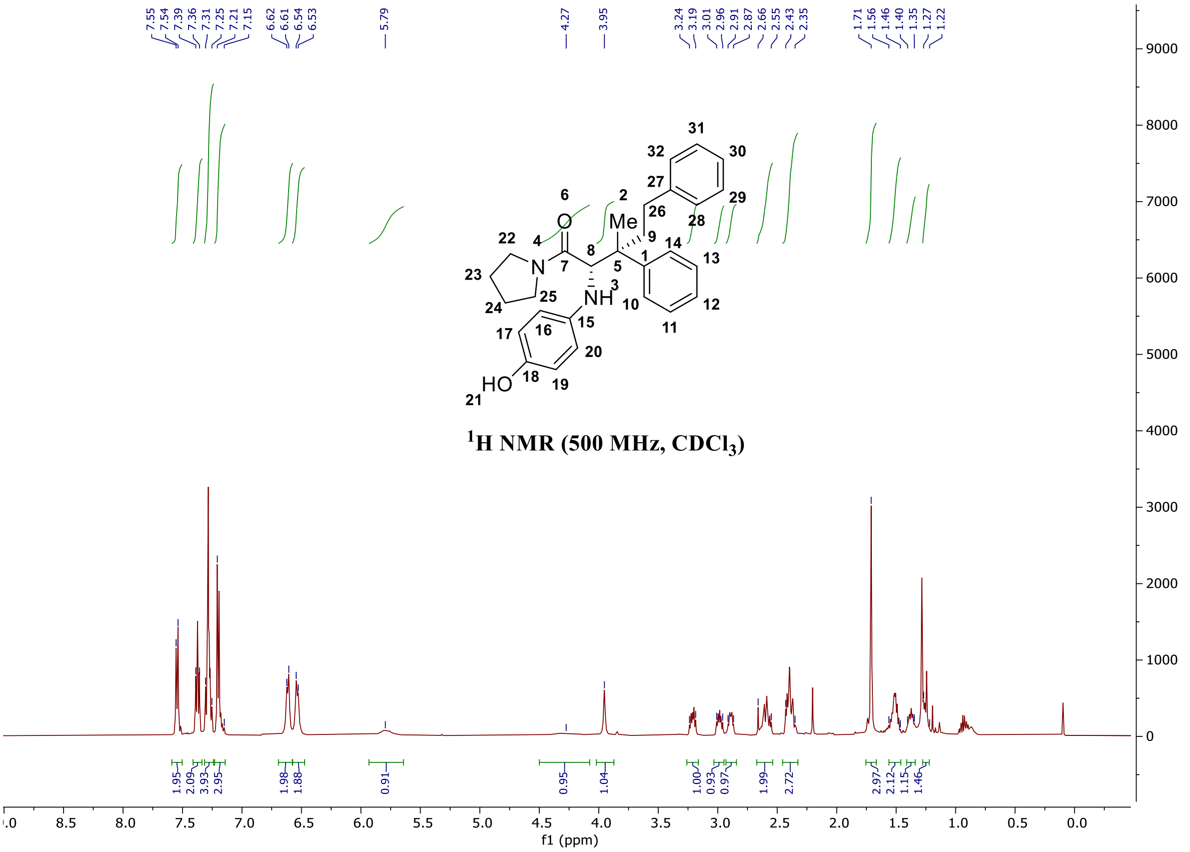

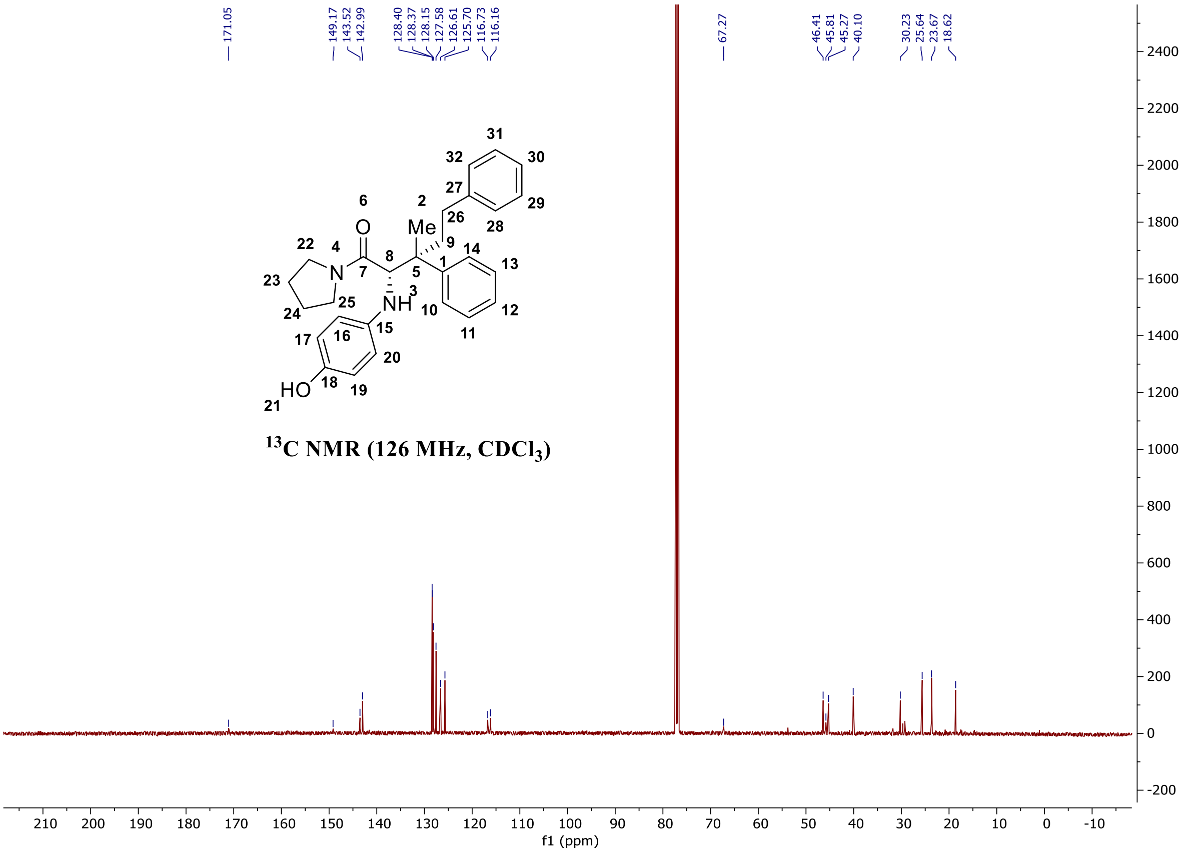

(*2S,3R*)-2-((4-Hydroxyphenyl)amino)-3-methyl-3-phenyl-1-(pyrrolidin-1-yl)pentan-1-one (3iw)

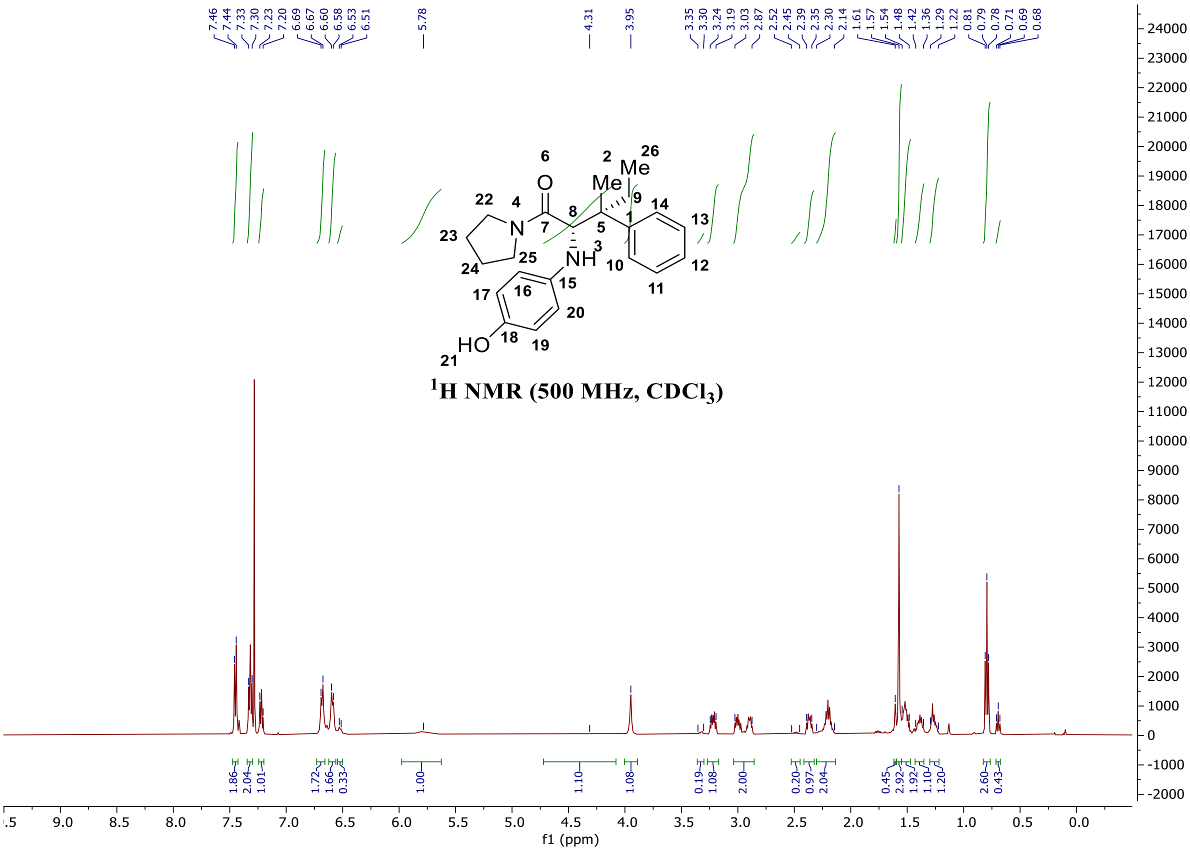

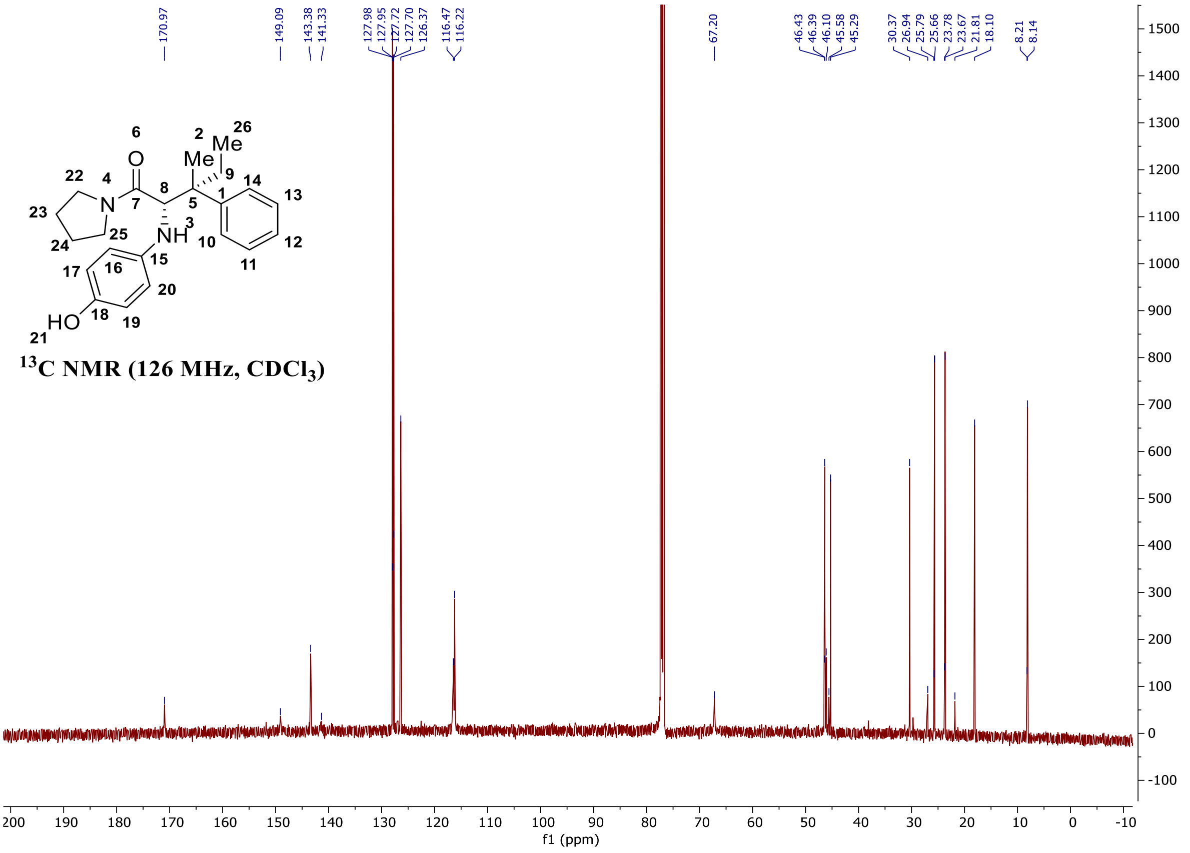

(*2S,3R*)-2-((4-Hydroxyphenyl)amino)-3-methyl-3-phenyl-1-(pyrrolidin-1-yl)nonan-1-one (3ix)

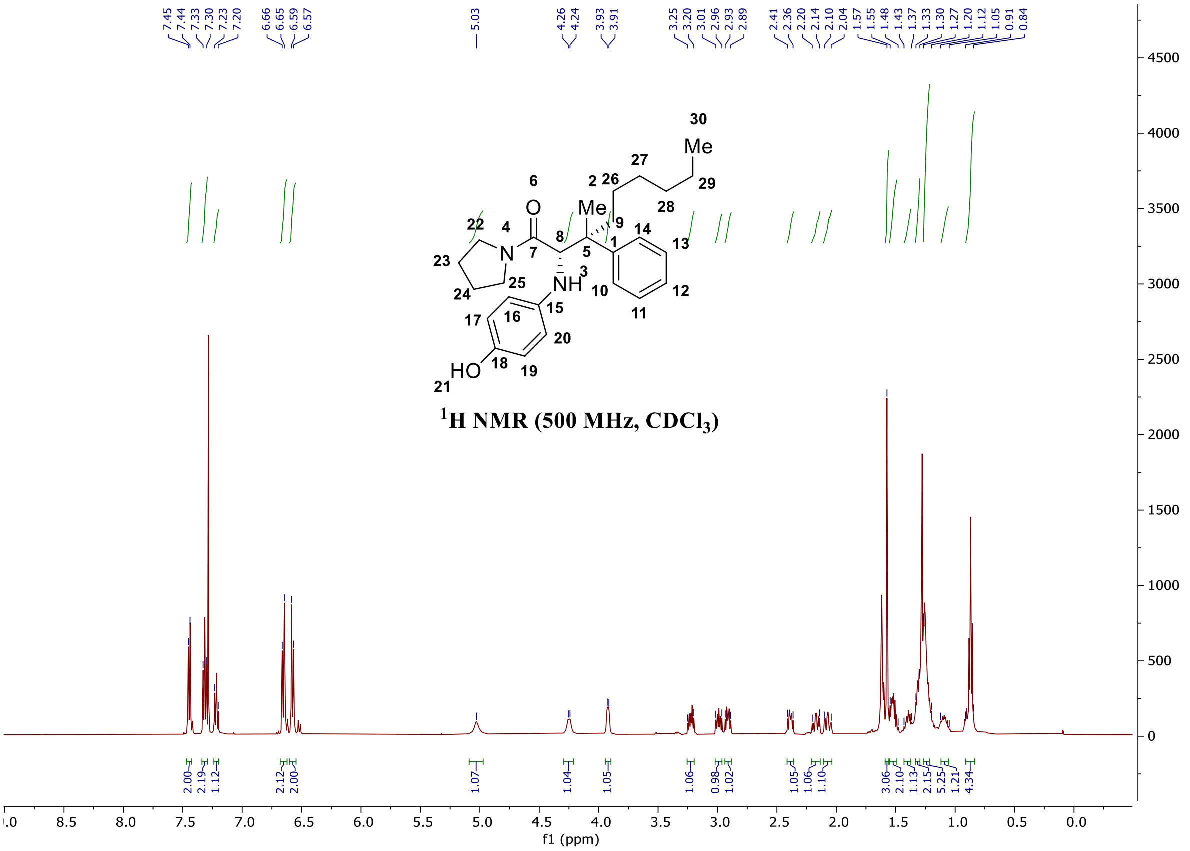

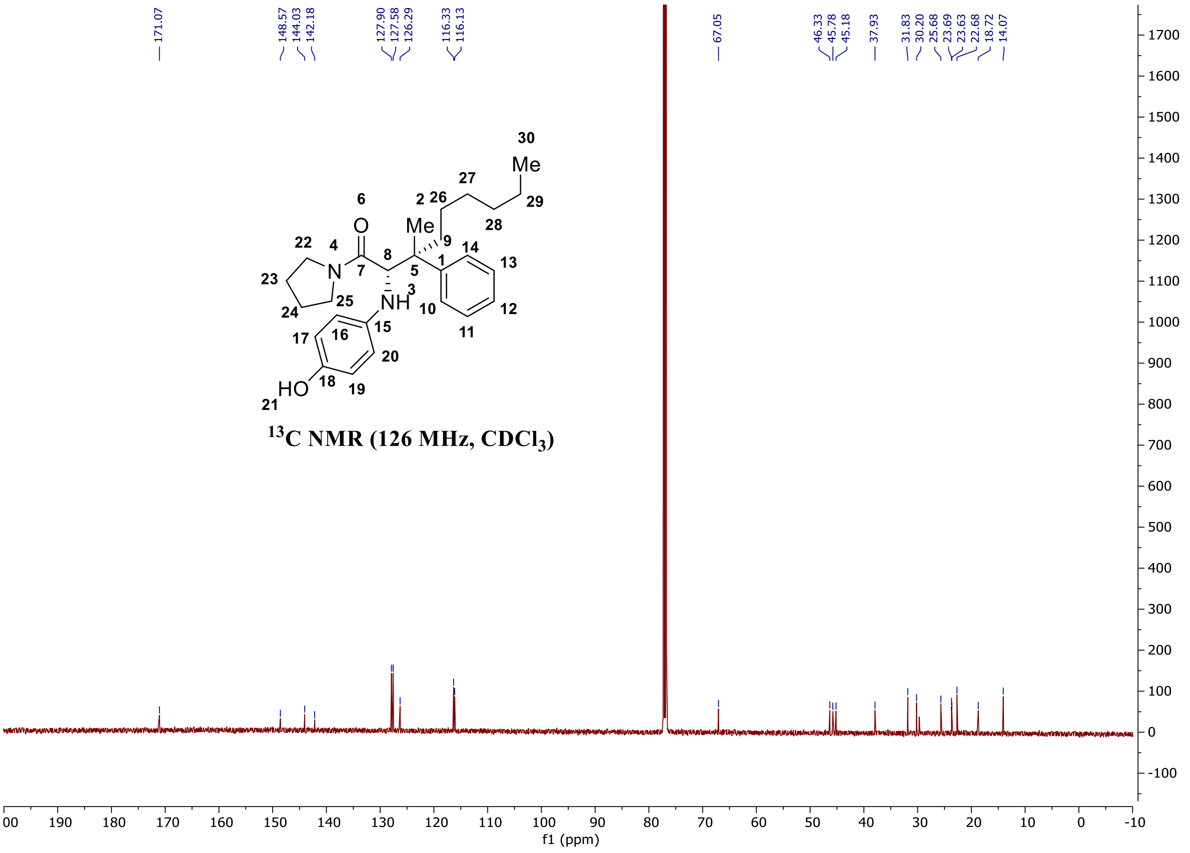

4-Bromo-*N*-((*2S*,*3R*)-3-methyl-1-oxo-3-phenyl-1-(pyrrolidin-1-yl)pentan-2-yl)benzamide (5)

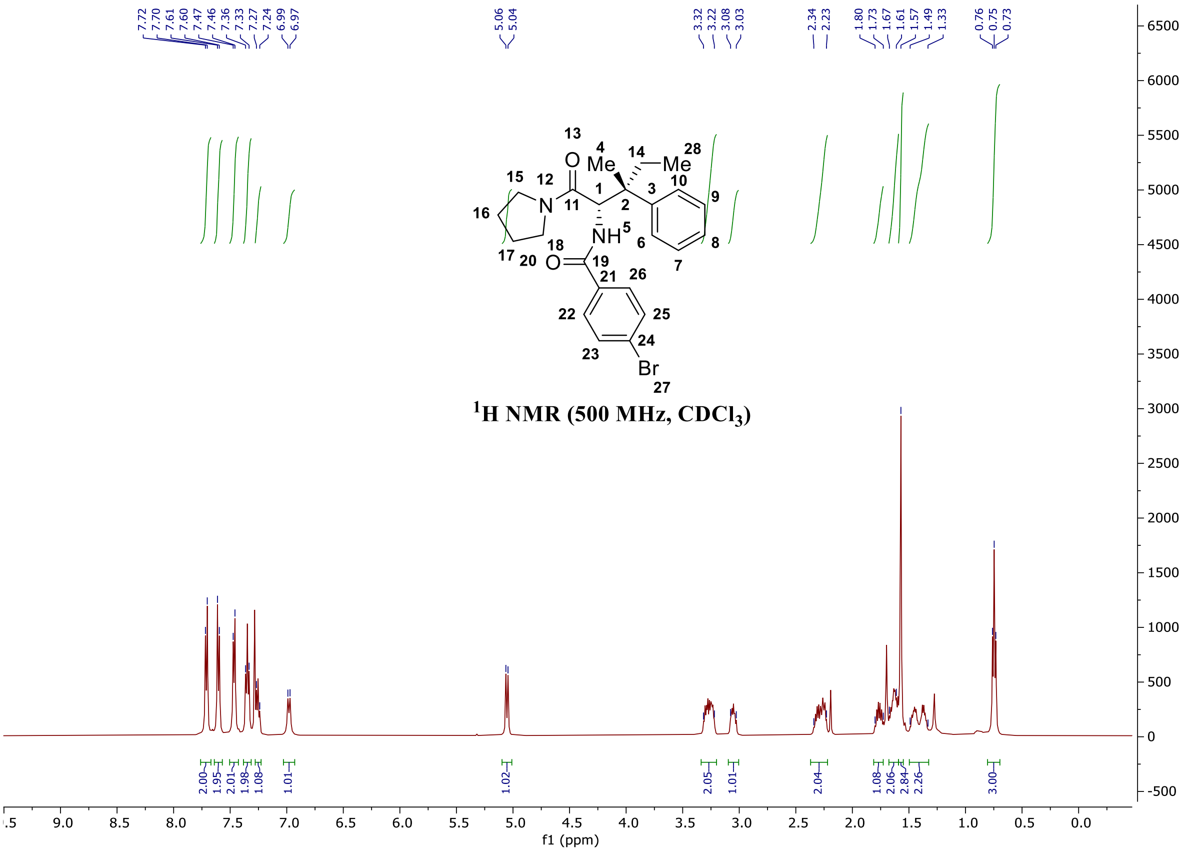

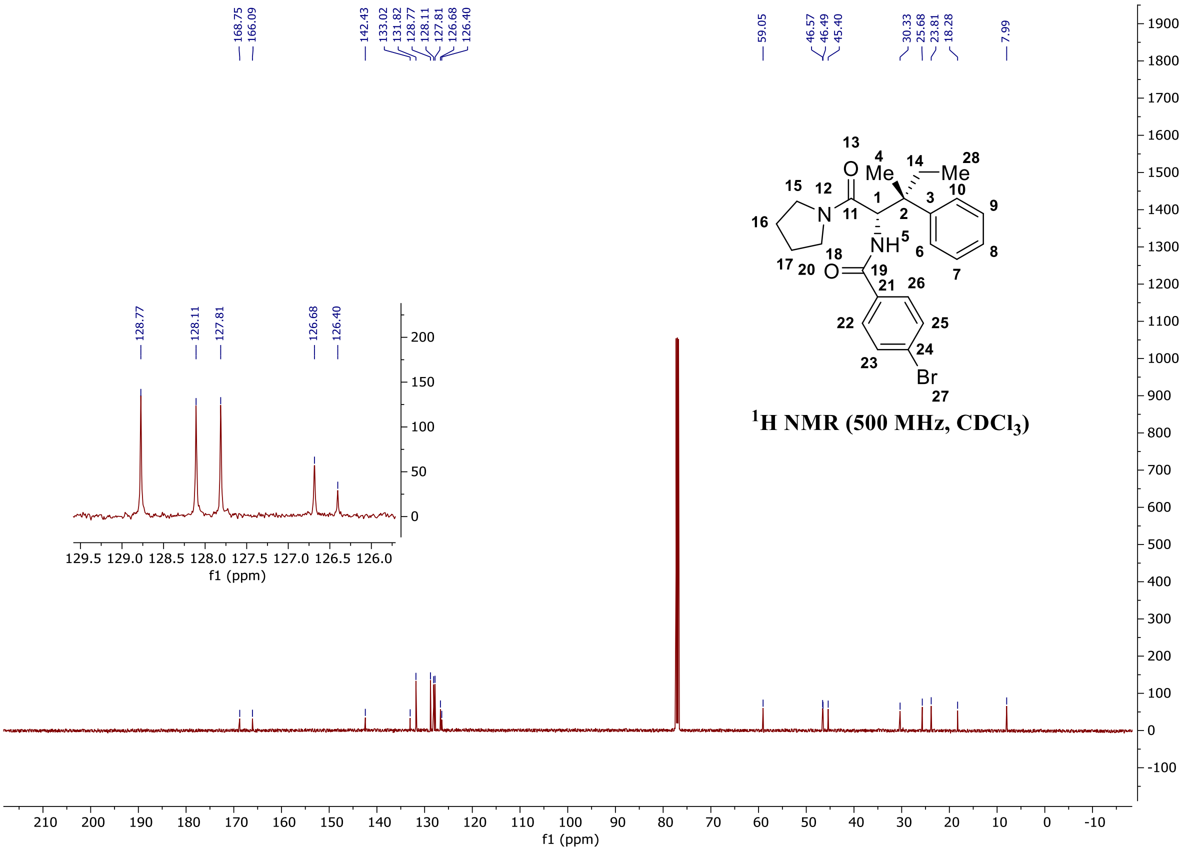

(*S*)-2-Amino-*N*,*N*,3-trimethyl-3-phenylbutanamide (6)

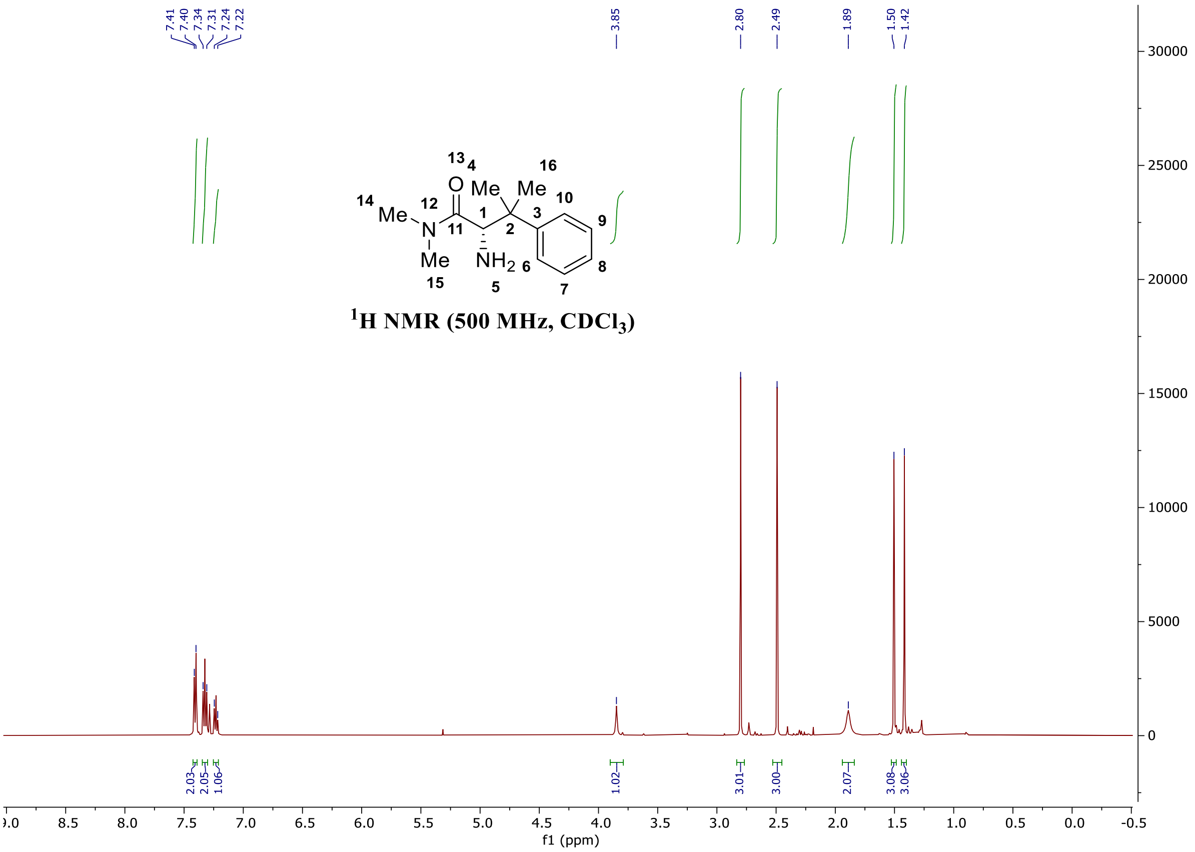

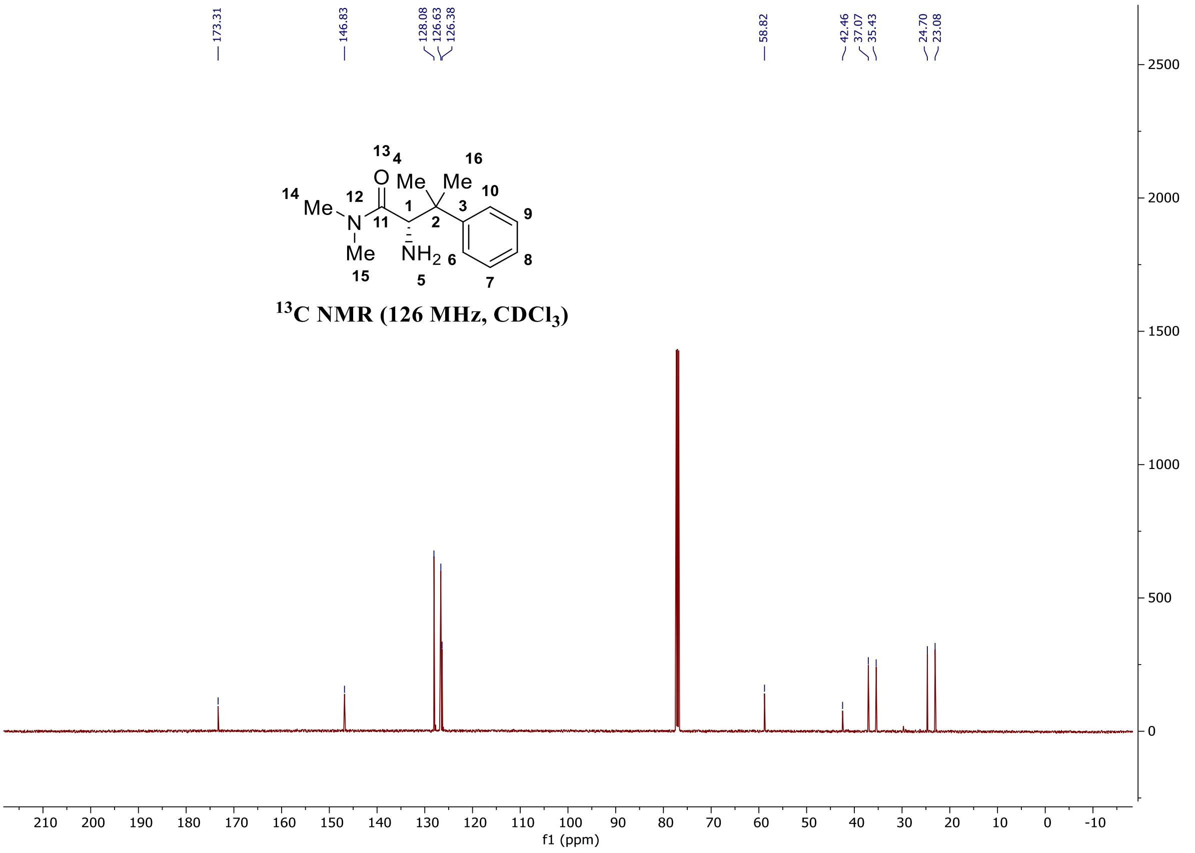

(*S*)-2-Amino-3-methyl-3-phenylbutanoic acid hydrochloride (7)

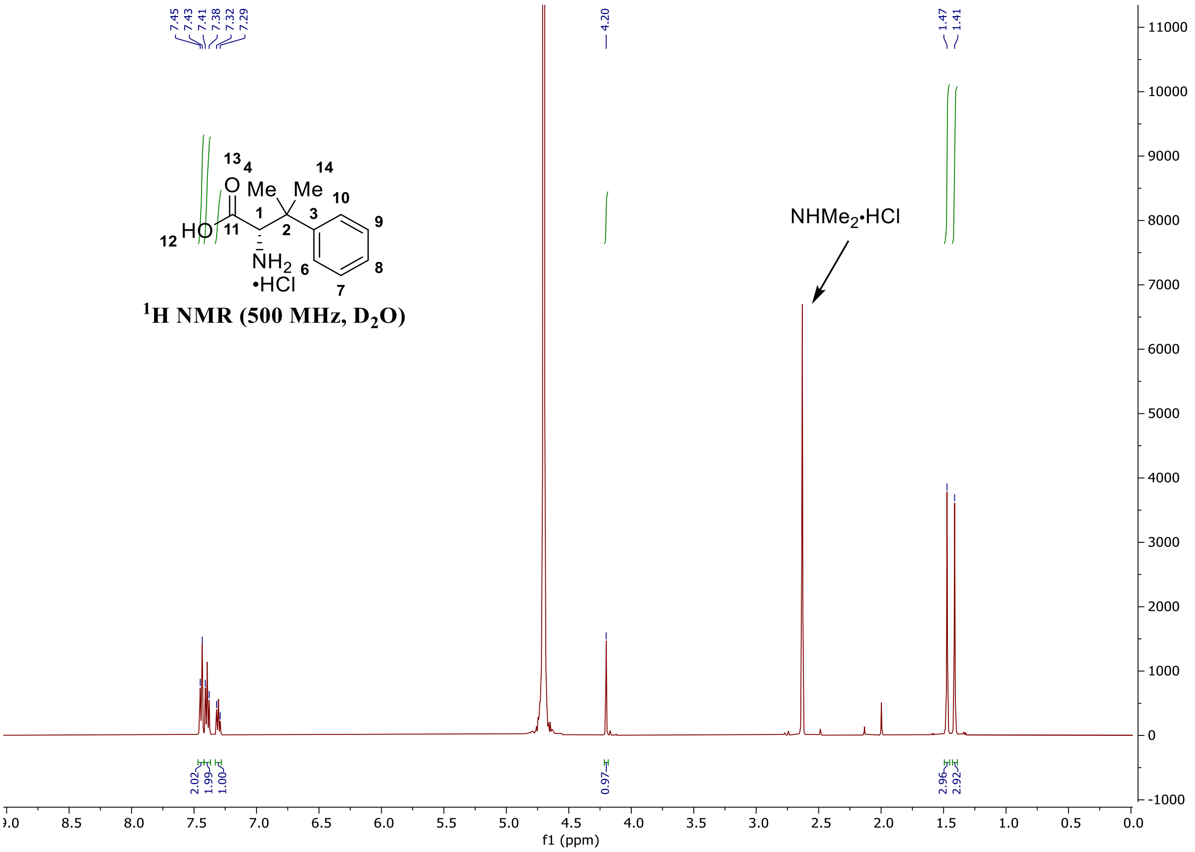

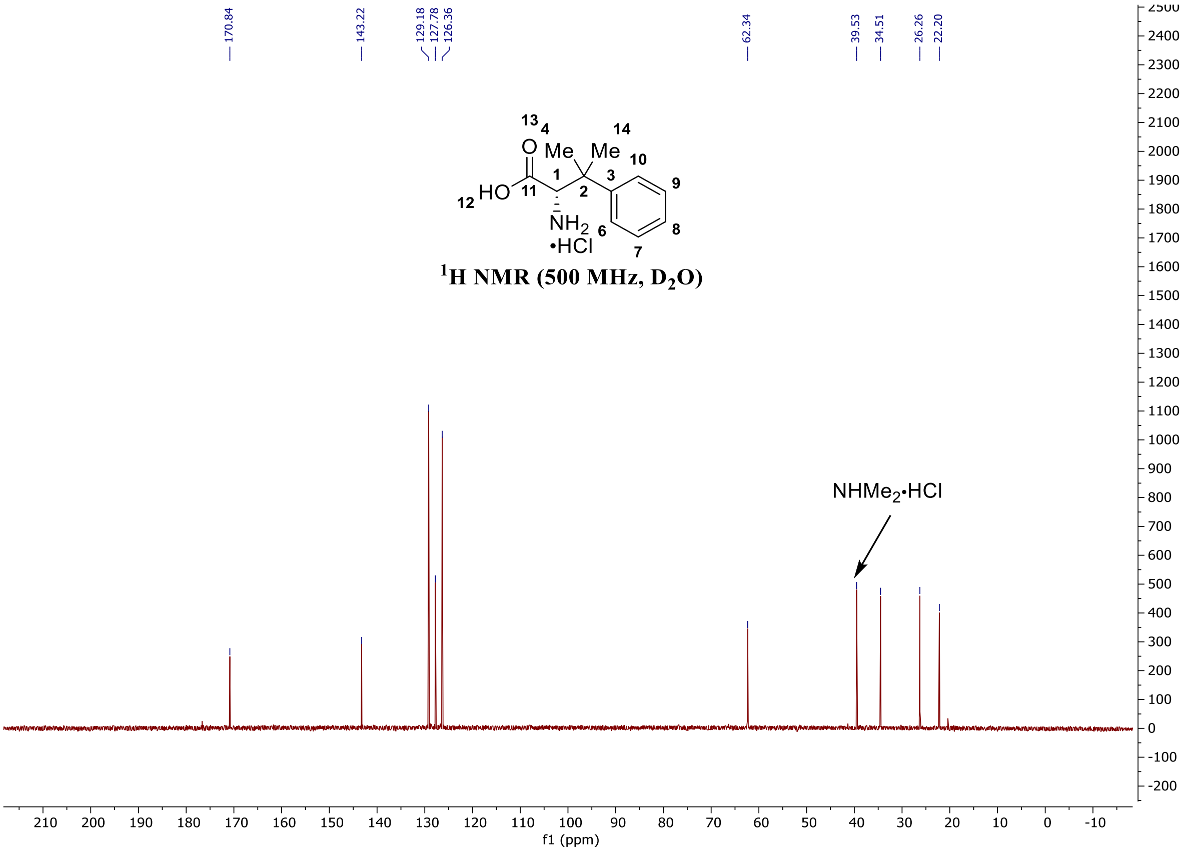

(*S*)-2-Amino-3-methyl-3-phenylbutan-1-ol (8)

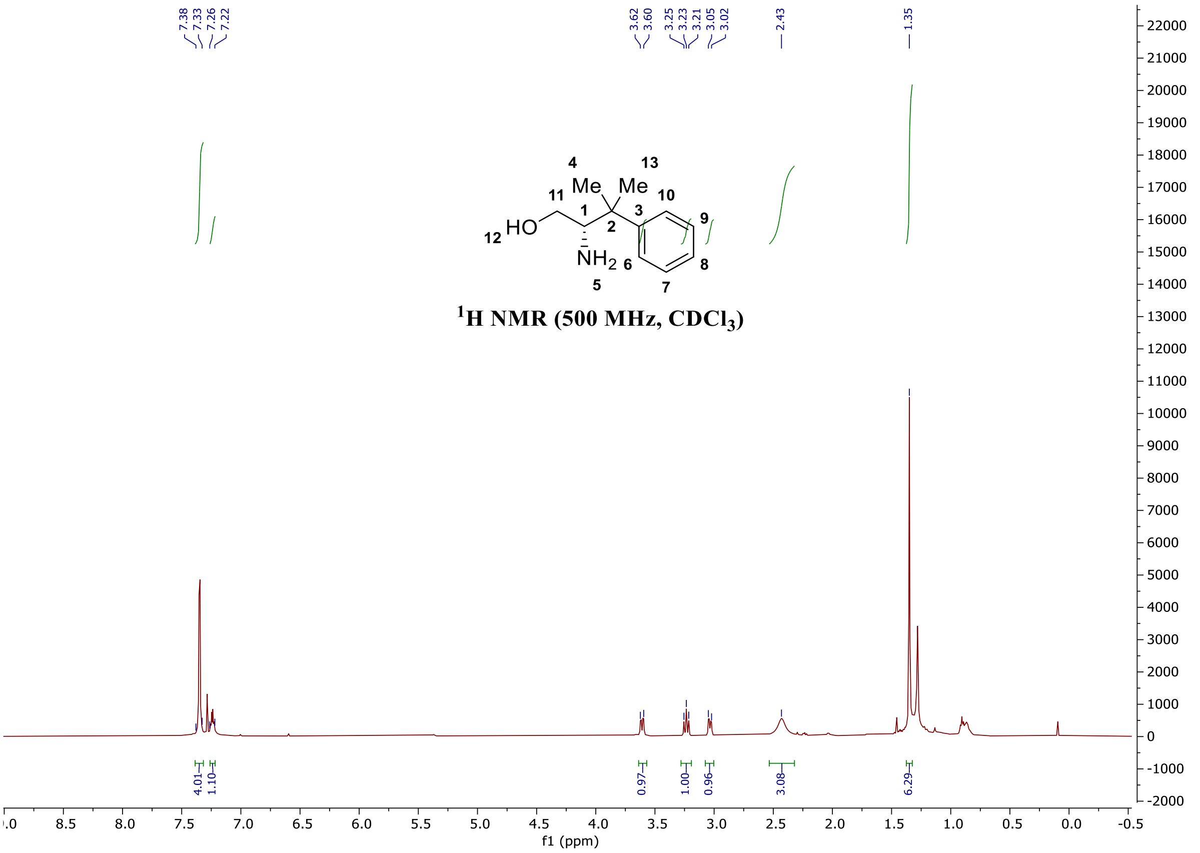

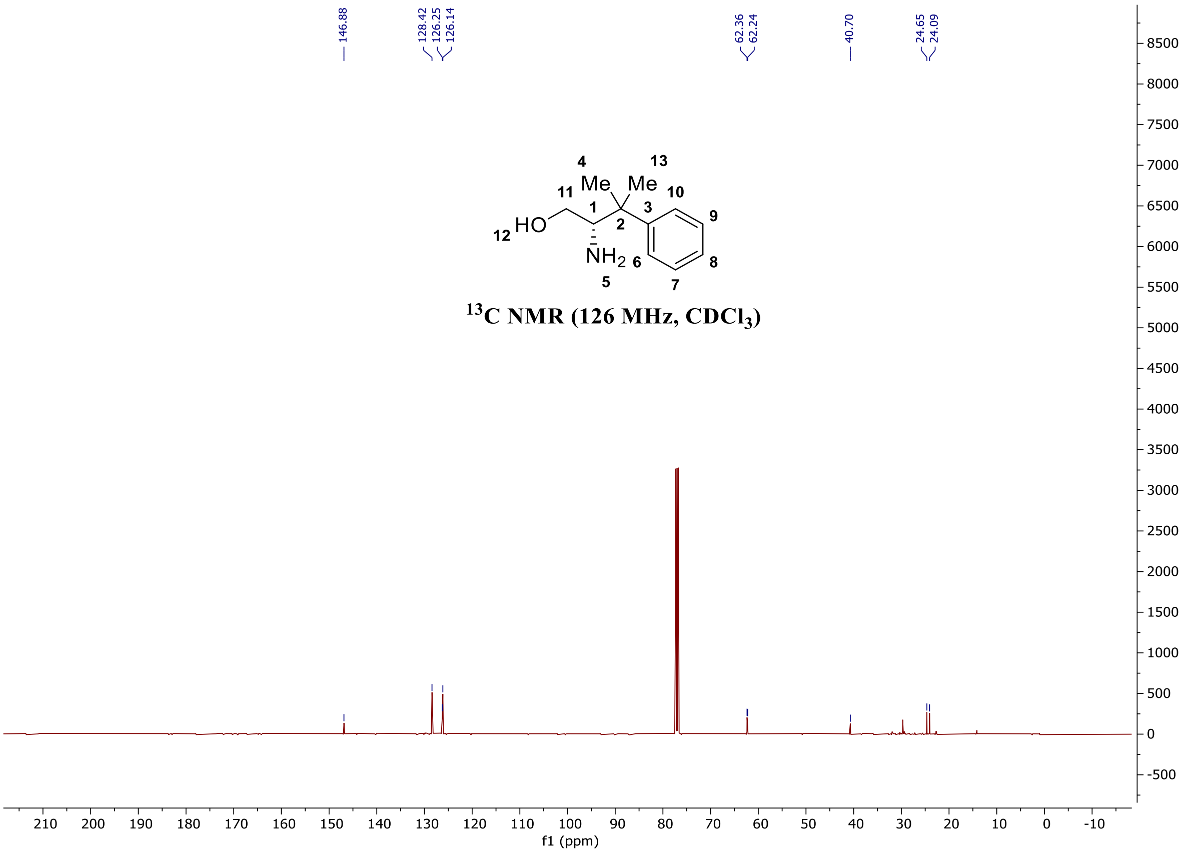

(*S*)-*N*-(1-Hydroxy-3-methyl-3-phenylbutan-2-yl)-4-methylbenzenesulfonamide (8-1)

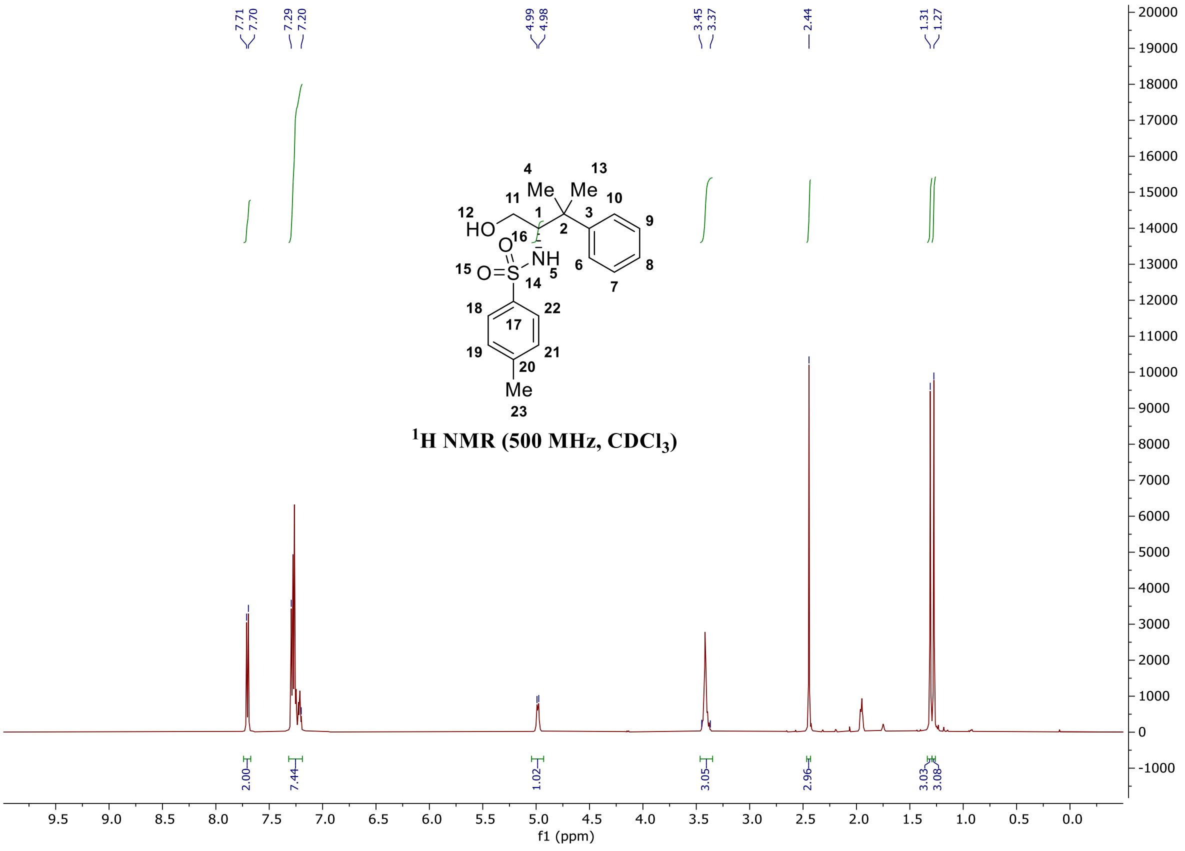

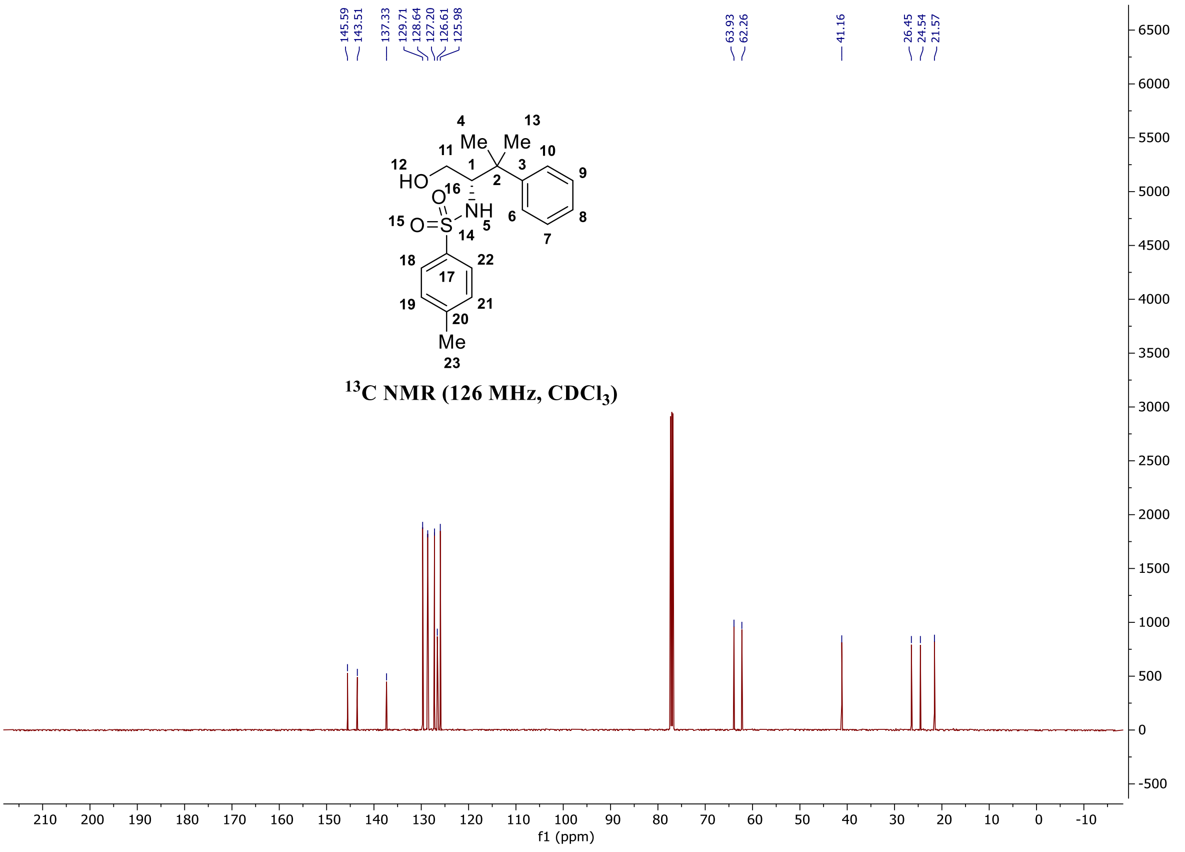

(*S*)-2-Amino-3-methyl-3-(naphthalen-2-yl)-1-(pyrrolidin-1-yl)butan-1-one (9)

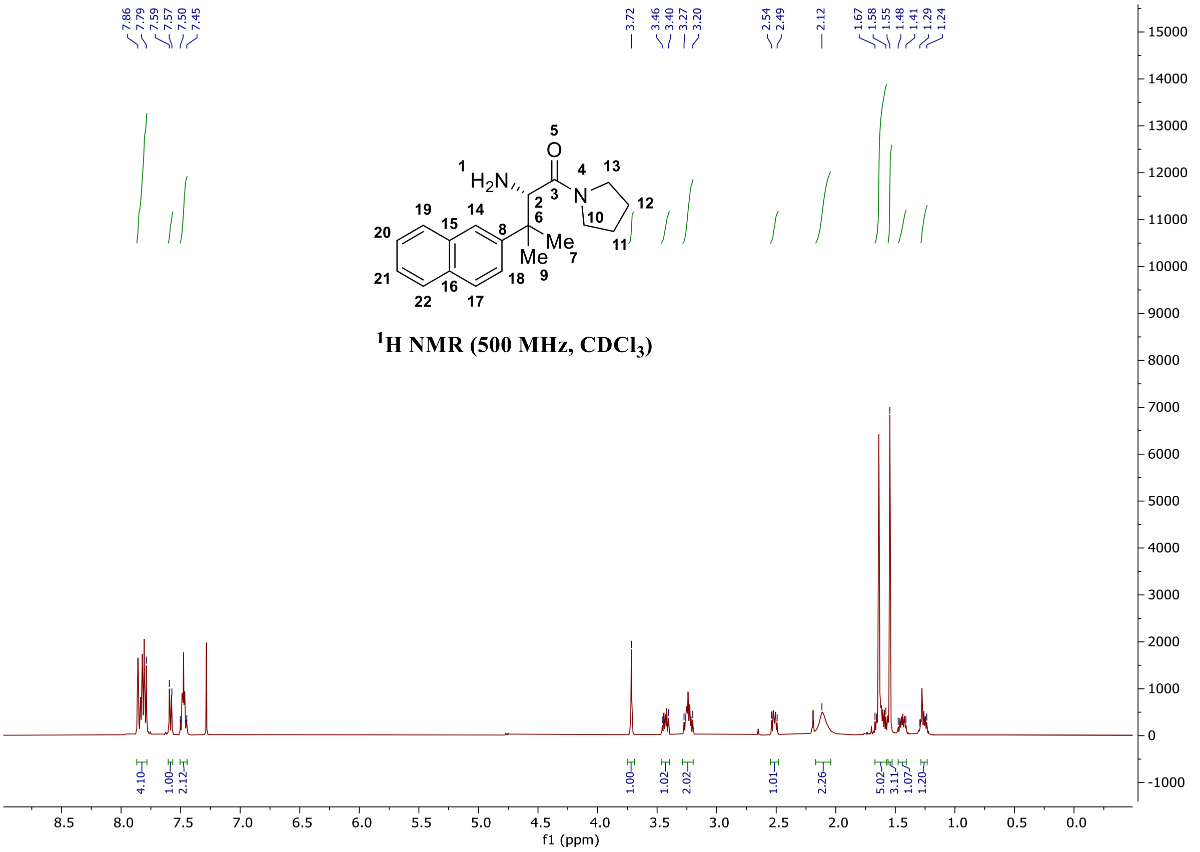

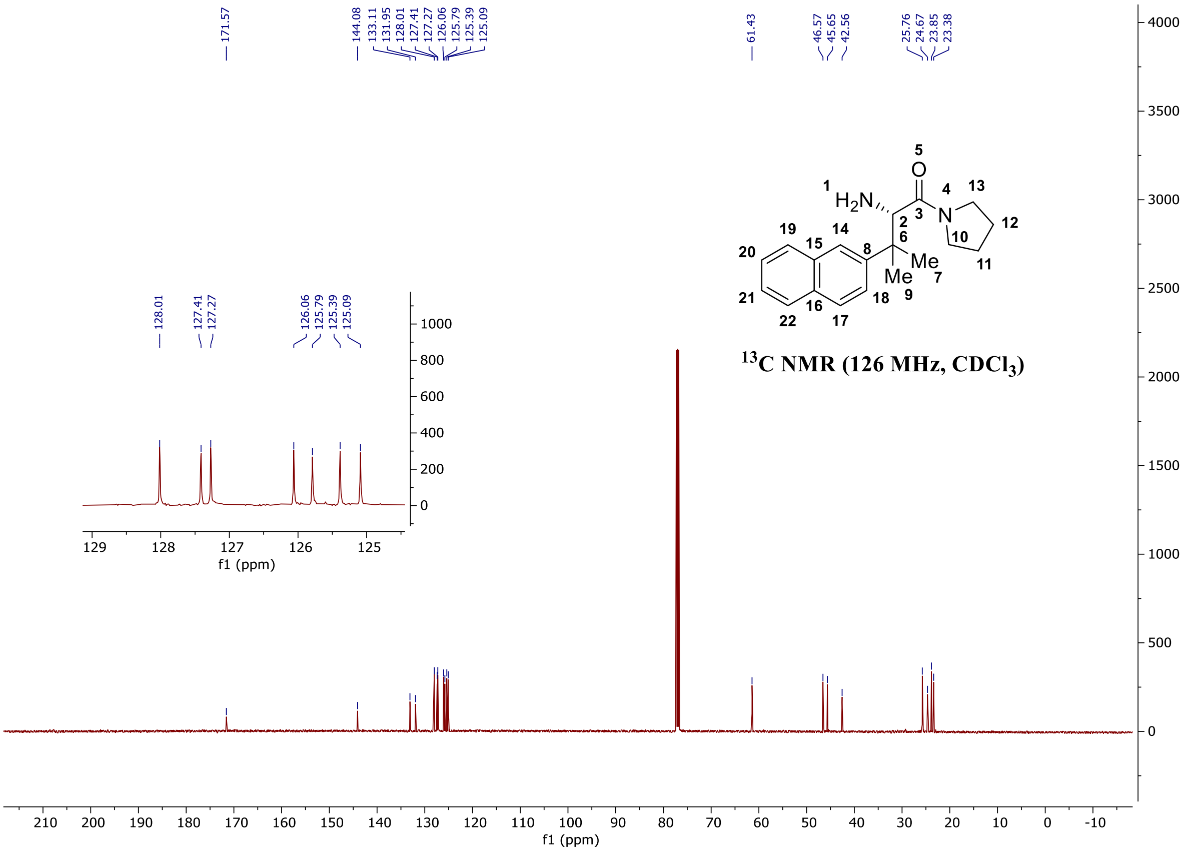

Supplement: Supplementary file 1 — Supporting Information [file ANIE-64-e202504477-s001.pdf]
